# Structure Elucidation, Total Synthesis, Antibacterial In Vivo Efficacy and Biosynthesis Proposal of Myxobacterial Corramycin**

**DOI:** 10.1002/anie.202210747

**Published:** 2022-11-21

**Authors:** Cédric Couturier, Sebastian Groß, Alexander von Tesmar, Judith Hoffmann, Selina Deckarm, Anouchka Fievet, Nelly Dubarry, Thomas Taillier, Christoph Pöverlein, Heike Stump, Michael Kurz, Luigi Toti, Sabine Haag Richter, Dietmar Schummer, Philippe Sizun, Michael Hoffmann, Ram Prasad Awal, Nestor Zaburannyi, Kirsten Harmrolfs, Joachim Wink, Emilie Lessoud, Thierry Vermat, Veronique Cazals, Sandra Silve, Armin Bauer, Michael Mourez, Laurent Fraisse, Corinne Leroi‐Geissler, Astrid Rey, Stéphanie Versluys, Eric Bacqué, Rolf Müller, Stephane Renard

**Affiliations:** ^1^ Evotec 1541, avenue Marcel Mérieux 69280 Marcy L'Etoile France; ^2^ Microbial Natural Products Helmholtz Institute for Pharmaceutical Research Saarland (HIPS) Helmholtz Centre for Infection Research (HZI) and Department of Pharmacy at Saarland University Campus Building E8.1 66123 Saarbrücken Germany; ^3^ Evotec 40 avenue Tony Garnier 69007 Lyon France; ^4^ Sanofi 13, Quai Jules Guesde 94400 Vitry-sur-Seine France; ^5^ Technische Hochschule Mittelhessen Wiesenstraße 14 35390 Gießen Germany; ^6^ Mikrobielle Stammsammlung Helmholtz Centre for Infection Research (HZI) Inhoffenstraße 7 38124 Braunschweig Germany; ^7^ Ecole d'Ingénieurs de Purpan 75 voie du Toec BP57611, 31076 Toulouse France; ^8^ Drug for Neglected Diseases Initiative Chemin Camille-Vidart 15 1202 Geneva Switzerland; ^9^ Charles River Laboratories 327 impasse du domaine rozier 69210 Saint-Germain-Nuelles France; ^10^ Evotec 195, route d'Espagne 31000 Toulouse France

**Keywords:** Antibiotics, Biosynthesis, Corramycin, Myxobacteria, Natural Products

## Abstract

Herein, we describe the myxobacterial natural product Corramycin isolated from *Corallococcus coralloides*. The linear peptide structure contains an unprecedented (2*R*,3*S*)‐γ‐*N*‐methyl‐β‐hydroxy‐histidine moiety. Corramycin exhibits anti‐Gram‐negative activity against *Escherichia coli* (*E. coli*) and is taken up via two transporter systems, SbmA and YejABEF. Furthermore, the Corramycin biosynthetic gene cluster (BGC) was identified and a biosynthesis model was proposed involving a 12‐modular non‐ribosomal peptide synthetase/polyketide synthase. Bioinformatic analysis of the BGC combined with the development of a total synthesis route allowed for the elucidation of the molecule's absolute configuration. Importantly, intravenous administration of 20 mg kg^−1^ of Corramycin in an *E. coli* mouse infection model resulted in 100 % survival of animals without toxic side effects. Corramycin is thus a promising starting point to develop a potent antibacterial drug against hospital‐acquired infections.

## Graphical abstract

Corramycin is a novel natural product produced by the myxobacterium *Corallococcus coralloides*. The structure, extraordinary total synthesis and biosynthesis of the antibiotic is described for the first time. Its promise as an in vivo septicemia model, the absence of cross‐resistance with commonly used antibiotic classes, and the lack of cytotoxicity make Corramycin a potential new drug in the fight against multidrug‐resistant pathogens.

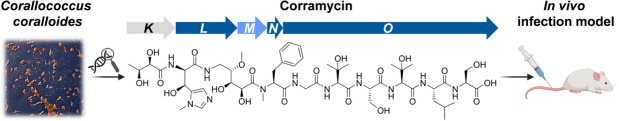

## Introduction

The rise of antimicrobial resistance (AMR) leads to difficult‐to‐treat nosocomial infections and is thus a threat for health care systems globally.[^1^, ^2^] Particularly some multidrug‐resistant (MDR) Gram‐negative pathogens belonging to the bacterial order of *Enterobacterales*, such as *E. coli*, have been ranked as critical priority by the World Health Organization (WHO).[^1^, ^2^] Some strains develop resistance towards almost all antibiotics currently used in the clinic, leaving no remaining treatment options.^[3]^ Most of the antibacterial drugs recently approved by the FDA derive from known antibiotic classes, e.g., the aminoglycoside Plazomicin^[4]^ and the Tetracycline derivative Eravacycline.^[5]^ However, development of resistance to these drugs is highly probable since resistance mechanisms against their congeners do already exist among pathogens. Consequently, it is crucial to discover and develop new antibiotics to fight infectious diseases in the future.^[6]^

Recently, a number of structures under development with new modes of action (MoA) were described, e.g., the Gram‐negative outer membrane biogenesis disrupting compound class of Darobactins,^[7]^ the chimeric peptidomimetic combination of Murepavadin and Polymyxin B_1_,^[8]^ and the small molecule MRL‐494.^[9]^ Another example are the Griselimycins, which are highly active against *Mycobacterium tuberculosis* by inhibiting the new antimicrobial target DnaN, the DNA polymerase sliding clamp.^[10]^

The main source of new structures in the antibiotic drug discovery process are natural products from bacteria, historically in particular from actinomycetes.^[11]^ However, previous screening efforts and recent bioinformatic analyses of the genomes of myxobacteria (Gram‐negative δ‐proteobacteria with the largest known genomes in the bacterial domain), revealed an equal potential for the biosynthesis of natural products.^[12]^ Myxobacterial genomes contain 6–10 % biosynthetic genes in 10–40 different BGCs on average, making them the perfect candidates for future drug screening efforts. Microbial natural products are very often produced by multifunctional enzyme complexes via two major pathways: the polyketide synthases (PKSs) and non‐ribosomal peptide synthetases (NRPSs).^[13]^ The multi‐modularity, functionality and plasticity of PKS and NRPS systems are described in detail elsewhere.^[14]^ Briefly summarized, both systems follow a similar assembly line logic, in which a cascade of condensation reactions links simple monomeric building blocks. *In silico* identification and analysis of BGCs encoding those mega enzymes in bacterial genomes improved genome mining approaches in the past. Moreover, the automated annotation of potential BGCs, the prediction of domain organization or substrate specificities in PKS and NRPS modules[^15^, ^16^] enable the assignment of known metabolites to previously unknown BGCs and predictions of the compound‘s stereochemistry based on DNA sequence analysis.^[17]^ The stereochemistry of amino acids in non‐ribosomal peptides can often be predicted by determination of the functional subtype of a condensation (C) domain.^[16]^ ^L^C_L_ domains catalyze peptide bond formation between two l‐amino acids, whereas ^D^C_L_ domains connect a d‐amino acid with an l‐amino acid.

Herein, we report the discovery of the novel natural product Corramycin which we isolated from the myxobacterium *Corallococcus coralloides* (*C. coralloides*). Structure elucidation via NMR revealed unprecedented structural features such as the (2*R*,3*S*)‐γ‐*N*‐methyl‐β‐hydroxy‐histidine moiety. Corramycin showed antibacterial activity in the low μM range against *E. coli*, including Carbapenem‐, Aminoglycoside‐ and Quinolone‐resistant clinical isolate strains, and no cytotoxicity against mammalian cell lines. Furthermore, after identification and in silico analysis of the BGC, we propose a biosynthesis model by a hybrid NRPS‐PKS assembly line, including the probable presence of a pre‐drug mechanism, and predicted the stereochemistry of 8 out of 13 stereocenters based on the functional subtype of the respective C domains. We also describe the total synthesis of Corramycin, which enabled the elucidation of the absolute stereo configuration, thereby verifying the prediction of the stereocenters. The total synthesis also allowed bypassing the low yields obtained by fermentation. Intravenous administration of Corramycin in mice infected with *E. coli* resulted in 100 % survival of all test animals after four days when administering at 20 mg kg^−1^ on the first day, importantly, without toxic side effects observed. Finally, we provide evidence that Corramycin showed a much lower frequency of resistance (FoR) using minimal rather than rich media which is suggested to be due to the uptake of Corramycin by two transporter systems in *E. coli*, SbmA and YejABEF.

## Results and Discussion

**Figure 1:**
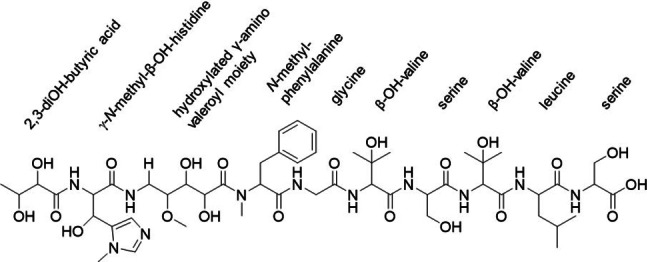
Two‐dimensional structure of Corramycin.

To screen for new anti‐infective natural products, we fermented numerous myxobacteria in liquid media in shake flasks, independently, and analyzed their production profile using uHPLC‐*hr*MS. One strain, *C. coralloides* ST201330 produced a compound with a *m/z* [M+H]^+^ value of 1184.56, which could not be matched to known natural products in SciFinder and DNP database searches. Thus, we purified this natural product that was named Corramycin (**1**) from the fermentation broth of ST201330 shake flask cultures. Notably, we also identified another strain, *C. coralloides* MCy10984, producing the same compound. Detailed analysis of the 1D and 2D NMR spectra revealed the two‐dimensional structure of Corramycin (Figure 1; Supporting Information section 1).

Corramycin exhibits a linear peptidic structure with eight α‐amino acids including several unique structural features such as the unprecedented γ‐*N*‐methyl‐β‐hydroxy‐histidine, a 5‐amino‐2,3‐dihydroxy‐4‐methoxy valeroyl moiety and an *N*‐terminal dihydroxy butyric acid moiety. The supplementation of isotope‐labelled precursor building blocks of Corramycin during the fermentation process coupled with uHPLC‐*hr*MS analysis additionally enabled the identification of several minor Corrramycin analogues, including des‐methylated (**2**), hydroxylated (**3**), double‐hydroxylated (**4**), and hydroxylated plus glycosylated (**5**) Corramycins (SI Figure S2 and Table S3).

To assess the antibacterial activity of Corramycin, the in vitro minimal inhibitory concentrations (MICs) against several *E. coli* strains and some other important human pathogens were determined. Good antibiotic activity was observed against *E. coli* ATCC35218 with a MIC of 1–4 μg mL^−1^, but also against additional *E. coli* strains, including MDR ones, and *Salmonella typhimurium* (*S. typhimurium*) (4 μg mL^−1^) (Table 1).

**Table 1:**
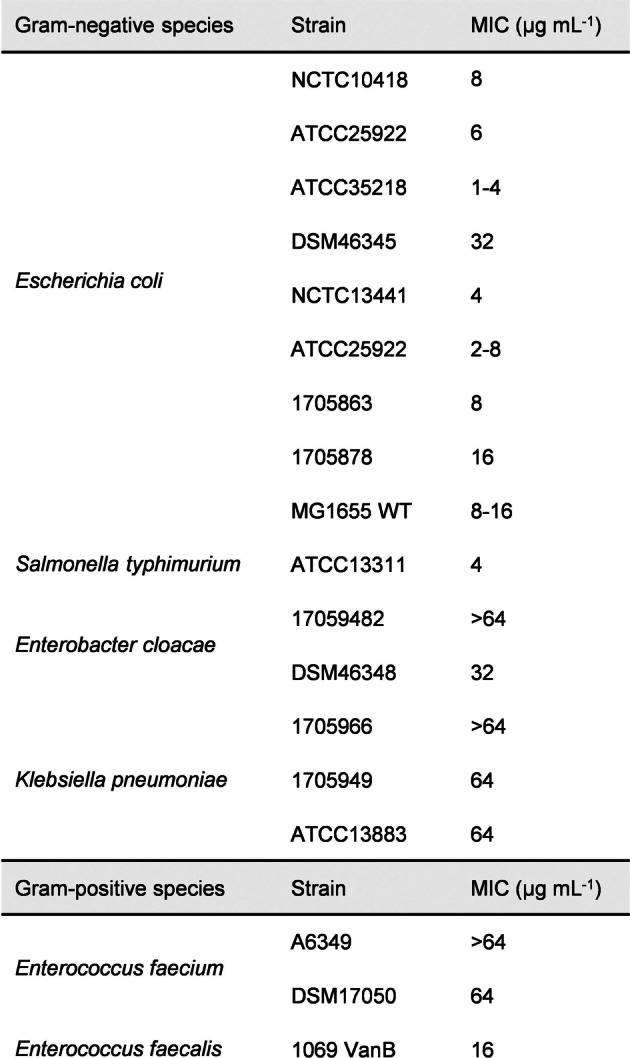
MIC of Corramycin (**1**) against selected Gram‐negative and Gram‐positive pathogens.

However, Corramycin showed only weak or no activity against several other clinically relevant Gram‐positive and Gram‐negative pathogens such as *Staphylococcus aureus*, *Enterococci*, *Pseudomonas aeruginosa* and *Klebsiella pneumoniae* (*K. pneumoniae*) (Supporting Information Table S4). To investigate if Corramycin exhibited a different MoA as compared to the antibiotics classes commonly used in the clinic, we assessed the MICs of Corramycin, Tobramycin, Cefotaxim, Cefoxitin, Ciprofloxacin, Tetracycline, and Rifampicin against 16 clinical isolate *E. coli* strains, which are classified Carbapenem‐resistant, Aminoglycoside‐resistant, or Quinolone‐resistant (Supporting Information Table S5). Notably, we could not observe cross‐resistance to any of the other antibiotics.

We next aimed for the identification of the corresponding BGC to gain insights into the biosynthesis and the stereochemistry of the unique structure of Corramycin. Considering the peptidic structure combined with the N‐terminal dihydroxy butyric acid and the 5 ‐amino‐2,3‐dihydroxy‐4‐methoxy valeroyl moiety, we assumed that Corramycin was produced by a hybrid NRPS‐PKS. Thus, we sequenced the genomic DNA of both producer strains and screened for the BGC in the genomes, eventually identifying five streamlined core genes, *comKLMNO*, encoding a 12‐modular hybrid NRPS‐PKS assembly line (Supporting Information Table S6). To identify additional genes putatively belonging to the BGC, such as genes encoding tailoring enzymes, we determined the BGC borders by comparing the genomic regions surrounding *comKLMNO* in both producer strains with the phylogenetically closely related non‐producer strain *C. coralloides* DSM2259. Fifteen putative genes were identified that presumably belong to the Corramycin BGC (Supporting Information Figure S23).

**Figure 2:**
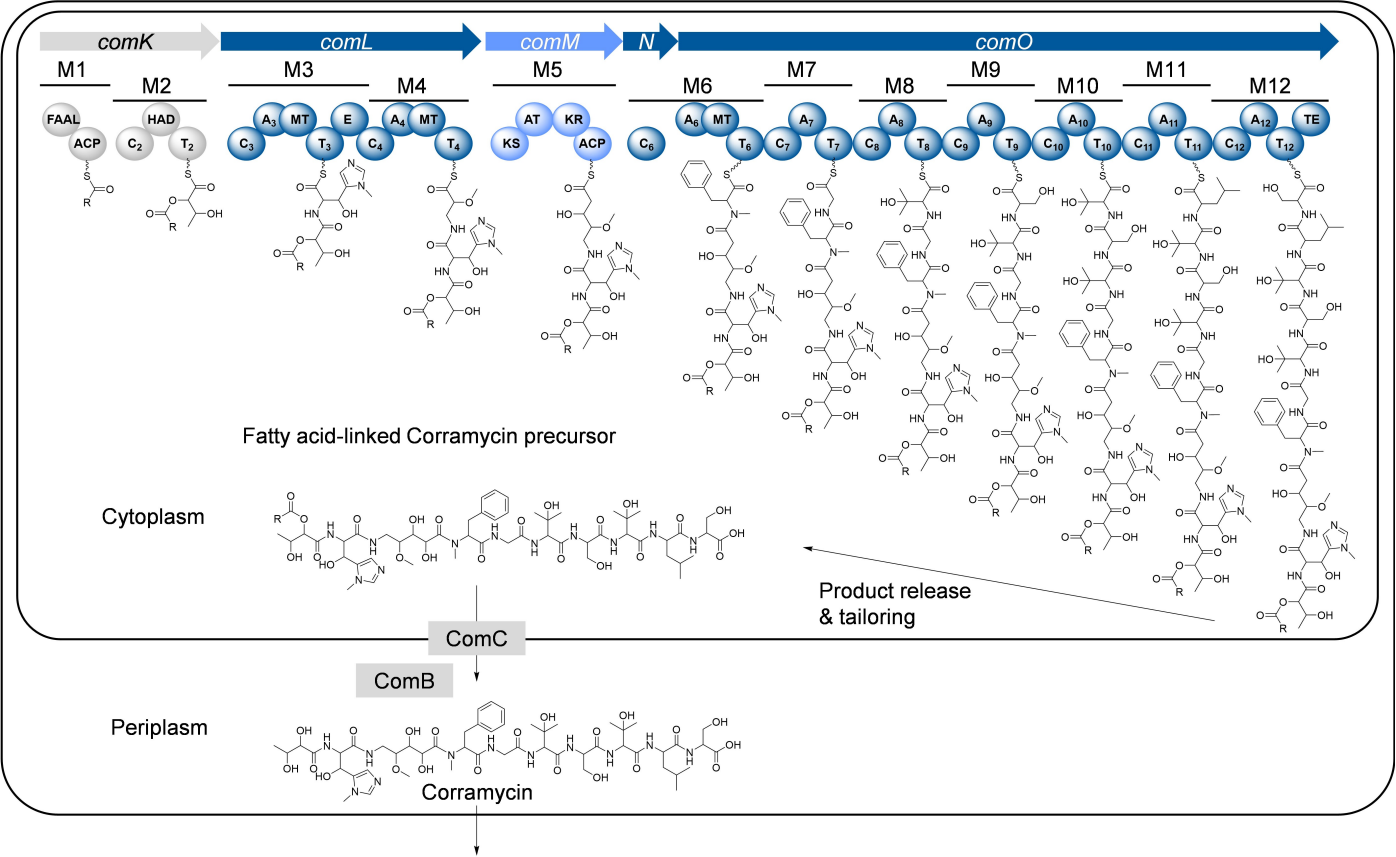
Proposed biosynthesis of Corramycin. The 12‐modular NRPS‐PKS assembly line produces a fatty acid‐linked Corramycin precursor which is hypothesized to be deacylated and transported into the periplasm by ComC and ComB prior to export by an unknown mechanism. FAAL: fatty‐acyl AMP ligase; ACP: acyl carrier protein; C: condensation domain; HAD: haloacid dehalogenase; T: thiolation domain; A: adenylation domain; MT: methyltransferase domain; E: epimerization domain; KS: ketosynthase; AT: acyl transferase; KR: ketoreductase; TE: thioesterase domain.

Based on the in silico analysis of the putative genes involved in the Corramycin biosynthesis and feeding experiments with isotope‐labelled precursors, we were able to propose a model for the biosynthesis (Figure 2, Supporting Information Table S3).

The first module of the assembly line, encoded by *comK*, contains a fatty‐acyl AMP ligase (FAAL), a member of the adenylate forming enzymes. FAALs are often associated with NRPS/PKS pathways as a starter unit catalyzing the acylation of the first building block.^[18]^ Since no acylated Corramycin analogs were detected in the fermentation broth extracts, we aimed to examine if the FAAL domain was indeed active. Therefore, we heterologously overexpressed and purified the FAAL domain from *E. coli* BL21 (DE3) and performed a malachite green assay.^[19]^ We observed activation of numerous linear fatty acids with different lengths by the FAAL domain (Supporting Information Figure S26, section 9). However, even upon re‐inspection searching for acylated Corramycin derivatives with the respective fatty acid chain lengths we did not find fatty acid‐linked Corramycin derivatives in the extracts of the producer strains. In the biosynthesis of the myxobacterial lipopeptide Vioprolide, an inactive, fatty acid‐linked precursor is biosynthesized and the maturation of the active product is achieved by hydrolysis of the fatty acid.^[20]^ Since the Corramycin FAAL domain is evidently active, we speculate that a similar mechanism applies for the biosynthesis of Corramycins. A hypothetical pre‐drug mechanism in Corramycin maturation could include ComB and ComC, a putative hydrolase and flippase‐like exporter, respectively. The latter protein shows significant homology with glycosyl transferases. Since both proteins harbor an *N*‐terminal peptide signal for periplasmic localization, the membrane‐bound ComC might export the acylated Corramycin precursors to the periplasm, where hydrolysis by ComB potentially facilitates maturation to the final, active product. However, further investigations are required in future experiments.

The second module in Corramycin biosynthesis, also encoded by *comK*, harbors an Fkbh‐like domain belonging to the haloacid dehalogenase (HAD) superfamily. The same architecture of the first two modules (FAAL‐ACP‐C‐HAD‐T) was described for the Vioprolide assembly line, in which an unusual esterification process leads to the incorporation of a glycerate building block.^[21]^ However, Corramycins harbor an *N*‐terminal *C‐*4 butyric acid moiety and glycerate only contains three *C‐*atoms. Thus, we assume that a glycerate moiety is incorporated and subsequently *C*‐methylated in Corramycin biosynthesis. Based on the feeding of d_3_‐methionine during fermentation of *C. coralloides* MCy10984 with subsequent analysis using LC–MS (Supporting Information section 4), we propose this reaction to be performed by ComJ, a radical‐SAM methyltransferase.

One intriguing chemical feature of Corramycins is the unprecedented γ‐*N*‐methyl‐β‐hydroxy‐histidine moiety. Based on the architecture of module 3 including a putative *N*‐methyltransferase (*N*‐MT) domain and an epimerization (E) domain, we assume that l‐histidine is activated by the A domain, *N*‐methylated by the *N*‐MT domain and subsequently epimerized to γ‐*N*‐methyl‐d‐histidine by the E domain. To our knowledge no NRPS‐associated *N*‐MT domain acting on the side chain of an aromatic amino acid was described before. On the contrary, the biosynthetic pathway for the β‐hydroxylation of histidine has been described. NikQ, a heme‐protein, has been shown to facilitate the β‐hydroxylation of histidine in Nikkomycin biosynthesis.^[22]^ Interestingly, free histidine is not accepted and has to be loaded onto a T domain by a respective A domain, before it is recognized by NikQ. However, we could not identify any cytochrome P450 dependent enzyme or other heme‐containing proteins in the Corramycin BGC. Thus, we assume a different mechanism taking place in Corramycin biosynthesis that must be addressed in future experiments.

Another unique feature of Corramycins is the unusual 5‐amino‐2,3‐dihydroxy‐4‐methoxy valeroyl moiety. Based on the in silico analysis of modules 4 and 5 coupled with feeding experiments (Supporting Information Table S3), we propose that aspartate is decarboxylated by ComE to β‐alanine, which is incorporated by module 4, hydroxylated, methylated *in cis* by an *O‐*methyltransferase domain, and finally extended by a *C*‐2 body by the type I PKS module 5.

Interestingly, two β‐hydroxy‐valines are found in Corramycins, which is a rarely observed building block in natural products. Independent supplementation of d_8_‐l‐valine, d_6_‐l‐hydroxy‐valine and ^13^ *C*_4_‐^15^ *N*‐l‐threonine during fermentation showed that only valine was accepted by the NRPS (Supporting Information Figure S22 and Table S3). Consequently, the hydroxylation of this building block occurs either on‐line or after product release. The β‐hydroxy‐valine moiety was also found in the myxobacterial non‐ribosomal peptide Myxoprincomide,^[23]^ however, its biosynthetic origin has thus far not been identified. For Corramycin biosynthesis further investigations are necessary to clarify the biosynthetic origin of β‐hydroxy‐valine, e.g., by the independent deletion of genes encoding putative hydroxylases.

**Figure 3:**
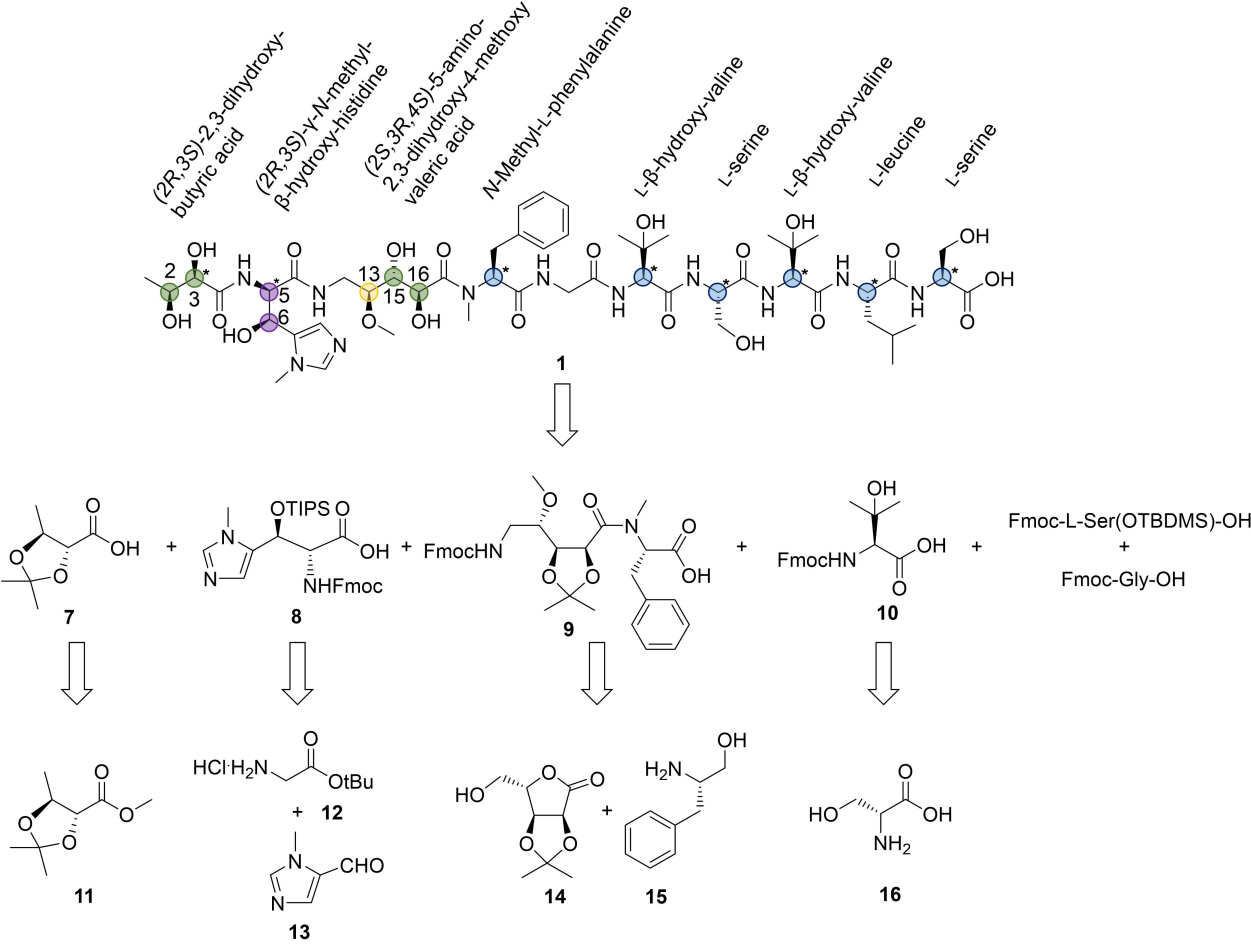
Absolute configuration and retrosynthetic analysis of Corramycin (**1**). The configuration of the thirteen stereocenters of Corramycin is given. Blue labelling: Stereocenters elucidated via hydrolysis and chiral GC/MS. Green labelling: relative configuration elucidated via acetonide synthesis and ROESY NMR. Yellow labelling: prediction of the relative configuration via partial synthesis, elucidation via total synthesis of Corramycin) and NMR spectra comparison. Violet labelling: elucidation via total synthesis of Corramycin (**1**) and NMR spectra comparison. Labelling with a star: predicted stereocenters after the in silico analysis of the BGC.

Since the Corramycin production titer (<0.1 mg L^−1^) by fermentation was low and the knowledge about optimal strain cultivation, production scale‐up procedures as well as genetic manipulability of the natural producer were limited, we aimed to develop a total synthesis route. However, structure elucidation by NMR only revealed at that point the two‐dimensional structure of Corramycin (Figure 1). First, we performed an in silico analysis of the module and domain architecture of the Corramycin assembly line which allowed to predict the configuration of eight stereocenters (labelled with a star in Figure 3). The HAD (FkbH) domain was proposed to load and dephosphorylate d‐1,3‐bisphosphoglycerate and was experimentally shown to activate d‐3‐phosphoglycerate by the Vioprolide FkbH domain.^[21]^ Consequently, we proposed an *S* configuration for position 2 in Corramycin. The analysis of all C domain subtypes revealed an ^L^C_L_ architecture for the C domain of module 3 (histidine) and a ^D^C_L_ architecture of the C domain of module 4. Since module 3 harbors an E domain, we postulate that l‐histidine is activated by module 3 and subsequently epimerized to a d‐histidine, which leads to an *R* configuration at the position 5 in Corramycin. The C domain subtypes of modules 6 and 8–12 were determined as ^L^C_L_ subtype and no additional E domains were identified. Consequently, we proposed the *S* configuration for phenylalanine, both β‐hydroxy‐valines, both serines and leucine. Since the remaining five stereocenters biosynthetically either arose by hydroxylation through tailoring enzymes or by downstream PKS modifications, we were not able to predict configurations due to a lack of bioinformatics tools. To fully elucidate the stereochemistry of Corramycin (**1**), we performed a total hydrolysis followed by chiral GC/MS analysis of the obtained amino acids. The hydrolysis revealed the absolute configuration of six stereocenters (labelled in blue in Figure 3). The relative configuration of the two *vic‐*dihydroxy units was elucidated via acetonide synthesis and ROESY NMR experiments (acetonide **6**, Supporting Information Figures S18–S20).

Based on this analysis, we started to synthesize all possible remaining 16 diastereomers of Corramycin to compare the respective NMR data with those of the isolated natural product. A solid‐phase peptide synthesis (SPPS) approach with a traditional Fmoc‐strategy and adequate protecting groups on the different hydroxyl groups was used to synthesize Corramycin starting from l‐serine. The non‐proteinogenic protected amino acids β‐hydroxy‐valine (**10**), 2,3‐dihydroxybutyric acid (**7**), *N*‐methyl‐β‐hydroxy‐histdine (**8**), and 5‐amino‐2,3‐dihydroxy‐4‐methoxyvaleric acid (**9**) were synthesized from commercially available building blocks as shown in the retrosynthetic scheme (Figure 3). Building block **10** was easily accessible by using a described strategy starting from d‐Serine (Supporting Information Figure S28) as well as building block **7**, which was obtained via saponification of commercially available ester **11** (Supporting Information Figure S31).

More steps were necessary for the synthesis of building block **9** (Supporting Information Figure S29), starting from a protected and commercial derivative of l‐ribose (**14**). Transformation of the free alcohol as leaving group, followed by azido formation, lactone opening with a protected phenylalanine derivative (**15**) and several protection‐, oxidation‐ and reduction‐steps allowed to obtain building block **9** in an overall acceptable yield. For the last building block **8**, we achieved to create the two stereocenters by enantioselective aldolisation using a titanium enolate derived from a chiral iminoglycinate (Supporting Information Figure S30). Using α‐pinene as a chiral auxiliary, an oxidation step followed by imine formation with protected glycine (**12**) delivered the starting compound for the aldolization step, followed by a few protection/deprotection steps. The synthesis route was finalized via a traditional Fmoc‐SPPS in standard conditions (HATU, DIEA, DMF, room temperature) starting from Fmoc‐l‐TBDMSO‐serine (Supporting Information Figure S32). Comparison of the NMR data of all 16 diastereomers with the NMR data of the isolated Corramycin confirmed the configurational correctness of the analysis and the in silico prediction. Furthermore, we elucidated the configuration of the missing stereocenters, providing the absolute configuration of Corramycin (Figure 3 (**1**)). The complete total synthesis is described in detail in the Supporting Information.

After establishing a total synthesis route to avoid dependance on the low yield fermentation process and to elucidate the absolute configuration of Corramycin, we planned to further investigate the potential of Corramycin as a starting point for the development of a new antibacterial drug. Therefore, we first performed a time‐kill curve experiment at 4x and 8x MIC of Corramycin using *E. coli* ATCC25922 cultivated in the MHB rich medium or in the M9 minimal medium, (Supporting Information Figure S71). This experiment revealed that Corramycin is rapidly bactericidal, independent of the medium used. However, we observed re‐growth in MHB after 6 hours, indicating rapid selection of mutants resistant to Corramycin under these conditions. Therefore, we performed a FoR experiment using *E. coli* ATCC25922 in MHB and M9 medium at 4x MIC of Corramycin. Importantly, the FoR was much higher in MHB medium (3.8×10^−6^) compared to M9 medium (3×10^−10^) (Supporting Information Table S9). Next, we sequenced the genome of the resistant *E. coli* clones cultivated in MHB medium to identify potentially responsible mutations.

A frameshift in the gene encoding the SbmA transporter gave a first hint about the bacterial uptake systems of Corramycin since the potential inactivation of SbmA, caused by the frameshift, may confer resistance towards Corramycin. Interestingly, clones harboring the mutated SbmA were only resistant towards Corramycin in MHB medium but not in M9 medium. Thus, we performed an additional resistance experiment with SbmA mutants in M9 medium. We sequenced the genome of clones which gained resistance towards Corramycin in M9 medium and found additional mutations (frameshifts) in YejE or YejF. Independent or combined deletions of *sbmA* and *yejEF* in *E. coli* ATCC25922 by λ red‐mediated homologous recombination (Supporting Information section 13) resulted in strains that showed cultivation medium‐dependent resistance towards Corramycin (Table 2). The uptake appears to be dependent solely on the SbmA transporter in MHB medium and on both SbmA and YejEF transporters in M9 medium. Functional complementation of SbmA and YejEF in *E. coli* ATCC25922 Δ*sbmA*/Δ*yejEF* revealed that solely SbmA restores the susceptibility to Corramycin in MHB medium.

**Table 2:**
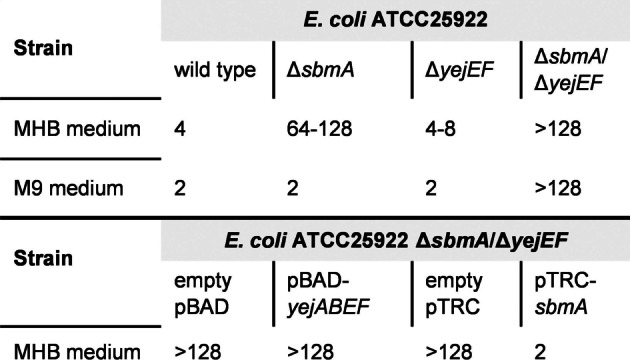
Susceptibility of *E. coli* towards Corramycin after deletion and complementation of SbmA or YejEF. pBAD and pTRC are cloning and expression vectors used for the complementation experiments. MIC values are given in μg mL^−1^.

One recognized bottleneck for the identification of new anti‐Gram‐negative scaffolds is the difficulty for compounds to penetrate the both the outer and inner membranes due to their differing properties.^[24]^ Corramycin is able to hijack several bacterial transporters in a seemingly redundant manner. SbmA is an ABC transporter shown to be responsible for the uptake of antibacterials such as Microcin J25^[25]^ and Bleomycin.^[26]^ SbmA is present in *E. coli* and *K. pneumoniae* but absent in other *Enterobacteriaceae*. where an ortholog can be identified under the name of YddA.^[27]^ YejABEF is an inner membrane ABC transporter, which is conserved in Gram‐negative bacteria and mediates the uptake of microcin C.^[28]^ Herein, both transporters, SbmA and YejABDEF, have been shown to play a vital role in the uptake of Corramycin with SbmA being the main uptake system when *E. coli* was cultivated in MHB medium.

Since the transport and accumulation of compounds in bacteria is one of the major bottlenecks in antibiotic development, potential drugs hijacking only a single transporter make quick resistance development likely, which was, e.g., shown for Fosfomycin,^[29]^ Kazugamycin^[30]^ and GE81112.^[31]^ This was also shown here, when SbmA is the only transporter leading to Corramycin uptake in MHB medium. The high FoR against Corramycin could have been an exclusion criterion for the further development of Corramycin towards a drug. However, the FoR was much lower in M9 minimal medium, where two transporter systems had to be mutated to decrease Corramycin susceptibility.

**Figure 4:**
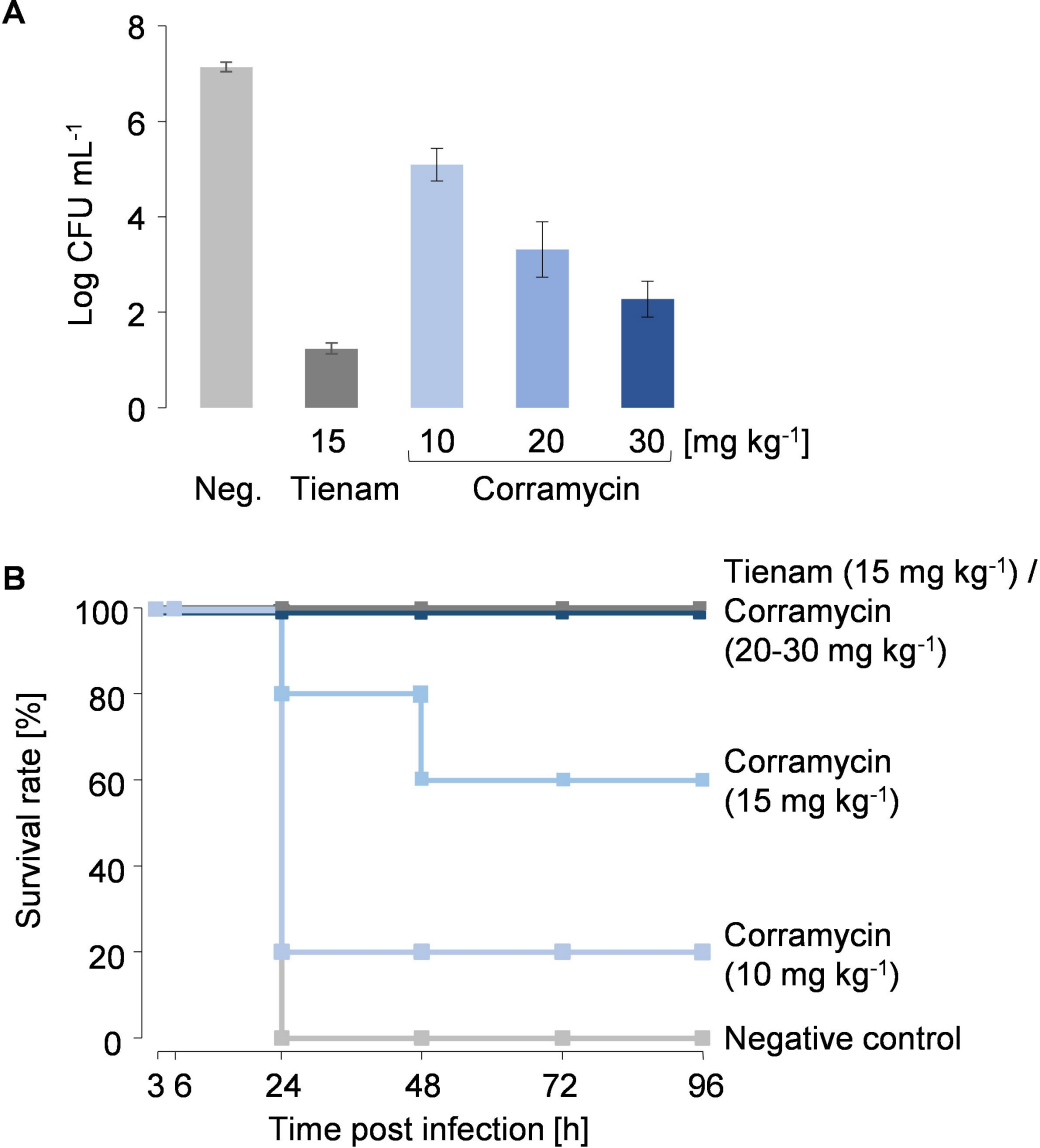
In vivo antibacterial activity of Corramycin in infected mice. A) Colony forming unit (CFU) load of *E. coli* ATCC35218 in blood 4 hours post infection with 2.10^7^ CFU. 5 mice per dose were used. Neg.: administration of 0.9 % NaCl (negative control, light grey bar); Tienam: Imipenem/Cilastatin treatment (positive control, dark grey bar); Corramycin treatment with different doses (light blue, blue and dark blue bars). B) Survival rate [in %] of 10 mice per dose 3, 6, 24, 48, 72 and 96 hours post infection.

As bacterial resistance development in minimal media is thought to be more mimetic to an actual infection in vivo, we decided to test the in vivo efficacy of Corramycin in infected mice, despite the poor FoR experiment results in the rich medium. The mice were first infected intraperitoneally with *E. coli* ATCC35218 and subsequently treated with different doses of Corramycin ranging from a total of 10 to 30 mg per kg body weight divided into two doses, 1 hour and 3 hours after infection. We observed a remarkable dose‐dependent reduction of the bacterial load 4 hours after infection resulting in survival of the mice up to 100 % after 4 days starting from the 20 mg kg^‐1^ total dose (Figure 4). In comparison, 0 % of survival was observed for the control mice, which were treated with the vehicle substance.

Of note, Corramycin is rapidly eliminated after *i. v*. administration, keeping the plasma concentration above the MIC for less than 30 minutes. However, despite this rapid elimination, the antibacterial effect was strong enough in the animals to achieve survival after a single day of treatment.

Considering the two potential Corramycin uptake transporters SbmA and YejEF, we investigated the susceptibility of *E. coli* ATCC25922 with independent or combined deletion of those transporters in the animal model. The deletion of SbmA and YejEF in *E. coli* resulted in a mild decrease of susceptibility to Corramycin, affording ED_50_s (effective dose for 50 % of the population) of 23.3 mg kg^−1^ and 21.4 mg kg^−1^, respectively, compared to the wild type control strain (6.1 mg kg^−1^) (Supporting Information section 14.3). The double deletion of SbmA and YejEF, however, lead to a significant increase in resistance with an ED_50_ of >40 mg kg^−1^.

Taken together, Corramycin showed promising in vivo efficacy in a mouse septicemia model despite poor pharmacokinetic properties and a moderate MIC. The decreased susceptibility towards Corramycin in an *E. coli* strain lacking the SbmA and YejEF transporter systems resulted in an increased ED_50_ in the infected mice, thus complementing the results that we had obtained in our in vitro experiments.

## Conclusion

In summary, Corramycin is a new antibiotic scaffold with unique or in natural products rarely observed structural features while displaying moderate to good anti‐Gram‐negative activity against numerous *E. coli* strains and *S. typhimurium*. Based on the unprecedented chemical structure of Corramycin and no observed cross‐resistance with antibiotic classes commonly used in the clinic, we assume a novel, not yet elucidated, MoA. The identification of the biosynthetic pathway in *C. coralloides* underpins that myxobacterial species are a promising source of new antibiotic natural products with completely different chemistry compared to previously identified antibiotics, e.g., from actinobacteria. Furthermore, we proposed a biosynthesis model including a possible detoxification mechanism involving fatty acid‐linked Corramycin precursors, which are exported and deacylated to secrete the active compound. Nevertheless, many proposed biosynthetic steps require further experimental investigations. Notably, the correct prediction of eight stereocenters based on the in silico analysis of the assembly line underlines the potential of bioinformatic tools in combination with chemical analysis methods in structure prediction of NRPS‐derived natural products.

We found a huge discrepancy between the FoR in rich medium compared to minimal medium due to the involvement of two transport systems in the Corramycin uptake. It is important to note that the typical development of an antibiotic would have been stopped with the high FoR found in the rich media. We here show that this process may be ineffective as the rich media typically employed in standardized FoR methods are certainly not appropriate to simulate infection processes. Minimal media might mimick in vivo conditions more realistically. Thus, antibiotics potentially useful for application in human or veterinary medicine may have been lost in the past. Corramycin serves as a prime example for such a scenario and our data clearly show the importance of more detailed studies using various media and of determining the potential mode(s) of resistance in the pathogen early in the drug discovery process.

In summary, the structural originality of Corramycin, the possibility to prepare analogues by total synthesis, the anti‐Gram‐negative activity, the lack of cross‐resistance with existing antibacterial classes, the mode of penetration and the promising efficacy in a mouse septicemia model, led to the conclusion that Corramycin is an attractive starting point for an antibacterial drug development program. Subsequent efforts aim at preparing new Corramycin analogues with more potent antimicrobial activities, a broadened spectrum against Gram‐negative pathogens and improved pharmaceutical properties, with the objective to identify a preclinical development candidate.

Sanofi holds a patent on the Corramycin molecule, its synthesis and antibiotic activity (WO 2019/053265A1).

## Conflict of interest

The authors declare no conflict of interest.

1

## Supporting information

As a service to our authors and readers, this journal provides supporting information supplied by the authors. Such materials are peer reviewed and may be re‐organized for online delivery, but are not copy‐edited or typeset. Technical support issues arising from supporting information (other than missing files) should be addressed to the authors.

Supporting Information

## Data Availability

The data that support the findings of this study are available from the corresponding author upon reasonable request.
