# Supplementary material for: Structure Elucidation, Total Synthesis, Antibacterial In Vivo Efficacy and Biosynthesis Proposal of Myxobacterial Corramycin
Source: Angew Chem Int Ed Engl. 2022 Nov 21;61(51):e202210747. doi: 10.1002/anie.202210747 (PMC10099666; doi:10.1002/anie.202210747)
Supplement: Supplementary file 1 — Supporting Information [file ANIE-61-0-s001.pdf]

Angewandte Chemie
Eine Zeitschrift der Gesellschaft Deutscher Chemiker

Supporting Information

Structure Elucidation, Total Synthesis, Antibacterial In Vivo Efficacy and Biosynthesis Proposal of Myxobacterial Corramycin

C. Couturier, S. Groß, A. von Tesmar, J. Hoffmann, S. Deckarm, A. Fievet, N. Dubarry, T. Taillier, C. Pöverlein, H. Stump, M. Kurz, L. Toti, S. Haag Richter, D. Schummer, P. Sizun, M. Hoffmann, R. Prasad Awal, N. Zaburannyi, K. Harmrolfs, J. Wink, E. Lessoud, T. Vermat, V. Cazals, S. Silve, A. Bauer, M. Mourez, L. Fraisse, C. Leroi-Geissler, A. Rey, S. Versluys, E. Bacqué, R. Müller, S. Renard**

Table of Contents

1. Isolation and structure elucidation of Corramycin 6

1.1 Isolation of Corramycin from *C. coralloides* ST201330 6

1.1.2 uHPLC-MS analysis 6

1.2 Structure Elucidation *via* MS^2 and NMR (two dimensional) 7

2. Configurational analyses - steps towards the absolute stereochemistry of Corramycin 19

2.1 Absolute configuration of the amino acid moieties *N*-MePhe, *β*-OH-Val, Ser and Leu 19

Hydrolysis and chiral GC/MS analysis 19

2.2 Prediction of the stereochemistry by *in silico* analysis of the BGC 19

2.3 Determination of the relative configuration of the two *vic*-dihydroxy units 19

2.4 Determination of the absolute configuration of methoxy-C13 via fragment synthesis 20

3. Total synthesis strategy to elucidate all remaining stereocenters 21

4. Supplementation of isotope-labelled precursors during fermentation 28

5. Determination of the minimal inhibitory concentration (MIC) *in vitro* 33

6. Investigation of the cross-resistance of Corramycin to clinically used antibiotics 34

7. Isolation of *C.* coralloides MCy10984 and ST201330 genomic DNA and illumina sequencing 35

8. *In silico* analysis of the Corramycin BGC 35

9. *In silico* analysis, protein purification and malachite green assay of the FAAL 41

10. Total synthesis of Corramycin 43

10.1 Retrosynthetic Analysis and Synthetic Strategy Overview 43

10.1.1 General procedure for preparation of compound (*S*)-2-((((9H-fluoren-9-yl)methoxy)carbonyl)amino)-3-hydroxy-3-methylbutanoic acid (10) 43

10.1.2 General procedure for preparation of compound (*S*)-2-((4S,5S)-5-((S)-2-((((9H-fluoren-9-yl)methoxy)carbonyl)amino)-1-methoxyethyl)-N,2,2-trimethyl-1,3-dioxolane-4-carboxamido)-3-phenylpropanoic acid (9) 44

10.1.3 General procedure for preparation of compound (2*R*,3*S*)-((((9H-fluoren-9-yl)methoxy)carbonyl)amino)-3-(1-methyl-H-imidazol-5-yl)-3-((triisopropylsilyl)oxy)propanoic acid (8) 45

10.1.4 Synthesis of compound (4R,5S)-2,2,5-trimethyl-1,3-dioxolane-4-carboxylic acid (7) ... 46

10.1.5 General procedure for the synthesis of Corramycin (Corramycin, 1) 46

10.2 Experimental Procedures 50

10.2.1 General procedure for the synthesis of Corramycin (1) 63

10.3 NMR data of all Building Blocks and synthetic Corramycin 65

10.3.1 NMR data of synthetic Corramycin 94

10.3.2 Comparison: ^{1}H NMR and 13C NMR of synthetic and natural Corramycin 101

11. Resistance development of *E. coli* towards Corramycin 103

11.1 Time-kill-curve (TKC) experiment 103

11.2 Determination of the frequency of resistance (FoR) 103

12. Isolation of genomic DNA from *E. coli*, Illumina sequencing and sequence analysis 104
13. λ red-mediated gene deletions of *sbmA* and *yejEF* 104
13.1 *sbmA* and *yejEF* single gene deletion.. 104
13.2 Complementation of SbmA and YejEF in the *E. coli* deletion mutant.................................... 106
14. Investigation of the Corramycin *in-vivo* activity in infected mice test animals and experimental ethics 106
14.1 Infection of the test animals and treatment with Corramycin *in vivo* 106
14.2 Corramycin elimination experiment.. 107
14.3 Determination of the effective dose for 50 % of the population (ED_{50}).................................... 107
References 108

Supplementary tables

Table S 1. NMR-data (700 MHz, D_2O+FA) for compound Corramycin (1). ... 7
Table S 2. Overview on the configurations for all 16 synthesized Corramycin-isomers. ... 23
Table S 3. Supplementation experiments of isotope-labelled Corramycin precursors and detection of Corramycin derivatives. ... 32
Table S 4. MIC values of Corramycin against all bacterial strains tested in this study. ... 33
Table S 5. Cross-resistance of Corramycin against tobramycin, Cefotaxim, Cefoxitin, Ciprofloxacin, Tetracycline and Rifampicin based on a MIC assay. ESBL: extended-spectrum β-lactamase; CP: carbapenem; AG: aminoglycoside; QL: quinolone; PRP ... 34
Table S 6. Proposed function of the biosynthetic genes involved in Corramycin biosynthesis. ... 37
Table S 7. NMR-data comparison (700 MHz, D_2O+FA) for natural and isolated Corramycin (1). ... 94
Table S 8. Frequency of resistance (FoR) of *E. coli* ATCC25922 against Corramycin at 4 × MIC in MHB or the minimal medium M9. ... 104
Table S 9. Oligonucleotides used for antibiotic resistance cassette amplification, *smbA* and *yejEF* gene deletions and verification of the deletions. ... 105

Supplementary figures

Figure S 1. Key 2D NMR correlations for Corramycin (1). ... 8
Figure S 2. High resolution mass spectra for all observed Corramycin derivatives (data recorded from a mixture of Corramycin derivatives, zoomed into the relevant *m/z* regions). Calculated exact mass and molecular formula for $[M+H]^+$ is given. A: Corramycins (2, *m/z* 1170), (1, *m/z* 1184), (3, *m/z* 1200) and (4, *m/z* 1216). B: Corramycin (5, *m/z* 1348). ... 10
Figure S 3. MS^2 Spectrum of Corramycin (1). Fragments were assigned according to the Biemann modication of the Roepstorff nomenclature. ... 11
Figure S 4. ^1H NMR of Corramycin (1), 700 MHz, D_2O+FA. ... 12
Figure S 5. ^{13}C NMR of Corramycin (1), 175 MHz, D_2O+FA. ... 13
Figure S 6. COSY NMR of Corramycin (1), 700 MHz, D_2O+FA. ... 14
Figure S 7. HSQC NMR of Corramycin (1), 175/700 MHz, D_2O+FA. ... 15
Figure S 8. HMBC NMR of Corramycin (1), 175/700 MHz, D_2O+FA. ... 16
Figure S 9. Sel-HMBC NMR of Corramycin (1), 175/700 MHz, D_2O+FA. ... 17
Figure S 10. MUSCLE alignment of the Corramycin FAAL with other FAALs. ... 18
Figure S 11. Determination of the absolute configuration of six Corramycin (1) stereocenters *via in silico* analysis and hydrolysis/chiral GC/MS (blue) and prediction of two stereocenters *via in silico* analysis (green). ... 19
Figure S 12. Structure of acetonide 6. ... 20
Figure S 13. Significant ROEs (red arrows) and relative stereochemistry of the two ketal moieties for acetonide 6. ... 20
Figure S 14. Synthesis of Corramycin fragments A and B for NMR comparison of the NMR data in order to elucidate the absolute configuration of C13. ... 21
Figure S 15. *N*-terminal part of Corramycin (1). All still unknown stereocenters after configuration analysis are highlighted and the remaining possible isomers are given. ... 23
Figure S 16. Absolute configuration of Corramycin (1). ... 24
Figure S 17. Example for GC/MS analysis: L-*N*-Me-Phe from hydrolysate of Corramycin (1) (R_t = 26.934 min, 98.5 % abundance). ... 25
Figure S 18. ^1H NMR spectrum of acetonide (6), 500 MHz, DMSO-d_6. ... 26
Figure S 19. ^{13}C NMR spectrum of acetonide (6), 125 MHz, DMSO-d_6. ... 26
Figure S 20. ROESY NMR spectrum of acetonide (6), 500 MHz, DMSO-d_6. ... 27
Figure S 21. Observed mass shifts after supplementation of isotope-labelled precursors during strain cultivation. ... 30
Figure S 22. d_8-L-valine incorporation into all Corramycin derivatives (1-5). ... 31
Figure S 23. Determination of the Corramycin BGC borders. ... 36

Figure S 24. Phylogenetic analysis of the Corramycin assembly line MT domains. The phylogenetic tree was generated by NapDos online server.[6] 39
Figure S 25. Phylogenetic tree of the Corramycin assembly line C and E domains. The phylogenetic tree was generated by NapDos online server.[6] 40
Figure S 26. Malachite green assay shows activation of numerous linear fatty acids with different lengths ranging from C6 to C13 by the FAAL domain. 41
Figure S 27. Retrosynthetic analysis of Corramycin (1). 43
Figure S 28. Synthetic strategy towards building block 10[16]. 44
Figure S 29. Synthetic strategy towards building block 9. 45
Figure S 30. Synthetic strategy towards building block 8. 46
Figure S 31. Synthesis of building block 7. 46
Figure S 32. Final steps *via* SPPS towards Corramycin (1). 49
Figure S 33. ^{1}H NMR of compound 18, 600 MHz, DMSO-d_6. 65
Figure S 34. ^{1}H NMR of compound 19, 600 MHz, DMSO-d_6. 66
Figure S 35. ^{1}H NMR of compound 10, 600 MHz, DMSO-d_6. 67
Figure S 36. ^{1}H NMR of compound 20, 600 MHz, $CDCl_3$. 68
Figure S 37. ^{13}C NMR of compound 20, 150 MHz, $CDCl_3$. 69
Figure S 38. ^{1}H NMR of compound 21, 600 MHz, $CDCl_3$. 70
Figure S 39. ^{13}C NMR of compound 21, 150 MHz, $CDCl_3$. 71
Figure S 40. ^{1}H NMR of compound 22, 600 MHz, $CDCl_3$. 72
Figure S 41. ^{13}C NMR of compound 22, 150 MHz, $CDCl_3$. 73
Figure S 42. ^{1}H NMR of compound 23, 600 MHz, $CDCl_3$. 74
Figure S 43. ^{13}C NMR of compound 23, 150 MHz, $CDCl_3$. 75
Figure S 44. ^{1}H NMR of compound 24, 600 MHz, $CDCl_3$. 76
Figure S 45. ^{13}C NMR of compound 24, 150 MHz, $CDCl_3$. 77
Figure S 46. ^{1}H NMR of compound 25, 600 MHz, $CDCl_3$. 78
Figure S 47. ^{13}C NMR of compound 25, 150 MHz, $CDCl_3$. 79
Figure S 48. ^{1}H NMR of compound 26, 600 MHz, $CDCl_3$. 80
Figure S 49. ^{13}C NMR of compound 26, 150 MHz, $CDCl_3$. 81
Figure S 50. ^{1}H NMR of compound 9, 600 MHz, DMSO-d_6. 82
Figure S 51. ^{13}C NMR of compound 9, 150 MHz, DMSO-d_6. 83
Figure S 52. ^{1}H NMR of compound 28, 600 MHz, $CDCl_3$. 84
Figure S 53. ^{13}C NMR of compound 28, 150 MHz, $CDCl_3$. 85
Figure S 54. ^{1}H NMR of compound 29, 600 MHz, $CDCl_3$. 86
Figure S 55. ^{13}C NMR of compound 29, 150 MHz, $CDCl_3$. 87
Figure S 56. ^{1}H NMR of compound 30, 600 MHz, $CDCl_3$. 88
Figure S 57. ^{13}C NMR of compound 30, 150 MHz, $CDCl_3$. 89
Figure S 58. ^{1}H NMR of compound 32, 600 MHz, $CDCl_3$. 90
Figure S 59. ^{13}C NMR of compound 32, 150 MHz, $CDCl_3$. 91
Figure S 60. ^{1}H NMR of compound 8, 600 MHz, $CDCl_3$. 92
Figure S 61. ^{13}C NMR of compound 8, 150 MHz, $CDCl_3$. 93
Figure S 62. Key 2D NMR correlations for Corramycin (1). 95
Figure S 63. ^{1}H NMR of synthetic Corramycin (1), 700 MHz, D_2O + FA. 96
Figure S 64. ^{13}C NMR of synthetic Corramycin (1), 700 MHz, D_2O + FA. 97
Figure S 65. HSQC NMR of synthetic Corramycin (1), 150/700 MHz, D_2O + FA. 98
Figure S 66. COSY NMR of synthetic Corramycin (1), 700 MHz, D_2O + FA. 99
Figure S 67. HMBC NMR of synthetic Corramycin (1), 150/700 MHz, D_2O + FA. 100
Figure S 68. ^{1}H NMR of synthetic (A, in red) and natural (B, in blue) Corramycin (1), 700 MHz, D2O + FA. 101
Figure S 69 ^{13}C NMR of synthetic (A, in red) and natural (B, in blue) Corramycin (1), 700 MHz, D2O + FA. 102
Figure S 70. Time-kill-curves (TKCs) for Corramycin at 4x MIC and 8x MIC using *E. coli* ATCC25922 grown in either CAMHB (a) or M9 medium (b). 103
Figure S 71. A) Blood bacteremia experiment. 107

1. Isolation and structure elucidation of Corramycin

1.1 Isolation of Corramycin from *C. coralloides* ST201330

For the isolation of Corramycin (**1**), a seed culture of the natural producer strain *C. coralloides* ST201330 was inoculated from a culture grown on solid yeast agar (1 % (w/v) fresh baker's yeast, 0.1 % (w/v) $CaCl_2$ x $2H_2O$, 0.477 % 4-(2-hydroxyethyl)-1-piperazineethanesulfonic acid (HEPES), 0.00005 % (w/v) vitamin B_{12}, 1.8 % agar, final pH 7.2) by scraping off all cells from the agar plate and transferring them into 100 mL liquid yeast medium (same composition as described above; without agar). Cultivation of the seed culture was performed in 300 mL shaking flasks for 7 days at 30 °C and 180 rpm on an orbital shaker (Multitron, Infors HT). Next, 50 mL of the seed culture was used to inoculate 500 mL production medium containing 0.5 % (w/v) oat flakes, 0.5 % (w/v) glucose, 0.5 % (w/v) glycerol, 0.5 % (w/v) $CaCl_2 \times 2H_2O$, 0.00005 % (w/v) vitamin B_{12} (added after autoclaving from sterile filtered stock) and 2 % (w/v) XAD16 absorber resin (final pH 8.5). The cultivation was performed in 2 L unbaffled shaking flasks at 30 °C for 7 to 9 days.

After centrifugation of the fermentation broth (45 L total volume), the supernatant was discarded and the cells and absorber resin were harvested and subsequently freeze dried. Next, five extraction steps were performed, three times with 4 L MeOH/H_2O (1:1) and two times with 4 L MeOH/H_2O (3:1), followed by filtering the extracts and loading them onto a column filled with 5.8 L of CHP-20P material (MCI gel, 75- 150 pm, Mitsubishi Chemical Corporation). Elution was carried out using a 2-propanol gradient from 5 % to 40 % over 60 minutes (min) and the eluate was collected in fractions, which were subsequently analyzed using uHPLC-MS (see section below). The fractions containing the unknown active metabolite with an *m/z* $[M+H]^+$ value of 1184.56 were combined, the solvent was evaporated on a rotary evaporator and finally lyophilized, yielding ~15 g dried extract. The dried extract was dissolved in 300 mL MeOH and purified in a preparative HPLC step using a Phenomenex Luna C-18 (2) column (10 pm; 250 mm × 50 mm). The separation was performed over a period of 40 min using a gradient from 25 % to 50 % MeOH (in H_2O plus 0.1 % formic acid (FA)) at a flow rate of 140 mL min^{-1}. The eluent was collected in fractions, of which those containing Corramycin were combined, the solvent was evaporated using a rotary evaporator and further dried using lyophilization. The dried extract was dissolved in 30 mL of MeOH/H_2O (1:1) and purified in a second preparative HPLC step using a Phenomenex phenyl-hexyl C-18 column (100 mm × 50 mm). Separation was carried out over a period of 40 min using a gradient from 25 % to 60 % MeOH (in H_2O) and 50 g L^{-1} ammonium acetate (pH 6.8) at a flow rate of 140 mL min^{-1}. Again, the column flow through was collected in fractions and the target compound containing fractions were combined and lyophilized after solvent evaporation, yielding ~230 mg of Corramycin.

<u>1.1.2 uHPLC-MS analysis</u>

For uHPLC-MS analysis we used an UltiMate 3000 LC System (Dionex) with an acquity UPLC BEH C-18 column (1.7 µm, 100 x 2 mm; Waters), equipped with a VanGuard BEH C-18 (1.7 µm; Waters) guard column coupled to an Apollo II ESI source (Bruker) and hyphenated to maXis 4G ToF mass spectrometer (Bruker). Separation was performed at a flow rate of 0.6 mL min^{-1} (eluent A: deionized water + 0.1 % FA, eluent B: acetonitrile + 0.1 % FA) at 45 °C using the following gradient: 5 % B for 30 s, followed by a linear gradient up to 95 % B in 18 min and a constant percentage of 95 % B for further 2 min. Original conditions were adjusted with 5 % B within 30 s and kept constant for 1.5 min. The LC flow was split to 75 µL min^{-1} before entering the mass spectrometer. Mass spectra were acquired in centroid mode in the range from 150 to 2,500 *m/z* at a 2 Hz full scan rate. Mass spectrometry source parameters were set to 500 V as end plate offset, 4000 V as capillary voltage, 1 bar nebulizer gas pressure, 5 L min^{-1} dry gas flow and 200 °C dry temperature. For MS^2 experiments, CID (collision-induced dissociation) energy varied linearly from 30, 35, 45, to 55 eV with respect to the precursor *m/z* from 300, 600, 1000, to 2000 *m/z*. MS full scan acquisition rate was set to 2 Hz and MS/MS spectra acquisition rates were ramped from 1 to 3 Hz for precursor ion intensities of 10 kcts to 1000 kcts. We used *Compass DataAnalysis* version 4.4 (Bruker) to interpret MS data.

1.2 Structure Elucidation *via* MS^2 and NMR (two dimensional)

Table S 1. NMR-data (700 MHz, D_2O+FA) for compound Corramycin (**1**).

position	δ_H, mult (*J* in Hz)	δ_C	CH_x	COSY	HMBC
**1**	1.14, br d (6.4)	18.1	CH_3	2,3	2, 3, 4[w]
**2**	3.97, m	67.9	CH	1,3	1[w], 4[w]
**3**	3.96, m	74.7	CH	1,2	1, 2, 4
**4**	-	174.2	C	-	-
**5**	5.02, d (*9.6*)	55.3	CH	6	4, 6, 7, 11
**6**	5.21, d (*9.5*)	63.9	CH	5	5, 7, 8, 11
**7**	-	132.2	C	-	-
**8**	7.47, br s	118.3	CH	none	6, 7, 9, 10[w]
**9**	[a]	136.2	CH	[a]	[a]
**10**	3.98, s	33.9	CH_3	none	7, 9
**11**	-	170.4	C	-	-
**12**	3.58, m[b]	38.3	CH_2	13, 15[b]	11, 13, 14
**13**	3.56, m[b]	78.6	CH	12, 15[b]	12, 14
**14**	3.32, s	57.0	CH_3	none	13
**15**	4.03, m	70.2	CH	16	12, 13
**16**	4.56, m	68.4	CH	15	13, 15, 17
**17**	-	173.6	C	-	-
**18**	3.03, s	32.2	CH_3	none	17, 19
**19**	5.36, dd (*10.8; 5.1*)	59.7	CH	20a, 20b	17, 18, 20a, 20b, 21, 25
**20a**	3.08, dd (*14.4; 11.2*)	32.8	CH_2	19, 20b	19, 21, 22/23a/b, 25
**20b**	3.44, dd (*14.4; 5.5*)			19, 20a	19, 21, 22/23a/b,
**21**	-	137.0	C	-	-
**22a, 22b**	7.38, m	128.6	CH	23a/b, 24[b]	21, 23a/b
**23a, 23b**	7.33, m	129.0	CH	22a/b, 24[b]	20, 22a/b, 24
**24**	7.31, m	126.8	CH	22a/b, 23a/b[b]	22a/b, 23a/b
**25**	-	172.3	C	-	-
**26a**	3.99, m	42.5	CH_2	26b	25, 27
**26b**	4.16, d (*17.0*)			26a	25, 27
**27**	-	170.7	C	-	-
**28**	4.55, m	60.6	CH	none	27, 29, 30/31, 32
**29**	-	71.9	C	-	-
**30, 31**	1.27-1.32, m[b]	25.0, 25.2/ 25.9, 26.0[c]	CH_3	none	28, 29
**32**	-	171.3	C	-	-

**33**	4.60, m	55.3	CH	34	32, 34, 35
**34**	3.89, m	61.1[d]	CH_2	33	33, 35
**35**	-	171.3	C	-	-
**36**	4.42, s	60.7	CH	none	35, 37, 38/39, 40
**37**	-	71.7	C	-	-
**38, 39**	1.27-1.32, m[b]	25.0, 25.2/ 25.9, 26.0[c]	CH_3	none	36, 37
**40**	-	171.0	C	-	-
**41**	4.48, m	52.3	CH	42,43[e]	40, 42, 43, 45
**42**	1.67, m[e]	39.6	CH_2	41, 43, 44a/b[e]	41, 43, 44a/b, 45[w]
**43**	1.67, m[e]	24.2	CH	41, 42, 44a/b[e]	41, 42, 44a/b
**44a**	0.89, d (*5.3*)	20.6	CH_3	42, 43, 44b[e]	42, 43, 44b
**44b**	0.94, d (*5.3*)	22.2	CH_3	42, 43, 44b[e]	42, 43, 44a
**45**	-	174.2	C	-	-
**46**	4.45, m	55.4	CH	47a, 47b	45, 47a/b, 48
**47a**	3.89, m	61.1[d]	CH_2	46, 47b	46, 48
**47b**	3.93, dd (*11.7, 5.4*)			46, 47a	46, 48
**48**	-	173.7	C	-	-

[a]no proton signal detectable (H9 only detectable without addition of FA. δ_H = 7.90 ppm, δ_C = 139.1 ppm, D_2O, 700 MHz); [b]overlapping signals, assignment of COSY correlations ambiguous; [c]assignment of C30/C31 and C38/C39 ambiguous as the proton signals highly overlap; [d]assignment of C34 and C47 ambiguous as the signals highly overlap; [e]overlapping signals of H42/H43, assignment of COSY correlations ambiguous; [w]weak HMBC signal

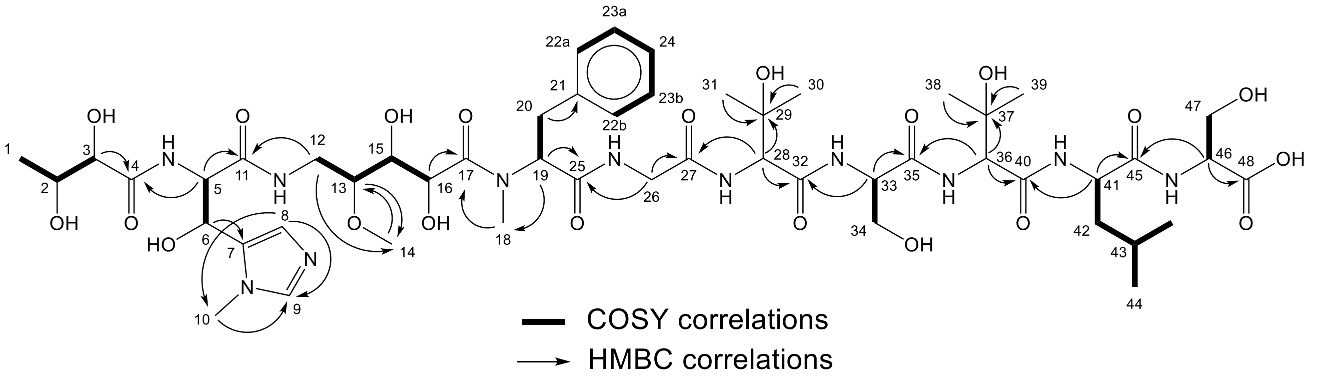

Figure S 1. Key 2D NMR correlations for Corramycin (1).

A first look at the NMR spectra clearly shows a generally peptidic structure. Further analysis reveals the amino acid building blocks *N*-Me-Phe, Gly, OH-Val, Ser, OH-Val, Leu and Ser (order towards C-terminus). All amino acids show their characteristic chemical shifts and the expected cross peaks in the COSY (Figure S 6) and HMBC (Figure S 8) spectra. The order is manifested by analyzing the HMBC correlations from the α-protons to the respective amide carbonyl groups in direct neighborhood. For better resolution, a selective HMBC was recorded (Figure S 9).

The *N*-terminal part of the molecule is linked to a 2,3-dihydroxylated butyric acid unit. The hydroxylation is indicated by the proton chemical shifts of around 4 ppm and the carbon shifts of 67.9 and 74.7 ppm, respectively. COSY correlations between H1, H2 and H3 as well as HMBC cross peaks to quaternary C4 (174.2 ppm) confirm the butyric acid structure. The HMBC cross peak from the α-proton of the next amino acid to C4 indicates the linkage of the butyric acid unit to this amino acid. This α-proton shows COSY correlation to H6, a CH group with a chemical shift of 5.21 ppm (^{1}H) and 63.9 (^{13}C), indicating a hydroxylation. C6 shows HMBC correlations to an *N*-methylated imidazole, the structure of which is confirmed by HMBC correlations as indicated in Table S 1. Finally, this hydroxylated *N*-Me-histidine is linked to a C-5 (pentanoic acid) unit that is in turn coupled *via* an amide bond to the *N*-terminal *N*-Me-Phe from the peptide chain. The pentanoic acid unit turns out to be substituted by two hydroxy groups (at C15 and C16) and one methoxy group at C13. COSY and HMBC cross peaks for substituted the pentanoic acid unit all fit to the expected correlations (Table S 1).

^{1}H NMR, ^{13}C NMR and 2D spectra were recorded using a Bruker Avance III Spectrometer Ascend-700 [700 MHz (1H) and 175 MHz (13C)] spectrometer in D_2O (with or without FA). The spectrometer is equipped with a He-cooled 5 mm TCI-CryoProbe (CP TCI 700S3 H-^{13}C/^{15}N-D-05 Z). Chemical shifts are reported in ppm relative to TMS and the solvent was used as the internal standard.

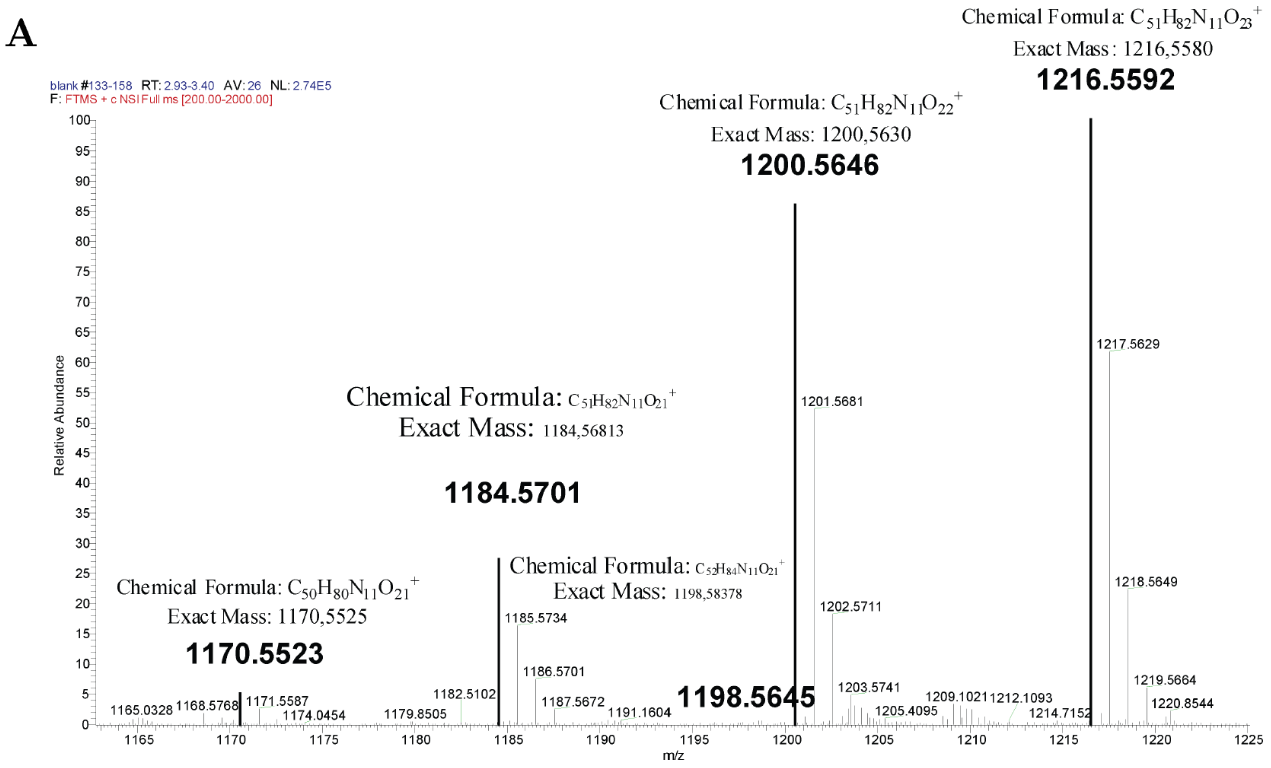

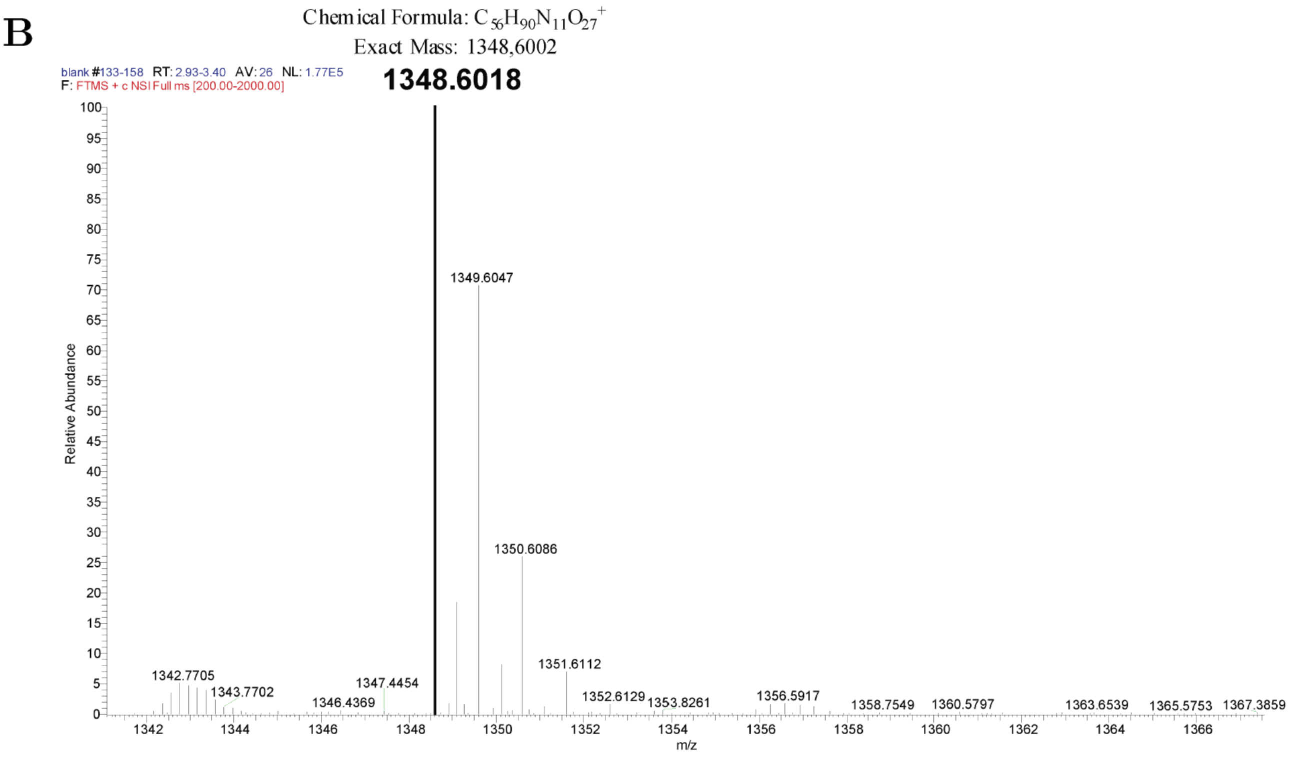

Figure S 2. High resolution mass spectra for all observed Corramycin derivatives (data recorded from a mixture of Corramycin derivatives, zoomed into the relevant *m/z* regions). Calculated exact mass and molecular formula for $[M+H]^+$ is given. A: Corramycins (**2**, *m/z* 1170), (**1**, *m/z* 1184), (**3**, *m/z* 1200) and (**4**, *m/z* 1216). B: Corramycin (**5**, *m/z* 1348).

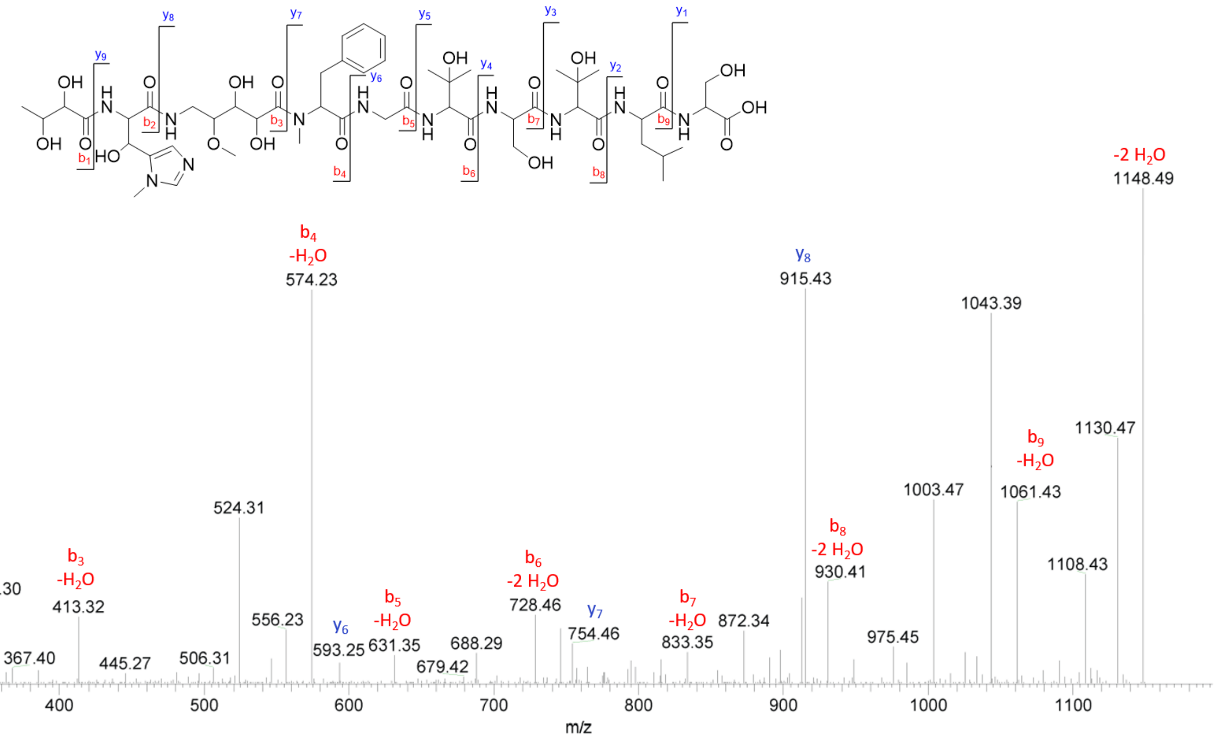

Figure S 3. MS[2] Spectrum of Corramycin (**1**). Fragments were assigned according to the Biemann modication of the Roepstorff nomenclature. Experimental details can be found in section 1.1.2.

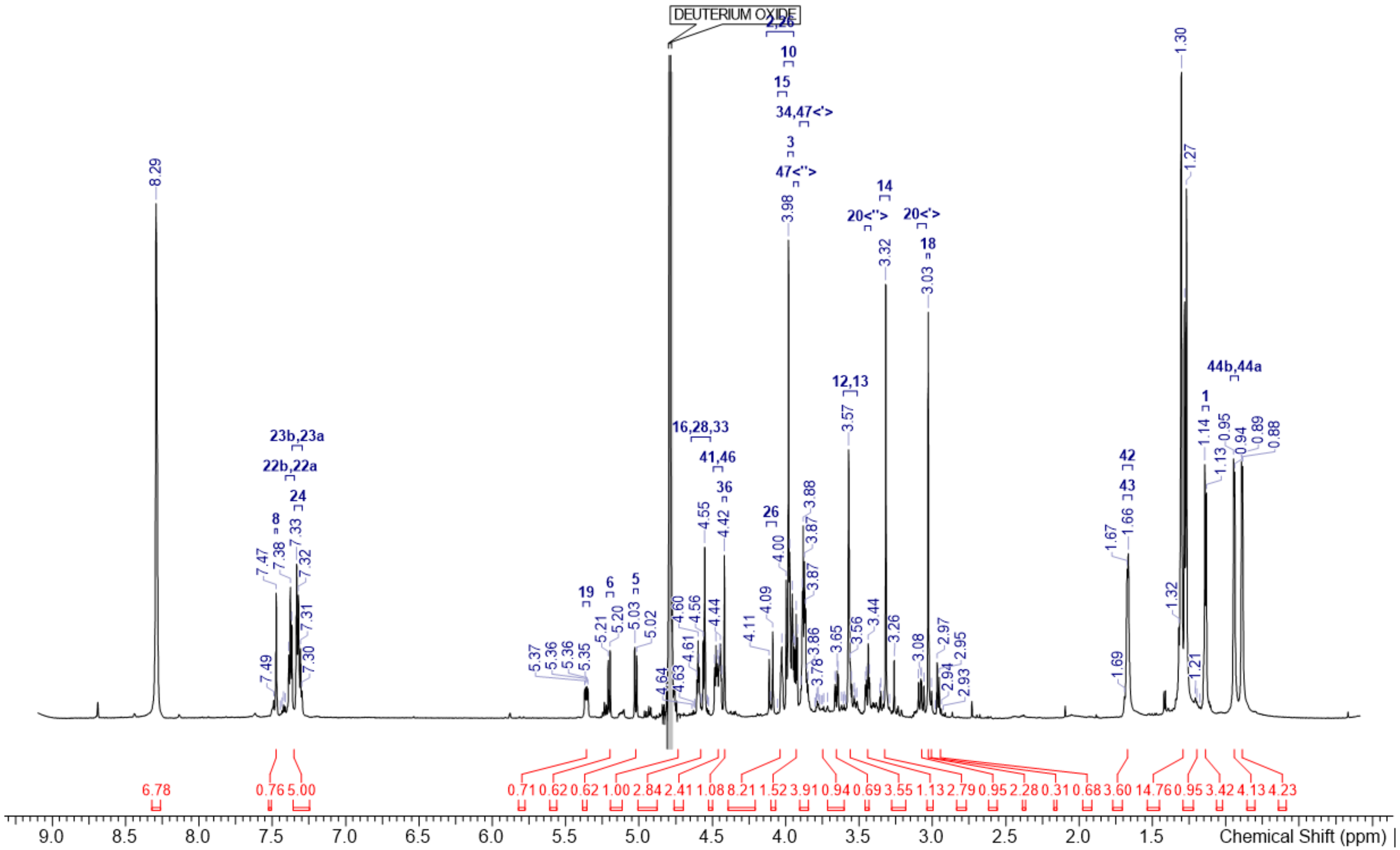

Figure S 4. ^{1}H NMR of Corramycin (1), 700 MHz, D_2O+FA.

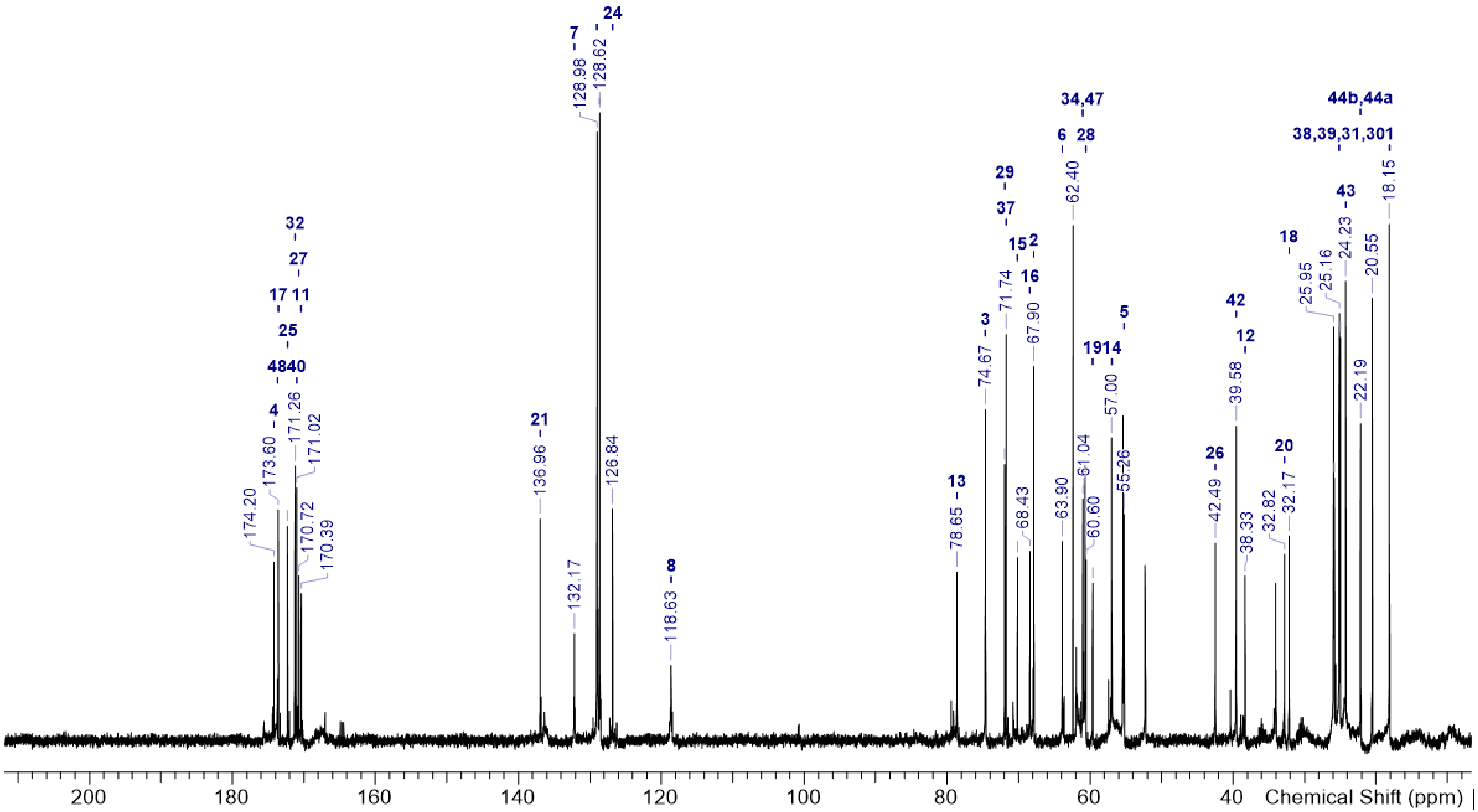

Figure S 5. ^{13}C NMR of Corramycin (1), 175 MHz, D_2O+FA.

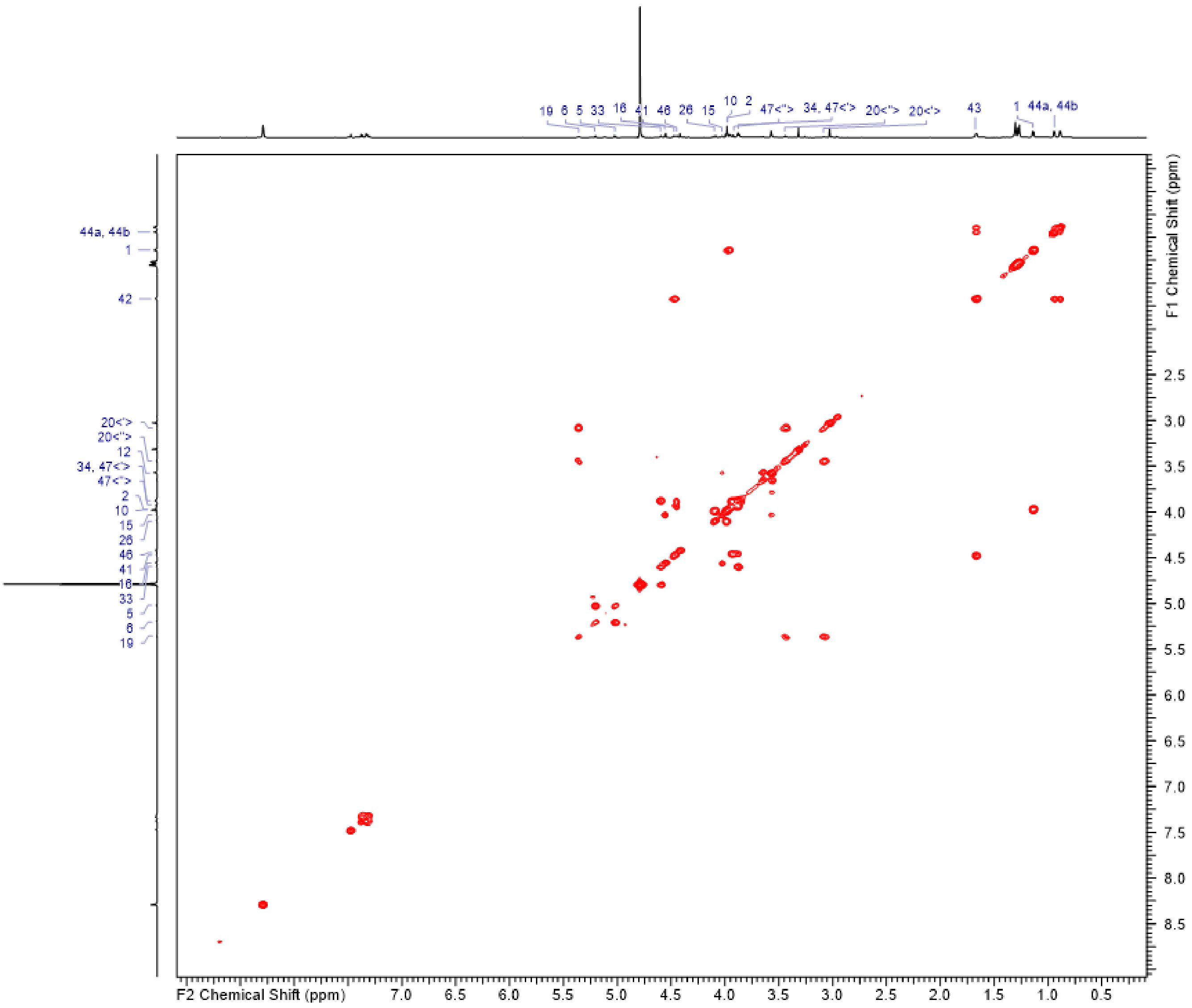

Figure S 6. COSY NMR of Corramycin (1), 700 MHz, D_2O+FA.

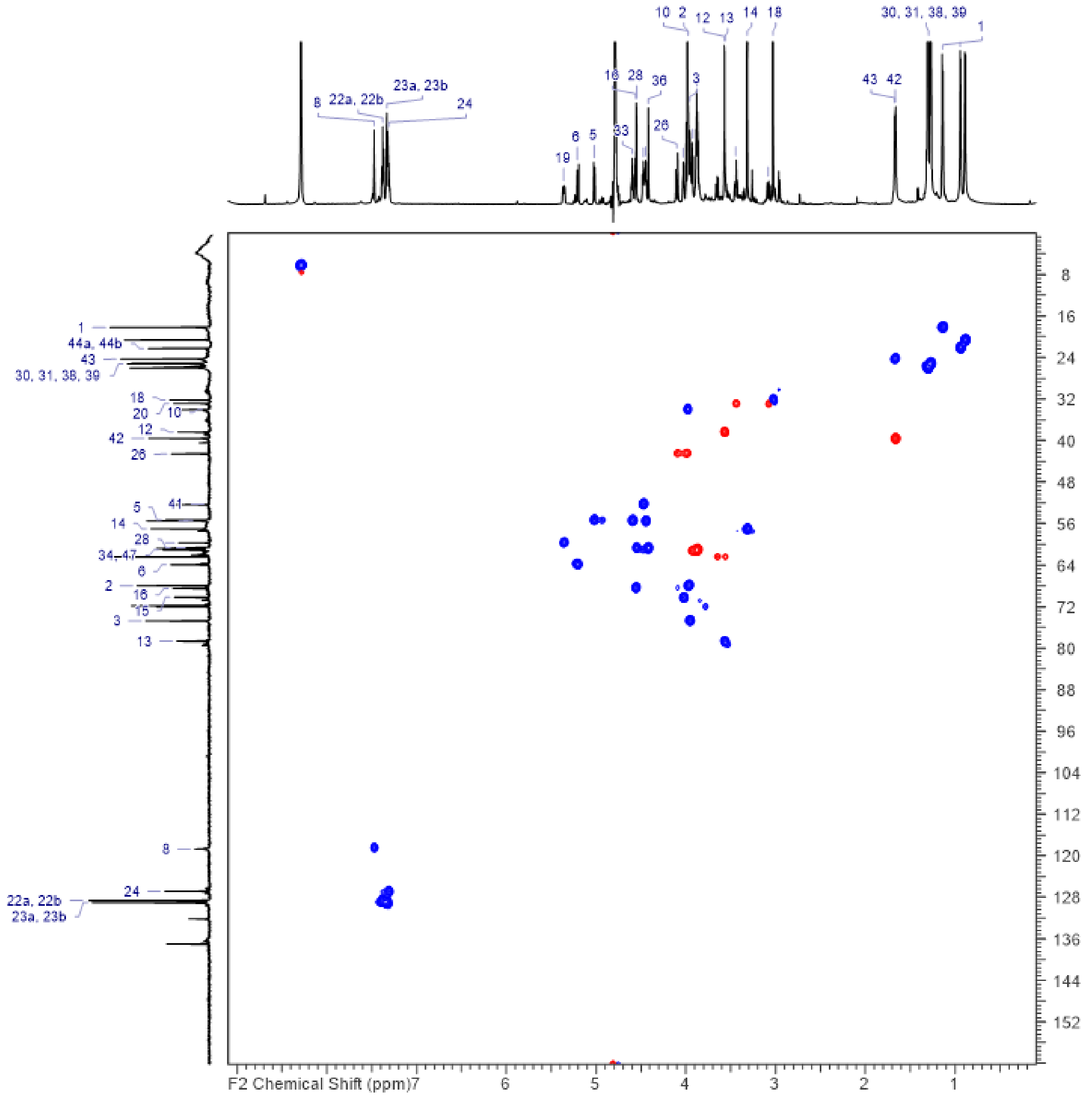

Figure S 7. HSQC NMR of Corramycin (1), 175/700 MHz, D_2O+FA.

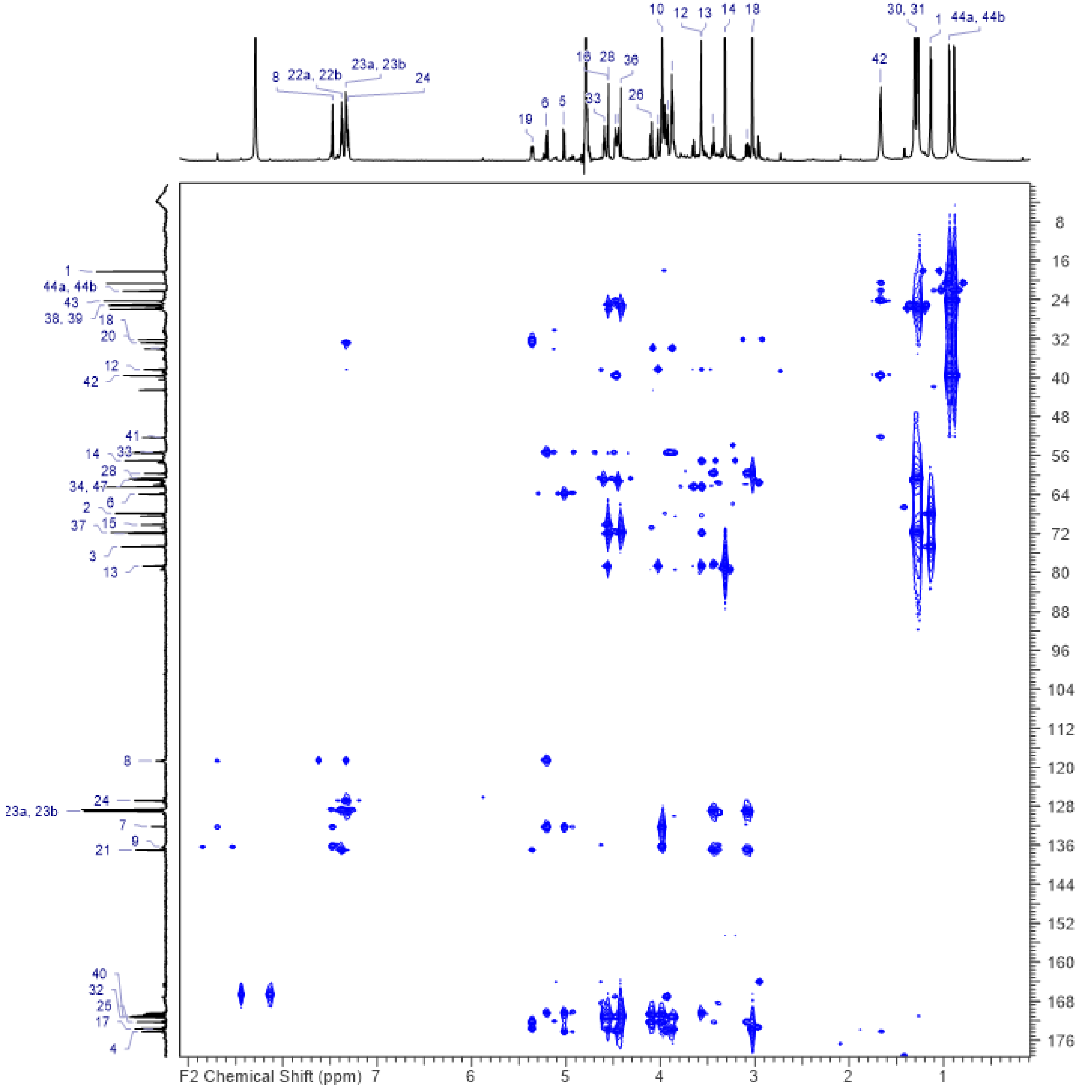

Figure S 8. HMBC NMR of Corramycin (1), 175/700 MHz, D_2O+FA.

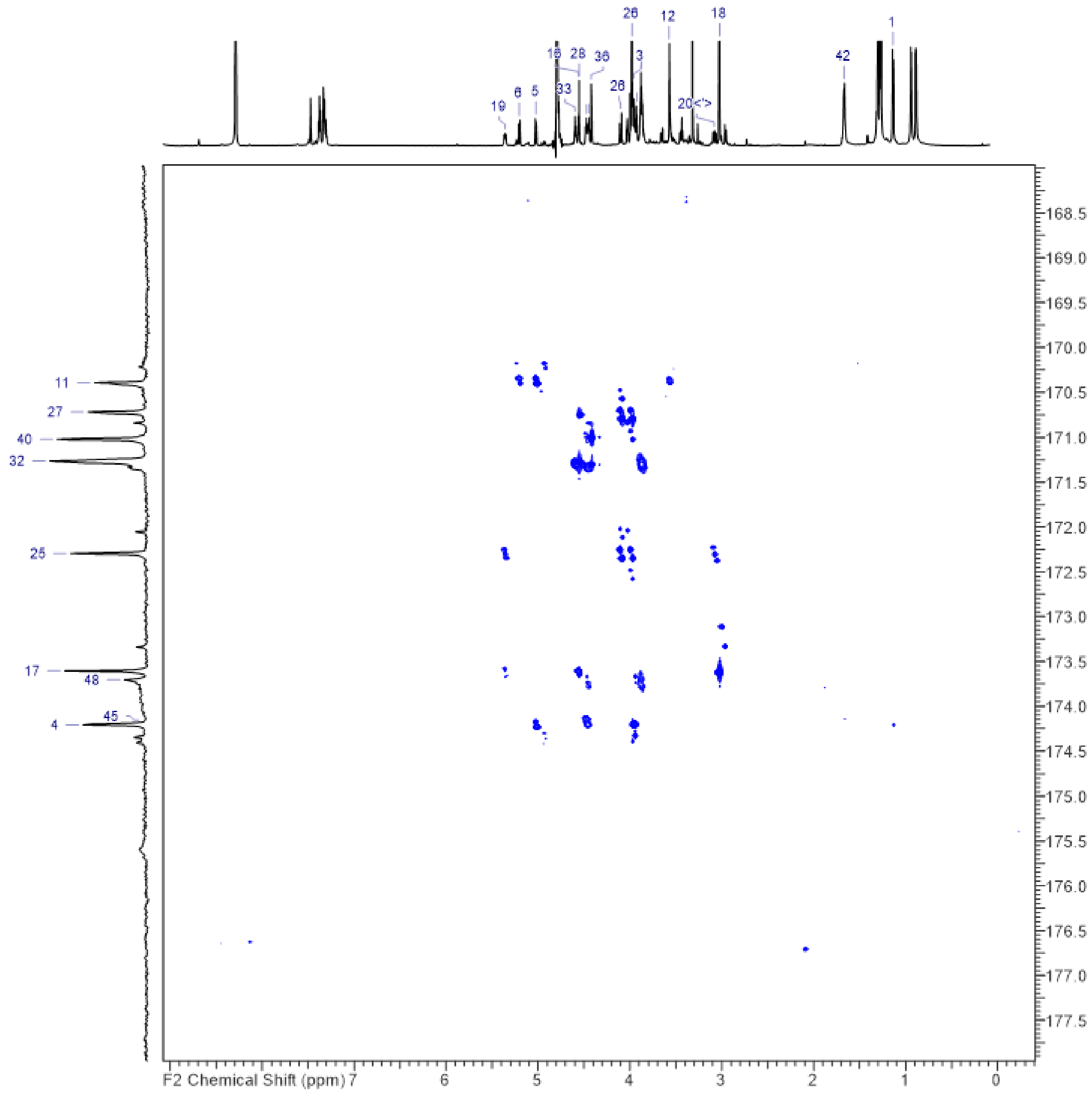

Figure S 9. Sel-HMBC NMR of Corramycin (1), 175/700 MHz, D_2O+FA.

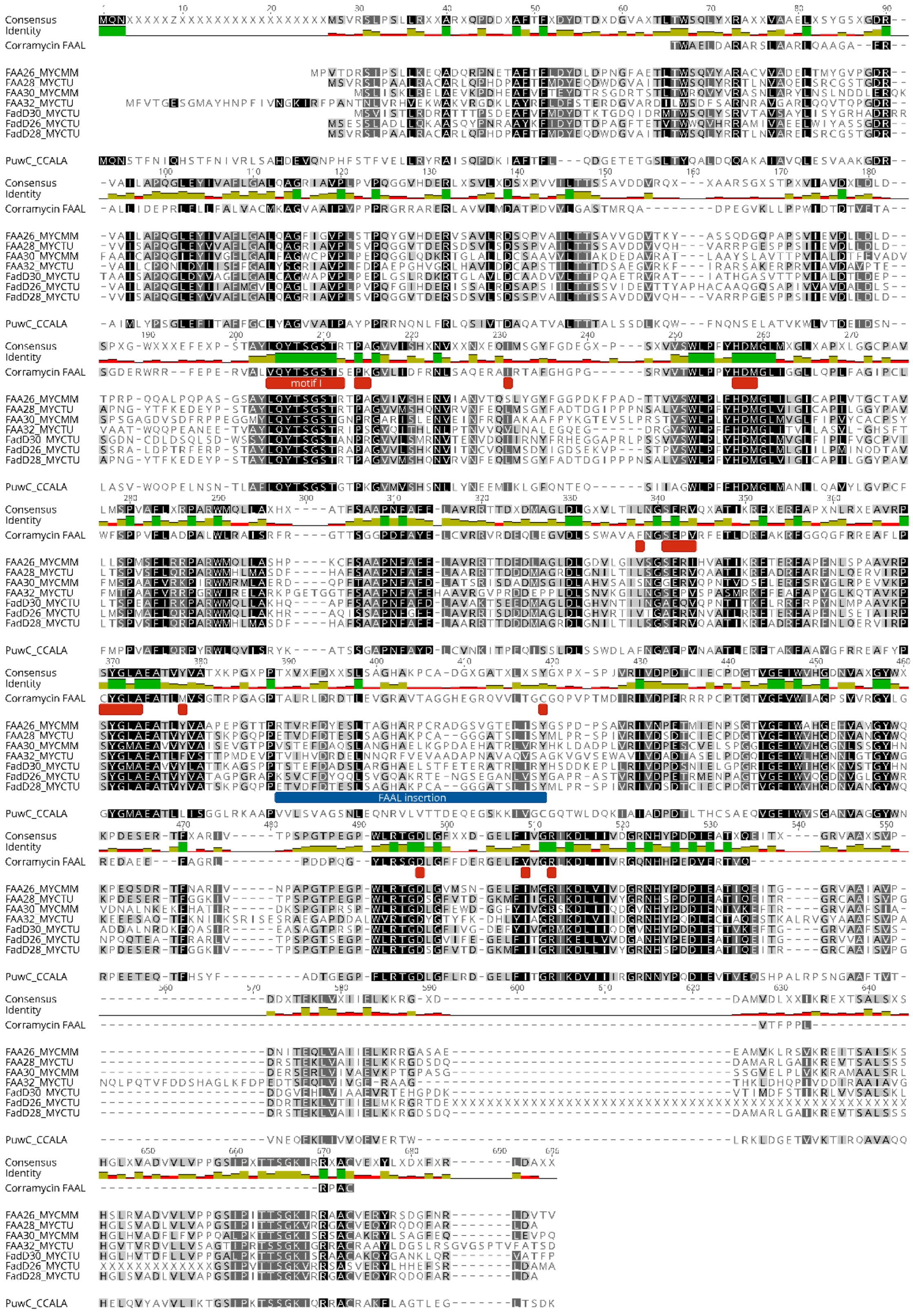

Figure S 10. MUSCLE alignment of the Corramycin FAAL with other FAALs. Active side residues (red bars) were identified using BLAST. The sequence insertion that is characteristic for FAALs and missing in fatty acid CoA-ligases (FACLs) is shown in blue.

2. Configurational analyses - steps towards the absolute stereochemistry of Corramycin

2.1 Absolute configuration of the amino acid moieties *N*-MePhe, *β*-OH-Val, Ser and Leu

<u>**Hydrolysis and chiral GC/MS analysis**</u>

Absolute configurations of the amino acid moieties *N*-MePhe, *β*-hydroxy valine, serine and leucine were determined after hydrolysis of Corramycin (**1**) followed by enantiomer labelling (ELAB, chiral GC)[1], shown in Figure S 11, blue labels. GC/MS analyses were performed at Chromatographie and Analysentechnik (C.A.T.) GmbH & Co KG (Tübingen, German) as a contract research organization. For the determination of *N*-MePhe, serine and leucine, commercially available reference samples (*D* and *L* amino acids) could be used. For the determination of β-hydroxy valine, the *D* enantiomer was synthesized as a reference sample from *S*-Fmoc serine methyl ester by a literature-known procedure (see section 10.2 Experimental Procedures, (Step 1.1 to Step 1.4). Figure S 17 shows the GC/MS analysis for *β*-hydroxy-valine, exemplary.

2.2 Prediction of the stereochemistry by *in silico* analysis of the BGC

Additionally to the hydrolysis, we also performed an *in silico* analysis of the biosynthetic gene cluster (BGC). A deep look into the module and domain architecture of the Corramycin assembly line gave us strong hints towards the absolute stereochemistry of eight stereocenters. The configuration of *N*-MePhe, *β*-hydroxy valine, serine and leucine was confirmed and two additional stereocenters could be predicted (Figure S 11 labelled in green, data see chapter 8).

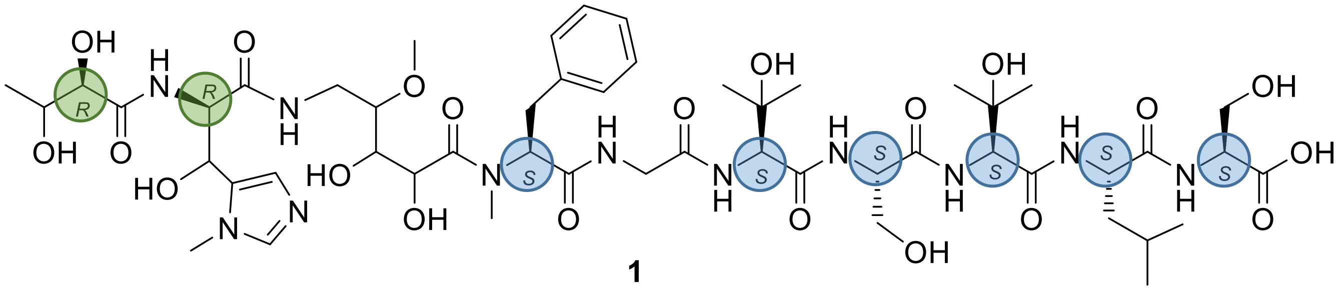

1

Figure S 11. Determination of the absolute configuration of six Corramycin (1) stereocenters *via in silico* analysis and hydrolysis/chiral GC/MS (blue) and prediction of two stereocenters *via in silico* analysis (green).

2.3 Determination of the relative configuration of the two *vic*-dihydroxy units

Relative configurations of the hydroxylated *γ*-amino valeric acid (5- Amino- 2,3- dihydroxy- 4- methoxy- pentanoic acid) and the dihydroxy butyric acid units were determined by NMR of the acetonide derivatives of Corramycin (**1**). A mixture of acetonide **6** and the monosubstituted derivative (see below) was obtained after treatment of Corramycin with 2,2- dimethoxypropane in the presence of catalytic amounts of CSA following a standard procedure[2]

The analysis of the NMR spectra was hampered by the presence of two compounds in a ration of ca. 2.5:1.0. Analysis of signals belonging to the main component led to the fully derivatized molecule, carrying two ketal protecting groups in the expected positions (Figure S 12, compound **6**). For the minor component, only one ketal group in position C15/C16 is observed. Nevertheless, the analysis of the ROESY spectrum led to an unambiguous assignment of the relative stereochemistry of C2/C3 and C15/C16, respectively.

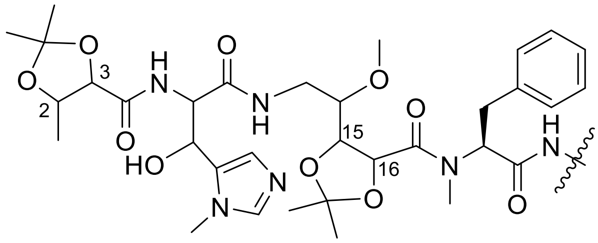

Figure S 12. Structure of acetonide 6.

Figure S 13 reveals significant ROEs observed for the two ketal moieties. In case of the *N*-terminal moiety, H2 and H3 experience intensive ROEs to different methyl groups of the ketal protecting group (signals at 1.31 ppm and 1.15 ppm, respectively). Therefore, the two protons must be oriented on opposite sites of the 5-membered ring (trans), leading to either *R,S* or *S,R* configuration.

In the second ketal moiety, H15 and H16 both experience an intensive ROE to the same methyl group at 1.25 ppm indicating a cis configuration of the two protons. Therefore, the absolute configuration is either *S,S* or *R,R*.

For ^{1}H, ^{13}C and ROESY NMR spectra of the acetonides, see Figure S 18 to Figure S 20.

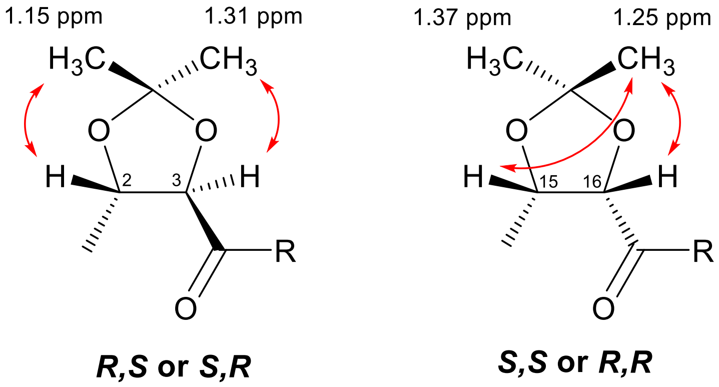

Figure S 13. Significant ROEs (red arrows) and relative stereochemistry of the two ketal moieties for acetonide 6.

2.4 Determination of the absolute configuration of methoxy-C13 via fragment synthesis

While the relative configurations of both dihydroxy units at C2/C3 and C15/C16 could be clearly elucidated from the 2D spectra of the acetonides, the configuration of the methoxy center in relation to the dihydroxy unit could not be unambiguously determined by NMR. However, after careful inspection of the NMR spectra of Corramycin and its corresponding acetonides we suspected the configuration of the 5-Amino-2,3-dihydroxy-4-methoxy-pentanoic acid unit to be more likely 2*S*,3*R*,4*S* (or 2*R*,3*S*,4*R*, respectively) than 2*S*,3*R*,4*R* (or 2*R*,3*S*,4*S*). In order to prove this hypothesis, model substances were synthesized from both *D*-ribono and *D*-lyxono lactone acetonides leading to the 2*R*,3*S*,4*R* and 2*S*,3*R*,4*R* configured capped fragments **A** and **B**, respectively (

Figure S **14**). Indeed, NMR signals of fragment **A** derived from *D*-ribono lactone displayed a good match with those of Corramycin while significant differences where observed for the resonances of fragment **B**. Therefore, the absolute configuration of C13/C15/C16 is predicted to be *S*/*R*/*S* or *R*/*S*/*R*. The detailed synthesis of the fragments is not shown as it basically followed the same protocols as described for Corramycin (**1**) in section 10.2 Experimental Procedures.

Remark: the nomenclature of the configuration of C15 changes depending on the derivatization. With a free hydroxy group, the configuration is (*R*), in the acetonide-derivative, the configuration is (*S*).

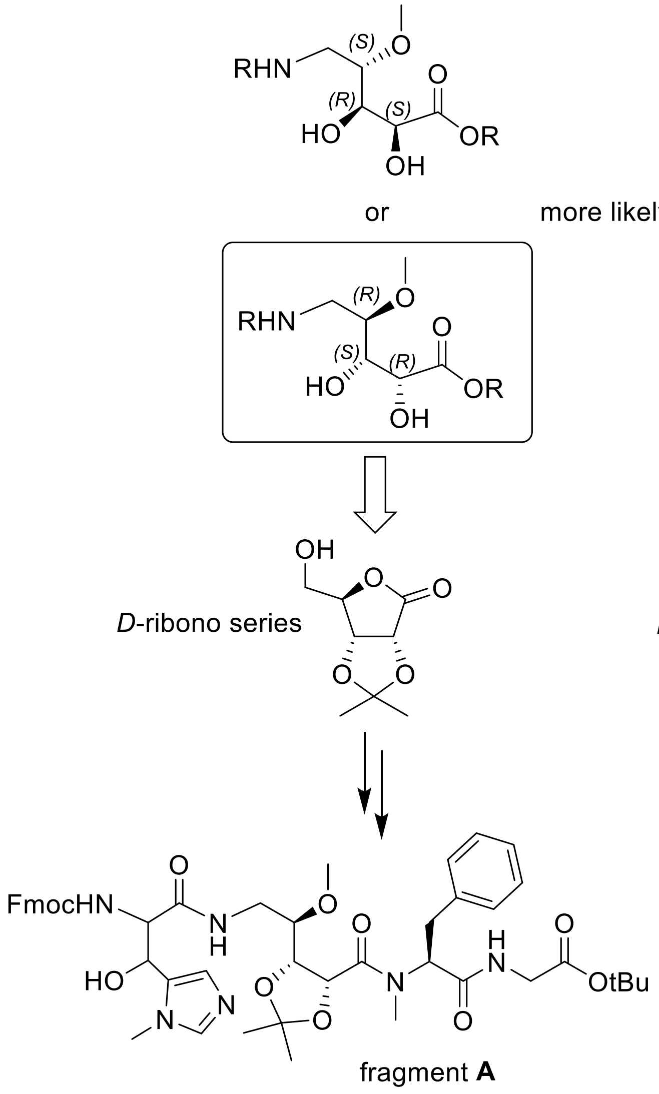

Figure S 14. Synthesis of Corramycin fragments A and B for NMR comparison of the NMR data in order to elucidate the absolute configuration of C13.

3. Total synthesis strategy to elucidate all remaining stereocenters

After extensive configuration analysis of the *N*-terminal part of Corramycin (**1**), 16 possible isomers are remaining as shown in Figure S 15. To fully elucidate the structure of Corramycin (**1**), all these 16 isomers were synthesized. As shown in Table S 2, 8 isomers were synthesized starting from *D*-ribose (C13/C15/C16 = *R*/*S*/*R)*, 8 isomers starting from *L*-ribose (C13/C15/C16 = *S*/*R*/*S).* The isomers synthesized from *D*-ribose were all inactive and the structure did not match with Corramycin (**1**). One compound (isomer No 10, Table S 2) from the *L*-ribose series was identical to Corramycin (**1**) (NMR data and biological activity matched). So, the absolute configuration of Corramycin (**1**) was finally elucidated as summarized in Figure S 16.

For NMR spectra of the matching *L*-ribose series isomer and a ^{1}H NMR spectrum overlay of synthetic and natural Corramycin (**1**), see

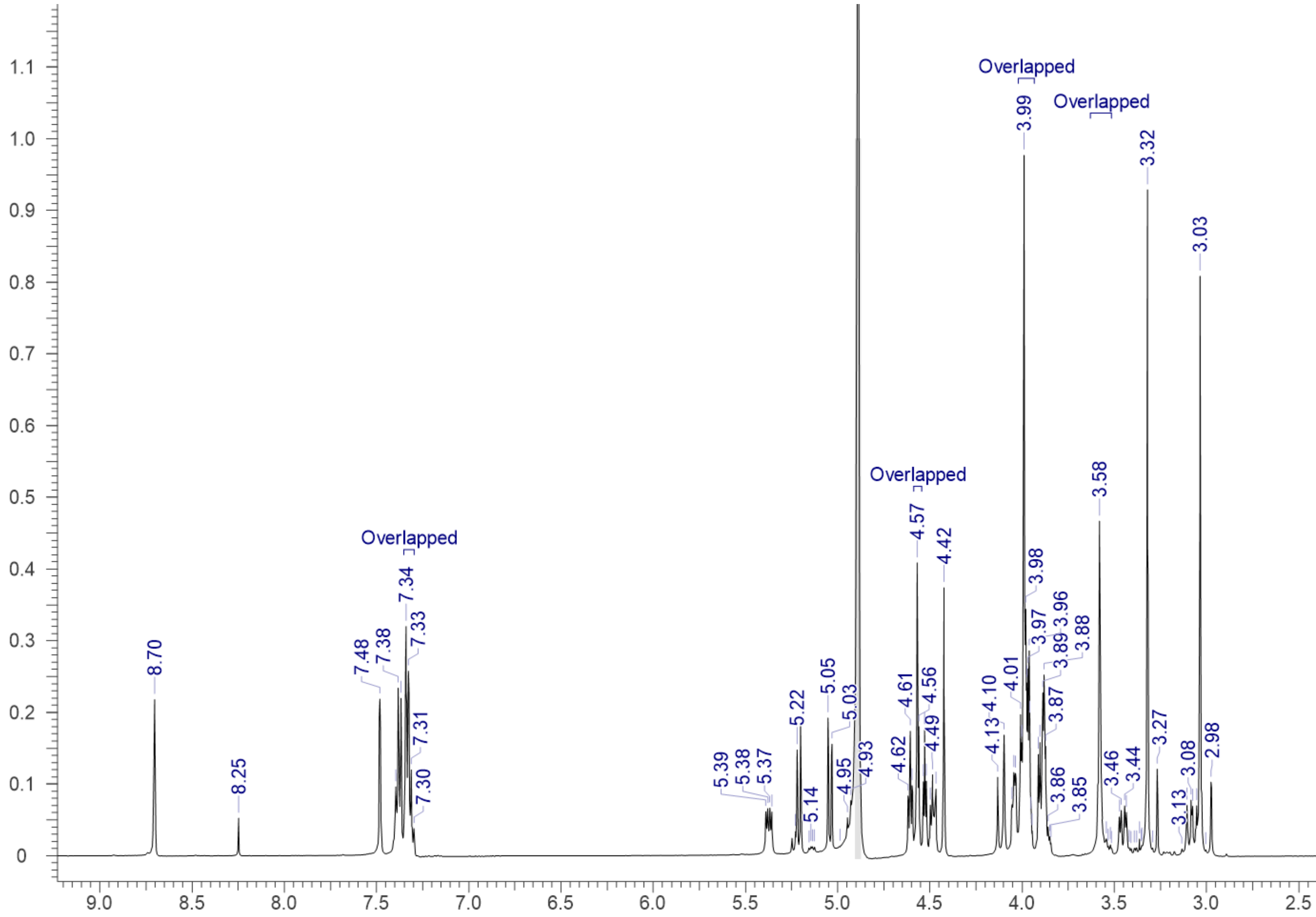

Figure **S 63** to Figure S 68 and for detailed total synthesis procedures, see section 8.

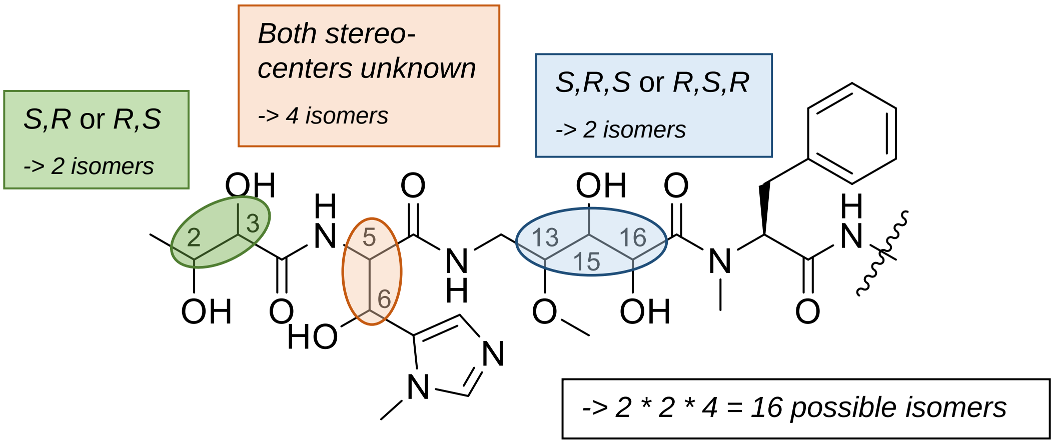

Figure S 15. *N*-terminal part of Corramycin (1). All still unknown stereocenters after configuration analysis are highlighted and the remaining possible isomers are given.

Table S 2. Overview on the configurations for all 16 synthesized Corramycin-isomers.

	Isomer No	C2	C3	C5	C6	C13	C15	C16	NMR data matching?
D-ribose series	1	*S*	*R*	*R*	*R*	*R*	*S*	*R*	no
	2	*S*	*R*	*R*	*S*	*R*	*S*	*R*	no
	3	*S*	*R*	*S*	*R*	*R*	*S*	*R*	no
	4	*S*	*R*	*S*	*S*	*R*	*S*	*R*	no
	5	*R*	*S*	*R*	*R*	*R*	*S*	*R*	no
	6	*R*	*S*	*R*	*S*	*R*	*S*	*R*	no
	7	*R*	*S*	*S*	*R*	*R*	*S*	*R*	no
	8	*R*	*S*	*S*	*S*	*R*	*S*	*R*	no
L-ribose series	9	*S*	*R*	*R*	*R*	*S*	*R*	*S*	no
	10	*S*	*R*	*R*	*S*	*S*	*R*	*S*	yes
	11	*S*	*R*	*S*	*R*	*S*	*R*	*S*	no
	12	*S*	*R*	*S*	*S*	*S*	*R*	*S*	no
	13	*R*	*S*	*R*	*R*	*S*	*R*	*S*	no
	14	*R*	*S*	*R*	*S*	*S*	*R*	*S*	no
	15	*R*	*S*	*S*	*R*	*S*	*R*	*S*	no
	16	*R*	*S*	*S*	*S*	*S*	*R*	*S*	no

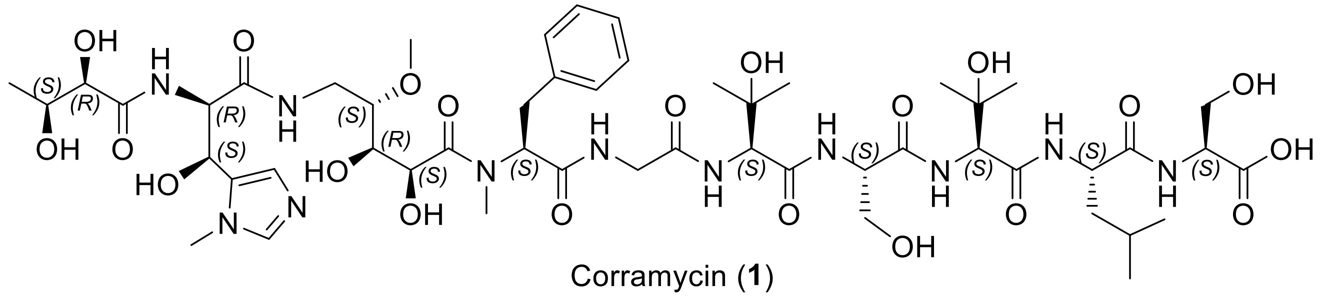

Corramycin (**1**)

Figure S 16. Absolute configuration of Corramycin (1).

Area Percent Report

Data Path : F:\Analytik\ANALYSEN11\HJ\005\
Data File : HJ005-01-02_1A1.D
Sample : Peptid (N-Me-Phe)
Acq On : 18 Feb 2011 14:31
Misc :
Integrator: ChemStation
Operator : HP2 / S1115
ALS Vial : 2
DataAcq Meth:MT_NMEPHE_SIM_10A.M

Signal : EIC Ion 162.00 (161.70 to 162.70): HJ005-01-02_1A1.D\data.ms

peak #	R.T. min	first scan	max scan	last scan	PK TY	peak height	corr. area	corr. % max.	% of total	
1	26.439	17411	17506	17683	M	28	2575	1.52%	1.496%	D N-Me-Phe
2	26.934	17700	17877	18497	M	1565	169549	100.00%	98.504%	L N-Me-Phe

Sum of corrected areas: 172124

DEFAULT.M Tue Feb 22 10:41:27 2011

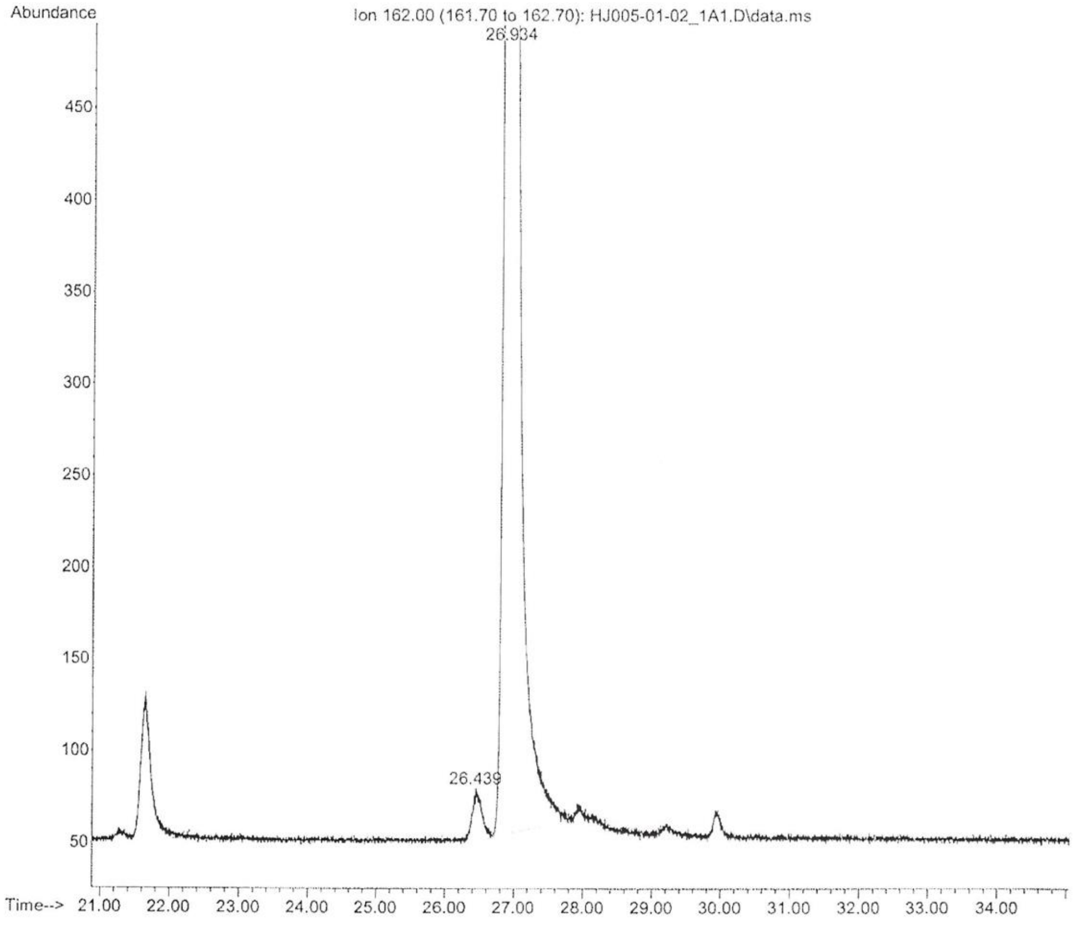

22. Feb. 2011

Figure S 17. Example for GC/MS analysis: L-*N*-Me-Phe from hydrolysate of Corramycin (**1**) (R_t = 26.934 min, 98.5 % abundance). Impurity: D-*N*-Me-Phe (R_t = 26.439 min, 1.5 % abundance).

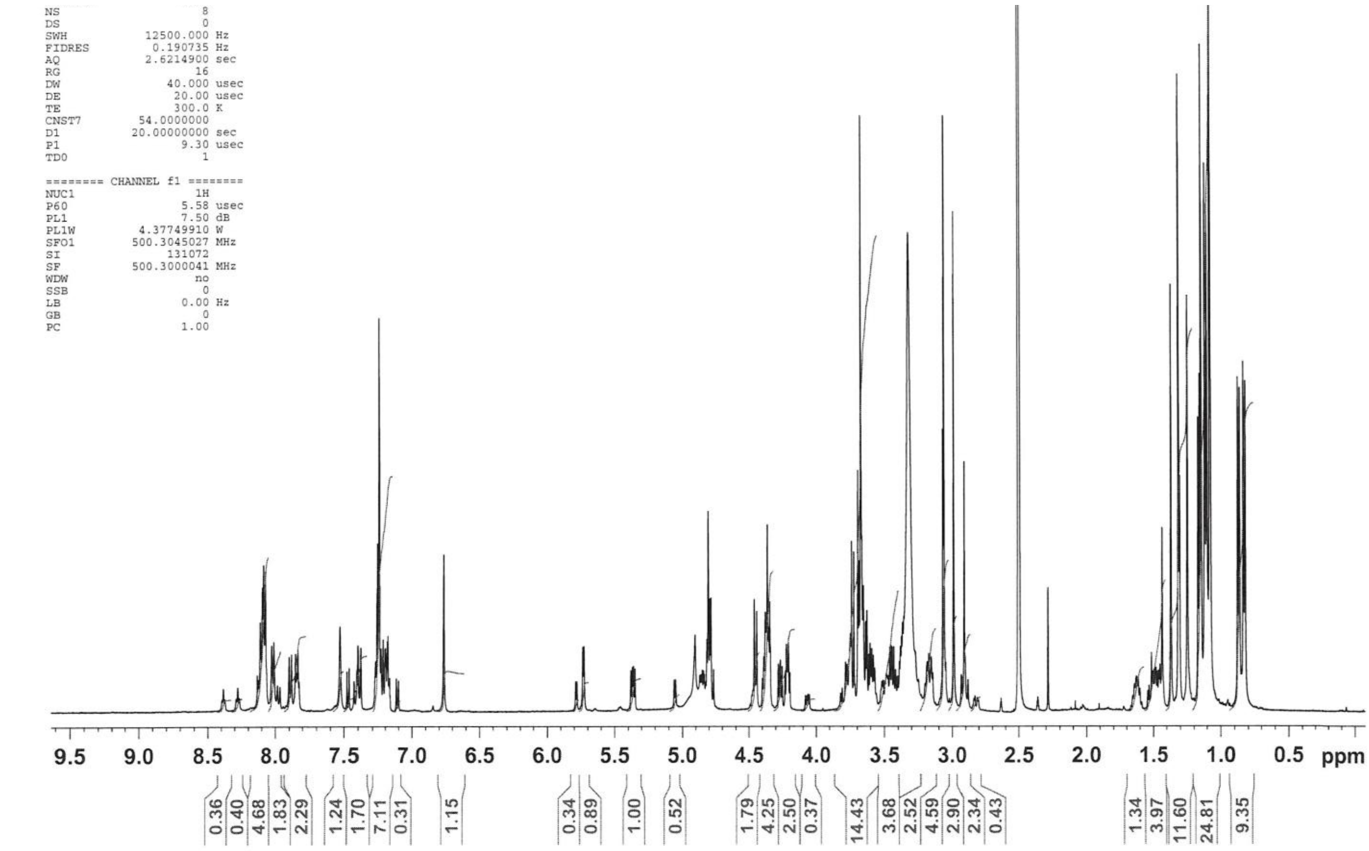

Figure S 18. ^{1}H NMR spectrum of acetonide (6), 500 MHz, DMSO-d_6.

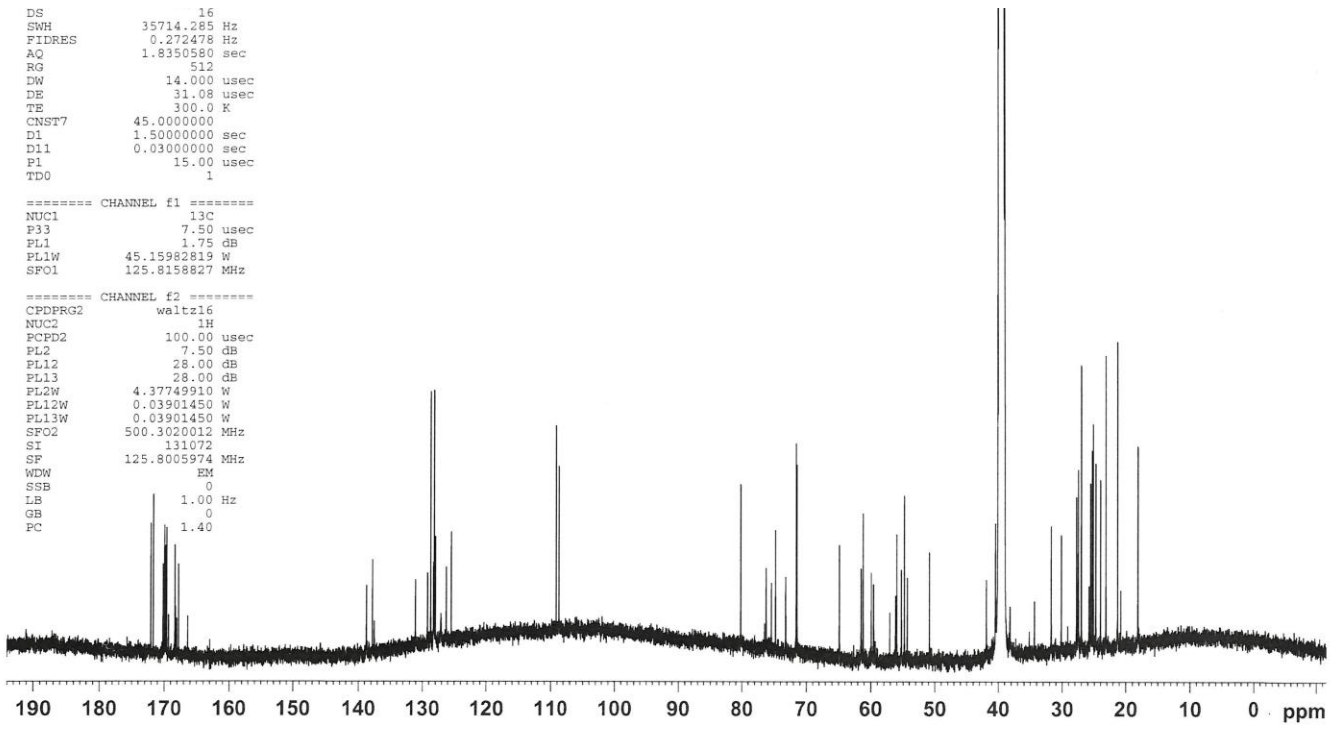

Figure S 19. ^{13}C NMR spectrum of acetonide (**6**), 125 MHz, DMSO-d_6.

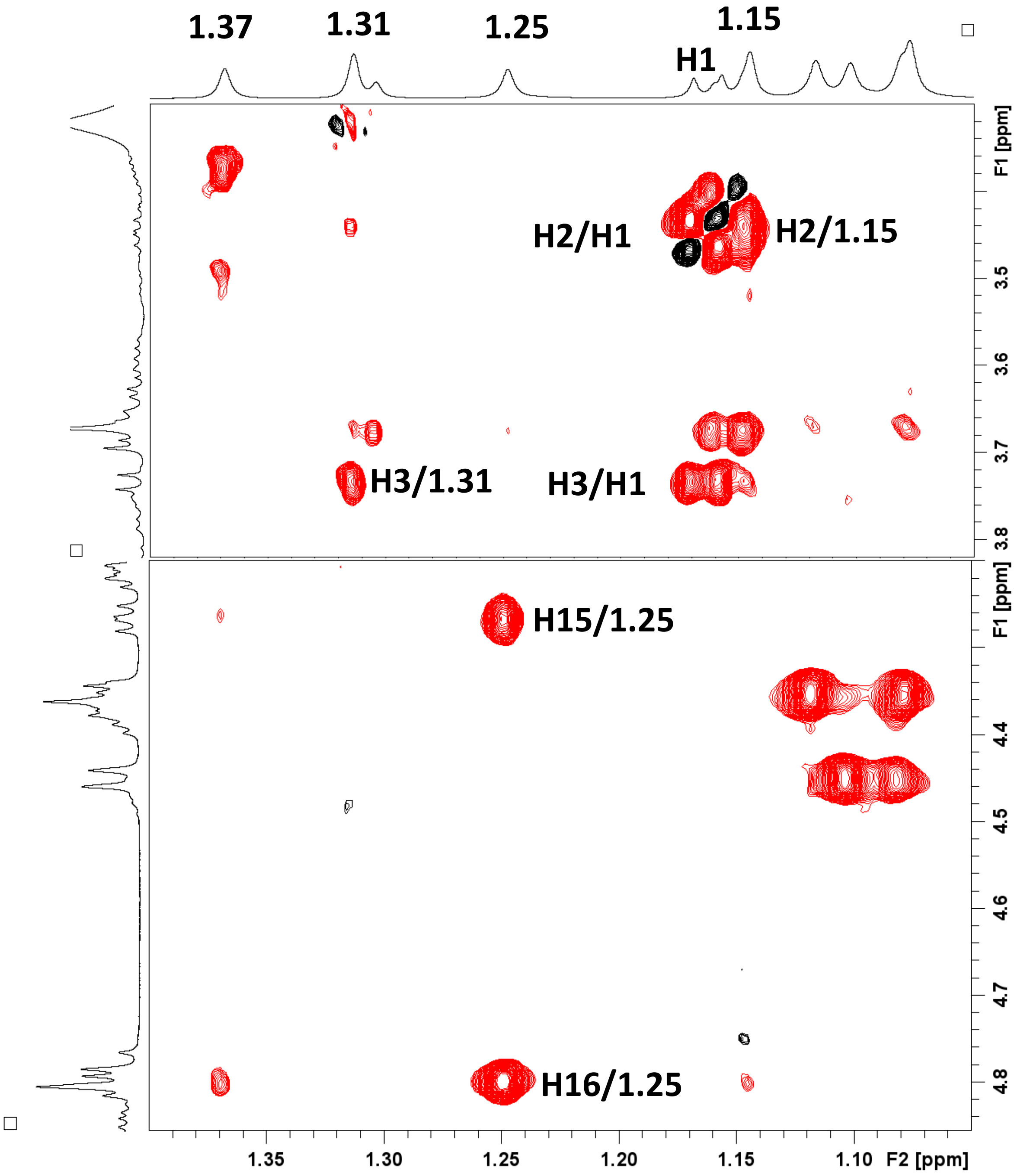

Figure S 20. ROESY NMR spectrum of acetonide (**6**), 500 MHz, DMSO-d_6.

4. Supplementation of isotope-labelled precursors during fermentation

For supplementation experiments with isotope-labelled precursors, the cultivation of *C. coralloides* MCy10984 was carried out in 50 mL unbaffled shaking flasks in 10 mL AMB medium (0.5 % (w/v) soluble starch, 0.25 % (w/v) casitone, 0.05 % (w/v) $MgSO_4 \times 7H_2O$, 0.025 % (w/v) K_2HPO_4, 10 mM HEPES, pH 7.0) for 3 days at 30 °C and 200 rpm on an orbital shaker (Multitron, Infors HT). Additionally, 0.00005 % (w/v) vitamin B_{12} and 2 % (v/v) XAD16 absorber resin were supplemented after medium sterilization. The following isotope-labelled precursors were fed at the time of inoculation reaching 1 mM end concentration: L-leucine-5,5,5-d_3, L-serine-2,3,3-d_3, L-valine-d_8, L-threonine-$^{13}C_4$-^{15}N, L-phenylalanine-d_5, L-methionine (methyl-d_3), sodium 1-^{13}C-acetate, sodium 2-^{13}C-acetate, 1,2-^{13}C-acetate, L-aspartate-$^{13}C_4$-^{15}N, glycine-2,2-d_2, β-hydroxy-L-valine-d_6 and glycerol-$^{13}C_3$.

Cells and XAD16 absorber resin were harvested by centrifugation at 3,200 × g for 20 min at 20 °C. Extraction was done two times for 60 min with 15 mL and 30 mL methanol under stirring at room temperature. Extracts were filtered using folded filter paper (8-12 µm pore size) and dried using a rotary evaporator. Dried extracts were dissolved in 100 µL MeOH and analyzed using a Dionex Ultimate 3000 RSLC system and a BEH C-18 (1.7 µm, 50 x 2.1 mm; Waters) column with an injection volume of 2 µL. Separation was achieved by a linear gradient with deionized water (A) + 0.1 % FA and acetonitrile (B) + 0.1 % FA at a flow rate of 600 µL min^{-1} and 45 °C. The gradient was initiated by a 0.33 min isocratic step at 5 % B, followed by an increase to 95 % B in 9 min to end up with a 1 min flush step at 95 % B before re-equilibration to initial conditions. The uHPLC system was coupled to an Orbitrap mass spectrometer (Thermo Scientic) by a Triversa NanoMate nano-ESI system (Advion). Mass spectra were acquired in centroid mode ranging from 200 to 2,000 *m/z* at a resolution of R = 30,000. The mass spectra are shown in Figure S 21 and Figure S 22.

Control

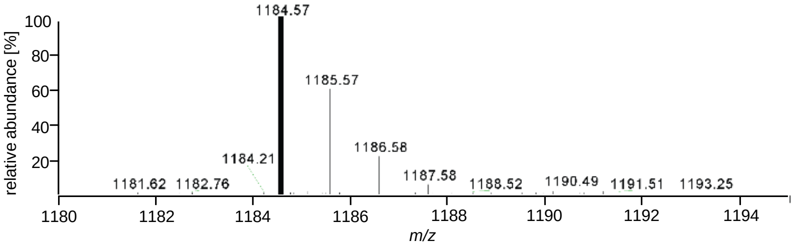

$^{13}C_2$-acetate

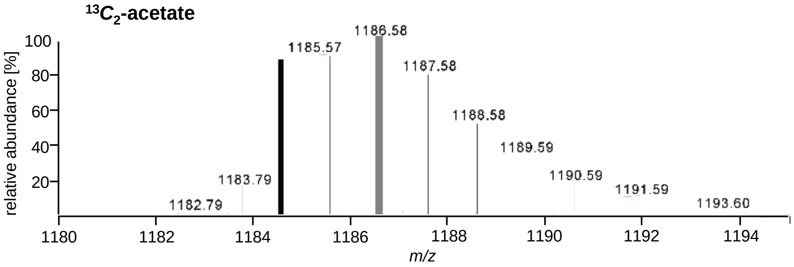

1-^{13}C-acetate

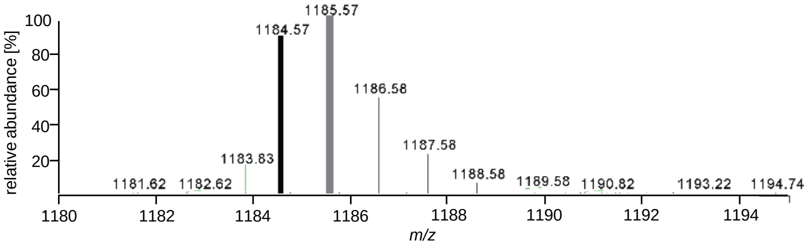

2-^{13}C-acetate

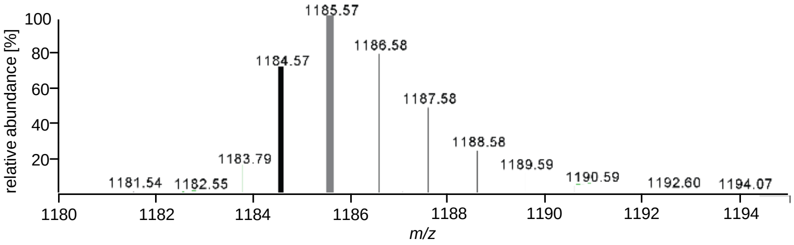

d_8-valine

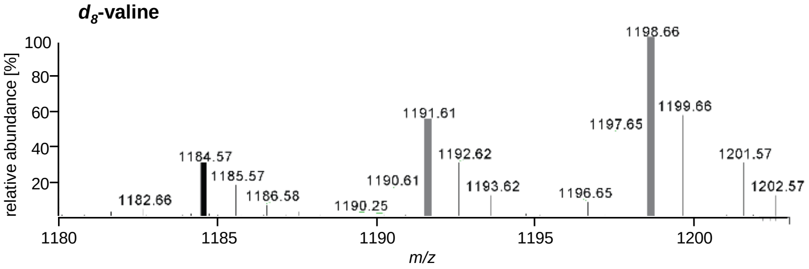

d_3-valine

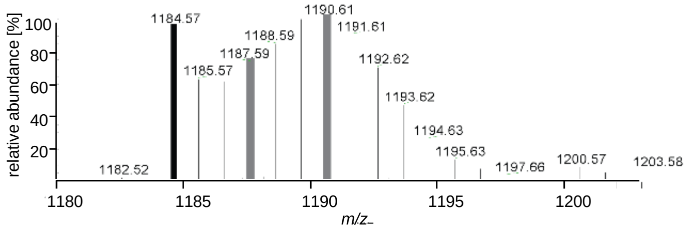

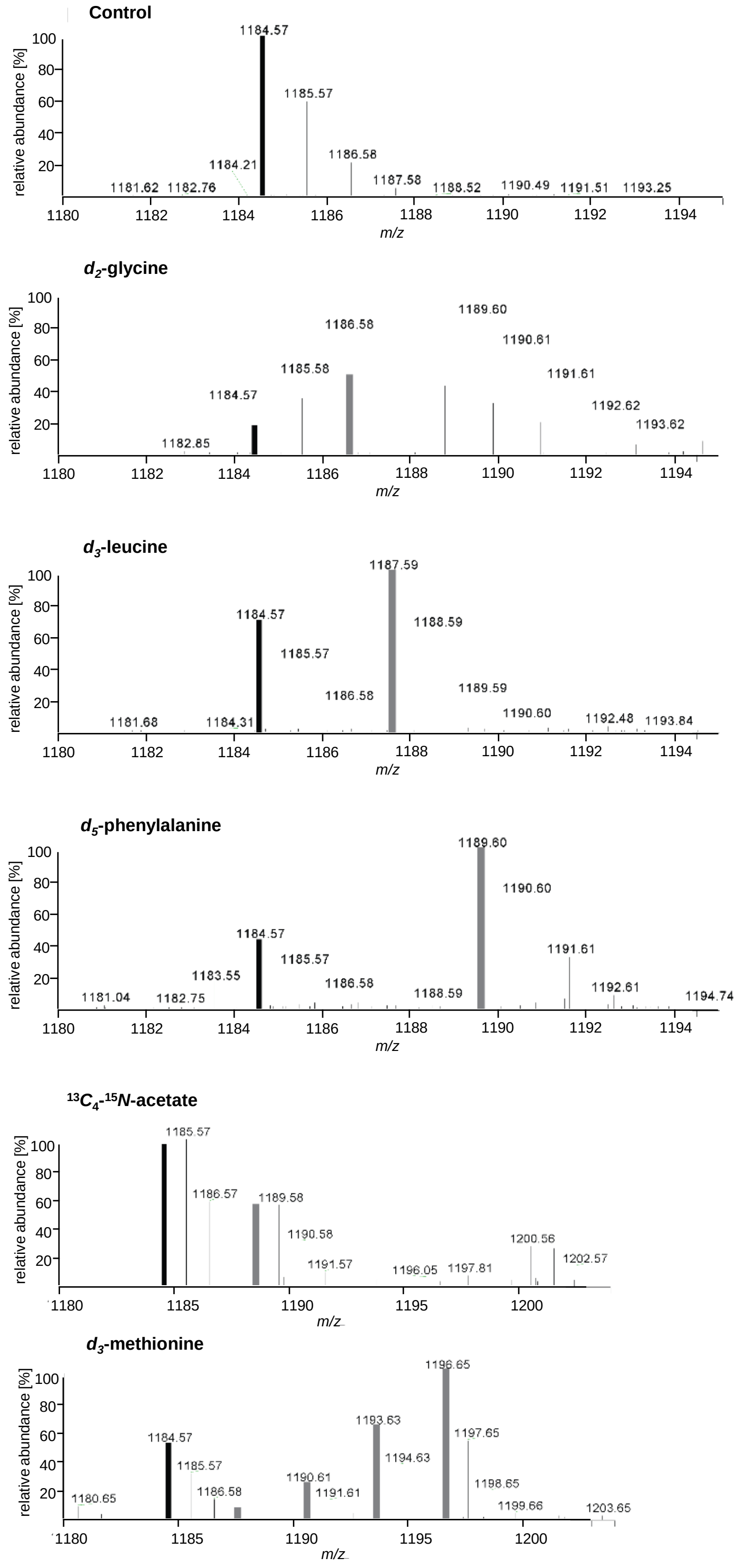

Figure S 21. Observed mass shifts after supplementation of isotope-labelled precursors during strain cultivation. The Corramycin mass peak (thick black line) and the new isotope peaks (thick grey lines) are highlighted.

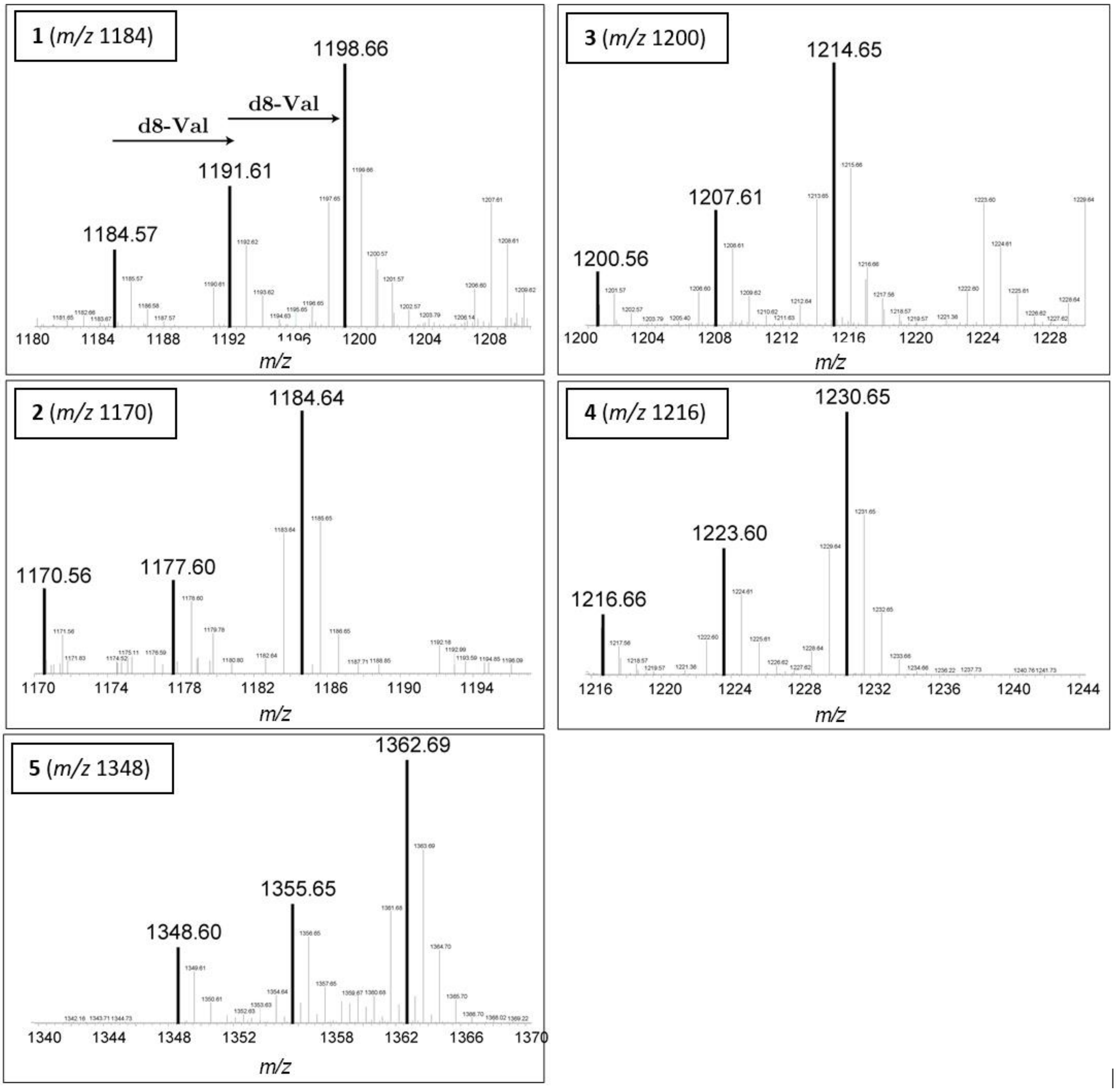

Figure S 22. d_8-L-valine incorporation into all Corramycin derivatives (**1-5**). Each single d_8-L-valine incorporation leads to a mass shift of 7.

Table S 3 depicts the observed mass shifts (Δ*m/z*), the respective moiety for which the mass shift was observed and the corresponding module catalyzing the incorporation of the moiety (left side of the Table), as well as additional Corramycin derivatives identified (right side).

Table S 3. Supplementation experiments of isotope-labelled Corramycin precursors and detection of Corramycin derivatives. On the left Δ*m/z* shows the observed mass shift in Corramycin after supplementation of the respective isotope-labelled precursor. The listed Corramycin derivatives were identified after isotope-labelled precursor supplementation. Δ*m/z* refers to the mass difference compared to Corramycin.

Supplementation				Corramycin derivatives			
**Isotope**	**Δ*m/z***	**Moiety**	**Module**	**Derivative**	***m/z***	**Δ*m/z***	**Modification**
d_3-Met	12	4 x CH_3	-	Corramycin (**1**)[a]	1184	-	
$^{13}C_4$-^{15}N-Asp	3	β-Ala	4	**2**	1170	-14	- CH_3
1-^{13}C-Ac	1	-	-	**3**	1200	+16	+ OH
2-^{13}C-Ac	1	Ac	5	**4**	1216	+32	+ 2 OH
$^{13}C_2$-Ac	2	-	-	**5**	1348	+164	+ 2 OH + pentose
d_5-Phe	5	Phe	6				
d_2-Gly	2	Gly	7				
d_8-Val	14	2 x OH-Val	8, 10				
d_3-Ser	6	2 x Ser	9, 12				
d_3-Leu	3	Leu	11				
d_6-OH-Val	-	-	-				
$^{13}C_4$-^{15}N-Thr	-	-	-				
$^{13}C_3$-Glycerol	-	-	-				

[a] Structure determined by NMR.

5. Determination of the minimal inhibitory concentration (MIC) *in vitro*

The MIC of Corramycin was evaluated following the Clinical & Laboratory Standard Institute (CLSI; https://clsi.org) and EUCAST (https://eucast.org) guidelines. First, inoculi of the bacterial test strains were prepared by culturing isolated colonies in cation-adjusted Müller Hinton broth (CAMHB; 2 g L^{-1} beef infusion solids, 1.5 g L^{-1} starch, 17.5 g L^{-1} casein hydrolysates, final pH 7.4) at 37 °C and 180 rpm for 18 h. Next, Corramycin, which was dissolved in DMSO, was transferred into the first row of a 96-well plate to reach an end concentration of 64 µg mL^{-1} (in 100 µL total volume). A 1:1 serial dilution in cation-adjusted Mueller Hinton broth (CAMHB) of the test compound was done from the top row to the bottom row of the 96-well plate to reach an end concentration of 0.125 µg mL^{-1} in the bottom row. Afterwards, the respective test strains were transferred into the wells, reaching a total volume of 100 µL and $5x10^5$ colony-forming units (CFU) mL^{-1}. After a 20 to 22 h of incubation, the optical density was evaluated on a microplate reader and the MIC endpoint was determined as the lowest concentration of antibiotic at which there was no visible growth observed. Each MIC experiment was performed in duplicates. Table S 4 depicts the MIC values of Corramycin against all bacterial strains tested in this study.

Table S 4. MIC values of Corramycin against all bacterial strains tested in this study.

**Gram-negative species**		
**Strain**		**MIC (µg mL^{-1})**
Citrobacter freundii	255041	>64
	ATCC8090	>64
Enterobacter cloacae	17059482	>64
	DSM46348	32
Enterobacter aerogenes	DSM12058	>64
Klebsiella pneumoniae	1705966	>64
	1705949	64
	ATCC13883	16-64
Acinetobacter baumanii	ATCC19606	64
	1705943	>64
	1705936	>64
Pseudomonas aeruginosa	ATCC9027	>64
	ATCC27853	>64
	DSM46317	>64
	ATCCBAA-47	>64
	ATCC27853	>64
	1705886	>64
	1705904	>64
	PAO1	>64
Bordetella bronchiseptica	NCTC8344	>64
Burkholderia cepacia	ATCC25416	>64
Stenotrophomonas maltophilia	ATCC13637	>64
	255074	>64
Serratia marcescens	255067	>64
	ATCC13880	>64
Proteus mirabilis	ATCC29906	>64
Proteus vulgaris	DSM46228	>64
**Gram-positive species**		
**Strain**		**MIC (µg mL^{-1})**
Enterococcus faecium	A6349	>64
	DSM17050	64
Enterococcus faecalis	1069VanB	16
Staphylococcus aureus	11540	>64
	ATCC33592	>64
	NRS643	>64
Streptococcus pneumoniae	02J1175	>64
	ATCC700671	>64

6. Investigation of the cross-resistance of Corramycin to clinically used antibiotics

The cross-resistance of Corramycin to Tobramycin, Cefotaxim, Cefoxitin, Ciprofloxacin, Tetracycline and Rifampicin was investigated by determination of the MIC according to the protocol described in the previous section. The MIC of the respective antibiotics against 16 clinical isolate *E. coli* strains, which were obtained from P. Nordmann's collection and were classified Carbapenem-resistant, Aminoglycoside-resistant or Quinolone-resistant, was determined (Table S 5).

Table S 5. Cross-resistance of Corramycin against tobramycin, Cefotaxim, Cefoxitin, Ciprofloxacin, Tetracycline and Rifampicin based on a MIC assay. ESBL: extended-spectrum β-lactamase; CP: carbapenem; AG: aminoglycoside; QL: quinolone; PRP

E. coli	1	2	3	4	5	6	7	8	9	10	11	12	13	14	15	16
isolate	*CTX-M-1*	*CTX-M-2*	*OXA-30*	*SHV-2a*	*TEM-3*	*IMP-1*	*IMP-4*	*KPC-2+TEM-1+OXA-9*	*OXA-48*	*OXA-48+ CTX-M-15*	*RMT-B*	*ARM-A*	*RMT-C*	*qep A*	*qnr A1*	*Qnr S1*
Resistance	ESBL	ESBL	ESBL-CP	ESBL	ESBL	ESBL-CP	ESBL-CP	ESBL-CP	ESBL-CP	ESBL-CP	AG	AG	AG	QL	QL	QL
Corramycin	8	4	16	4	8	8	4	8	>64	16	>64	16	32	64	2	8
Tobramycin	2	2	2	32	16	2	16	8	32	1	1	>64	>64	0.5	32	32
Ciprofloxacin	32	<0.125	<0.125	16	<0.125	<0.125	<0.125	>64	64	<0.125	<0.125	>64	>64	>64	1	32
Cefotaxim	>64	64	1	64	64	0.25	>64	>64	>64	>64	>64	>64	>64	>64	32	>64
Cefoxitin	8	4	4	4	4	16	>64	64	16	16	16	8	>64	8	8	8
Tetracyclin	>64	32	>64	64	2	>64	2	>64	>64	64	64	>64	>64	4	4	>64
Rifampicin	32	32	32	16	16	16	16	32	64	16	16	>64	32	32	>64	32

7. Isolation of *C.* coralloides MCy10984 and ST201330 genomic DNA and illumina sequencing

For genomic DNA isolation, *C. coralloides* ST201330 and *C. coralloides* MCy10984 were cultivated in 50 mL AMB medium (0.5 % (w/v) soluble starch, 0.25 % (w/v) casitone, 0.05 % (w/v) $MgSO_4 \times 7H_2O$, 0.025 % (w/v) K_2HPO_4, 10 mM HEPES, 0.00005 % (w/v) vitamin B_{12} and 2 % (v/v) XAD16 absorber resin (the latter two were supplemented after medium sterilization), pH 7.0) for 5 days at 30 °C and 180 rpm on an orbital shaker (Multitron, Infors HT). The cultures were harvested by centrifugation at 3,200 × g for 15 min at 4 °C and the cell pellet was resuspended in 5 mL 10 mM Tris-HCl (pH 7.5) plus 300 µL Proteinase K solution (10 mg mL^{-1}) and 600 µL SDS-solution (10 %). The mixture was incubated at 55 °C for 2 h. Subsequent extraction using phenol:chloroform:isoamylalcohol (P:C:I; 25:24:1) was conducted three times: One volume of P:C:I was added and the mixture was incubated at room temperature for 60 min under constant tube invertation (5 rpm). After centrifugation at 3,200 × g for 10 min at room temperature, the upper phase was extracted again. A fourth extraction of the upper phase with C:I (24:1) was performed prior to addition of 1/10 volume of 3 M Na-acetate (pH 5.5) and 2.5 volumes of ice-cold 100 % EtOH. The precipitated DNA was washed once in 1 mL of 70 % EtOH and subsequently air-dried overnight. The DNA-pellet was resuspended in 500 µL of 10 mM Tris-HCl buffer (pH 7.5).

Illumina sequencing of the genomic DNA of *C. coralloides* ST201330 was performed in cooperation with SEQ-IT GmbH & Co. KG (Germany, 67655 Kaiserslautern). Raw sequencing data obtained from the MiSeq platform comprised 26,054,366 paired-end reads and 14,483,850 mate-pair reads. For both libraries the read length was 250 bp. Assembly into contigs with Abyss-pe assembler (version 1.3.6), resulted in 26 sequences with a total length of 10,243,981 bp. Genome sequencing of strain MCy10984 was performed using Illumina sequencing technology (in-house service). Raw sequencing data obtained from the MiSeq platform comprised 9,509,890 paired-end reads with the length of 250 bp each. Assembly of the raw sequencing data resulted in 59 sequences with a total length of 11,107,395 bp.

8. *In silico* analysis of the Corramycin BGC

Geneious v10.1.3 (Biomatters Ltd.) was used for routine DNA and protein sequence analysis tasks. BLAST (basic local alignment search tool; https://blast.ncbi.nlm.nih.gov/Blast.cgi), antiSMASH (a comprehensive resource for the genome mining of BGCs)[3] and MUSCLE alignment[4] were used to compare protein sequences of the cluster with sequences of characterized protein members of the same family. The prediction of NRPS A domain substrate specificities was performed using NRPSpredictor2.[5] Construction of phylogenetic trees was done with NaPDOS online server.[6]

First, the borders of the Corramycin BGC were determined by comparing genome sequences of the two producer strains, *C. coralloides* MCy10984 and *C. coralloides* ST201330, with the non-producer strain *C. coralloides* DSM2259 using MUSCLE alignment (Figure S 23).[4] The BGC represents a well-defined insertion into the genome of *C. coralloides* and has a size of 59 kb with a GC content of 71 %. Fifteen genes are presumably involved in Corramycin biosynthesis. Based on the coding strand on which the genes are located and the small intergenic regions between adjacent genes on the same coding strand, we assume that the BGC is organized in five putative transcriptional units: *comABC*, *comDEF*, *comG*, *comH* and *comIJKLMNO*.

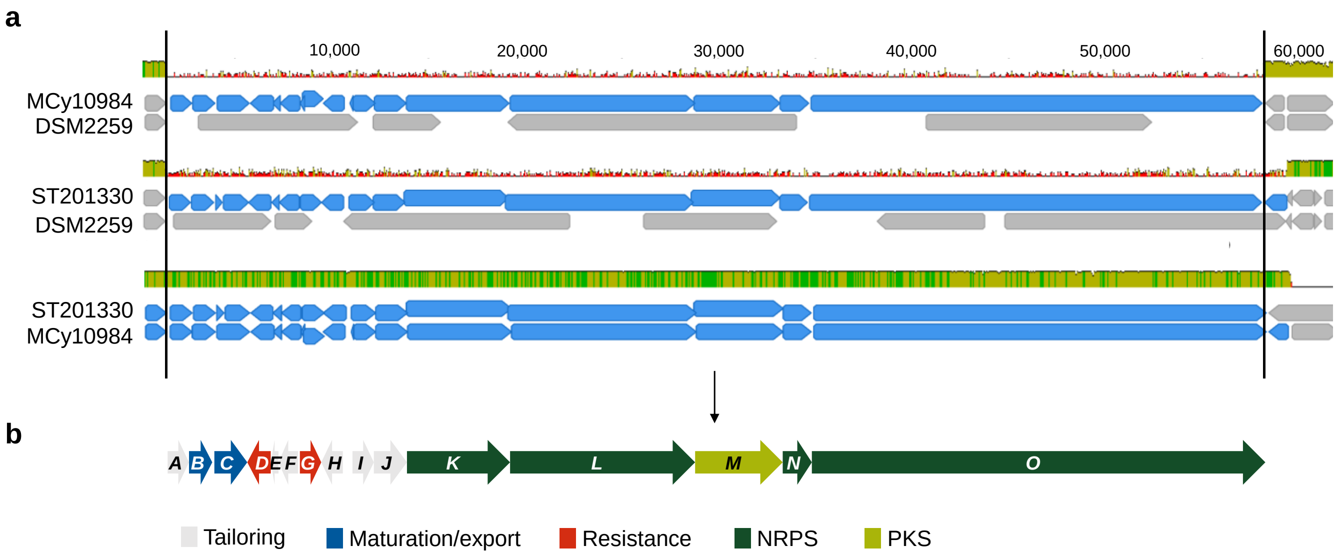

Figure S 23. Determination of the Corramycin BGC borders. a: MUSCLE alignments of the BGCs and adjacent genomic regions from *C. coralloides* Mcy10984 and ST201330 with the genome of the non-producer strain *C. coralloides* DSM2259. Arrows show putative genes that belong (blue) or do not belong (grey) to the Corramycin BGC based on the respective alignment. The cluster borders were determined by integrating data from all three alignments and are depicted as black lines. b: Corramycin BGC and putative functions of the encoded proteins. Parts of the figure were generated with Geneious (version 2020.0 created by Biomatters; available from https://www.geneious.com).

Next, we analyzed each gene potentially belonging to the BGC by using the BLAST algorithm to identify the closest homologs and propose the biosynthetic function (Table S 6).

Table S 6. Proposed function of the biosynthetic genes involved in Corramycin biosynthesis.

Gene	Size (aa)	Proposed function		Predicted domain specificity	Homolog	Identity (%)
comA	370	JmjC domain containing protein, hydroxylase			JMJ30	25
comB	378	o-Phtalyl amidase / hydrolase			P0C2Y0	25
comC	424	Flippase-like exporter (membrane-bound)			arnF	31
comD	383	DNA replication and repair			RecF	38
comE	130	Aspartate decarboxylase			panD	78
comF	342	Luciferase-like monooxygenase			BtrO	27
comG	364	Kinase			murA	26
comH	388	JmjC domain containing protein, hydroxylase			KDM8	32
comI	372	Limonene 1,2-monooxygenase			limB	24
comJ	524	Vitamin B_{12}-dependent radical-SAM methyltransferase			bchE	26
comK	5.379	NRPS	M1 (FAAL-ACP)	Acyl		
			M2 (C-HAD-T)	D-1,3-BPG		
comL	9.690	NRPS	M3 (C-A-MT-T-E)	L-His		
			M4 (C-A-MT-T)	β-Ala		
comM	4.554	PKS	M5 (KS-AT-KR-ACP)	Ac		
comN	1.419	NRPS	M6 (C)	L-Phe		
comO	23.328	NRPS	M6 (A-MT-T)			
			M7 (C-A-T)	Gly		
			M8 (C-A-T)	L-Val		
			M9 (C-A-T)	L-Ser		
			M10 (C-A-T)	L-Val		
			M11 (C-A-T)	L-Leu		
			M12 (C-A-T-TE)	L-Ser		

BLAST analysis of *comA* found cupin-like transcription factors as closest homologs. However, *InterPro* scan[7] showed that ComA presumably contains a JmjC domain, which was shown to be involved in the hydroxylation of aspartate residues.[8] The JmjC domain was also described in JmjC domain-containing factor inhibiting hypoxia, which shows homology to cupin-like metalloenzyme domains.[9] Furthermore, ComA showed homology to clavaminate synthase-like 2-oxoglutarate-dependent dioxygenase with an active center containing Fe (II). Including this, ComA is most probably a hydroxylase. Likewise, *comF*, *comH* and *comI* encode for various hydroxylases. The mentioned hydroxylases are presumably catalyzing the β-hydroxylation of L- or D-histidine, L-valine and the two hydroxylations of β-alanine and the PKS incorporated acetate moiety.

The genes *comB* and *comC*, encoding a putative hydrolase and flippase-like exporter and might potentially be involved in a hypothetical pre-drug mechanism involving fatty acid-bound Corramycin as hypothesized in the main text. ComD shows homology to RecF, a protein involved in the repair of DNA double-strand breaks in *E. coli,*[10] making it a candidate for a potential Corramycin self-resistance mediator and indicating that the Corramycin mechanism of action might be linked to DNA double-strand breaks. The potential function of ComE as a β-alanine decarboxylase involved in the biosynthesis of the 5-amino-2,3-dihydroxy-4-methoxy-pentanoyl moiety was discussed in the main text. ComG is homologous to members of the protein-kinase superfamily and might also be related to self-resistance development by inactivation of Corramycin through phosphorylation similar to aminoglycoside resistance mechanism. ComJ shows similarity to radical SAM methyltransferases and might be involved in the biosynthesis of the *N*-terminal Corramycin *C*-4 butyric acid moiety as proposed in the main text.

The Corramycin NRPS-PKS assembly line, encoded by *comKLMNO*, includes three methyltransferase (MT) domains. Two *N*-MT domains were identified in modules 3 and 6 embedded in the respective A domains. The third MT is located downstream of module 4 and was not annotated automatically by standard NRPS prediction tools. A BLAST search of module 4 revealed a stretch of approximately 300 amino acids as an FkbM-like methyltransferase. The three known members of this MT protein family are all stand-alone *O*-MTs.[11] Phylogenetic analysis of the three MTs in the Corramycin assembly line with 54 MTs using the NaPDoS online server[6] showed that the *N*-MT of module 6 clusters with NRPS-associated *N*-MTs (Figure S 24) that have been shown to catalyze methylation of amide nitrogens.[12] This is in accordance with the Corramycin structure, since the A domain of module 6 is specific for phenylalanine (Table S 6) and the phenylalanine moiety is *N*-methylated. The designated *N*-MT of module 3 does not cluster with any known MT domain found in natural product biosynthetic pathways. Thus, we assume that this domain catalyzes the *N*-methylation of histidine. So far no NRPS-associated *in cis* *N*-MT acting on an aromatic substrate was described. Furthermore, we assume that the stand-alone *O*-MT of module 4 catalyzes the *O*-methylation of the β-alanine moiety.

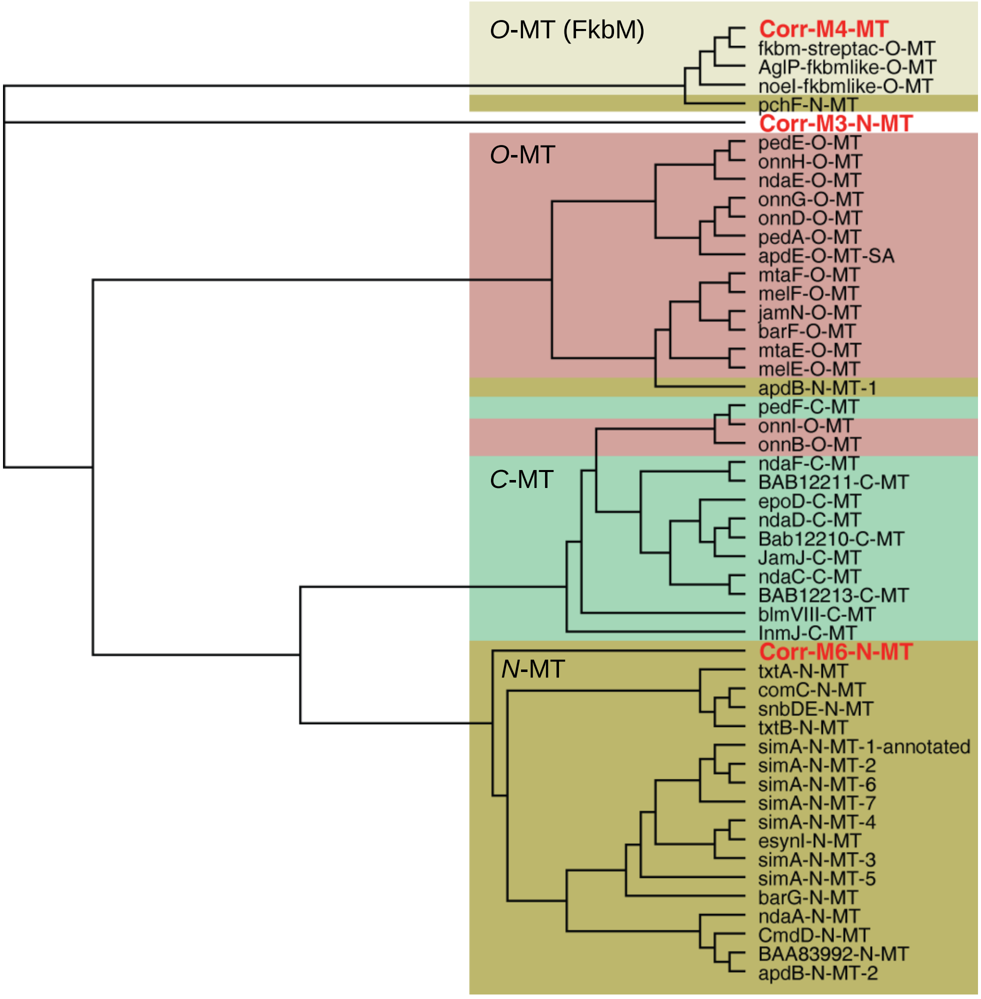

Figure S 24. Phylogenetic analysis of the Corramycin assembly line MT domains. The phylogenetic tree was generated by NapDos online server.[6]

A phylogenetic analysis of the Corramycin assembly line C and E domains was performed to elucidate their functional subtypes and predict the stereochemistry of the incorporated building block (Figure S 25). The C domains of modules 2, 3 and 6-12 cluster with other $^{L}C_{L}$ domains, indicating that the respective building blocks incorporated are L-amino acids, except for the module 7 incorporated glycine, which is non-chiral. The module 3 E domain clusters with other E domains and the module 4 C domain clusters with $^{D}C_{L}$ domains.

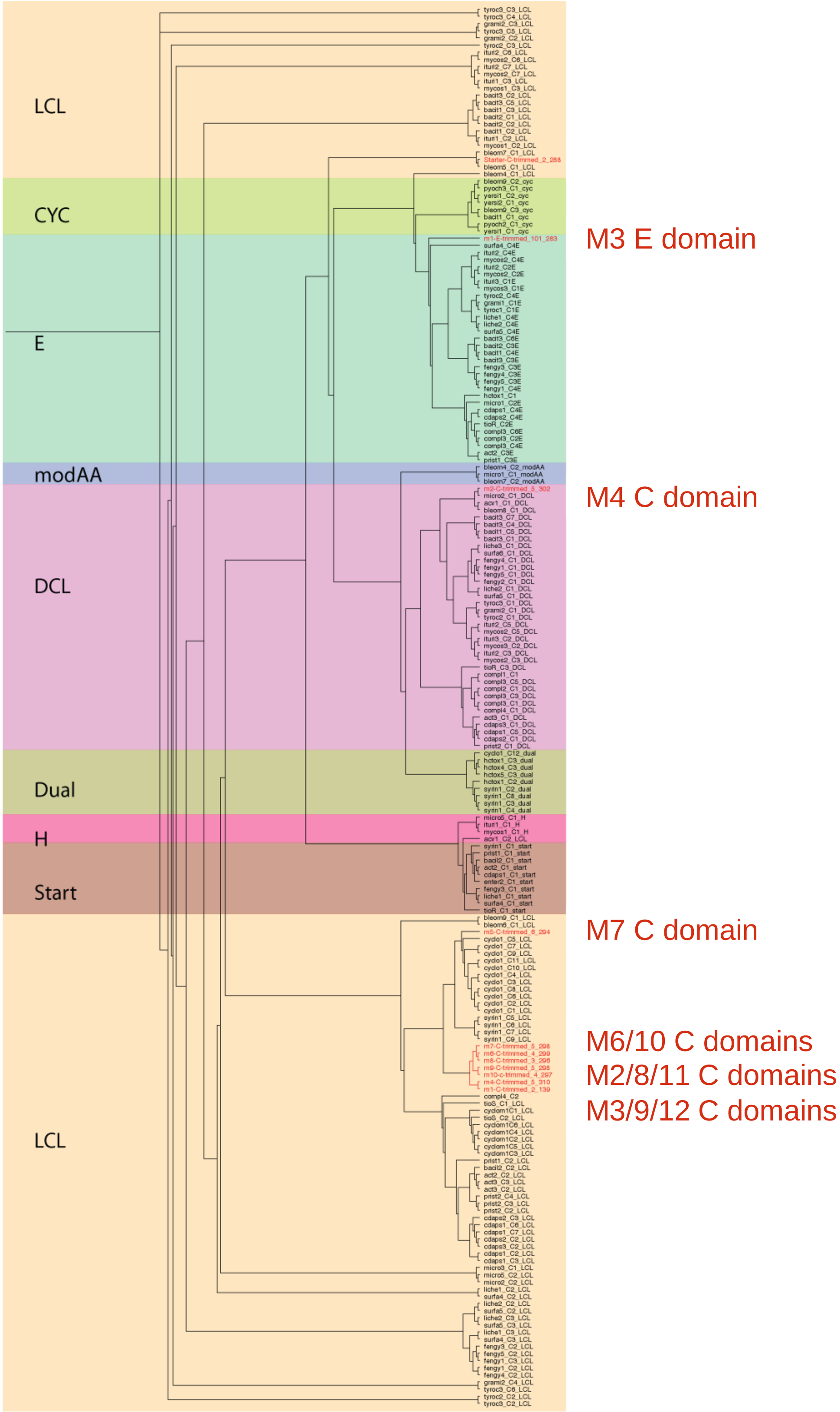

Figure S 25. Phylogenetic tree of the Corramycin assembly line C and E domains. The phylogenetic tree was generated by NapDos online server.[6]

9. *In silico* analysis, protein purification and malachite green assay of the FAAL

FAAL homologs were identified using BLAST with the Corramycin FAAL sequence as query. The respective amino acid sequences were compared by using the MUSCLE alignment[4] algorithm implemented in *Geneious* v10.1.3 software (Figure S 10). Since no acylated Corramycin analogs were detected in the fermentation broth extracts, initially, we aimed to examine if the FAAL domain was indeed active and to investigate its substrate specificity. Therefore, we purified the FAAL domain and amplified the FAAL-encoding sequence from genomic DNA of MCy10984 via PCR according to standard protocols[13] using the primers FAAL-FP (AAAAAAACATGTTGGACCTCGCTCGGA) and FAAL-RP (AAAAAAAAGCTTCTACGCCGG CGAG). The PCR construct was cloned into a petM44 expression vector with an N-terminal 6xHis-MBP tag and heterologously expressed in *E. coli* BL21 (DE3) cells. Cultivation was performed in 500 mL LB (1 % (w/v) tryptone, 0.5 % (w/v) NaCl, 0.5 % (w/v) yeast extract, pH 7.6) at 16 °C for 16 h after gene expression by addition of 0.1 mM IPTG. Afterwards the culture was harvested at 3,200 × g for 10 min at 4 °C and the cell pellet was resuspended in lysis buffer (150 mM NaCl, 25 mM Tris, 40 mM imidazole, pH 7.5), sonicated and centrifuged under the same conditions as before. The supernatant was loaded onto a gravity flow column containing NiNTA-loaded sepharose and washed with lysis buffer prior to elution using imidazole (250 mM. The MBP tag was cleaved using HRV3C protease during overnight dialysis against SEC buffer (150 mM NaCl, 25 mM Tris, pH 7.5) at 4 °C, and removed by a second Ni-NTA chromatography step. After passing through a Superdex 200 16/60 pg column (GE Healthcare Life Sciences), the protein was concentrated using a 30 kDa cutoff filter and stored at -80 °C in 10 % glycerol. Protein purity was determined by SDS-PAGE. Protein concentration was determined spectrophotometrically upon determining the respective extinction coefficient from the amino acid sequence using the PROTPARAM webserver (http://web.expasy.org/protparam/).[14]

In addition to that, a malachite green assay was performed as described previously[15] to determine the substrate specificity of the FAAL domain. 4 µM FAAL was incubated with 2 µM ATP and 2 µM linear fatty acid (C_6-C_{13}) in reaction buffer (150 mM NaCl, 25 mM Tris-HCl, pH 7.5) containing inorganic phosphatase at 37 °C for 1 h. The reaction was stopped by adding 20 µL malachite green solution and the absorption at 625 nm was measured after 10 min. The relative increase of absorption was calculated based on a negative control without fatty acid substrate. Therefore, all reactions were performed in triplicates. We observed activation of numerous linear fatty acids with different lengths by the FAAL domain (Figure S 26).The highest activity was observed for decanoic acid and the spectrum of activated fatty acid ceased at tridecylic acid and hexanoic acid. Moreover, the FAAL has a substrate specificity for long-chain fatty acids ranging from 6 to 13 *C*-atoms

The broad substrate tolerance of the FAAL is not a surprising finding since lipopeptides often occur with various fatty acid chain lengths on the same peptide backbone.[16] However, even upon re-inspection we did not find fatty acid-linked Corramycin derivatives in the extracts of the producer strains.

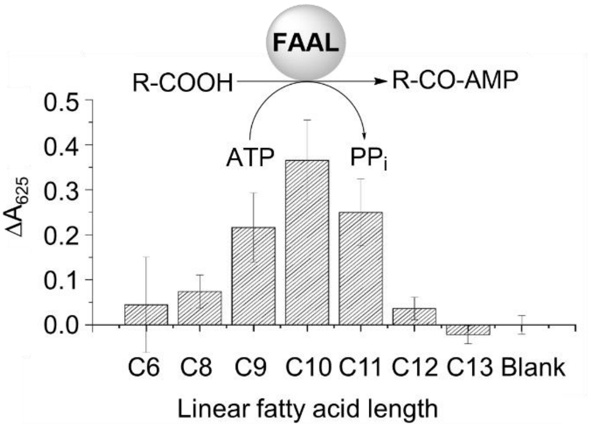

Figure S 26. Malachite green assay shows activation of numerous linear fatty acids with different lengths ranging from C6 to C13 by the FAAL domain.

There are numerous examples of lipopeptides harboring FAAL-incorporated fatty acid residues which are vital for their bioactivity.[17] On the contrary, in the biosynthesis of the myxobacterial lipopeptide Vioprolide, a fatty acid-linked precursor is biosynthesized and the maturation of the active product is achieved by hydrolysis of the fatty acid.[18] Since the Corramycin FAAL domain is evidently active, we speculated that inactive, acylated Corramycins are produced in terms of self-protection of the producer strain and, similarly to Vioprolide biosynthesis, subsequently hydrolyzed to their active form. However, in contrast to the Vioprolide case, we could thus far not identify any acylated Corramycin precursors and, consequently, further investigations are required in future experiments.

10. Total synthesis of Corramycin

10.1 Retrosynthetic Analysis and Synthetic Strategy Overview

Retrosynthetic analysis disconnected the peptidic part on the right part of the molecule including the unusual Hydroxyl-Valine **10** and the different building blocks as the "ribo-Phe" moiety **9**, the δ-*N*-methyl-β-hydroxy histidine **8** and the dihydroxybutyric acid **7** (Figure S 27). A solid-phase peptidic approach (SPPS) starting from the first L-Serine seemed to be obvious by using traditional Fmoc-strategy and adequate protecting groups on the different hydroxyl groups[19].

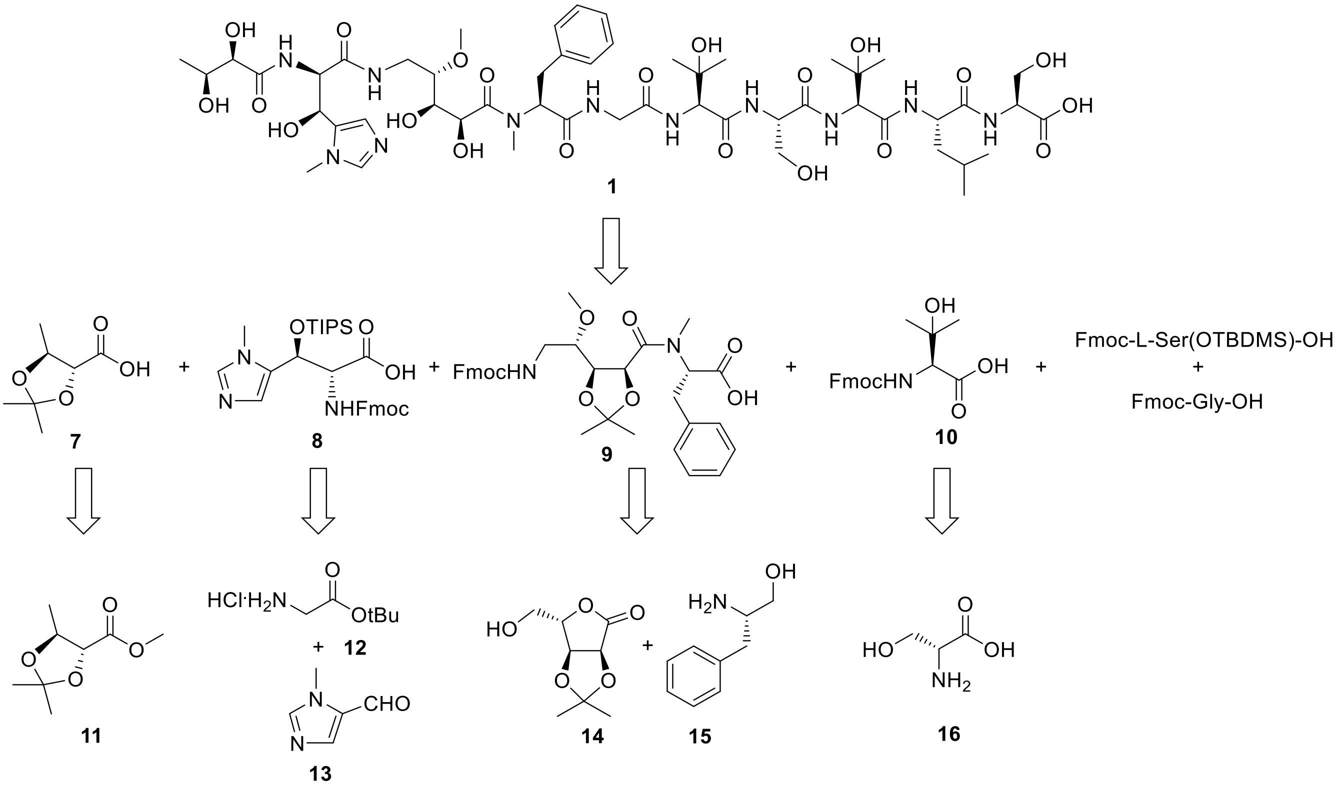

Figure S 27. Retrosynthetic analysis of Corramycin (1).

<u>10.1.1 General procedure for preparation of compound (*S*)-2-((((9H-fluoren-9-yl)methoxy)carbonyl)amino)-3-hydroxy-3-methylbutanoic acid (10)</u>

The first building block **10** was easily accessible by using a described strategy starting from D-Serine (Figure S 28)[20]. The esterification of the acid by a methyl group followed by Fmoc protection of the amino group furnished the intermediate **18**. Then the addition of the Grignard reagent MeMgBr to the ester delivered the tertiary alcohol and final oxidation of the alcohol the building block **10** in an acceptable overall yield (33 % for 4 steps).

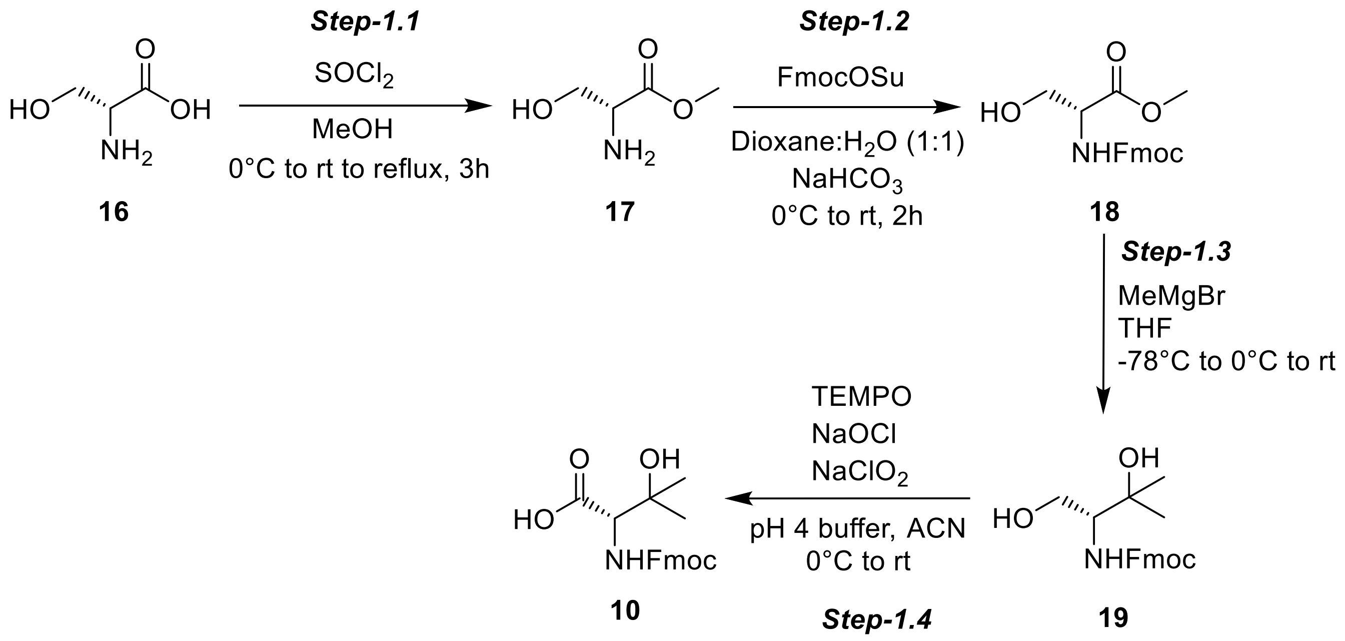

Figure S 28. Synthetic strategy towards building block 10[16].

10.1.2 General procedure for preparation of compound (*S*)-2-((4S,5S)-5-((S)-2-((((9H-fluoren-9-yl)methoxy)carbonyl)amino)-1-methoxyethyl)-N,2,2-trimethyl-1,3-dioxolane-4-carboxamido)-3-phenylpropanoic acid (9)

More steps were necessary for the synthesis of the second building block **9** (Figure S 29). Protected and commercial derivative of L-Ribose presented the advantage to have 3 stereocenters already fixed. After transformation of the free alcohol as leaving group, azido was incorporated to furnish compound **21**. Then lactone opening with derivative of phenylalanine **20** obtained from compound **15** delivered intermediate **23** with 57 % yield. Methylation of the free alcohol and amide followed by deprotection of the primary alcohol with TBAF and oxidation furnished acid **26**. Final transformation of azido into Fmoc-protected amine under hydrogenation conditions allowed to obtain building block **9** in an overall acceptable yield (12 % for 7 steps).

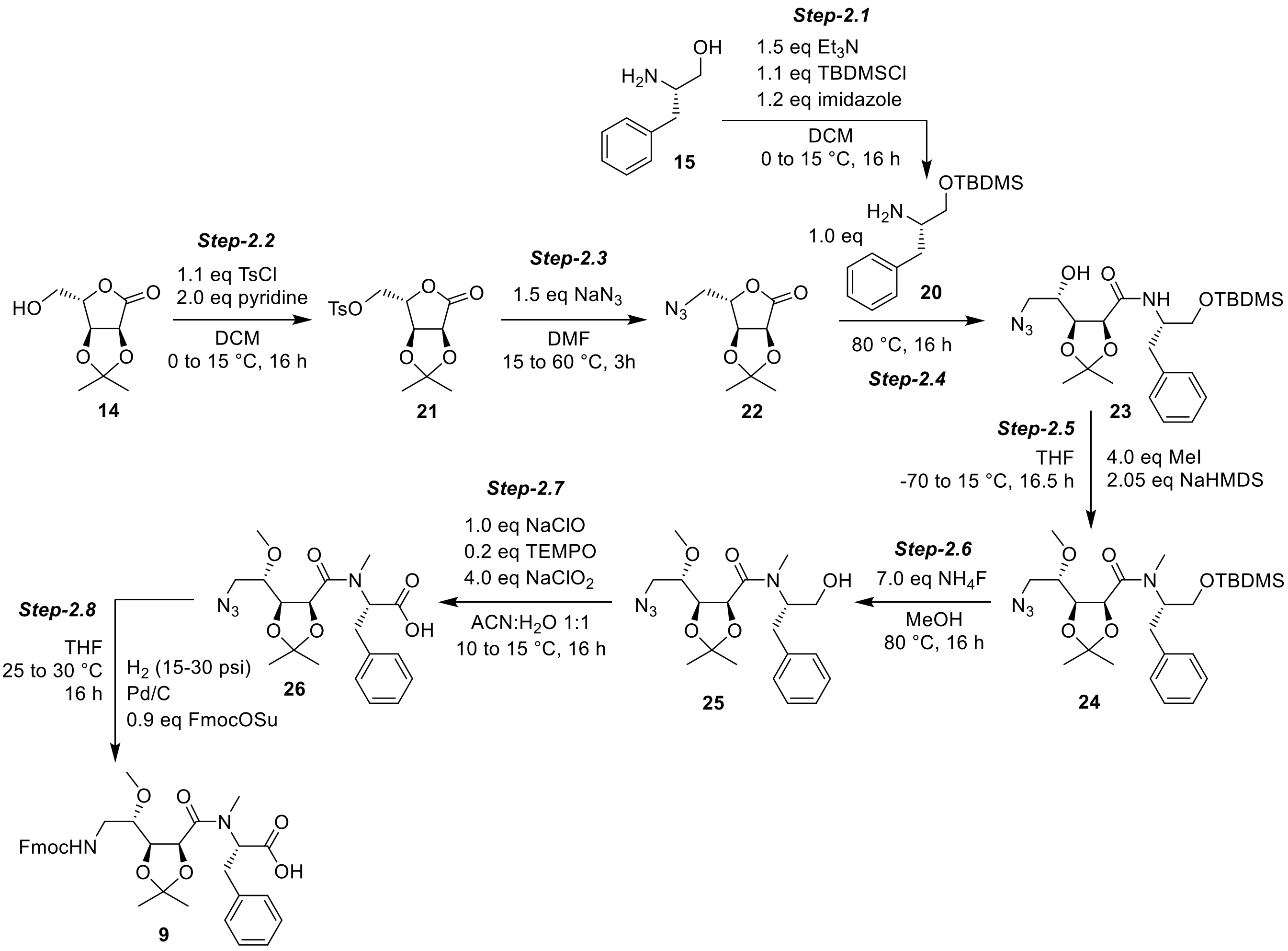

Figure S 29. Synthetic strategy towards building block 9.

10.1.3 General procedure for preparation of compound (2*R*,3*S*)-((((9H-fluoren-9-yl)methoxy)carbonyl)amino)-3-(1-methyl-H-imidazol-5-yl)-3-((triisopropylsilyl)oxy)propanoic acid (8)

Due to the presence of two unnatural stereocenters in the third building block **8**, we envisaged to create these stereocenters by enantioselective aldolisation using a titanium enolate derived from a chiral iminoglycinate (Figure S 30).[21–24] Using α-pinene as a chiral auxiliary, oxidation step followed by imine formation with protected glycine delivered compound **29**. The aldolisation step with imidazole **13** using ClTi(OiPr)$_3$ as a Lewis acid followed by acidic deprotection delivered compound **31** which was directly protected with Fmoc group to furnish compound **32** with an excellent dr ratio >99% as no other diastereoisomers were seen in the different LCMS and NMR spectra of compound **26**, **27**, **28** and building block **8**. Alcohol protection and acid deprotection delivered the building block **8** in a good overall yield (5 % for 7 steps).

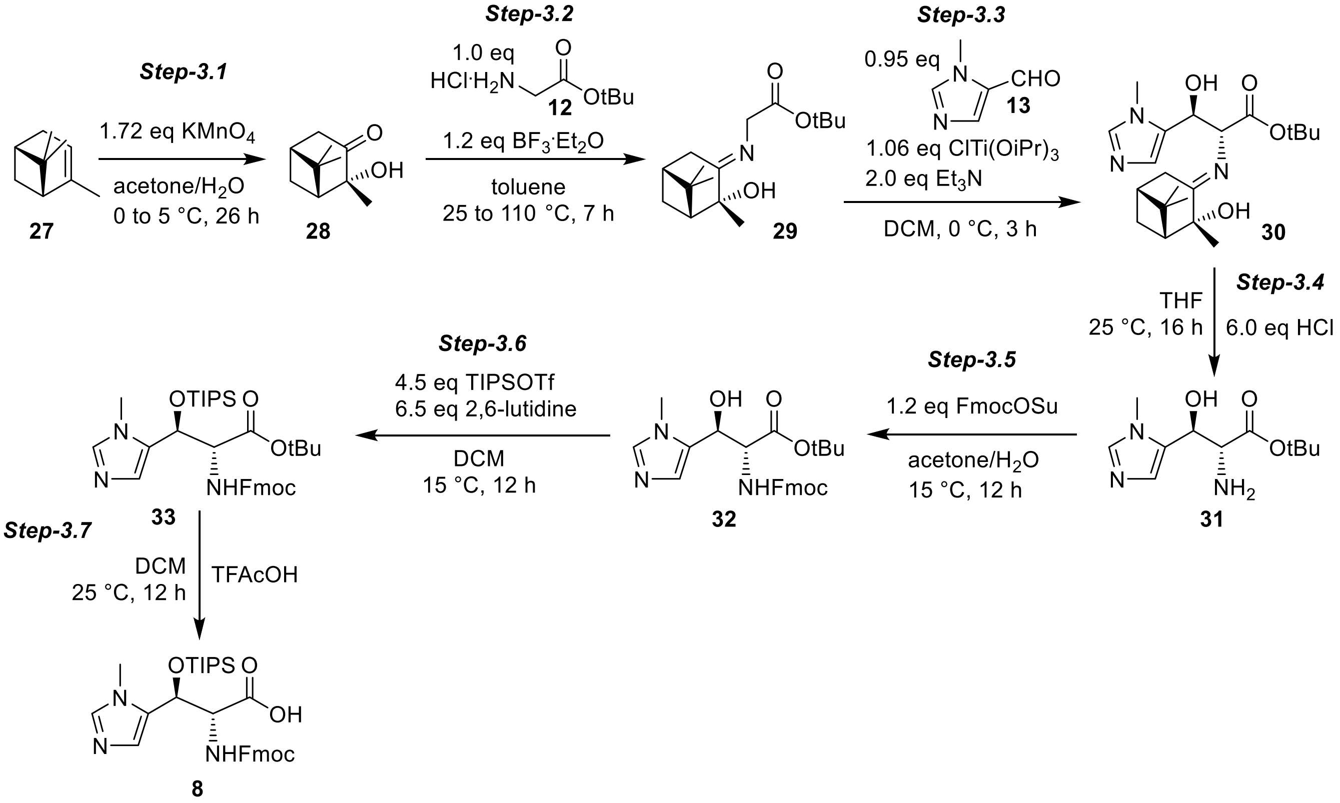

Figure S 30. Synthetic strategy towards building block 8.

10.1.4 Synthesis of compound (4R,5S)-2,2,5-trimethyl-1,3-dioxolane-4-carboxylic acid (7)

According to[25–27]

The last building block **7** was synthesized by a saponification of the commercial starting material **11** with lithium hydroxide in a mixture THF/water at room temperature for 15 h in order to deliver compound **7** with a quantitative yield.[22–24,28]

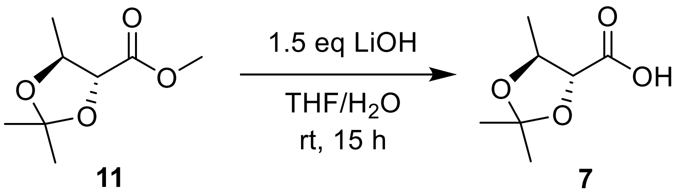

Figure S 31. Synthesis of building block 7.

10.1.5 General procedure for the synthesis of Corramycin (Corramycin, 1)

We set out to start the coupling of each amino acids and building blocks (Figure S 32) with a traditional Fmoc-SPPS in standard conditions (HATU, DIEA, DMF, room temperature) from Fmoc-L-TBDMSO-Serine. Fmoc-Leu-OH, Fmoc-L-HydroxyVal-OH **10**, Fmoc-L-TBDMSO-Ser-OH, Fmoc-L-HydroxyVal-OH **10** and Fmoc-Gly-OH. Then the building block "ribo-Phe" **9** was incorporated in lower temperature (-20 °C) in order to minimize the epimerization at the Phe position. ~10 % of the other diastereoisomer was generally observed at room temperature whereas ~2 % was present at lower temperature. The building block δ-*N*-methyl-β-hydroxy histidine **8** was then incorporated following

by the final coupling with the building block dihydroxybutyric acid **7**. Silyl protecting groups were removed with TBAF followed by cleavage of the peptide from the resin with HFIP. Final acidic deprotection allowed to deliver after reverse-phase purification the final Corramycin **1**. We were pleased to see that the NMR data (

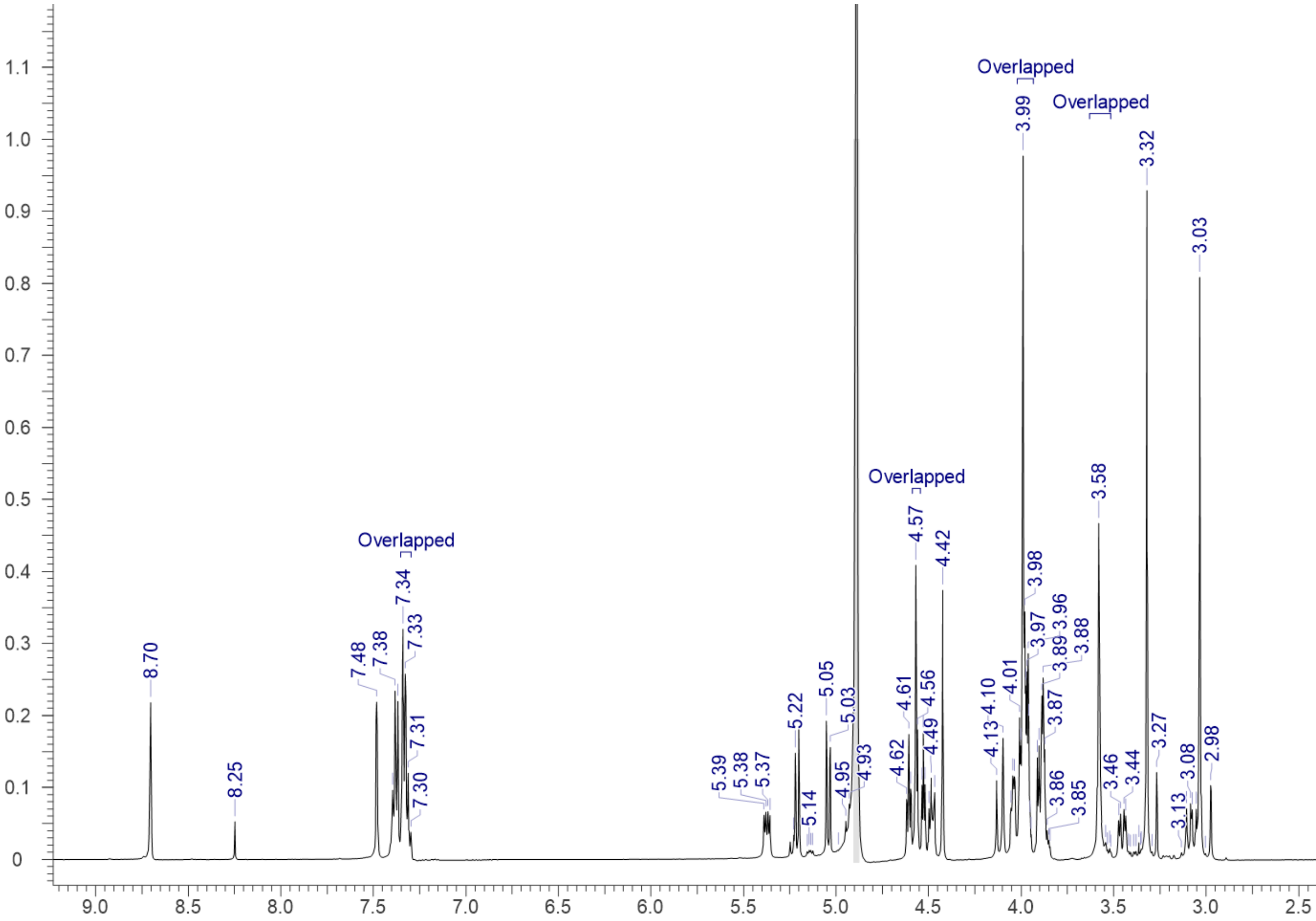

Figure **S 63** to Figure S 68) matched completely with those of the natural product.

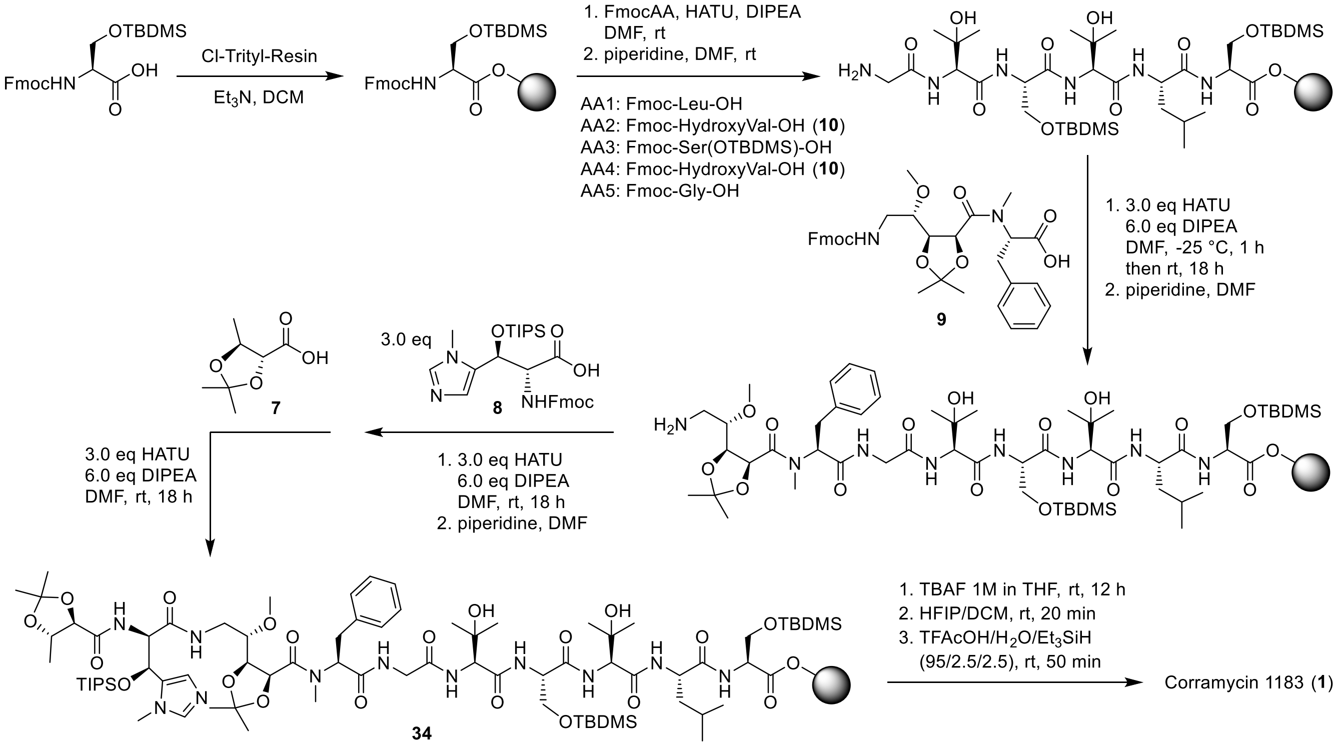

Figure S 32. Final steps *via* SPPS towards Corramycin (1).

10.2 Experimental Procedures

General:

NMR: NMR spectra were recorded on Bruker Avance 400 and 600 spectrometers. For ^{1}H NMR spectra, δ values were referenced to $CDCl_3$ (7.26 ppm), DMSO-d_6 (2.50 ppm), or D_2O (4.79 ppm). For ^{13}C NMR spectra, *δ* values were referenced to $CDCl_3$ (77.0 ppm).

LC/UV/MS: LCMS analyses were performed on HPLC or UHPLC / UV detection at 220 nm / single quadrupole MS analyzer with Electrospray ionization (ESI) source operating in positive mode. The LCMS analytical characteristics of the products are the ratio m/z of the observed pseudomolecular ion (MH+) and the retention time (R_t) of the corresponding compound, observed in UV usually at 220 nm, and expressed in minutes.

LCMS: LCMS_X-Select (FA)

Column: X-Select CSH C-18 (4.6*50 mm, 2.5u), mobile phase: A.0.1 % FA in water B. 0.1 % FA in acetonitrile, inj volume; 5.0µL, flow rate: 1.0 mL min^{-1}, gradient program: 2 % B to 98 % B in 2.8 minute, hold till 4.8 min, at 5.0 min B conc is 2 % up to 7.0 min.

Detailed experimental synthesis data:

Step 1.1. Synthesis of methyl *D*-serinate (**17**):

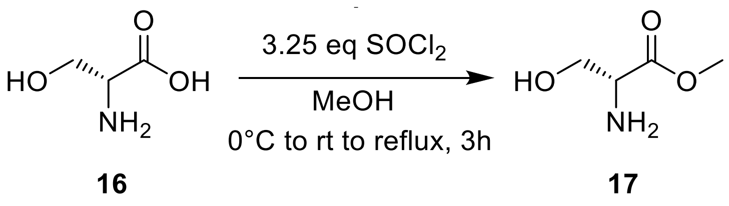

To a stirred solution of *D*-serine **16** (100 g, 952.3 mmol) in methanol at 0 °C was added thionyl chloride (224 mL, 3092.4 mmol) dropwise and stirred at the same temperature for 1 h. The reaction mixture was then heated at 70 °C for 3 h. After completion of the reaction (monitored by TLC), the reaction mixture was concentrated under reduced pressure resulting in the crude compound. The crude compound was purified by trituration with diethyl ether and *n*-hexane to afford 140 g (94 % yield) of compound **17** as a white solid.

^{1}H NMR (600 MHz, DMSO-d_6) *δ*: 8.64 (br. s, 2H), 5.30 (br. s, 1H), 4.04-4.07 (m, 1H), 3.82 (d, *J* = 3.42 Hz, 2H), 3.72 (s, 3H).

Step 1.2. Synthesis of methyl (((9H-fluoren-9-yl)methoxy)carbonyl)-*D*-serinate (**18**):

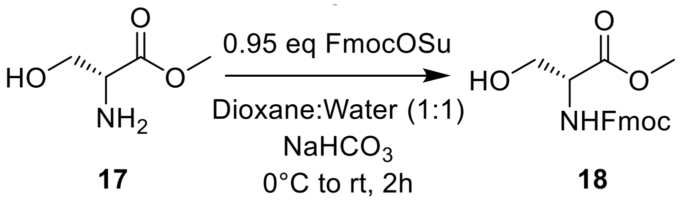

17 **18**

To a stirred solution of methyl *D*-serinate **17** (170 g, 1096.7 mmol) and sodium bicarbonate (230 g, 2740.4 mmol) in water at 0 °C was added solution of Fmoc-OSu (351 g, 1041.8 mmol) in dioxane dropwise using dropping funnel. The reaction mixture was further stirred at room temperature for 1 h. After completion of the reaction (monitored by TLC), the reaction mixture was diluted with ethyl acetate and water. The organic layer was separated and the remaining aqueous layer was re-extracted with ethyl acetate. The combined organic layer was washed with 0.2 N HCl solution (1000 mL), followed by brine solution, organic layer dried over anhydrous Na_2SO_4, filtered and concentrated under reduced pressure. The resulting crude compound was purified by trituration with diethyl ether and *n*-hexane to afford 270 g (72 % yield) of compound **18** as a white solid.

^{1}H NMR (600 MHz, DMSO-d_6) δ: 7.88 (d, *J* = 7.53 Hz, 2H), 7.72 (dd, *J* = 3.76, 7.28 Hz, 2H), 7.58 (d, *J* = 7.78 Hz, 1H), 7.37-7.43 (m, 2H), 7.29-7.34 (m, 2H), 4.95 (t, *J* = 6.02 Hz, 1H), 4.26-4.34 (m, 2H), 4.18-4.26 (m, 1H), 4.13 (td, *J* = 5.27, 7.78 Hz, 1H), 3.65 (t, *J* = 5.65 Hz, 2H), 3.62 (s, 3H).
LCMS : $[M+H]^+$ = 342.4; R_t = 1.91 min.

Step 1.3. Synthesis of (9H-fluoren-9-yl)methyl (*R*)-(1,3-dihydroxy-3-methylbutan-2-yl)carbamate (**19**):

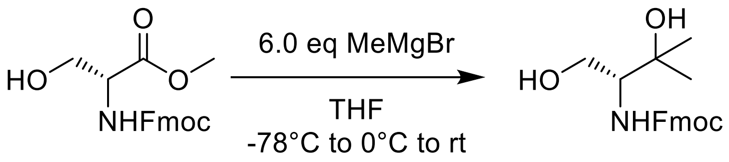

18 **19**

To solution of methyl (((9H-fluoren-9-yl)methoxy)carbonyl)-*D*-serinate **18** (100 g, 293.2 mmol) in THF at -78 °C was added 1.4 M solution of methyl magnesium bromide in THF (1256 mL, 1758.8 mmol) dropwise. The reaction mixture was allowed to attain room temperature and stirred for another 4 h. After completion of the reaction, the reaction mixture was poured over 1 N HCl solution (3 Lit) and stirred for 5 min. The resulting solution was extracted with ethyl acetate (3 x 1000 mL) and the combined organic layer was washed with water and brine and was dried over anhydrous Na_2SO_4, filtered and concentrated under reduced pressure. The resulting crude compound was purified by trituration with diethyl ether and *n*-hexane to afford 69 g (69 % yield) of compound **19** as a white solid.

^{1}H NMR (600 MHz, DMSO-d_6) δ: 7.90 (d, *J* = 7.38 Hz, 2H), 7.74 (d, *J* = 7.38 Hz, 2H), 7.39-7.45 (m, 2H), 7.30-7.37 (m, 2H), 6.88 (d, *J* = 8.86 Hz, 1H), 4.49 (t, *J* = 5.17 Hz, 1H), 4.39 (s, 1H), 4.31-4.37 (m, 1H), 4.20-4.26 (m, 2H), 3.67-3.73 (m, 1H), 3.37-3.47 (m, 2H), 1.09 (s, 3H), 1.03 (s, 3H).
LCMS: $[M+H]^+$ = 342.1; R_t = 1.92 min.

Step 1.4. Synthesis of (*S*)-2-((((9H-fluoren-9-yl)methoxy)carbonyl)amino)-3-hydroxy-3-methylbutanoic acid (**10**):

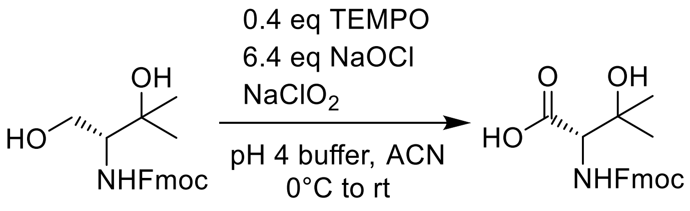

19 **10**

To a solution of (9H-fluoren-9-yl)methyl (*R*)-(1,3-dihydroxy-3-methylbutan-2-yl)carbamate **19** (100 g, 293.2 mmol) in acetonitrile and phosphate buffer solution pH = 4 at 5 °C was added TEMPO (18.2 g, 116.5 mmol) in one lot. To the resulting solution was added solution of sodium chlorite (169.7 g, 1876.3 mmol) in water (500 mL) and 10 % solution of sodium hypochlorite (300 mL) simultaneously within 30 min while maintaining the temperature between 0 °C to 5 °C. The reaction mixture was warmed to room temperature and stirred for 16 h. After completion of the reaction (monitored by TLC), the reaction mixture was diluted with ethyl acetate, water and the organic layer was separated. The separated aqueous layer was acidified with diluted HCl solution and extracted with ethyl acetate (2 x 500 mL). The combined organic layer was dried over anhydrous Na_2SO_4, filtered and concentrated under reduced pressure. The crude compound was stirred in 8 % sodium bicarbonate solution and extracted with ethyl acetate (3 x 500 mL). The aqueous layers were separated and acidify with 1 N HCl solution and re-extracted with ethyl acetate (3 x 500 mL). The combined organic layer was washed with brine, dried over anhydrous Na_2SO_4, filtered and concentrated under reduced pressure to yield 75 g of target compound **10** with 93 % HPLC purity. The compound was purified further to enhance the purity, the 75 g of target was taken in 10 % sodium sulphite solution (750 mL) and stirred for 15 min and filtered through Buchner funnel. The solid obtained was stirred in 1 N HCl solution (750 mL) and extracted with ethyl acetate (2 x 500 mL). The combined organic layer was washed with brine solution, dried over anhydrous Na_2SO_4, filtered and concentrated under reduced pressure to afford 75 g (72 % yield) of compound **10** as an off white solid.

^{1}H NMR (600 MHz, DMSO-d_6) δ: 12.49 (br. s, 1H), 7.89 (d, J = 7.34 Hz, 2H), 7.74 (d, J = 7.34 Hz, 2H), 7.37-7.44 (m, 2H), 7.24-7.35 (m, 3H), 4.70 (br. s, 1H), 4.20-4.32 (m, 3H), 3.96 (d, J = 9.29 Hz, 1H), 1.20 (s, 3H), 1.17 (s, 3H).
LCMS: $[M+Na]^+$ = 378.1; R_t = 1.89 min.

***Step-2.1*:** Synthesis of (*S*)-1-((tert-butyldimethylsilyl)oxy)-3-phenylpropan-2-amine (**20**)

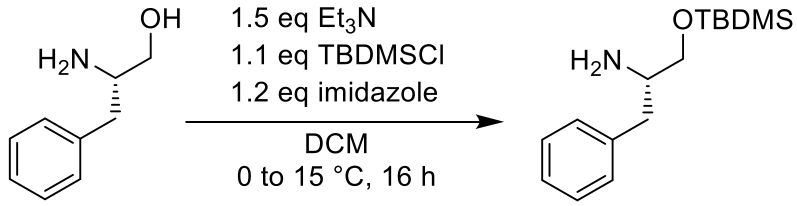

15 **20**

Two parallel reactions were carried out. To a solution of compound **15** (600 g, 3.97 mol, 1.0 *eq*) in anhydrous dichloromethane (3.00 L) was added TBDMSCl (658 g, 4.36 mol, 1.1 *eq*), Et_3N (602 g, 5.95 mol, 1.5 *eq*) and imidazole (324 g, 4.76 mol, 1.2 *eq*) at 0 °C. The mixture was stirred at 15 °C for 16 h. TLC (R_f = 0.30) showed the reaction was completed. The two reactions were combined for work up. The mixture was poured into water (3.0 L) at 0 °C and extracted with dichloromethane (1 L, 600 mL and 500 mL). The organic layer was washed with 1 N hydrochloric acid aqueous solution (600 mL x 3) and brine (600 mL x 2), dried over anhydrous sodium sulfate and concentrated *in vacuo* to give compound **20** (1.25 kg, 3.73 mol, 47 % yield) as a light yellow oil.

^{1}H NMR (600 MHz $CDCl_3$) δ 7.28-7.31 (m, 2H), 7.19-7.22 (m, 3H), 3.57 (dd, *J* = 10.8, 6.4 Hz, 1H), 3.44 (dd, *J* = 9.6, 6.8 Hz, 1H), 2.92 -3.10 (m, 1H), 2.79 (dd, *J* = 13.6, 5.2 Hz, 1H), 2.51 (dd, *J* = 13.2, 8.4 Hz, 1H), 1.45 (br.s., 2H), 0.91 (s, 9H), 0.06 (d, *J* = 1.2 Hz, 6H).

^{13}C NMR (125 MHz $CDCl_3$) δ = 139.21, 129.27, 128.43, 126.22, 77.44, 77.12, 76.81, 67.49, 54.37, 40.50, 25.95, 18.30, - 5.34.

LCMS: $[M+H]^+$= 265.9 ; R_t = 1.308 min.

HRMS (ESI/Q-TOF) m/z: $[M+H]^+$ calculated for $C_{15}H_{28}NOSi$ = 266.1934,; found= 266.1953.

Step-2.2: Synthesis of ((3a*S*,4*S*,6a*S*)-2,2-dimethyl-6-oxotetrahydrofuro[3,4-d][1,3]dioxol-4-yl)methyl 4-methylbenzenesulfonate (**21**)

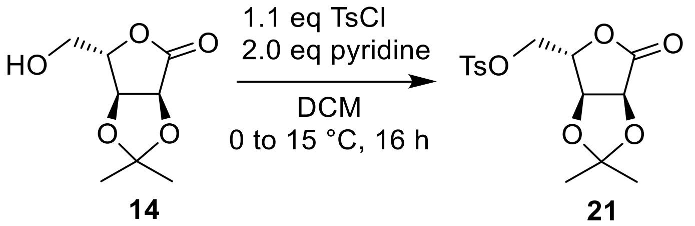

Two parallel reactions were carried out. To a solution of compound **14** (500 g, 2.66 mol, 1.0 *eq*) in dichloromethane (2.50 L) was added pyridine (420 g, 5.31 mol, 2.0 *eq*) and 4-methylbenzenesulfonyl chloride (557 g, 2.92 mol, 1.1 *eq*) at 0 °C. The mixture was stirred at 15 °C for 16 h. TLC (petroleum ether:ethyl acetate = 3:1, R_f = 0.30) showed the reaction was completed. The two reactions were combined for work up. The mixture was poured into water (3.0 L) at 0 °C, and the organic phase was washed with 0.5 N hydrochloric acid aqueous solution (3.0 L, 3.0 L, 2.0 L, 2.0 L). The organic phase was washed with brine (2.0 L, 1.5 L), dried over anhydrous sodium sulfate, filtered and concentrated *in vacuo*. The crude product was triturated with petroleum ether (7.5 L) at 15 °C and stirred for 12 h. The suspension was filtered and the filter cake was dried under vacuum to give compound **21** (1.5 kg, 4.36 mol, 82 % yield) as a white solid.

^{1}H NMR: (600 MHz $CDCl_3$) δ 7.75 (d, *J* = 8.4 Hz, 2H), 7.38 (d, *J* = 8.0 Hz, 2H), 4.74-4.79 (m, 2H), 4.69 (s, 1H), 4.34 (dd, *J* = 11.2, 1.6 Hz, 1H), 4.18 (dd, *J* = 11.2, 2.4 Hz, 1H), 2.46 (s, 3H), 1.45 (s, 3H), 1.38 (s, 3H).

^{13}C NMR (125 MHz $CDCl_3$) δ = 173.07, 145.88, 131.70, 130.23, 127.92, 113.82, 79.05, 77.35, 77.05, 76.73, 74.49, 68.28, 26.64, 25.48, 21.69.

LCMS:$[M+H]^+$= 360.1 ; R_t = 1.308 min.

HRMS (ESI/Q-TOF) m/z: $[M+NH_4]^+$ calculated for $C_{15}H_{22}NO_7S$ = 360.1111, found = 360.1112.

***Step-2.3*:** Synthesis of (3a*S*,6*S*,6a*S*)-6-(azidomethyl)-2,2-dimethyldihydrofuro[3,4-d]-[1,3]dioxol-4(3aH)-one (**22**)

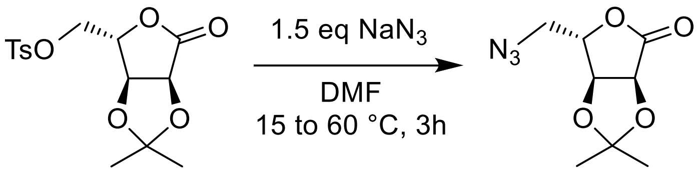

21 **22**

Two parallel reactions were carried out. A solution of compound **21** (493 g, 1.44 mol, 1.0 *eq*) in *N,N*-dimethylformamide (2.45 L) was degassed and purged with nitrogen for three times, then sodium azide (140 g, 2.16 mol, 1.5 *eq*) was added at 15 °C slowly. The mixture was stirred at 60 °C for 3 h (inner temperature: 50 °C). TLC (petroleum ether:ethyl acetate = 3:1, R_f = 0.5) showed the reaction was completed. Three reactions were combined for work up. The mixture was poured into water (15 L) and ethyl acetate (5.0 L) at 0 °C. The aqueous phase was extracted with ethyl acetate (5.0 L x 3). The combined organic phase was washed with brine (3.0 L x 2), dried over anhydrous sodium sulfate, filtered and concentrated *in vacuo* to give compound **22** (800 g, 87 % purity) as a brown oil.

^{1}H NMR: (600 MHz $CDCl_3$) *δ* 4.80 (d, *J* = 5.6 Hz, 1H), 4.63 (t, *J* = 2.9 Hz, 1H), 4.61 (d, *J* = 6.0 Hz, 1H), 3.75 (dd, *J* = 13.2, 3.2 Hz, 1H), 3.64 (dd, *J* = 13.2, 2.4 Hz, 1H), .1.42 (s, 3H), 1.33 (s, 3H).

^{13}C NMR (125 MHz $CDCl_3$) *δ* = 173.46, 113.55, 80.09, 78.01, 77.47, 77.15, 76.83, 75.06, 52.44, 26.59, 23.40.

LCMS: $[M+H]^+$= 231.1 ; R_t = 1.727 min.

HRMS (ESI/Q-TOF) m/z: $[M+NH_4]^+$ calculated for $C_8H_{15}N_4O_4$ = 231.1088 found = 231.1084.

***Step-2.4*:** Synthesis of (4*S*,5*S*)-5-((*S*)-2-azido-1-hydroxyethyl)-*N*-((S)-1-((tert-butyldimethylsilyl)oxy)-3-phenylpropan-2-yl)-2,2-dimethyl-1,3-dioxolane-4-carboxamide (**23**)

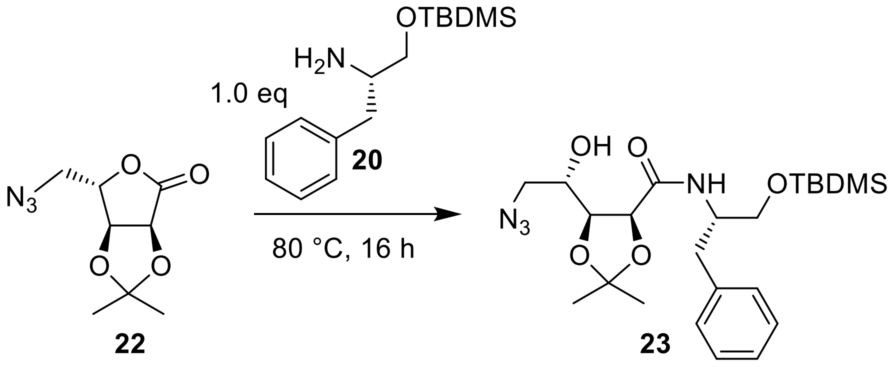

Two parallel reactions were carried out. Compound **22** (360 g, 1.48 mol, 1.0 *eq*) was dissolved in compound (*S*)-1-((tert-butyldimethylsilyl)oxy)-3-phenylpropan-2-amine **20** (495 g, 1.48 mol, 1.0 *eq*) in a 3 L three-necked round bottom flask. The mixture was stirred at 80 °C for 16 h under nitrogen. TLC (petroleum ether:ethyl acetate = 5:1, R_f = 0.60) showed the reaction was completed. Two reactions were combined for work up. The mixture was purified by column chromatography (SiO_2, petroleum ether**/**ethyl acetate **=** 20**/**1 to 5**/**1) to give compound **23** (900 g, 57 % yield) as a brown oil.

^{1}H NMR: (600 MHz $CDCl_3$) δ 7.27-7.30 (m, 2H), 7.21-7.25 (m, 4H), 4.88 (d, *J* = 3.6 Hz, 1H), 4.56 (d, *J* = 7.6 Hz, 1H), 4.29-4.39 (m, 2H), 3.59-3.66 (m, 2H), 3.35 (dd, *J* = 12.8 Hz, 3.2 Hz, 1H), 3.26 (d, *J* = 12.8 Hz, 3.2 Hz, 1H), 2.91-3.00 (m, 2H), 2.80-2.85 (m, 1H), 1.50 (s, 3H), 1.36 (s, 3H), 0.95 (s, 9H), 0.08 (d, *J* = 2.8 Hz, 6H).

^{13}C NMR (125 MHz $CDCl_3$) δ = 170.48, 137.61, 129.46, 128.47, 126.62, 110.34, 77.85, 77.36, 77.04, 76.82, 76.72, 69.76, 63.33, 53.45, 51.19, 37.78, 26.91, 25.85, 24.48, 18.22, -5.49.

HRMS (ESI/Q-TOF) m/z: $[M+H]^+$ calculated for $C_{23}H_{39}N_4O_5Si$ = 479.2684, found, 479.2689.

***Step-2.5*:** Synthesis of (4*S*,5*S*)-5-((*S*)-2-azido-1-methoxyethyl)-*N*-((*S*)-1-((tert-butyldimethylsilyl)oxy)-3-phenylpropan-2-yl)-*N*,2,2-trimethyl-1,3-dioxolane-4-carboxamide (**24**)

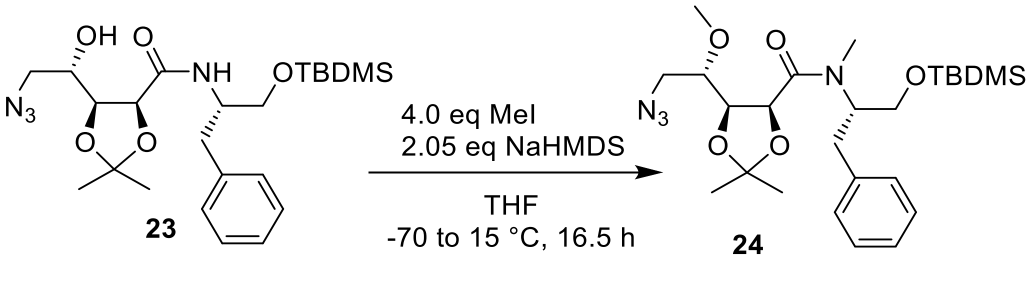

Two parallel reactions were carried out. A solution of compound **23** (400 g, 836 mmol, 1.0 *eq*) in tetrahydrofuran (1.5 L) was degassed and purged with nitrogen for three times, then NaHMDS (1 M, 1.71 L, 2.05 *eq*) was added drop-wise at -70 °C. After addition, the mixture was stirred at this temperature for 30 min and then MeI (474 g, 3.34 mol, 4.0 *eq*) was added drop-wise at -70 °C. The resulting mixture was warmed to 15 °C and stirred at 15 °C for 16 h. TLC (petroleum ether:ethyl acetate = 3:1, R_f = 0.30) (PMA) showed the reaction was completed. Two reactions were combined to work up. The mixture was poured into saturated ammonium chloride aqueous solution (6.0 L) at 0 °C. The mixture was extracted with ethyl acetate (2.0 L, 1.5 L x 3). The combined organic phase was washed with brine (1.0 L x 2),

dried over anhydrous sodium sulfate, filtered and concentrated *in vacuo*. The residue was added petroleum ether (4.0 L) and stirred for 12 h. The suspension was filtered and the filter cake was dried under vacuum to give compound **24** (800 g, 1.58 mol, 94 % yield) as a white solid.

^{1}H NMR: (600 MHz $CDCl_3$) δ 7.20-7.30 (m, 5H), 5.11 (d, J = 5.6 Hz, 1H), 4.79-4.84 (m, 1H), 4.35-4.39 (m, 1H), 3.73-3.77 (m, 2H), 3.58-3.65 (m, 2H), 3.32-3.33 (m, 1H), 3.33 (s, 3H), 2.98 (s, 3H), 2.68-2.87 (m, 1H), 2.66-2.68 (m, 1H), 1.29-1.33 (m, 3H), 0.80-0.88 (s, 9H), 0.04 (s, 6H).

^{13}C NMR (125 MHz $CDCl_3$) δ = 173.20, 172.82, 144.00, 143.85, 134.92, 134.68, 134.46, 134.07, 132.43, 131.98, 115.45, 115.15, 83.15, 83.02, 82.71, 82.39, 80.94, 79.95, 79.56, 68.72, 66.81, 65.65, 62.07, 61.94, 54.59, 54.37, 41.66, 39.98, 36.47, 33.58, 33.45, 33.12, 31.46, 31.40, 31.28, 23.81, 23.77, 0.10.

LCMS: $[M+H]^+$= 507.3 ; R_t = 1.727 min.

HRMS (ESI/Q-TOF) m/z: $[M+H]^+$ calculated for $C_{25}H_{43}N_4O_5Si$ = 507.2997, found, 507.2990.

Step-2.6: Synthesis of (4*S*,5*S*)-5-((*S*)-2-azido-1-methoxyethyl)-*N*-((*S*)-1-hydroxy-3-phenylpropan-2-yl)-*N*,2,2-trimethyl-1,3-dioxolane-4-carboxamide (**25**)

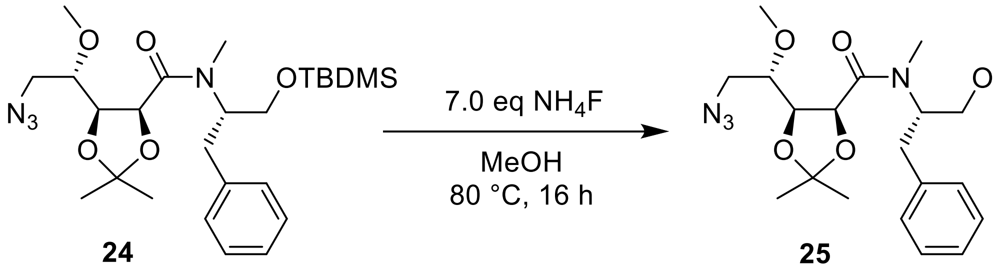

Two parallel reactions were carried out. To a solution of compound **24** (400 g, 789 mmol, 1.0 *eq*) in methanol (2.00 L) was added ammonium fluoride (205 g, 5.53 mol, 7.0 *eq*). The mixture was stirred at 80 °C to reflux for 16 h. TLC (dichloromethane:methanol = 20:1, R_f = 0.30) showed the reaction was completed. Two reactions were combined to work up. The reaction mixture was cooled to 15 °C to give a suspension. The suspension was filtered and the filtrate was concentrated *in vacuo.* The residue was added water (3.0 L) and ethyl acetate (5.0 L), and stirred for 30 min. The aqueous phase was extracted with ethyl acetate (2.0 L, 1.0 L) in turn. The combined organic phase was washed with brine (1.0 L x 2), dried over anhydrous sodium sulfate, filtered and concentrated *in vacuo* to give compound **25** (500 g, 1.27 mol, 80 % yield, 99 % purity) as a light yellow solid.

^{1}H NMR: (600 MHz $CDCl_3$) δ 7.21-7.33 (m, 5H), 4.85-5.08 (m, 1H), 4.36-4.71 (m, 2H), 3.62-3.77 (m, 5H), 3.36-3.42 (m, 1H), 3.26- 3.32 (m, 3H), 2.92-3.02 (m, 3H), 2.80 (m, 1H), 2.69-2.73 (m, 1H), 1.48-1.53 (m, 3H) 1.32-1.37 (m, 3H).

^{13}C NMR (125 MHz $CDCl_3$) δ = 167.06, 166.01, 136.12, 135.57, 127.31, 127.03, 126.95, 126.72, 124.96, 124.68, 108.09, 107.96, 75.56, 75.25, 74.93, 73.57, 73.41, 72.24, 72.02, 60.14, 56.48, 54.62, 54.36, 47.08, 46.91, 34.18, 32.78, 28.81, 25.84, 25.45, 23.86, 23.71.

LCMS: $[M+H]^+$= 393.2 ; R_t = 2.800 min.

HRMS (ESI/Q-TOF) m/z: $[M+H]^+$ calculated for $C_{19}H_{29}N_4O_5$ = 393.2132, found, 393.2110.

***Step-2.7*:** Synthesis of (*S*)-2-((4*S*,5*S*)-5-((*S*)-2-azido-1-methoxyethyl)-*N*,2,2-trimethyl-1,3-dioxolane-4-carboxamido)-3-phenylpropanoic acid (**26**)

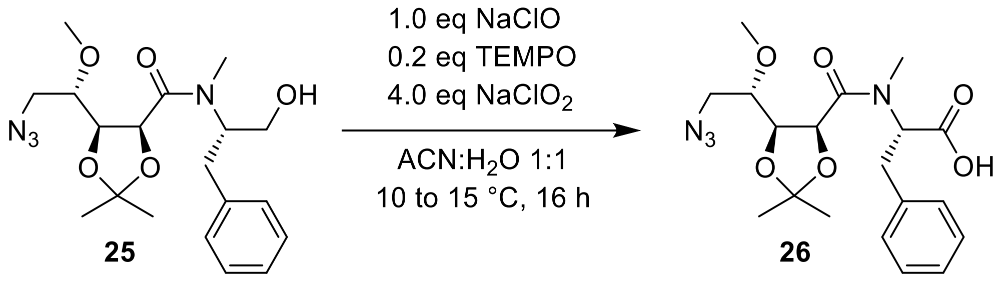

Two parallel reactions were carried out. To a solution of compound **25** (170 g, 433 mmol, 1.0 eq) in acetonitrile (425 mL) was added TEMPO (13.6 g, 86.6 mmol, 0.20 *eq*) and dropwsie a solution of $NaClO_2$ (157 g, 1.73 mol, 4.0 eq) in water (425 mL). The mixture was cooled to 10 °C and NaClO (222 g, 433 mmol, 14.5 % purity, 1.0 eq) was added drop-wise at 10-15 °C. The mixture was stirred at 15 °C for 16 h. TLC (dichloromethane:methanol = 10:1, R_f = 0.2) showed the reaction was completed. Every reaction mixture was added sodium thiosulfate (85 g) and ice (100 g) at 0-10 °C in three portions, and then two reactions were combined to work up. The mixture was concentrated *in vacuo* to remove acetonitrile. The mixture was extracted with ethyl acetate (300 mL x 5), and the combined organic phase was washed with saturated citric acid aqueous solution (300 mL) and brine (300 mL), dried over anhydrous sodium sulfate, filtered and concentrated *in vacuo.* The residue was added ethyl acetate (350 mL) and stirred for 10 h. The suspension was filtered and the filter cake was dried under vacuum to give compound **26** (320 g, 748 mmol, 86 % yield, 95 % purity) as a white solid.

^{1}H NMR: (600 MHz $CDCl_3$) δ 7.15-7.25 (m, 5H), 5.44 (dd, *J* = 8.8, 6.4 Hz, 1H), 4.70 (d, *J* = 6.0 Hz, 1H), 4.26-4.38 (m, 1H), 3.59-3.65 (m, 2H), 3.29-3.30 (m, 2H), 3.19 (s, 3H), 2.95-2.96 (m, 1H), 2.91 (s, 3H), 1.38 (s, 3H), 1.26 (s, 3H).

^{13}C NMR (125 MHz $CDCl_3$) δ = 173.31, 168.88, 168.86, 136.52, 129.29, 128.80, 128.766, 128.59, 127.08, 126.82, 110.22, 109.98, 77.36, 77.30, 77.04, 76.72, 75.62, 73.95, 73.61, 67.89, 60.85, 57.07, 56.44, 49.08, 35.50, 34.91, 31.38, 27.53, 27.13, 25.58, 25.55

LCMS: $[M+H]^+$= 407.2 ; R_t = 2.835 min.

HRMS (ESI/Q-TOF) m/z: $[M+H]^+$ calculated for $C_{19}H_{27}N_4O_6$ = 407.1925, found = 407.1923

Step-2.8: Synthesis of (*S*)-2-((4*S*,5*S*)-5-((*S*)-2-((((9H-fluoren-9-yl)methoxy)carbonyl)amino)-1-methoxyethyl)-*N*,2,2-trimethyl-1,3-dioxolane-4-carboxamido)-3-phenylpropanoic acid (**9**)

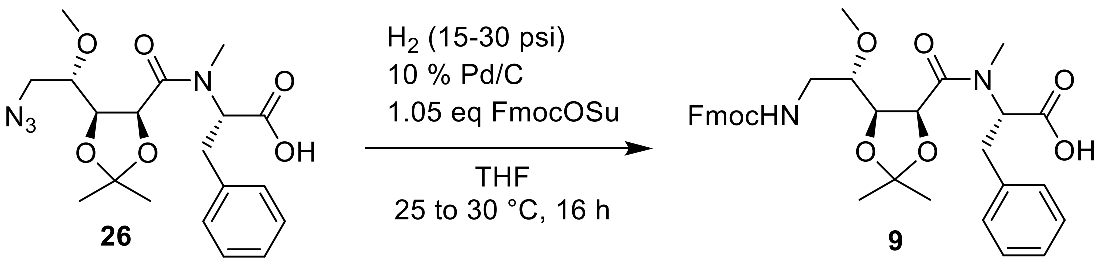

Three parallel reactions were carried out. To a solution of compound **26** (100 g, 185 mmol, 1.0 eq) in tetrahydrofuran (500 mL) was added 10 % Pd/C (15.0 g, 50 % purity) and FMOC-OSu (65.4 g, 194 mmol, 1.05 eq) under nitrogen. The suspension was degassed under vacuum and purged with hydrogen several times. The mixture was stirred under hydrogen (15 psi) at 25 °C for 10 h. The mixture was heated to 30 °C and stirred for 6 h under hydrogen (30 psi). TLC (dichloromethane:methanol = 10:1, R_f = 0.6) showed the reaction was completed. Three reactions were combined for work up. The mixture was filtered and the filtrate was concentrated *in vacuo.* The mixture was added methyl tertiary-butyl ether (1.5 L) and water (3.0 L) and stirred for 30 min. The pH value was adjusted to around 8 by progressively adding solid sodium bicarbonate. The mixture was filtered and the filtrate of the aqueous phase was extracted with methyl tertiary-butyl ether (1.0 L x 3). The pH value of the aqueous phase as adjusted to around 3 by progressively adding solid citric acid. The mixture was extracted with ethyl acetate (1.0 L x 5) in turn. The combined organic phase was washed with brine (500 mL x 3), dried over anhydrous sodium sulfate, filtered and concentrated i*n vacuo* to give the crude product of compound **9** (280 g, 95 % purity, 45 % yield) as a white solid.

^{1}H NMR: (600 MHz DMSO-d6) δ 12.85 (br.s., 1H), 7.89 (d, *J* = 7.6 Hz, 2H), 7.68-7.77 (m, 2H), 7.34-7.41 (m, 2H), 7.28-7.34 (m, 3H), 7.19- 7.24 (m, 4H), 5.21-5.25 (m, 1H), 4.82-4.84 (m, 1H), 4.19-4.27 (m, 4H), 3.35-3.37 (m, 1H), 3.21-3.23 (m, 2H), 3.18-3.19 (m, 1H), 2.96 (s, 3H), 2.82 (s, 3H), 2.50 (s, 1H), 1.35 (s, 3H), 1.26 (s, 3H).

^{13}C NMR (125 MHz $CDCl_3$) δ = 172.41, 171.93, 168.37, 167.36, 156.78, 156.73, 144.38, 144.32, 141.14, 138.08, 129.75, 129.17, 128.71, 128.04, 127.48, 126.81, 125.75, 125.72, 120.52, 109.33, 77.04, 76.39, 73.58, 65.91, 57.53, 56.74, 55.36, 47.16, 40.54, 40.34, 40.13, 39.92, 39.71, 39.50, 39.29, 34.83, 31.46, 27.88, 27.49, 26.19.

LCMS: $[M+H]^+$ = 603.2 ; R_t = 1.761 min).

HRMS (ESI/Q-TOF) m/z: $[M+H]^+$ calculated for $C_{34}H_{39}N_2O_8$ = 603.2701, found = 603.2698

Step-3.1: Synthesis of (1*R*,2*R*,5*R*)-2-hydroxy-2,6,6-trimethyl-norpinan-3-one (**28**)

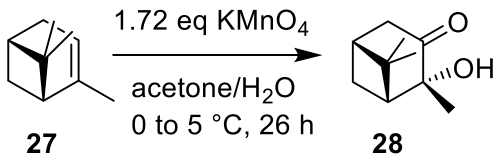

Four parallel reactions were carried out. To a cold (0 to 5 °C) solution of (1*S*,5*S*)-4,6,6-trimethylbicyclo[3.1.1]hept-3-ene **27** (500 g, 3.67 mol) in acetone (5.00 L) and H_2O (400 mL) was added $KMnO_4$ (998 g, 6.31 mol) with stirring over 10 h. The reaction mixture was stirred at 0 - 5 °C for an additional 16 h. TLC (petroleum ether/EtOAc = 5/1, R_f = 0.5) showed the reaction was completed. Four reactions were worked up together. The mixture was filtered and the solid cake was washed with TBME (2 L x 4). The combined solution was evaporated to about 600 mL. The mixture was diluted with TBME (2 L) and washed with water (800 mL), saturated aqueous sodium bicarbonate (600 mL) and dried over Na_2SO_4. The solvent was removed under reduced pressure to obtain an oil. The crude product was distilled in vacuum (80 °C, oil pump), and recrystallized using pentane (4 L) for 6 times to give compound **28** (530 g, 3.15 mol, 21 % yield, 99 % purity) as a white solid.

^{1}H NMR: (600 MHz $CDCl_3$): δ ppm 2.62 - 2.61 (m, 2H), 2.47 - 2.44 (m, 1H), 2.13 – 2.10 (m, 2H), 1.69 (d, *J* = 10.8 Hz, 1H), 1;39 (s, 3H), 1.37 (s, 3H), 0.89 (s, 3H).

^{13}C NMR (125 MHz $CDCl_3$) δ = 214.03, 49.66, 42.92, 39.24, 38.29, 28.38, 22.27, 25.22, 22.82.

LCMS: $[M+H]^+$ = 169.1 ; R_t = 2.067 min.

HRMS (ESI/Q-TOF) m/z: $[M+NH_4]^+$ calculated for $C_{10}H_{20}NO_2$ = 186.1488, found = 186.1477

Step-3.2: Synthesis of [(1*R*,2*R*,5*R*)-2-Hydroxy-2,6,6-trimethyl-bicyclo[3.1.1]hept-(3E)-ylideneamino]-acetic acid tert-butyl ester (**29**)

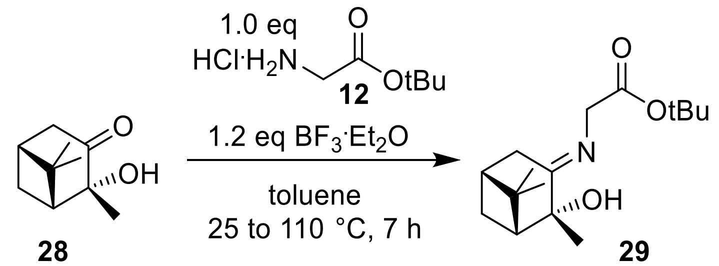

Two parallel reactions were carried out. Triethylamine (231 g, 2.28 mol) was added to the solution of glycine *tert*-butyl ester hydrochloride (**12**, 316 g, 1.88 mol) in toluene (4 L) at 25 °C, then the solution was stirred at 30 °C for 2 h and the solid was filtered. Compound **28** (320 g, 1.90 mol) was added to the filtrate, and the mixture was heated to 60 °C, then $BF_3{\cdot}Et_2O$ (54.0 g, 380 mmol) was added and the solution was stirred in a Dean-Stark apparatus at 110 °C for 5 h. TLC (petroleum ether/EtOAc = 3/1, Product: R_f = 0.8) showed the reaction was complete. Two reactions were worked up together. The solvent was evaporated to give compound **29** (1.75 kg, crude) as a yellow oil.

^{1}H NMR: (600 MHz $CDCl_3$): δ ppm 4.07 (m, 1H), 2.47 (m, 1H), 2.34 (m, 1H), 2.06 (m, 1H), 1.56 (m, 1H), 1.50 (s, 3H), 1.47 (s, 9H), 1.38 (m, 1H), 1.32 (s, 3H), 0.86 (s, 3H).

^{13}C NMR (125 MHz $CDCl_3$) δ = 179.45, 169.33, 81.17, 76.43, 53.31, 50.400, 42.95, 38.50, 38.24, 33.59, 28.17, 28.06, 27.27, 25.07, 22.83.

LCMS: $[M+H]^+$ = 266.1 ; R_t = 1.420 min.

HRMS (ESI/Q-TOF) m/z: $[M+H]^+$ calculated for $C_{16}H_{28}NO_3$ = 282.2063, found = 282.2041.

Step-3.3: Synthesis of (2*R*,3*S*)-3-Hydroxy-2-[(1*R*,2*R*,5*R*)-2-hydroxy-2,6,6-trimethyl-bicyclo[3.1.1]hept-(3E)-ylideneamino]-3-(3-methyl-3H-imidazol-4-yl)-propionic acid tert-butyl ester (**30**)

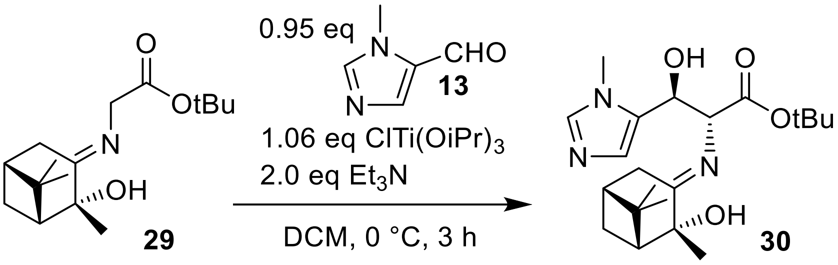

Three parallel reactions were carried out. To a solution of compound **29** (350 g, 1.24 mol) in DCM (1.4 L) added a solution of chloro(triisopropoxy)titanium (340 g, 1.31 mol) in DCM (500 mL) a solution of compound **13** (123 g, 1.12 mol) in DCM (500 mL) and anhydrous Et_3N (252 g, 2.49 mol) dropwise at 0 °C. Then the solution was stirred at 0 °C for 3 h. TLC (DCM/MeOH = 10/1, R_f = 0.3) showed the reaction was completed. Three parallel reactions were worked up together. The mixture was poured into a cold saturated solution of NaCl (1.4 L). The mixture was filtered. The aqueous solution was extracted with DCM (4 L) for two times. The combined organic phase was dried over Na_2SO_4 and the solvent was evaporated under vacuum to give compound **30** (1.63 kg, crude) as a yellow solid.

^{1}H NMR: (600 MHz $CDCl_3$): δ ppm 7.21 (s, 1H), 7.10 (s, 1H), 5.09 (d, J = 6 Hz, 1H), 4.47 (d, J = 5.6 Hz, 1H), 3.61 (s, 3H), 2.98 (m, 1H), 2.57 (d, J = 3.2 Hz, 1H), 2.43 (d, J = 3.2 Hz, 1H), 2.27 (m, 1H), 1.99 (m, 1H), 1.49 (s, 3H), 1.44 (m, 1H), 1.34 (m, 1H), 1.29 (s, 9H), 1.16 (d, J = 6 Hz, 3H), 0.88 (s, 3H).

^{13}C NMR (125 MHz $CDCl_3$) δ = 180.93, 168.38, 137.72, 131.41, 128.10, 82.10, 76.61, 67.04, 65.77, 50.51, 45.88, 42.97, 38.62, 38.41, 33.87, 31.86, 28.17, 28.07, 27.83, 27.26, 25.27, 23.04, 8.87.

LCMS: $[M+H]^+$ = 376.2 ; R_t = 1.300 min.

HRMS (ESI/Q-TOF) m/z: $[M+NH_4]^+$ calculated for $C_{20}H_{32}N_4O_4$ = 392.2412, found = 392.2554

Step-3.4: Synthesis of (2*R*,3*S*)-2-(9H-Fluoren-9-ylmethoxycarbonylamino)-3-hydroxy-3-(3-methyl-3H-imidazol-4-yl)-propionic acid tert-butyl ester (31)

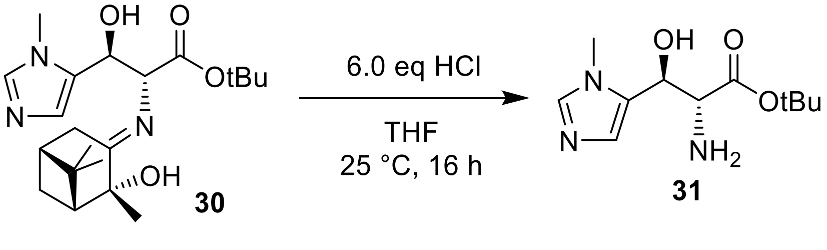

Five parallel reactions were carried out. Compound **30** (320 g, 817 mmol) was dissolved in HCl (1.2 M, 4.1 L) and THF (2.2 L). The resulting mixture was stirred at 25 °C for 16 h. TLC (dichloromethane/methanol = 10/1, R_f = 0.1) showed the reaction was complete. Five parallel reactions were combined. The reaction mixture was washed with EtOAc (1.50 L) for three times. The aqueous phase was basified with $NaHCO_3$ to pH = 7 – 8 to give compound **31** (986 g, crude) in a yellow water solution (4.00 L) and was directly used for the next step.

Step-3.5: Synthesis of tert-butyl (2*R*,3*S*)-2-(9H-fluoren-9-ylmethoxycarbonylamino)-3-(3-methylimidazol-4-yl)-3-triisopropylsilyloxy-propanoate (**32**)

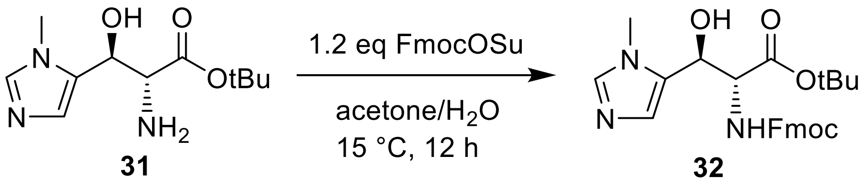

31 **32**

Five parallel reactions were carried out. Acetone (800 mL) and H_2O (500 mL) was added followed by Fmoc-O-Su (329 g, 975 mmol) to the compound **31** (196 g, 812 mmol). The resulting mixture was stirred at 15 °C for 12 h. LCMS showed the reaction was complete. Five parallel reactions were worked up together. The reaction mixture was extracted with EtOAc (3.5 L x3). The combined organic phase was washed with brine (2 L), dried over Na_2SO_4, concentrated to give compound **32** (2.00 kg, crude) as a yellow oil. The crude product was purified by column chromatography (SiO_2, Dichloromethane/Methanol = 50/1 to 10/1) to give 580 g of compound **32**.

^{1}H NMR: (600 MHz $CDCl_3$): δ ppm 7.76 (d, J = 7.6 Hz, 2H), 7.56 (d, J = 7.2 Hz, 2H), 7.38 (m, 3H), 7.29 (m, 2H), 6.95 (s, 1H), 5.85 (d, J = 7.2 Hz, 1H), 5.30 (s, 1H), 5.10 (d, J= 4.4 Hz, 1H), 4.65 (d, J = 6 Hz, 1H), 4.38 (m, 2H), 4.19 (m, 1H), 3.69 (s, 3H), 1.41 (s, 9H).

^{13}C NMR (125 MHz $CDCl_3$) δ = 173.23, 168.77, 156.45, 143.65, 141.28, 138.80, 127.78, 127.12, 126.94, 125.06, 120.01, 83.38, 67.33, 67.04, 58.24, 53.47, 47.04, 32.49, 27.90, 25.43

LCMS: $[M+H]^+$ = 464.2 ; R_t = 1.180 min.

HRMS (ESI/Q-TOF) m/z: $[M+H]^+$ calculated for $C_{26}H_{30}N_3O_5$ = 464.2180, found = 464.2219

Step-3.6: Synthesis of (2*R*,3*S*)-tert-butyl 2-((((9H-fluoren-9-yl)methoxy)carbonyl)amino)-3-(1-methyl-H-imidazol-5-yl)-3-((triisopropylsilyl)oxy)propanoate (**33**)

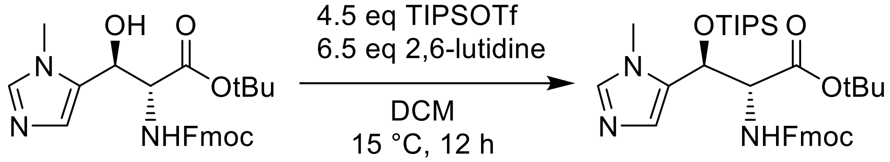

32 **33**

Three parallel reactions were carried out. To a solution of compound **32** (192 g, 414 mmol) in DCM (1. L) was added 2,6-Lutidine (288 g, 2.69 mol) and TIPSOTf (571 g, 1.86 mol) drop wise at 0-5 °C, then the reaction was stirred at 15 °C for 12 h. LCMS (starting material: R_t = 1.15 min, product: RT = 1.37 min) showed the starting material was consumed completely. The three reactions were combined for work-up. Saturated NH_4Cl aqueous solution (2 L) was added and then separated and the aqueous was extracted with DCM (1 L x2), the combined organic phase was washed with saturated citric acid solution (1.5 L x2), brine(1 L), dried over Na_2SO_4, filtered and concentrated under reduced pressure to afford compound **33** (771 g, 1.24 mol) as a yellow oil.

^{1}H NMR: (600 MHz $CDCl_3$): δ ppm 7.77 (m, 2H), 7.57 (m, 2H), 7.39 (m, 3H), 7.32 (m, 2H), 9.67 (s, 1H), 5.57 (d, J = 8.4 hz, 1H), 5.32 (d, J = 3.2 Hz, 1H), 4.53 (m, 1H), 4.40 (m, 2H), 4.19 (m, 1H), 3.73 (s, 3H), 1.43 (s, 9H), 1.02 (m, 21H)

^{13}C NMR (125 MHz $CDCl_3$) δ = 167.95, 157.64, 155.79, 143.79, 143.69, 141.33, 138.91, 136.52, 130.19, 129.19, 127.75, 127.07, 125.01, 124.97, 120.14, 120.02, 82.99, 69.52, 67.05, 60.10, 47.09, 32.53, 27.99, 24.49, 17.86, 12.36.

LCMS: $[M+H]^+$ = 620.3 ; R_t = 1.420 min.

HRMS (ESI/Q-TOF) m/z: $[M+H]^+$ calculated for $C_{35}H_{50}N_3O_5Si$ = 620.3514, found = 620.3540.

Step-3.7: Synthesis of (2*R*,3*S*)-((((9H-fluoren-9-yl)methoxy)carbonyl)amino)-3-(1-methyl-H-imidazol-5-yl)-3-((triisopropylsilyl)oxy)propanoic acid (**8**)

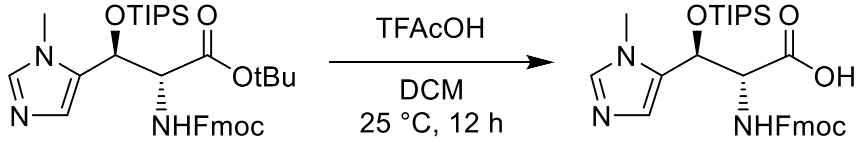

33 **8**

Three parallel reactions were carried out. To a solution of compound **33** (257 g, 414 mmol) in DCM (1 L) was added TFAcOH (1.01 kg, 8.82 mol) at 0 °C, then the mixture was stirred at 25 °C for 12 h. LCMS (product: Rt = 1.27 min) showed the starting material was consumed completely. Three reactions were combined for work-up. The reaction was concentrated under reduced pressure to give a residue. The residue was dissolved with DCM (1.5 L), then the organic phase was washed with water (1 L x 2), adjust to pH = 7 with saturated $NaHCO_3$, filtered and the filter cake was washed with MTBE (1 L) to give a crude product. The crude product was triturated with MeOH (500 mL x2) to afford compound **8** (255 g, 437 mmol, 35 % yield) as a white solid.

^{1}H NMR: (600 MHz $CDCl_3$): δ ppm 7.86 (d, *J* = 7.6 Hz, 2H), 7.74 (d, *J* = 9.6 Hz, 1H), 7.62 (d, *J* = 7.2 Hz, 1H), 7.58 (d, *J* = 7.6 Hz, 1H), 7.53 (s, 1H), 7.37 - 7.40 (m, 2H), 7.29 - 7.32 (m, 2H), 6.85 (s, 1H), 5.20 (d, *J* = 8.8 Hz, 1H), 4.50 (t, *J* = 9.2 Hz, 1H), 4.15 - 4.21 (m, 1H), 4.02 - 4.08 (m, 2H), 3.74 (s, 3H), 0.87 - 0.97 (m, 21H)

^{13}C NMR (125 MHz $CDCl_3$) δ = 171.81, 155.58, 143.76, 143.53, 140.61, 139.58, 129.51, 129.37, 127.59, 127.05, 126.99, 125.41, 125.14, 120.02, 67.38, 65.87, 58.66, 46.44, 32.14, 17.63, 17.53, 11.77.

LCMS: $[M+H]^+$ = 564.3; R_t = 2.093 min.

HRMS (ESI/Q-TOF) m/z: $[M+H]^+$ calculated for $C_{31}H_{42}N_3O_5Si$ = 564.2888, found, 564.2855.

The aldolisation step with imidazole **13** using ClTi(OiPr)3 as Lewis acid followed by acidic deprotection delivered compound **31** which was directly protected with Fmoc group to furnish compound **32** with an excellent dr ratio >99% as no other diastereoisomers were seen in the different LCMS and NMR spectra of compound **26**, **27**, **28** and building block **8**.

Synthesis of compound (4R,5S)-2,2,5-trimethyl-1,3-dioxolane-4-carboxylic acid (7)

According to [25–27].

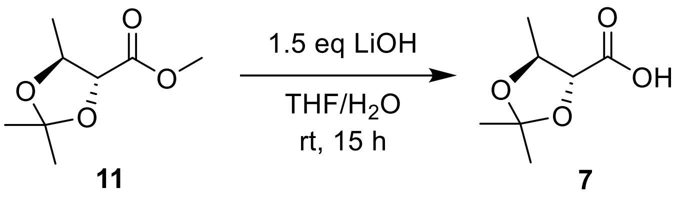

To a solution of 2.26 g (12,97 mmol)(4R,5S)-methyl 2,2,5-trimethyl-1,3-dioxolane-4-carboxylate **11** in THF (24 mL) is added a solution of Lithium hydroxide (466 mg, 19.46 mmol) in water (34 mL). The resulting mixture was stirred at room temperature for 15 h. A 10 % aqueous solution of citric acid was added until pH 5 and ethyl acetate was added. The organic phase was separated and the aqueous was extracted with EtOAc. The combined organic phase was washed with brine, dried over $MgSO_4$, filtered and concentrated under reduced pressure to afford compound **7** (2 g, 12.97 mol) as a white powder.

NMR data were in agreement with previously reported values:

^{1}H NMR (600 MHz, CDCl3) δ 10.23 (br, 1H), 4.24 (dq, J = 8.3, 5.9 Hz, 1H), 4.09 (d, J = 8.3 Hz, 1H), 1.49 (s, 3H), 1.48 (d, J = 5.9 Hz, 3H), 1.46 (s, 3H)

^{13}C NMR (125 MHz, CDCl3) δ 175.0, 111.0, 79.8, 75.2, 27.1, 25.6, 18.5.

HRMS (ESI/Q-TOF) m/z: $[M+H]^+$ calculated for $C_7H_{13}O_4$ 161.0808, found 161.0816.

10.2.1 General procedure for the synthesis of Corramycin (1)

Peptide Synthesis. Solid-phase peptide synthesis was carried out on a 0.1 mmol scale using Fmoc chemistry on 2-chlorotrityl polystyrene chloride resin (100-200 mesh, 1 % DVB) of charge 1.55 mmol g^{-1} from Novabiochem). Reactions were performed in a custom-built 20 mL glass fritted column fitted with a T-joint and three-way T-bore PTFE stopcock.

For the coupling of the first amino acid Fmoc-Ser(OTBDMS)-OH:

The resin was in Dichloromethane. The resin was swollen in DCM for 30 min and the DCM is then drained off. The resin was transferred into a round-bottomed flask. A solution of 3.3 g (3.0 eq) of Fmoc-L-Ser(OTBDMS)-OH and 3.8 mL (3.0 eq.) of DIPEA in 30 mL of DCM was then poured onto the resin. The mixture obtained was stirred overnight. The reaction mixture was then drained off and the resin was washed three times with DMF, once with MeOH and then three times with DCM, with draining after each wash. After drying under reduced pressure for 4 h, 4.3 g of resin were obtained.

For the coupling of the other amino acids and building blocks 10, 9, 8 and 7:

The resin was preswollen by bubbling in DMF (5 mL, 10 min) with argon. Between deprotections and couplings the vessel was drained under argon pressure and washed with DMF (3 × 5 mL). The Fmoc group was removed by bubbling with 20 % piperidine in DMF (3 × 5 mL × 5 min). The deprotection steps were monitored by UV (254 nm) detection of the washings. All amino acids and building blocks **10**, **9**, **8** and **7** (3.0 eq) were preactivated by shaking with HATU (6.0 eq) and DIPEA (6 .0 eq) in DMF (5 mL) for 5 min. The resin was bubbled in the coupling solution for 1 h, drained, and washed with DMF (3 × 5 mL).

The deprotection and coupling steps were continued to complete the peptide synthesis. The resin-bound peptide was washed with DCM (3 × 5 mL) and dried under argon for 20 min.

For the deprotection of TIPS and TBDMS group:

The resin was bubbled in a solution of tetra-*n*-butylammonium fluoride 1N in THF (2 mL) for 12 h. The mixture was then drained off and the resin is washed three times with THF, one time with methanol and three times with dichloromethane with draining after each wash. After drying under reduced pressure for 4 h.

For the deprotection of acetal and cleavage from the resin:

The resin was transferred to a screw top vial containing TFAcOH/TIPS/H2O (95/2.5/2.5, 5 mL) and gently shaken for 2 h. The cleavage solution was filtered and concentrated in vacuo, and the crude peptide was precipitated with cold diethyl ether. The crude peptide was dissolved in H2O:ACN (1:1, 5 mL) and purified using prep-scale HPLC:

Phenomenex C-18 column, flow rate 10 mL min^{-1}, detected at 220 nm. Gradient: Starting from 20 % ACN (0.1 % TFAcOH) and 80 % H_2O (0.1 % TFAcOH) for 5 min, ramping up to 55 % ACN over 30 min, then ramping up to 95 % ACN over 3 min, staying at 95 % ACN for 3 min, ramping down to 20 % ACN over 2 min, then staying at 20 % ACN for 5 min. The product containing fractions were pooled, concentrated, frozen, and lyophilized to yield the product as a white powder.

HRMS (ESI/Q-TOF) m/z: $[M]^+$ calculated for $C_{51}H_{81}N_{11}O_{21}$ 1183.5608, found, 1183.5613

10.3 NMR data of all Building Blocks and synthetic Corramycin

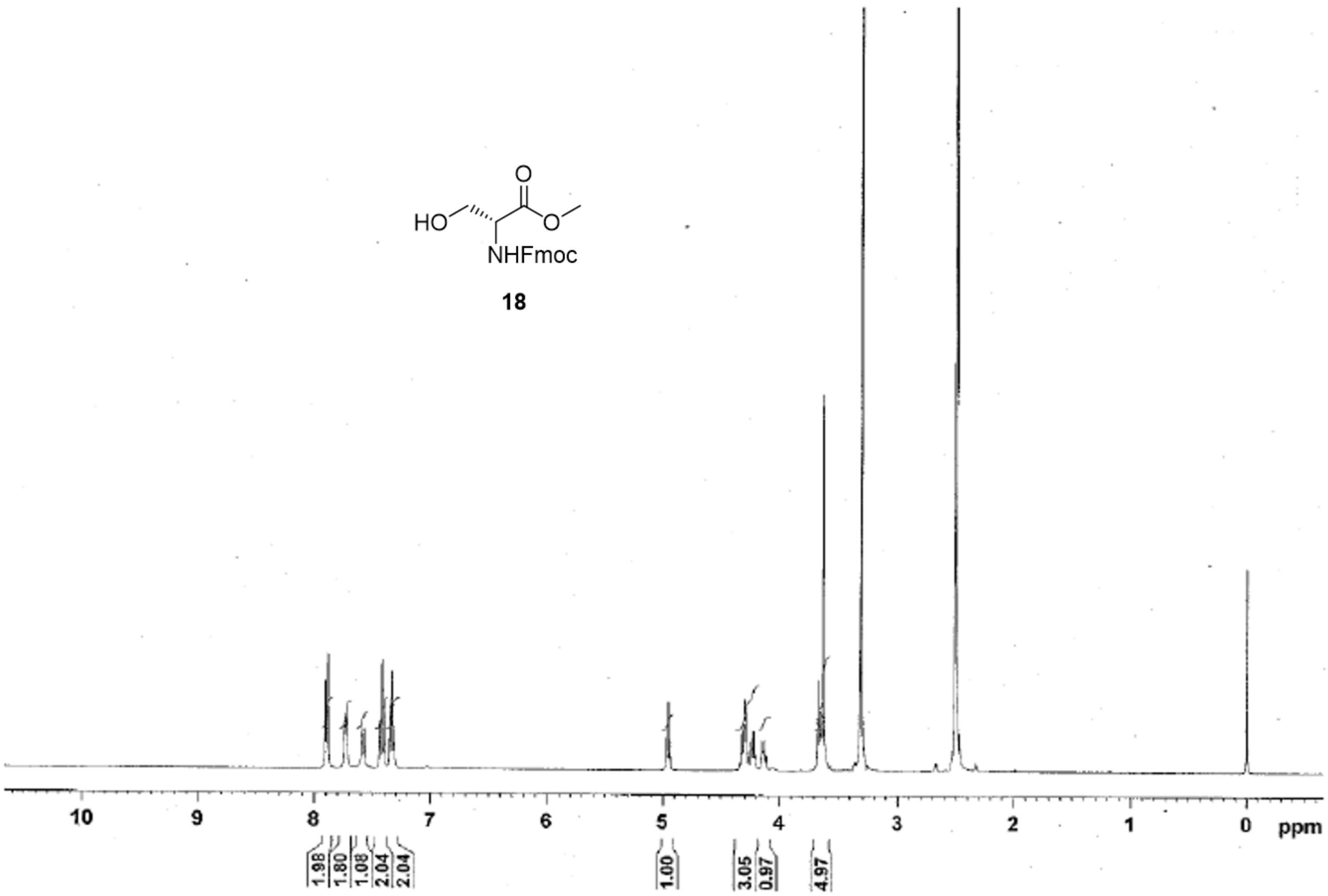

Figure S 33. ^{1}H NMR of compound **18**, 600 MHz, DMSO-d_6.

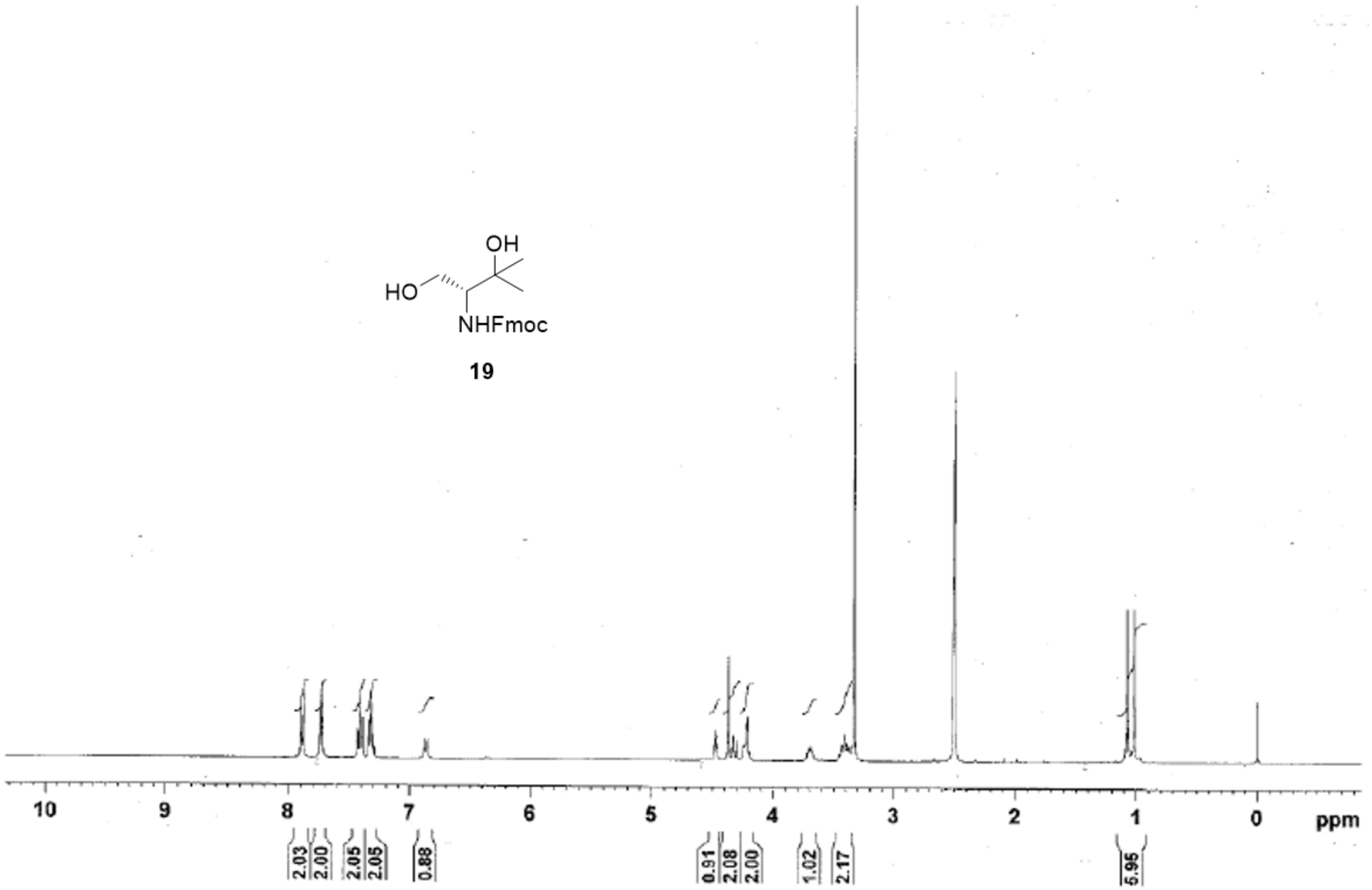

Figure S 34. ^{1}H NMR of compound 19, 600 MHz, DMSO-d_6.

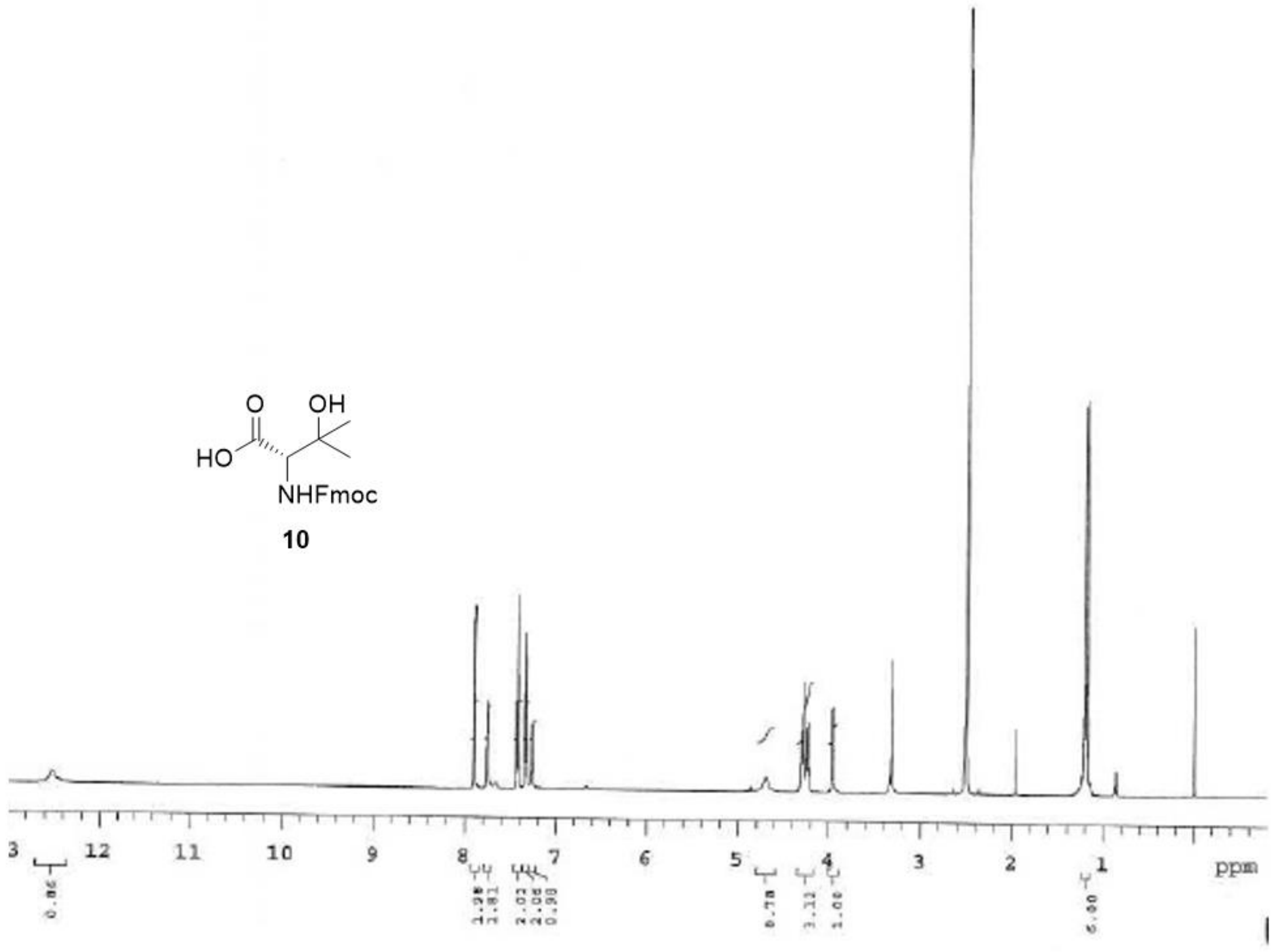

Figure S 35. ^{1}H NMR of compound 10, 600 MHz, DMSO-d_6.

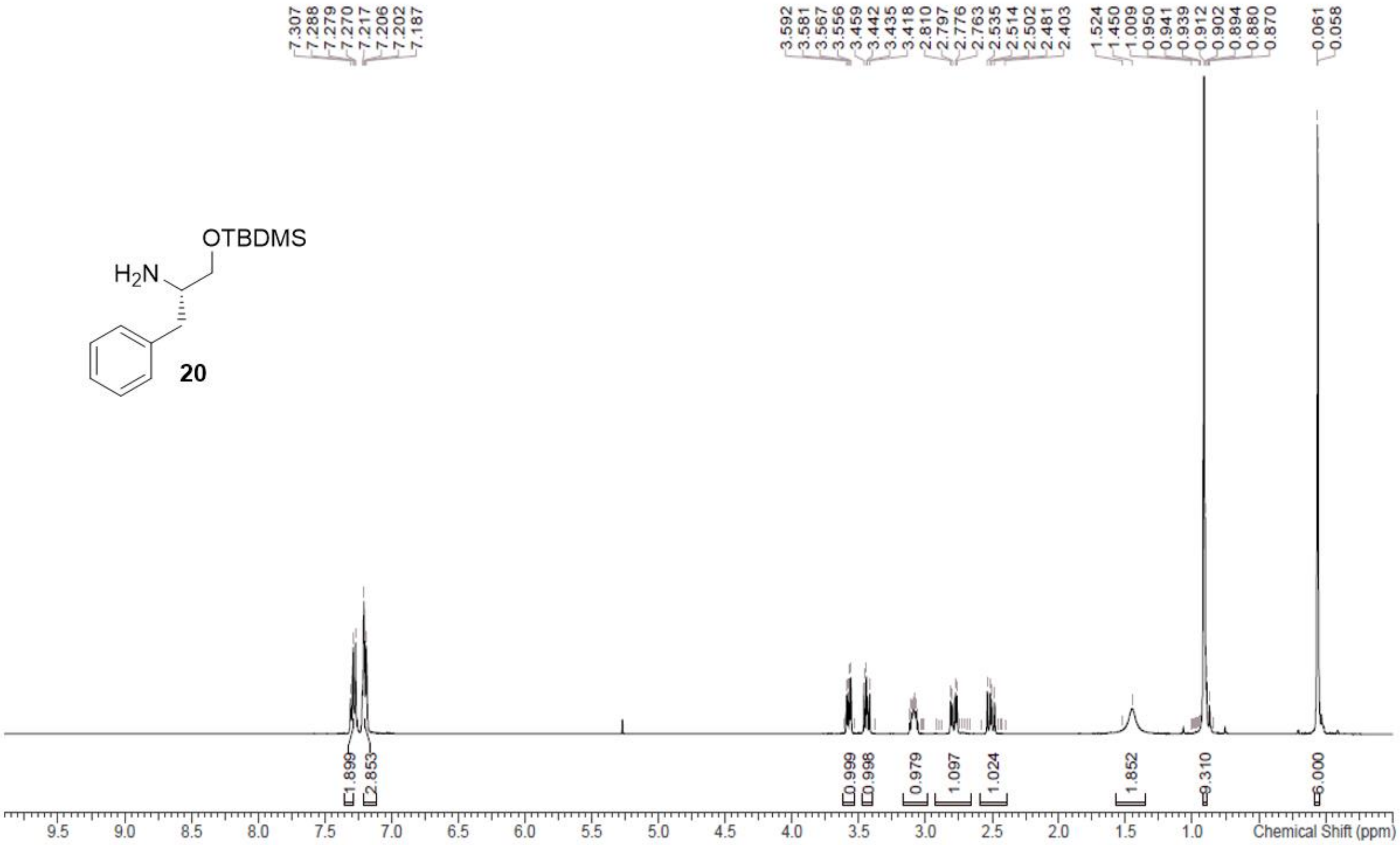

Figure S 36. ^{1}H NMR of compound 20, 600 MHz, $CDCl_3$.

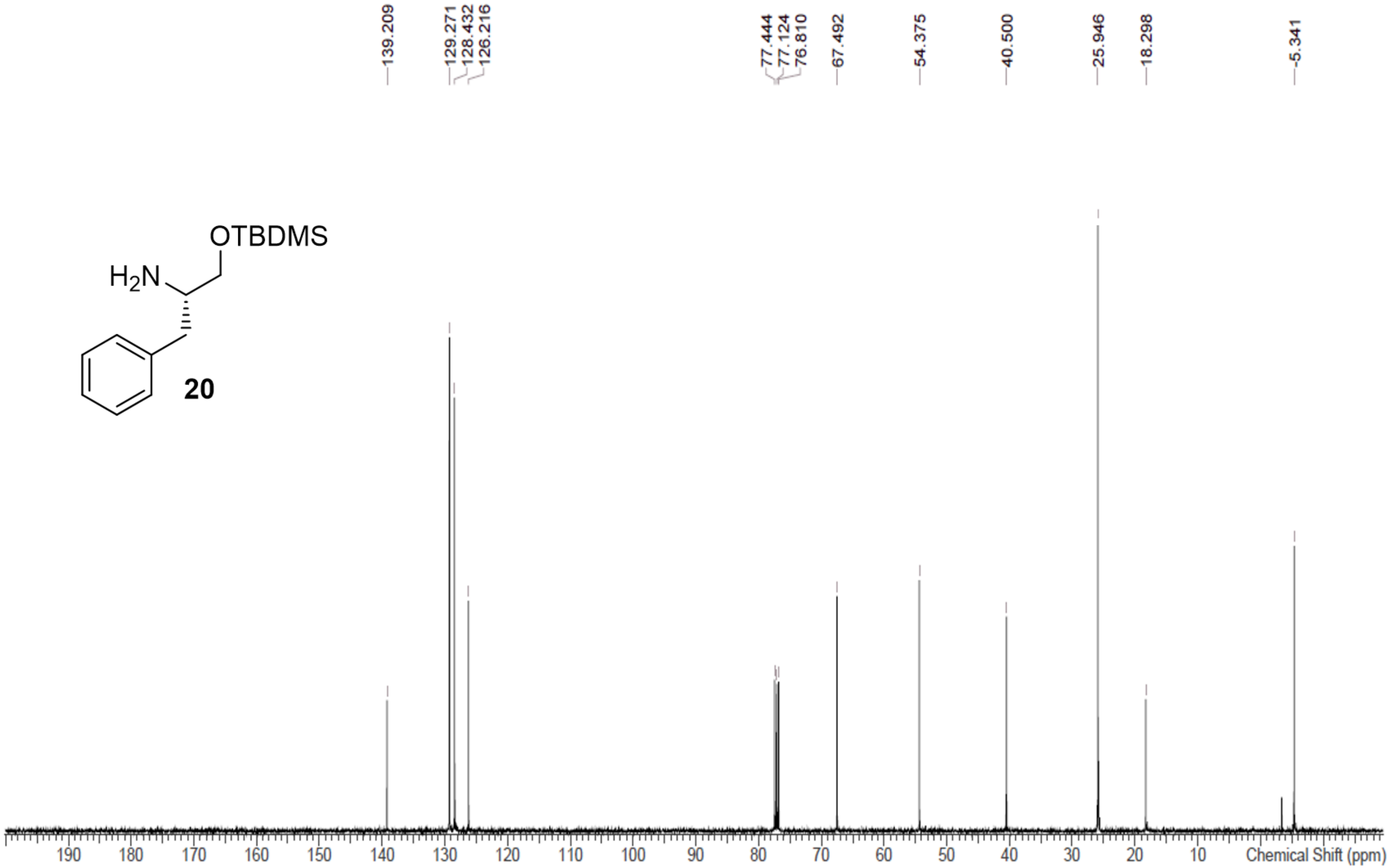

Figure S 37. ^{13}C NMR of compound 20, 150 MHz, $CDCl_3$.

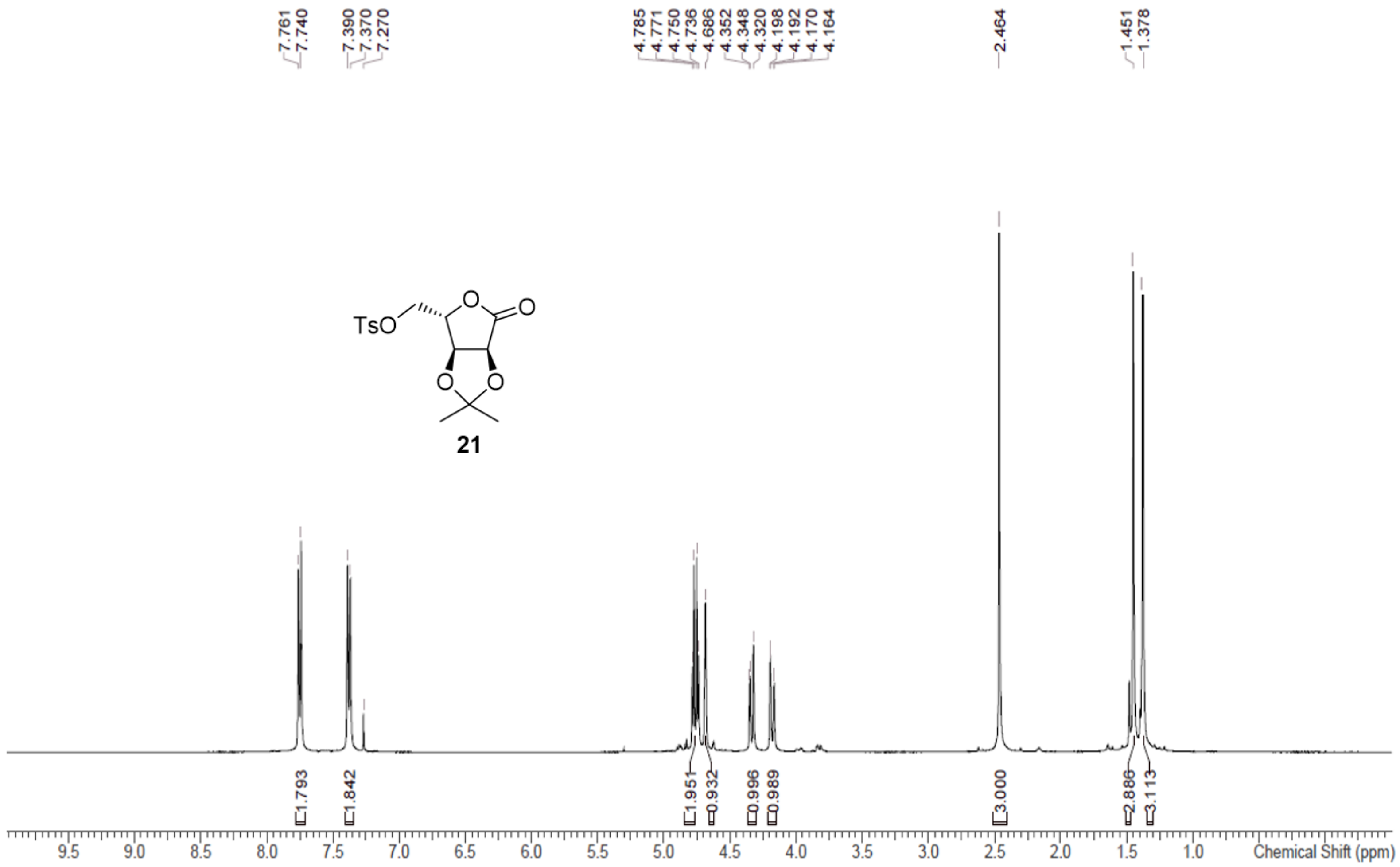

Figure S 38. ^{1}H NMR of compound 21, 600 MHz, $CDCl_3$.

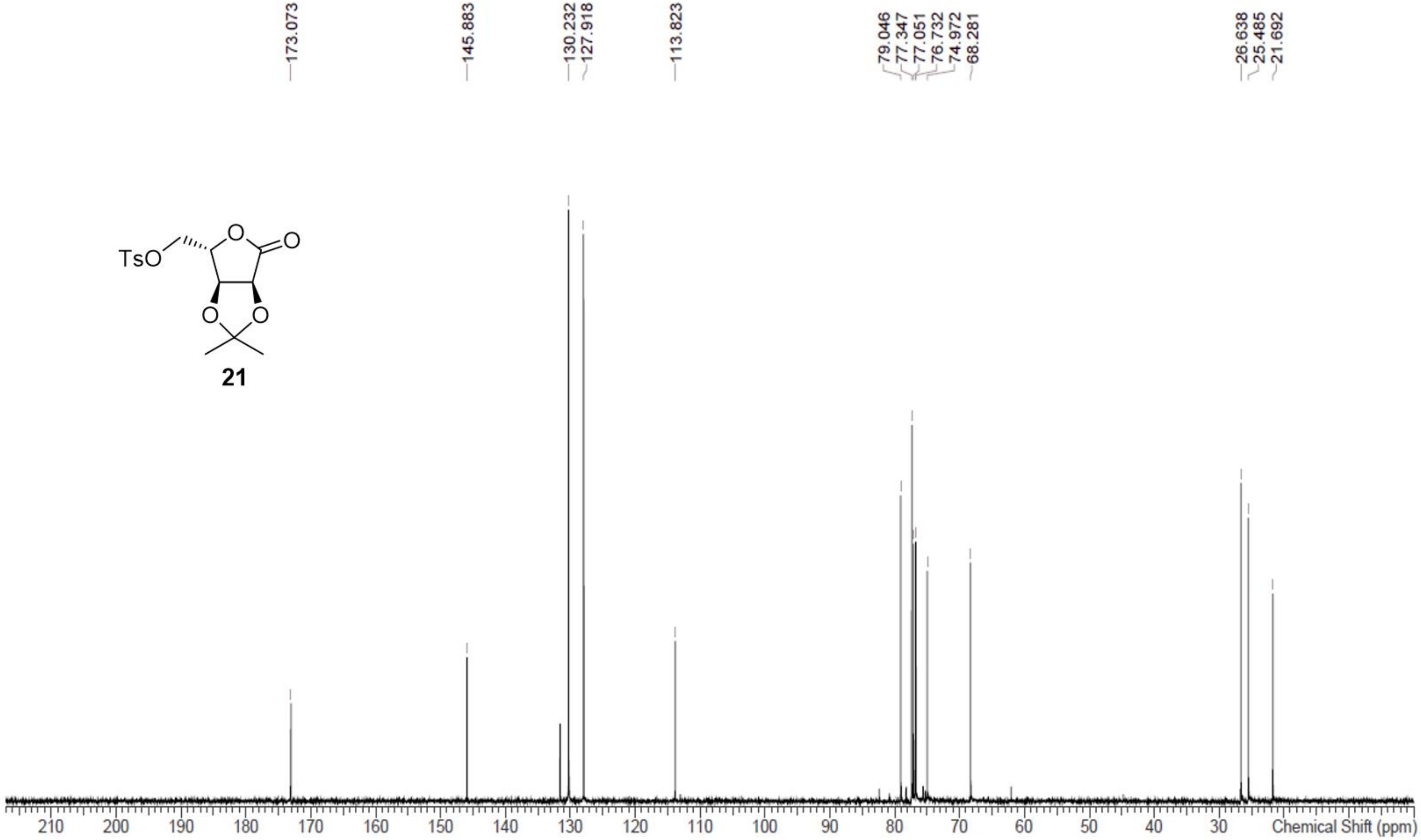

Figure S 39. ^{13}C NMR of compound 21, 150 MHz, $CDCl_3$.

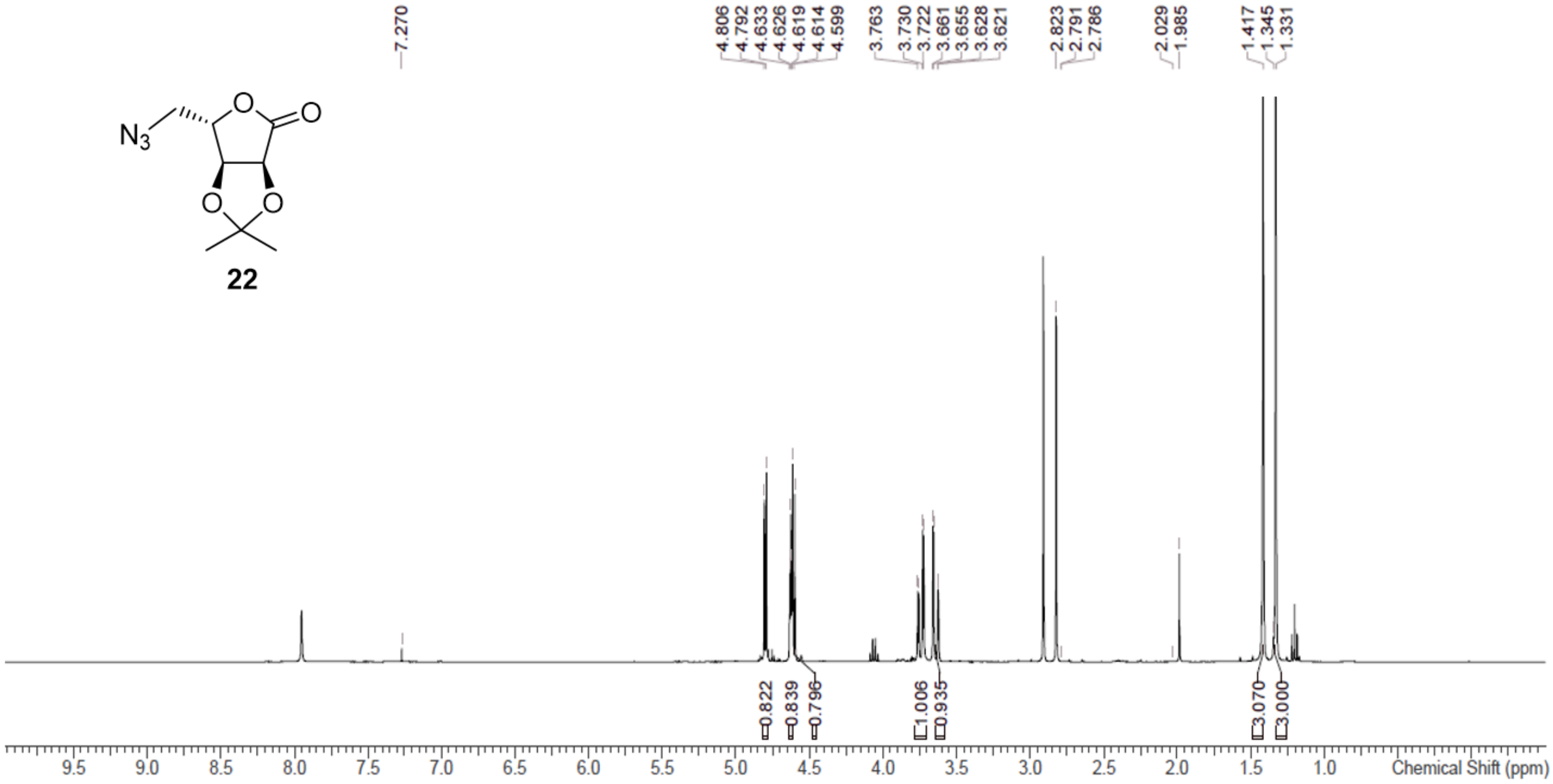

Figure S 40. ^{1}H NMR of compound 22, 600 MHz, $CDCl_3$.

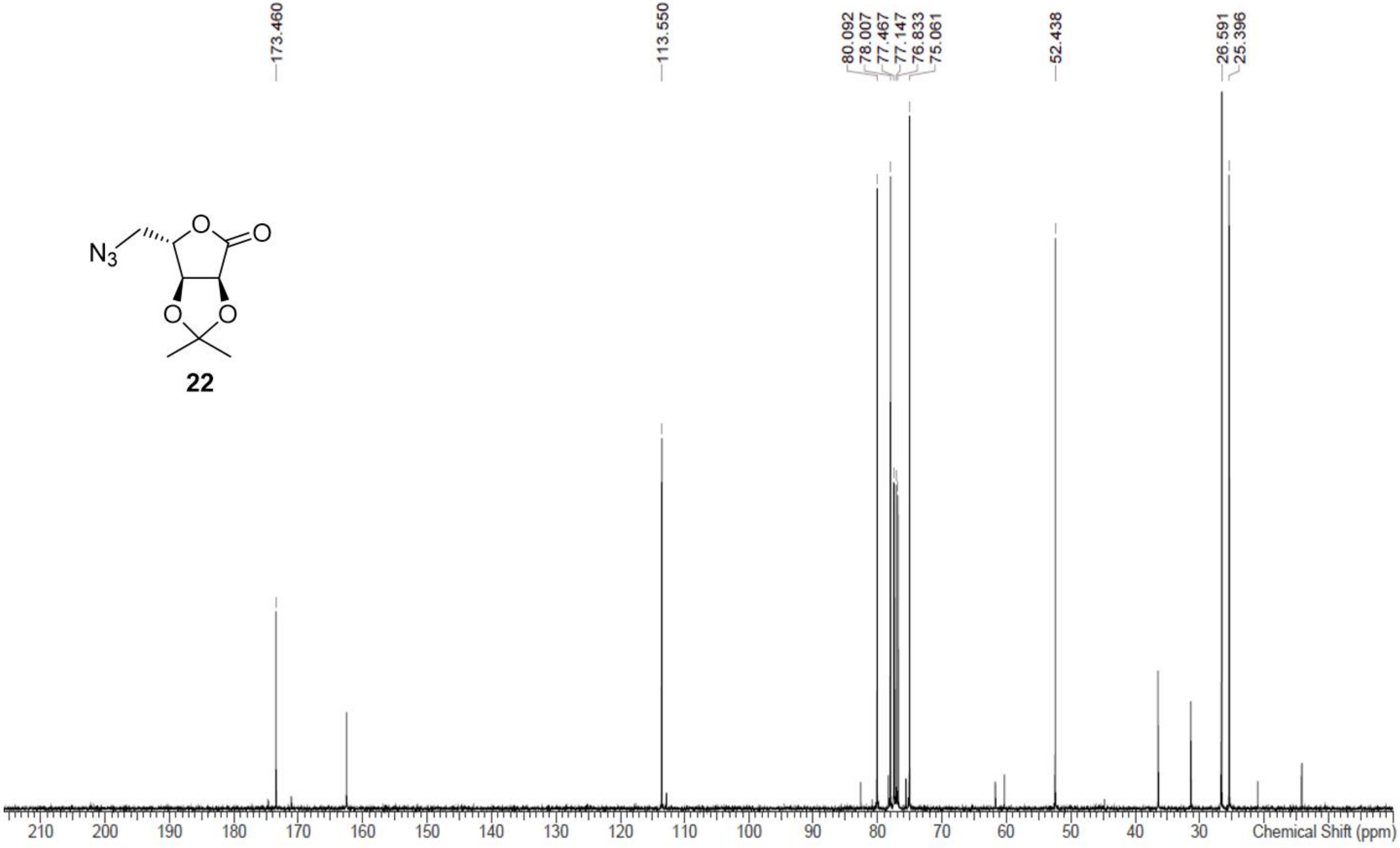

Figure S 41. ^{13}C NMR of compound 22, 150 MHz, $CDCl_3$.

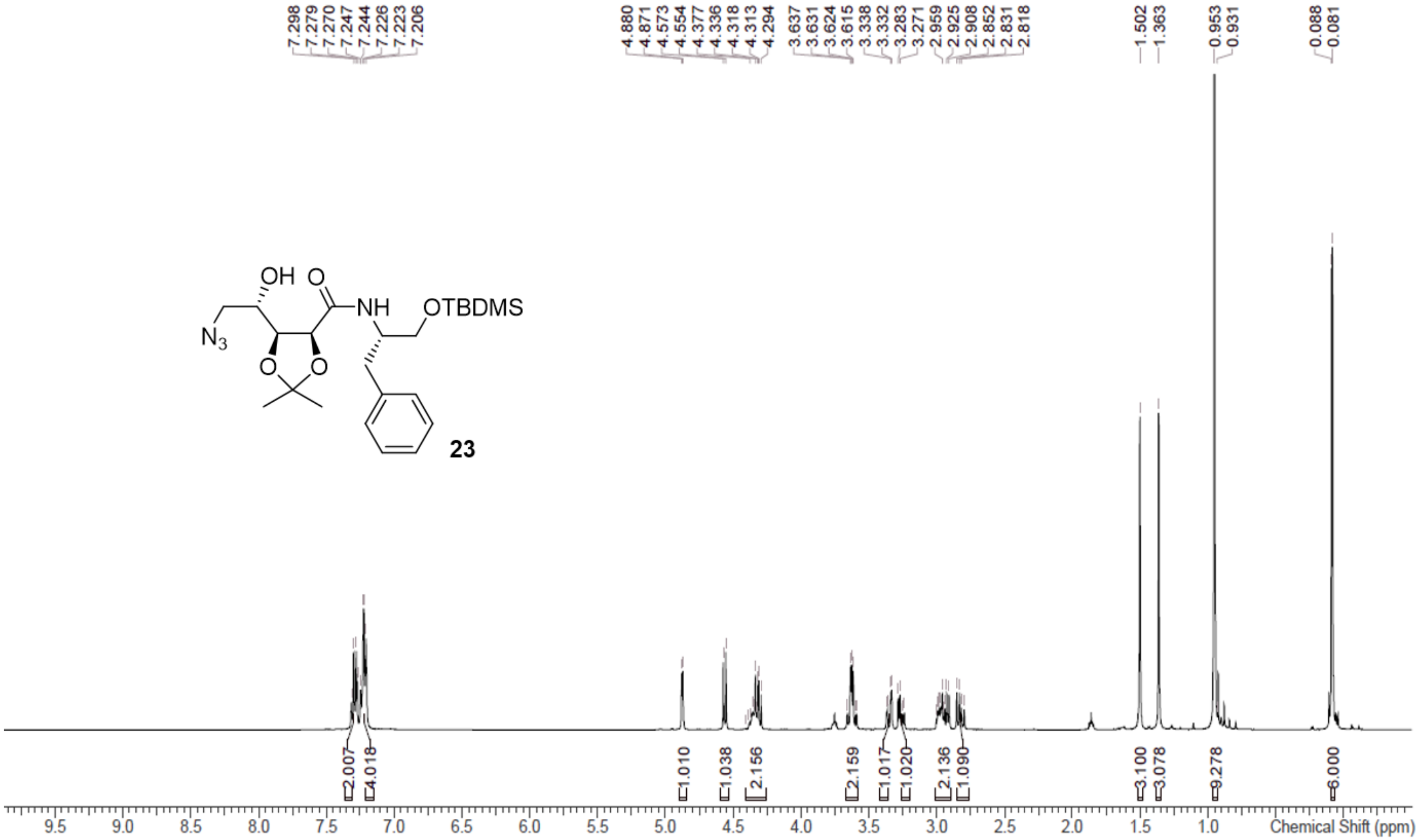

Figure S 42. ^{1}H NMR of compound 23, 600 MHz, $CDCl_3$.

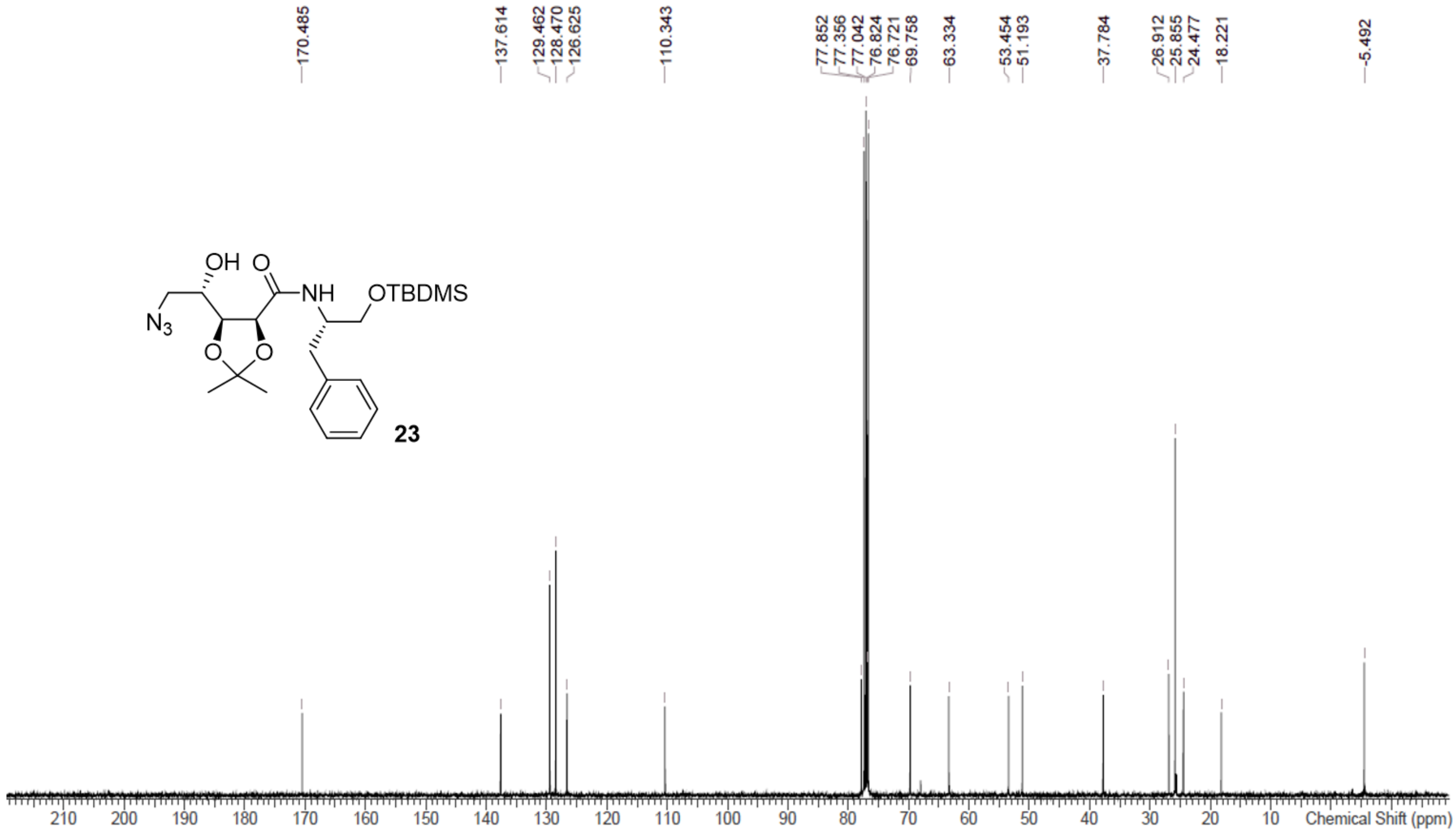

Figure S 43. ^{13}C NMR of compound 23, 150 MHz, $CDCl_3$.

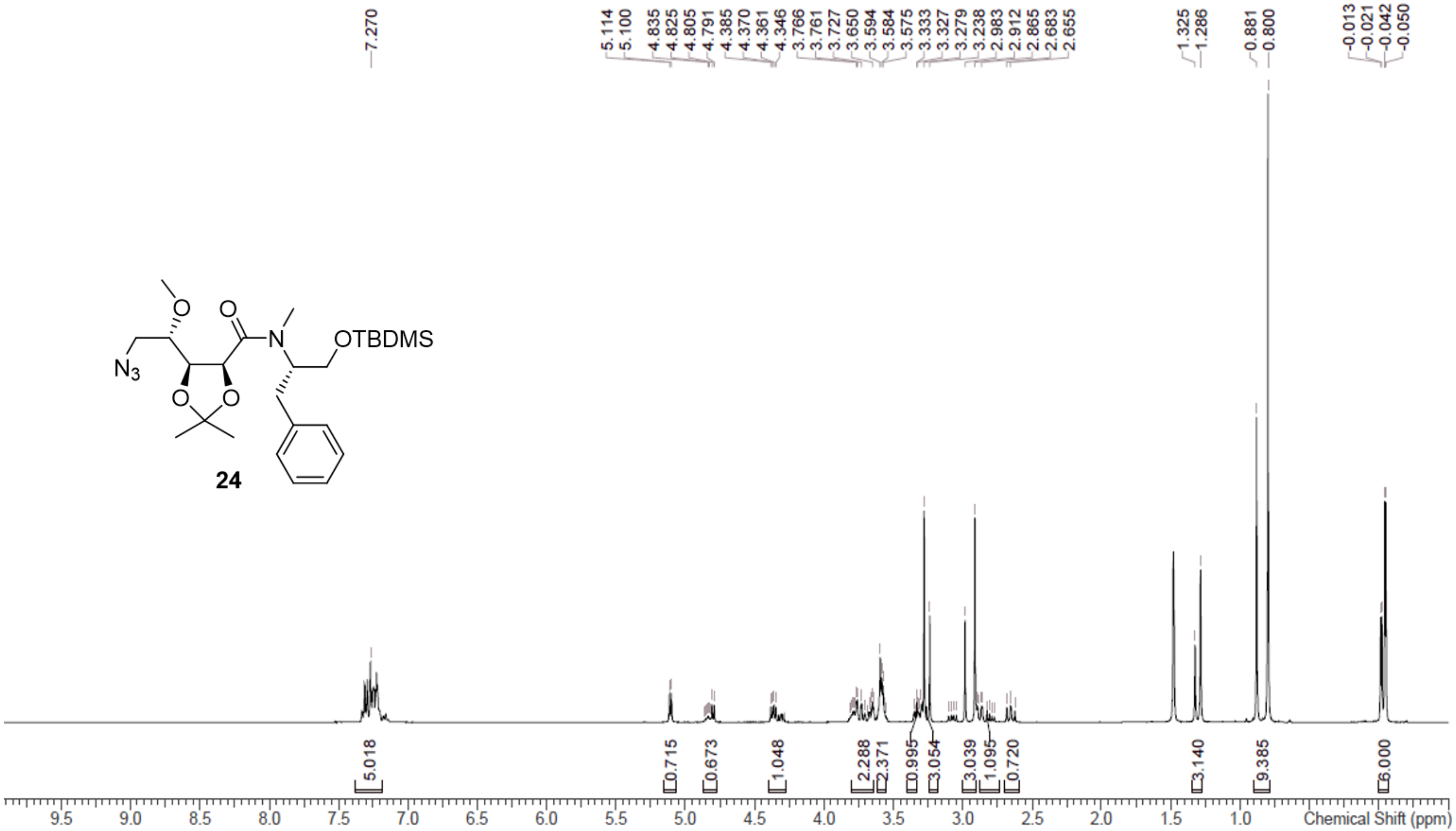

Figure S 44. ^1H NMR of compound 24, 600 MHz, $CDCl_3$.

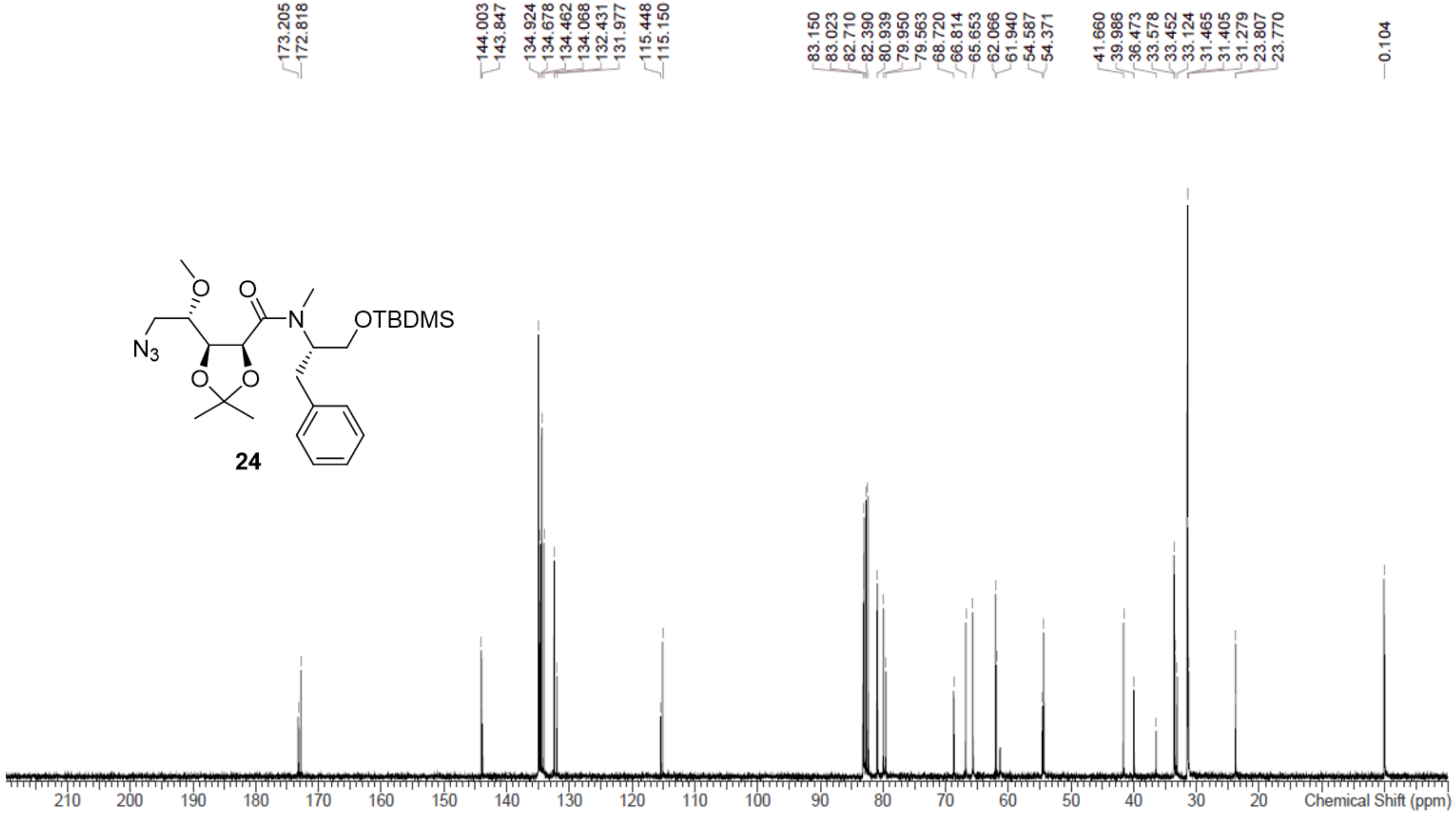

Figure S 45. ^{13}C NMR of compound 24, 150 MHz, $CDCl_3$.

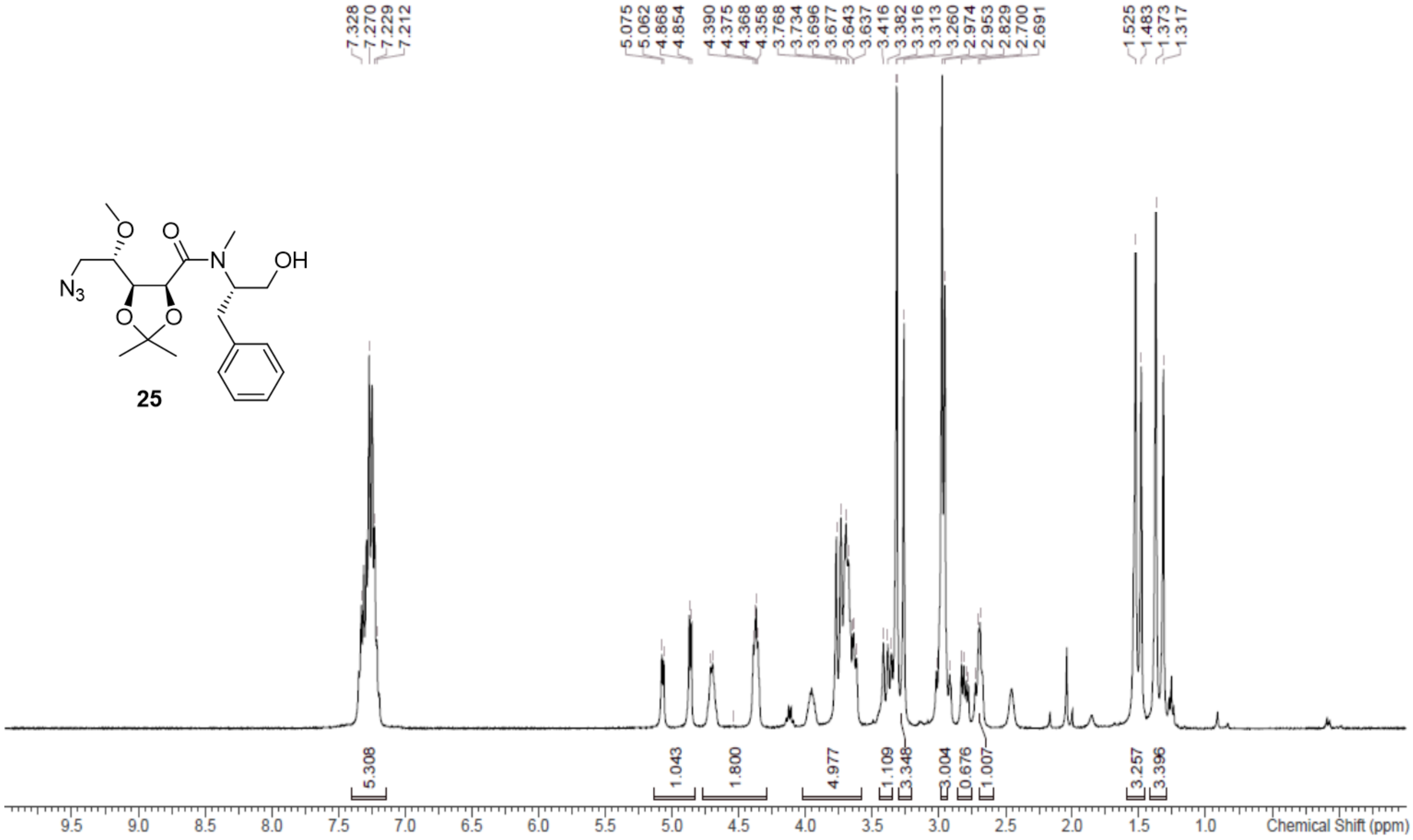

Figure S 46. ^{1}H NMR of compound 25, 600 MHz, $CDCl_3$.

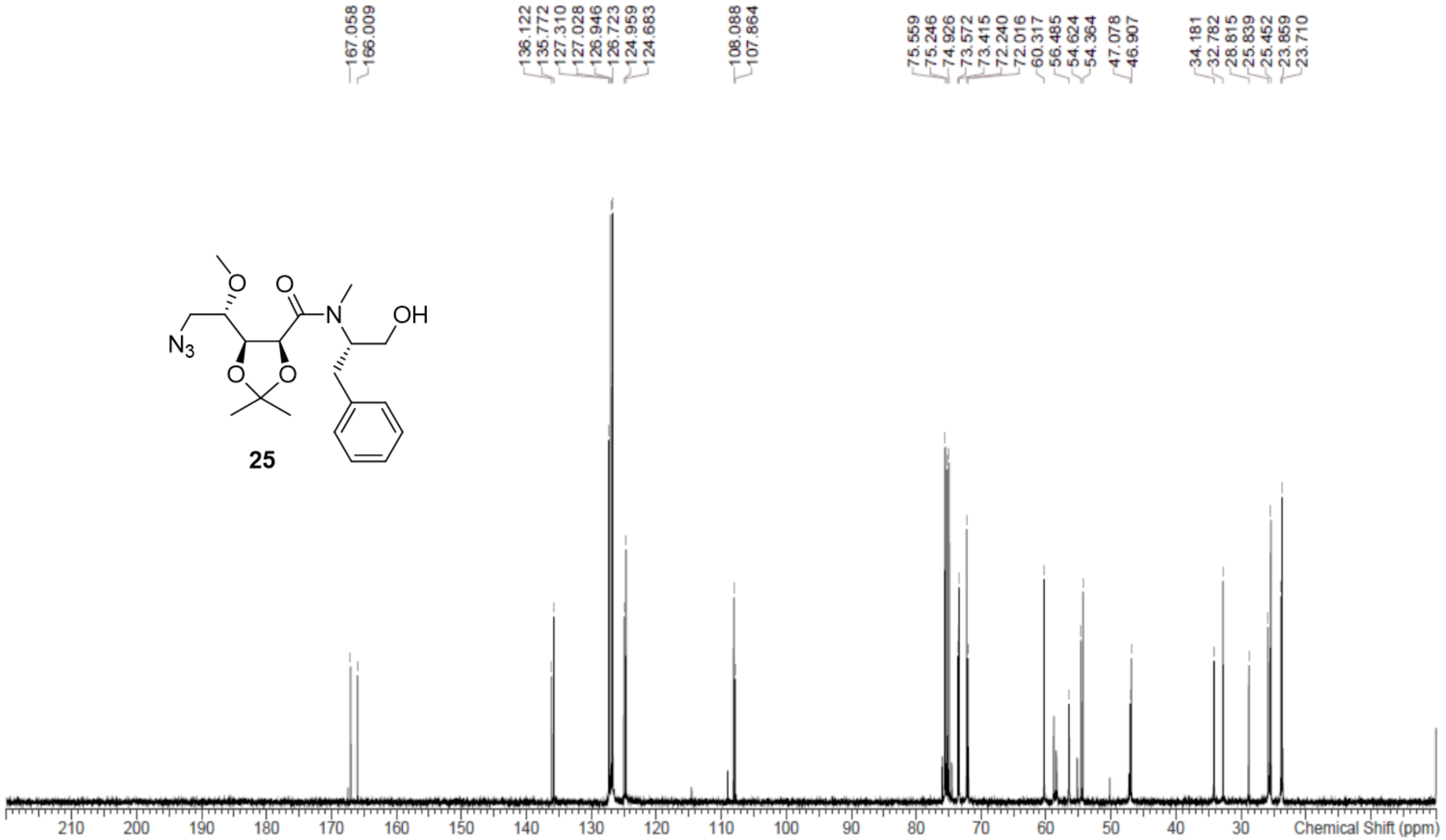

Figure S 47. ^{13}C NMR of compound 25, 150 MHz, $CDCl_3$.

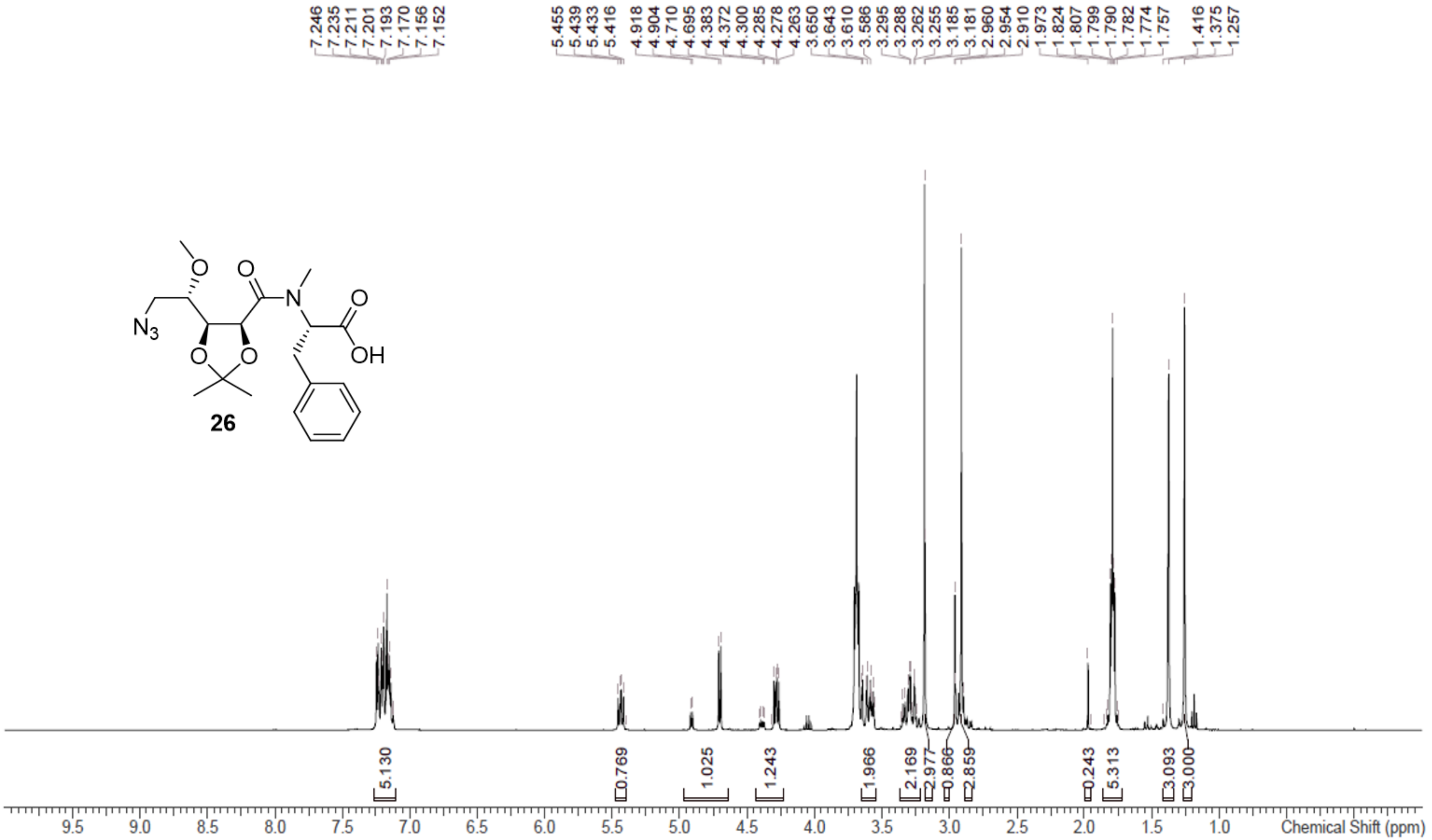

Figure S 48. ^{1}H NMR of compound 26, 600 MHz, $CDCl_3$.

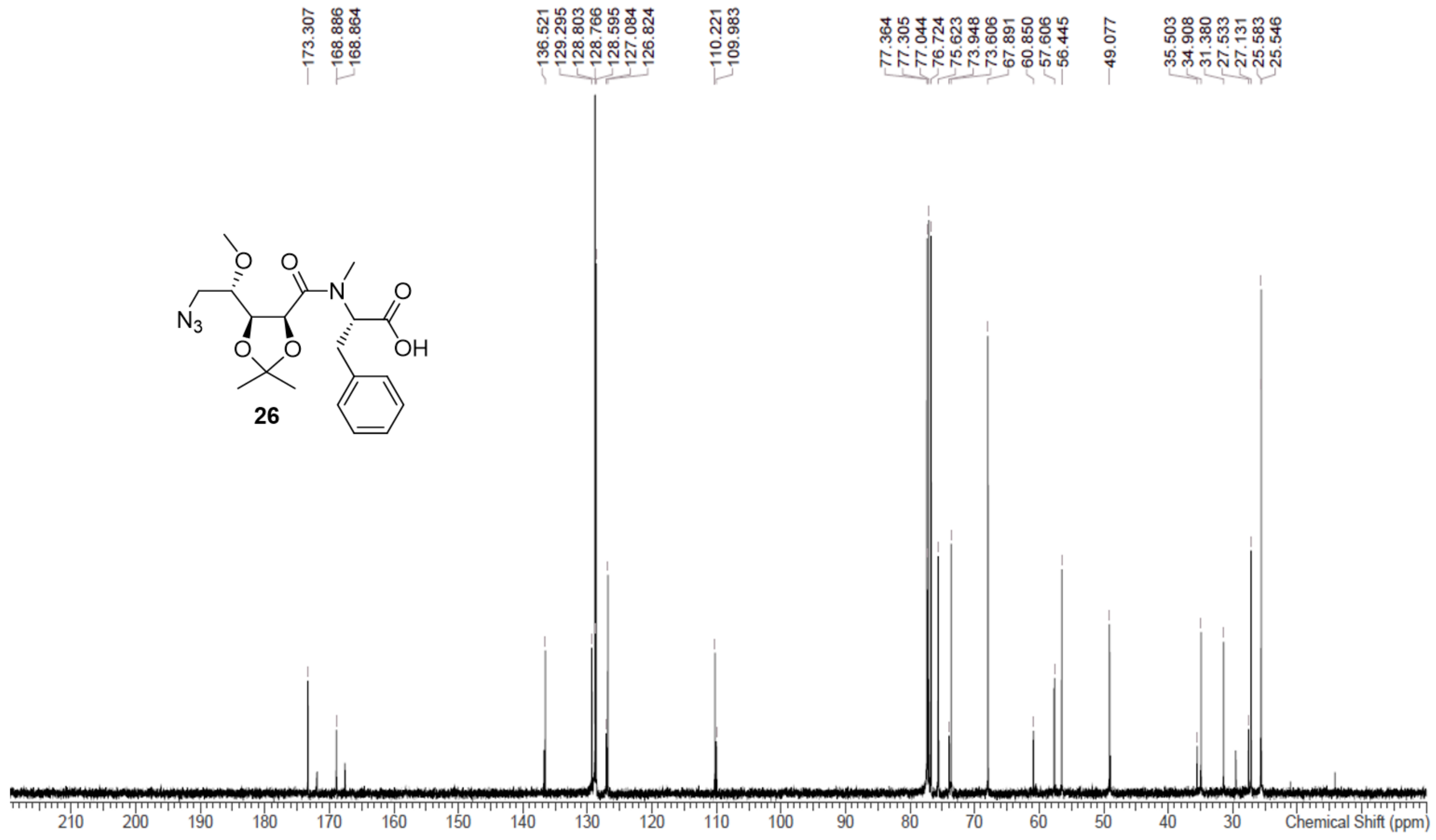

Figure S 49. ^{13}C NMR of compound 26, 150 MHz, $CDCl_3$.

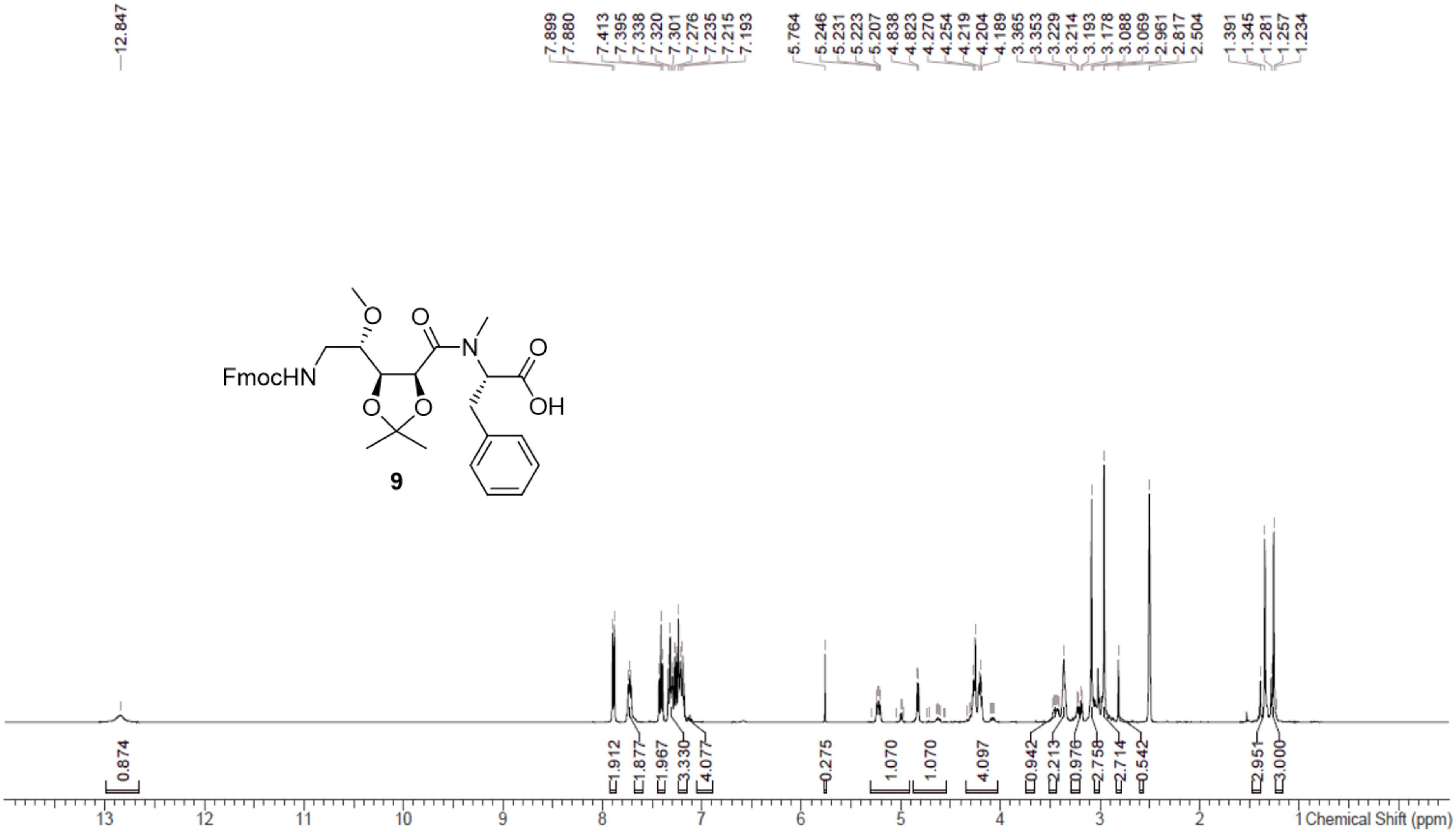

Figure S 50. ^{1}H NMR of compound 9, 600 MHz, DMSO-d_6.

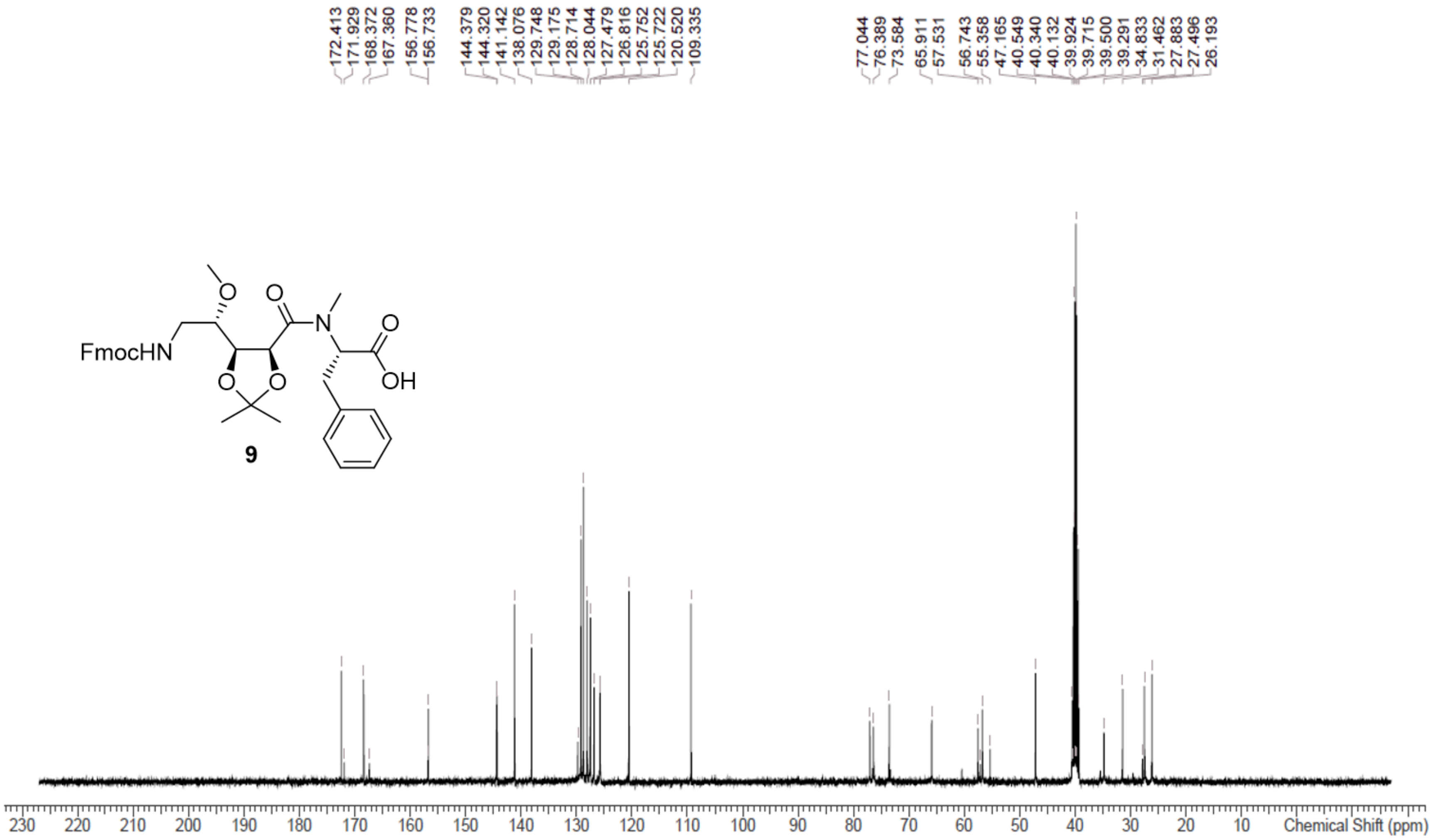

Figure S 51. ^{13}C NMR of compound 9, 150 MHz, DMSO-d_6.

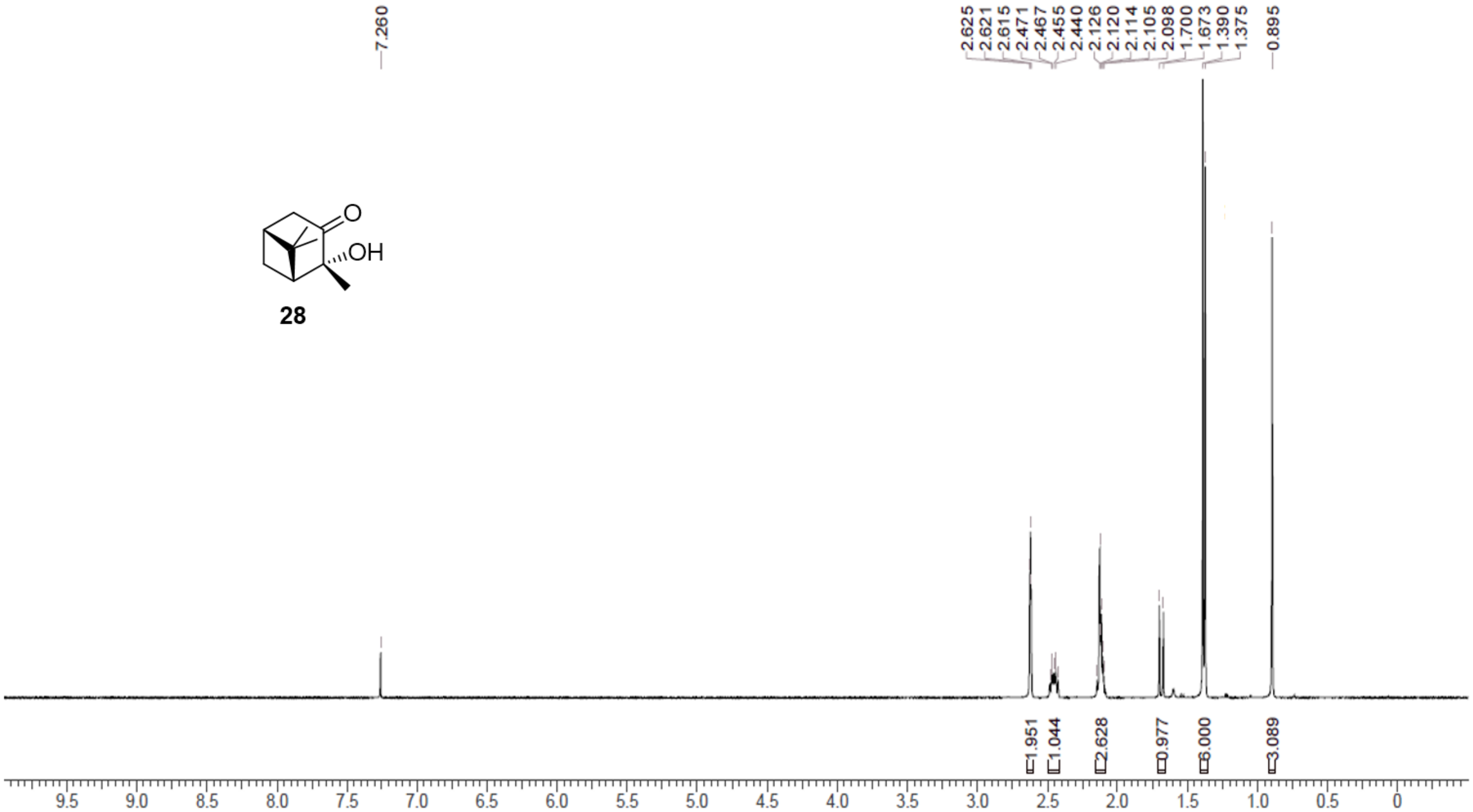

Figure S 52. ^{1}H NMR of compound 28, 600 MHz, $CDCl_3$.

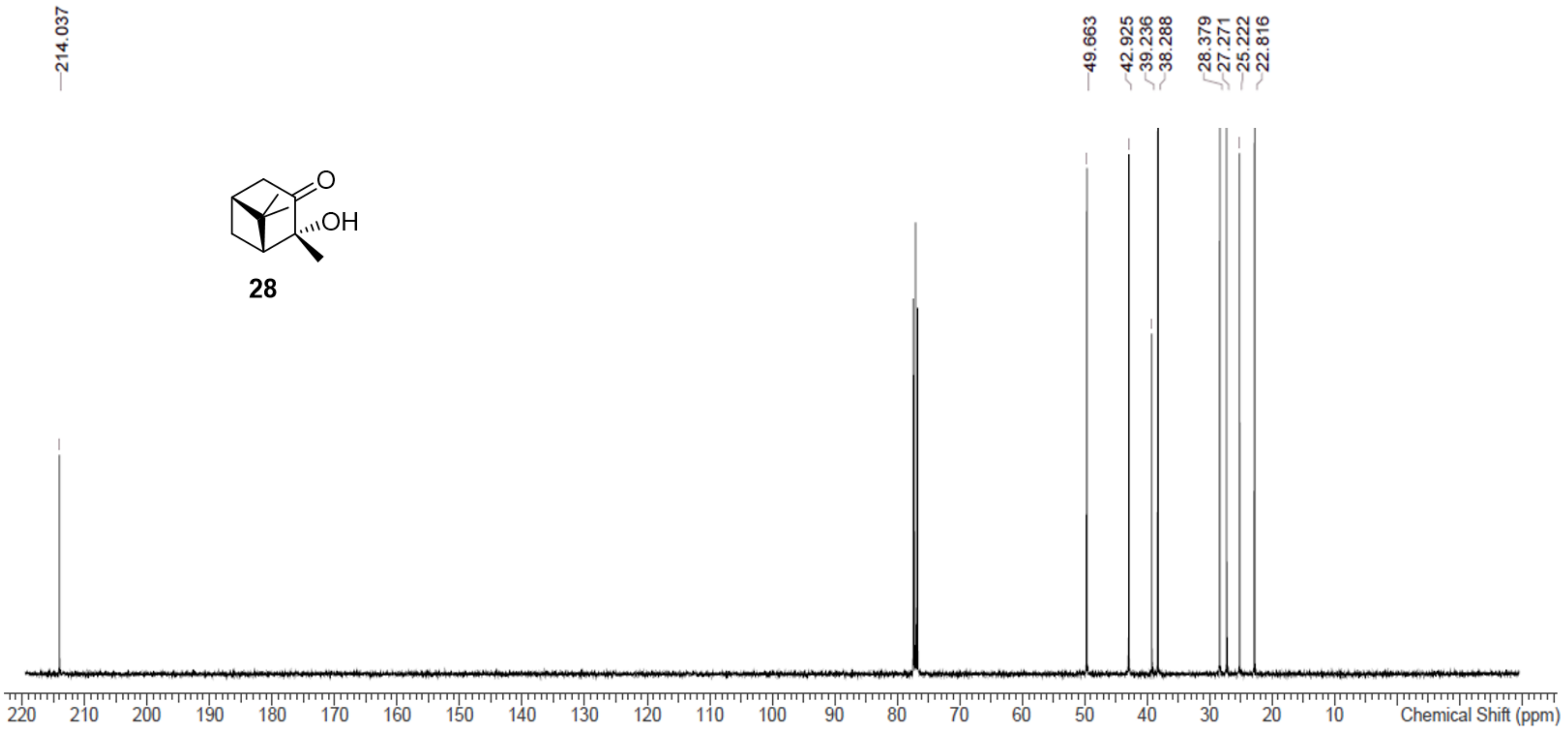

Figure S 53. ^{13}C NMR of compound 28, 150 MHz, $CDCl_3$.

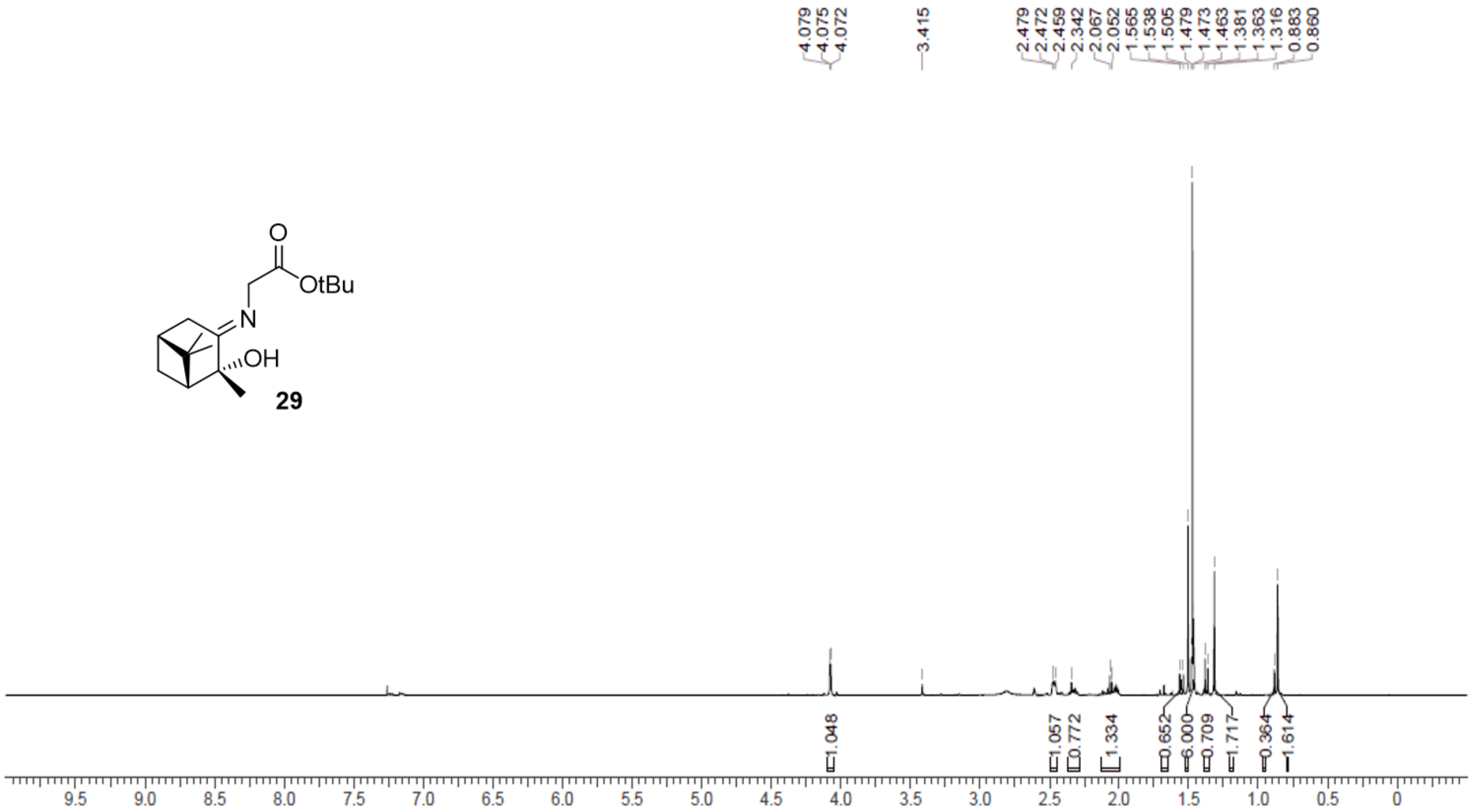

Figure S 54. ^{1}H NMR of compound 29, 600 MHz, $CDCl_3$.

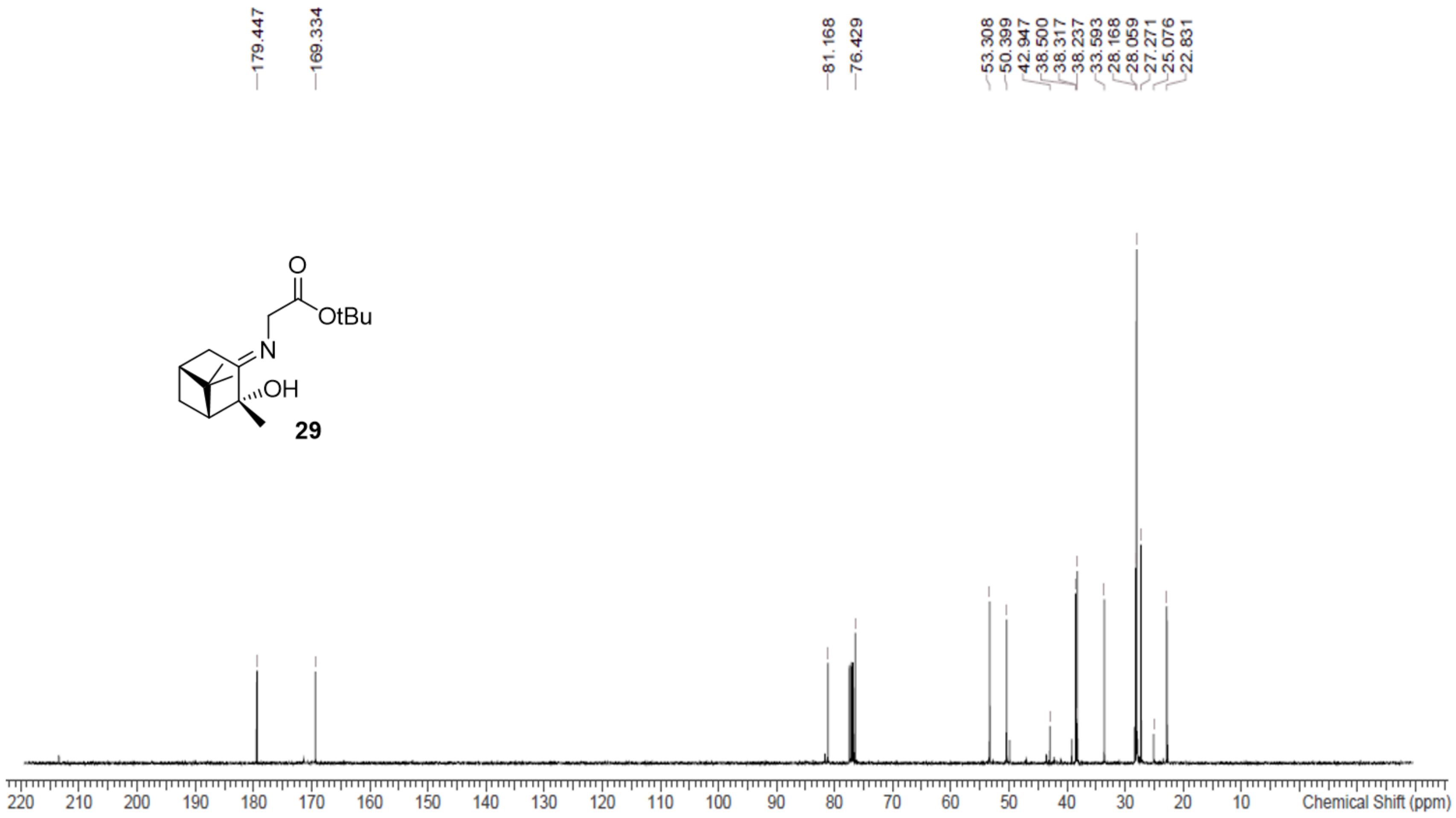

Figure S 55. ^{13}C NMR of compound 29, 150 MHz, $CDCl_3$.

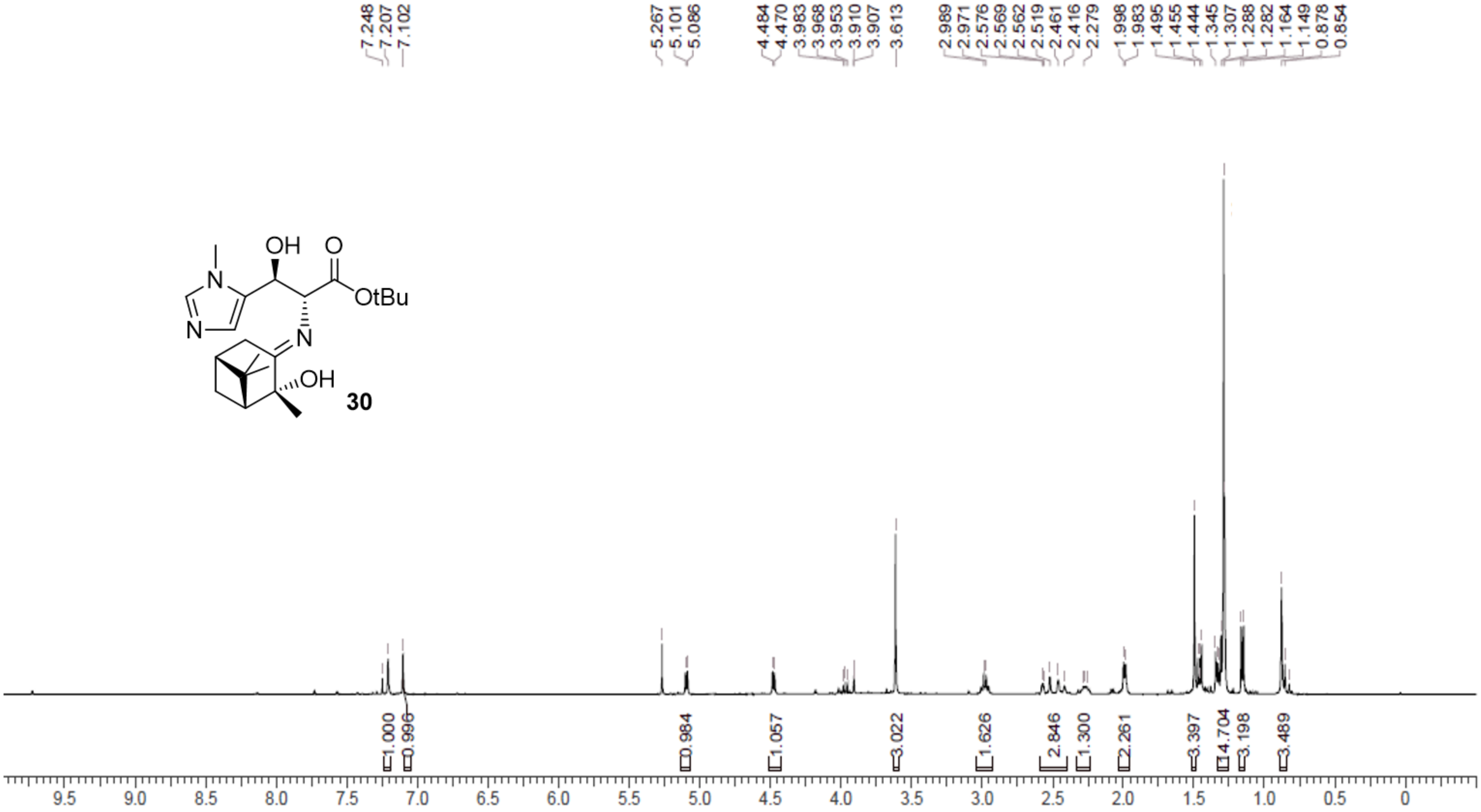

Figure S 56. ^{1}H NMR of compound 30, 600 MHz, $CDCl_3$.

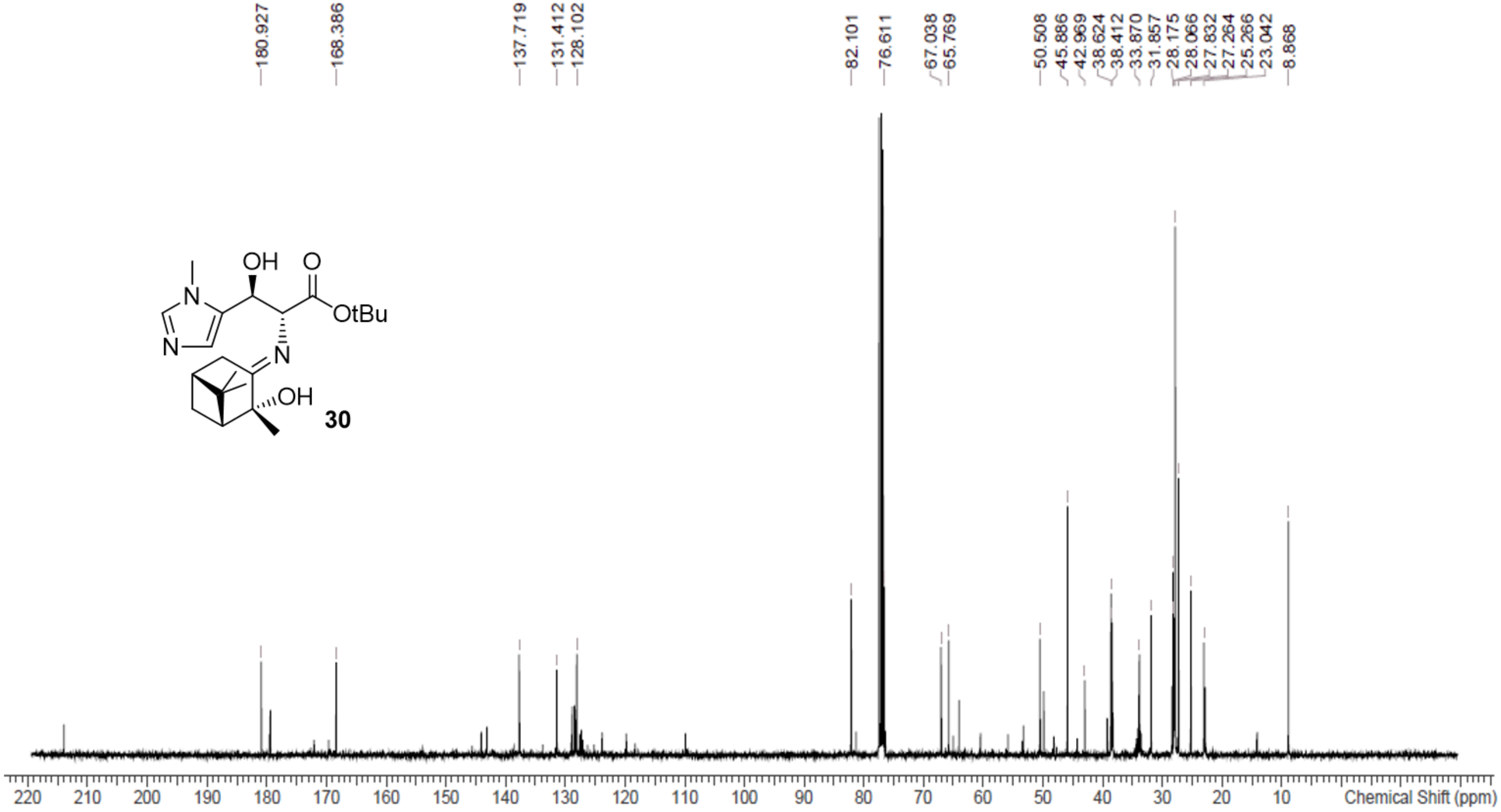

Figure S 57. ^{13}C NMR of compound 30, 150 MHz, $CDCl_3$.

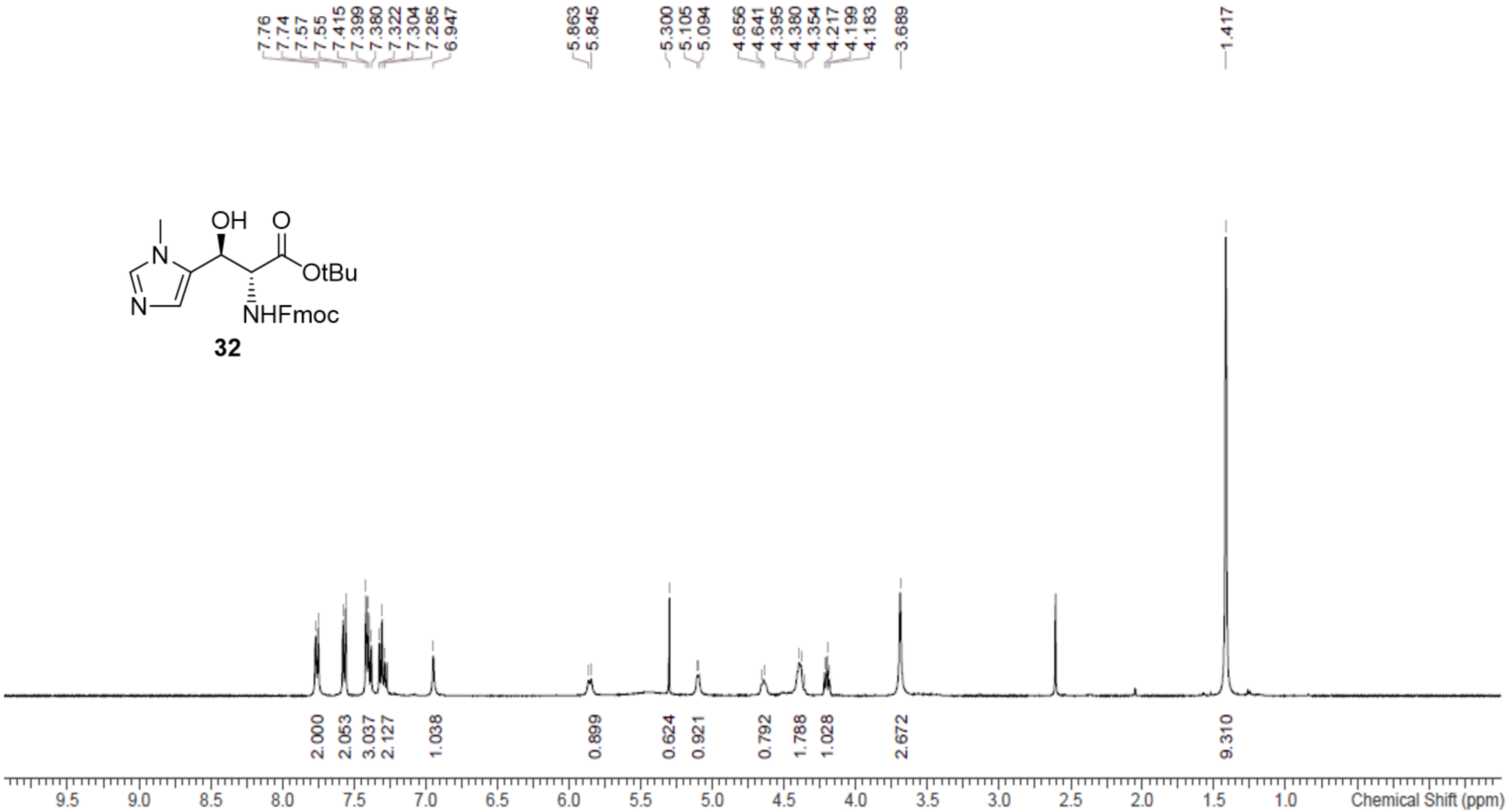

Figure S 58. ^{1}H NMR of compound 32, 600 MHz, $CDCl_3$.

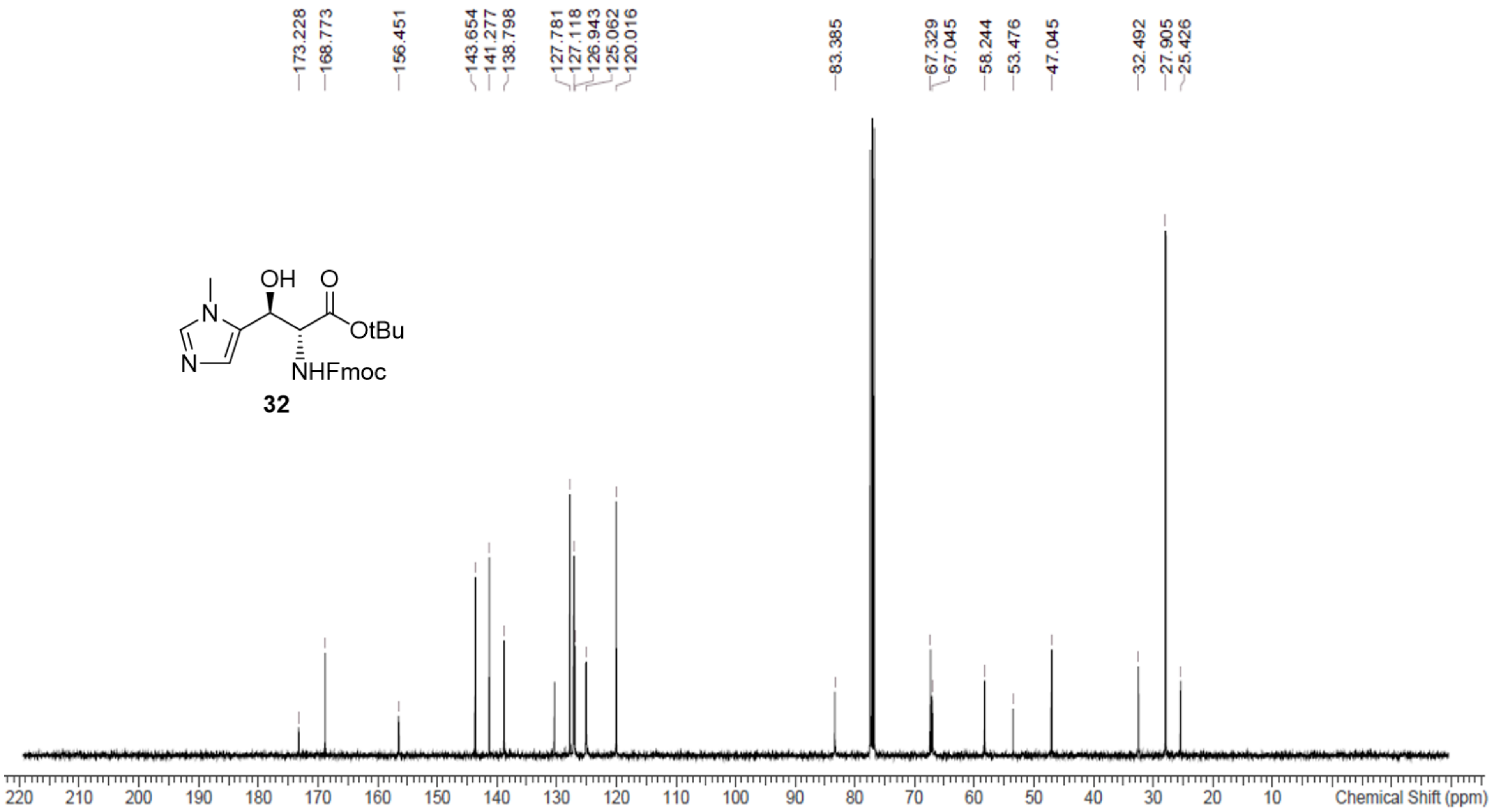

Figure S 59. ^{13}C NMR of compound 32, 150 MHz, $CDCl_3$.

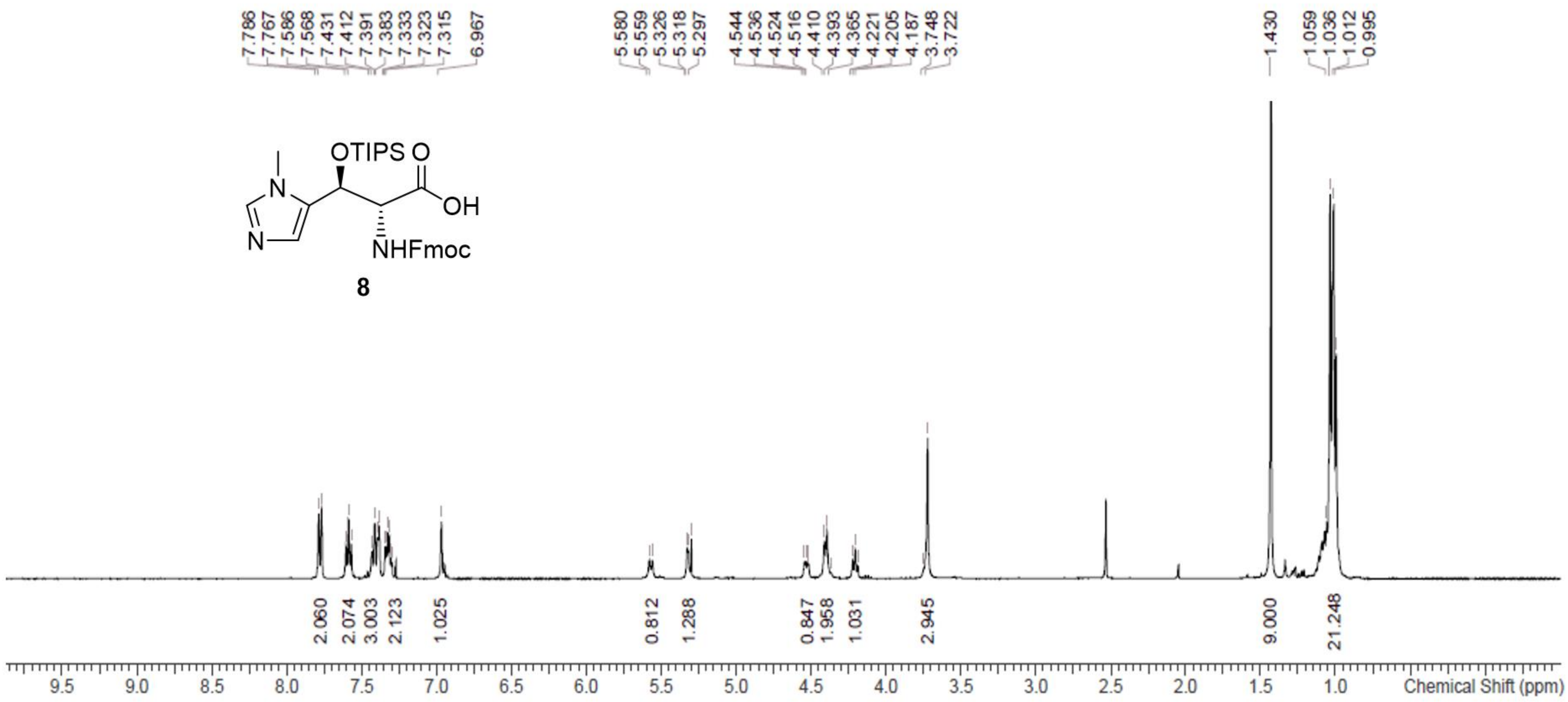

Figure S 60. ^{1}H NMR of compound 8, 600 MHz, $CDCl_3$.

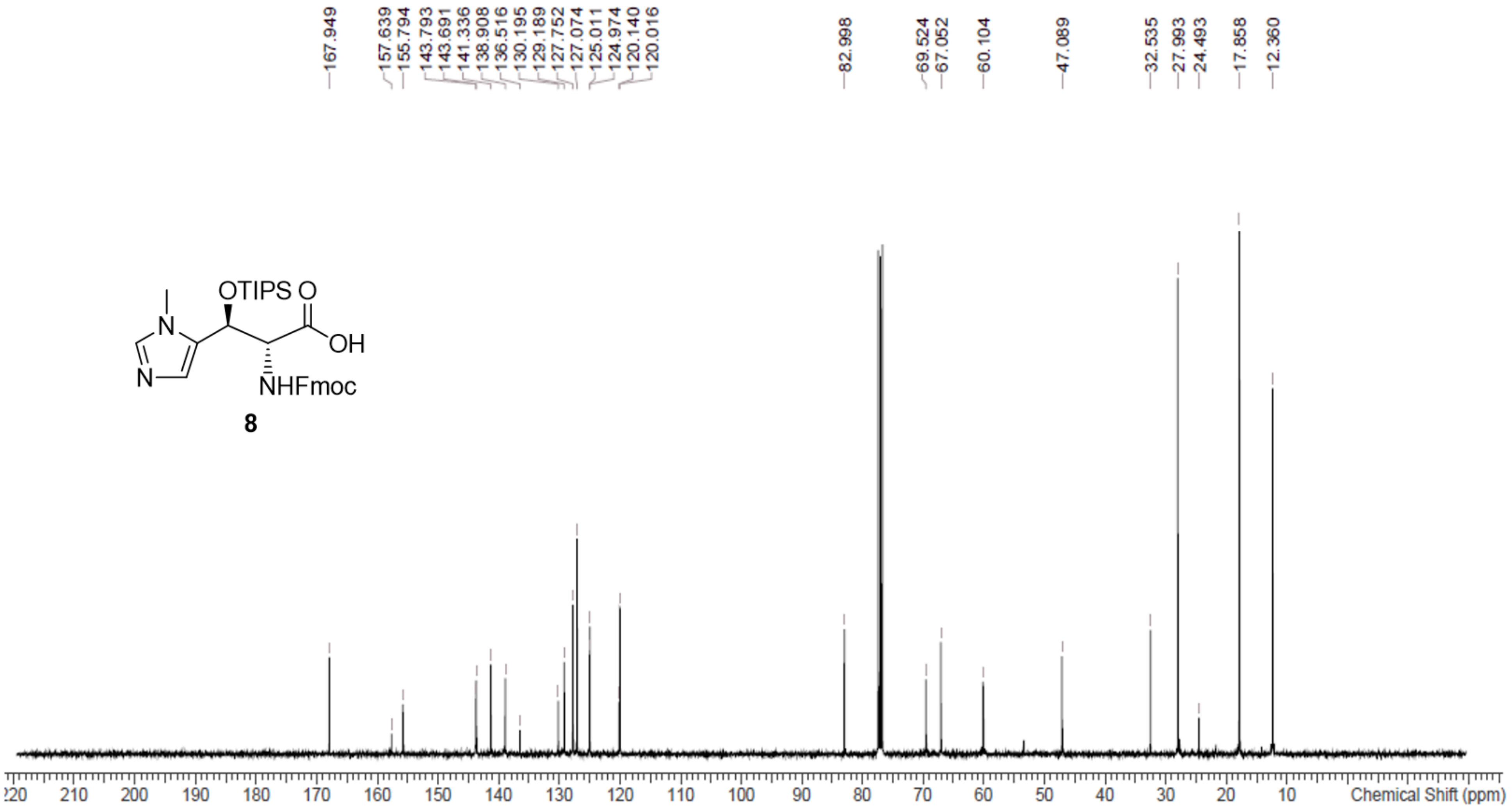

Figure S 61. ^{13}C NMR of compound 8, 150 MHz, $CDCl_3$.

10.3.1 NMR data of synthetic Corramycin

Table S 7. NMR-data comparison (700 MHz, D_2O+FA) for natural and isolated Corramycin (**1**).

position	δ_H, mult (*J* in Hz) natural	δ_H, mult (*J* in Hz) synthetic	δ_C natural	δ_C synthetic
**1**	1.14, br d (6.4)	1.14, d (6.3)	18.1	18.1
**2**	3.97, m	3.97, m	67.9	67.8
**3**	3.96, m	3.96, m	74.7	74.6
**4**	-	-	174.2	174.3
**5**	5.02, d (*9.6*)	5.03, d (*9.7*)	55.3	55.2
**6**	5.21, d (*9.5*)	5.21, d (*9.5*)	63.9	63.98
**7**	-	-	132.2	132.1
**8**	7.47, br s	7.47, s	118.3	118.6
**9**	[a]	[a]	136.2	136.3
**10**	3.98, s	3.99, s	33.9	34.0
**11**	-	-	170.4	170.3
**12**	3.58, m[b]	3.58, m[b]	38.3	38.2
**13**	3.56, m[b]	3.57, m[b]	78.6	78.6
**14**	3.32, s	3.32, s	57.0	57.0
**15**	4.03, m	4.04, m	70.2	70.1
**16**	4.56, m	4.57, m	68.4	68.3
**17**	-	-	173.6	173.5
**18**	3.03, s	3.03, s	32.2	32.1
**19**	5.36, dd (*10.8; 5.1*)	5.37, dd (*10.8; 5.1*)	59.7	59.8
**20a**	3.08, dd (*14.4; 11.2*)	3.08, dd (*14.4; 11.2*)	32.8	32.8
**20b**	3.44, dd (*14.4; 5.5*)	3.45, dd (*14.4; 5.0*)		
**21**	-	-	137.0	136.9
**22a, 22b**	7.38, m	7.38, m	128.6	128.6
**23a, 23b**	7.33, m	7.34, m	129.0	128.9
**24**	7.31, m	7.31, m	126.8	126.8
**25**	-	-	172.3	172.3
**26a**	3.99, m	3.98, m	42.5	42.4
**26b**	4.16, d (*17.0*)	4.12, d (*17.0*)		
**27**	-	-	170.7	170.7
**28**	4.55, m	4.56, m	60.6	60.6
**29**	-	-	71.9	71.9
**30, 31**	1.27-1.32, m[b]	1.28-1.32, m[b]	25.0, 25.2/ 25.9, 26.0[c]	24.9, 25.1/ 25.7, 25.8[c]

**32**	-	-	171.3	171.3
**33**	4.60, m	4.61, m	55.3	55.3
**34**	3.89, m	3.88, m[b]	61.1/61.1[b]	60.9/61.0[b]
**35**	-	-	171.3	171.3
**36**	4.42, s	4.42, s	60.7	60.7
**37**	-	-	71.7	71.7
**38, 39**	1.27-1.32, m[b]	1.28-1.32, m[b]	25.0, 25.2/ 25.9, 26.0[c]	24.9, 25.1/ 25.7, 25.8[c]
**40**	-	-	171.0	170.9
**41**	4.48, m	4.48, m	52.3	52.1
**42**	1.67, m[e]	1.68, m[e]	39.6	39.5
**43**	1.67, m[e]	1.68, m[e]	24.2	24.1
**44a**	0.89, d (*5.3*)	0.89, d (*5.8*)	20.6	20.5
**44b**	0.94, d (*5.3*)	0.95, d (*5.7*)	22.2	22.0
**45**	-	-	174.2	174.1
**46**	4.45, m	4.53, m	55.4	54.8
**47a**	3.89, m	3.88, m[b]	61.1/61.1[b]	60.9/61.0[b]
**47b**	3.93, dd (*11.7, 5.4*)	3.95, m		
**48**	-	-	173.7	173.0

[a]no proton signal detectable (H9 only detectable without addition of FA. δ_H = 7.90 ppm, δ_C = 139.1 ppm, D_2O, 700 MHz); [b]overlapping signals

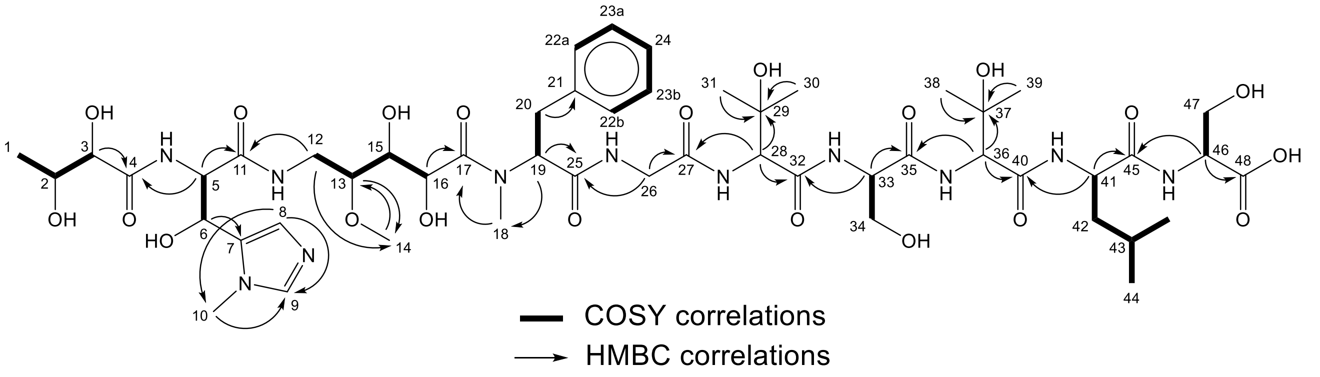

Figure S 62. Key 2D NMR correlations for Corramycin (1).

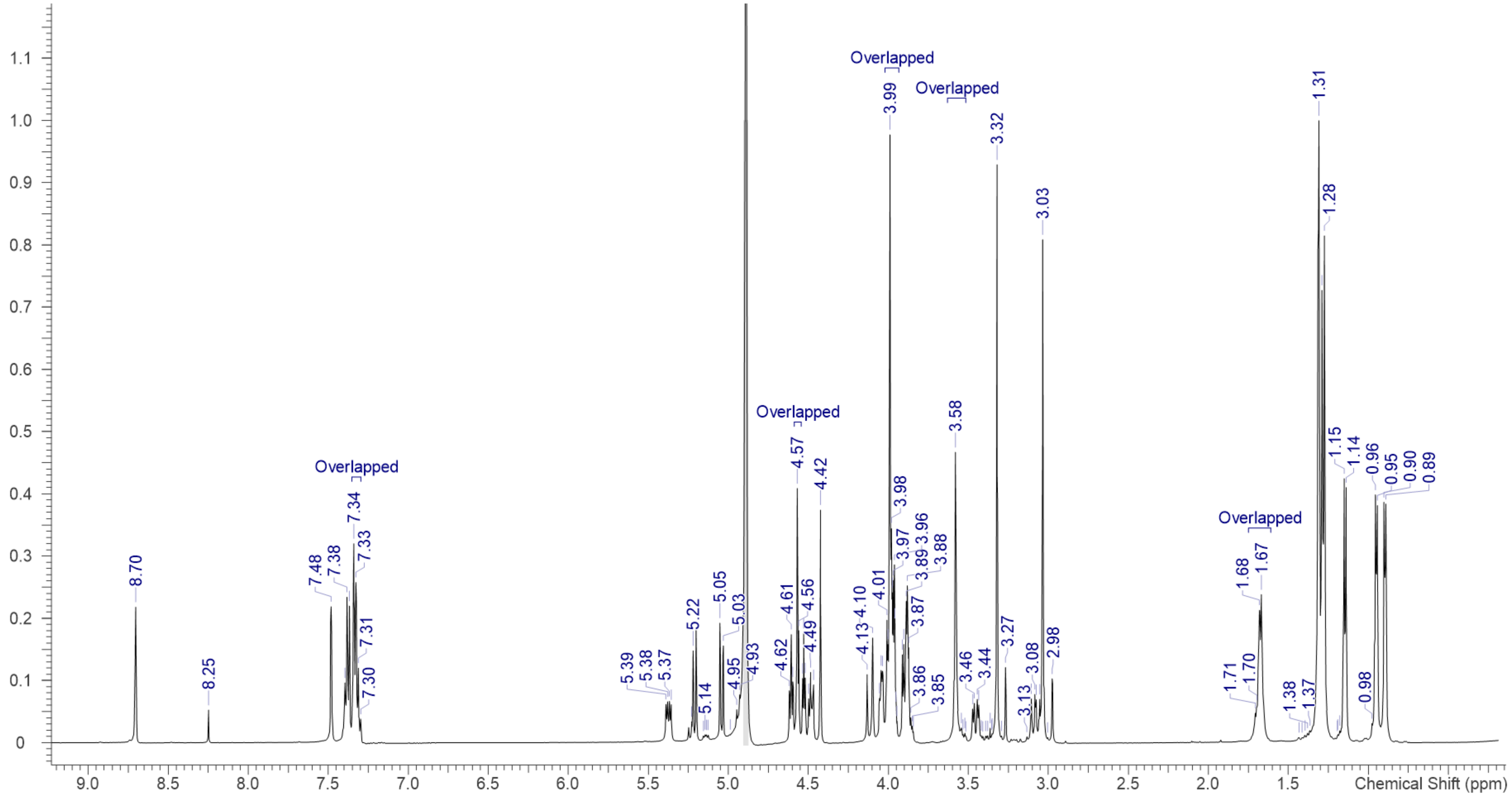

Figure S 63. ^{1}H NMR of synthetic Corramycin (1), 700 MHz, D_2O + FA.

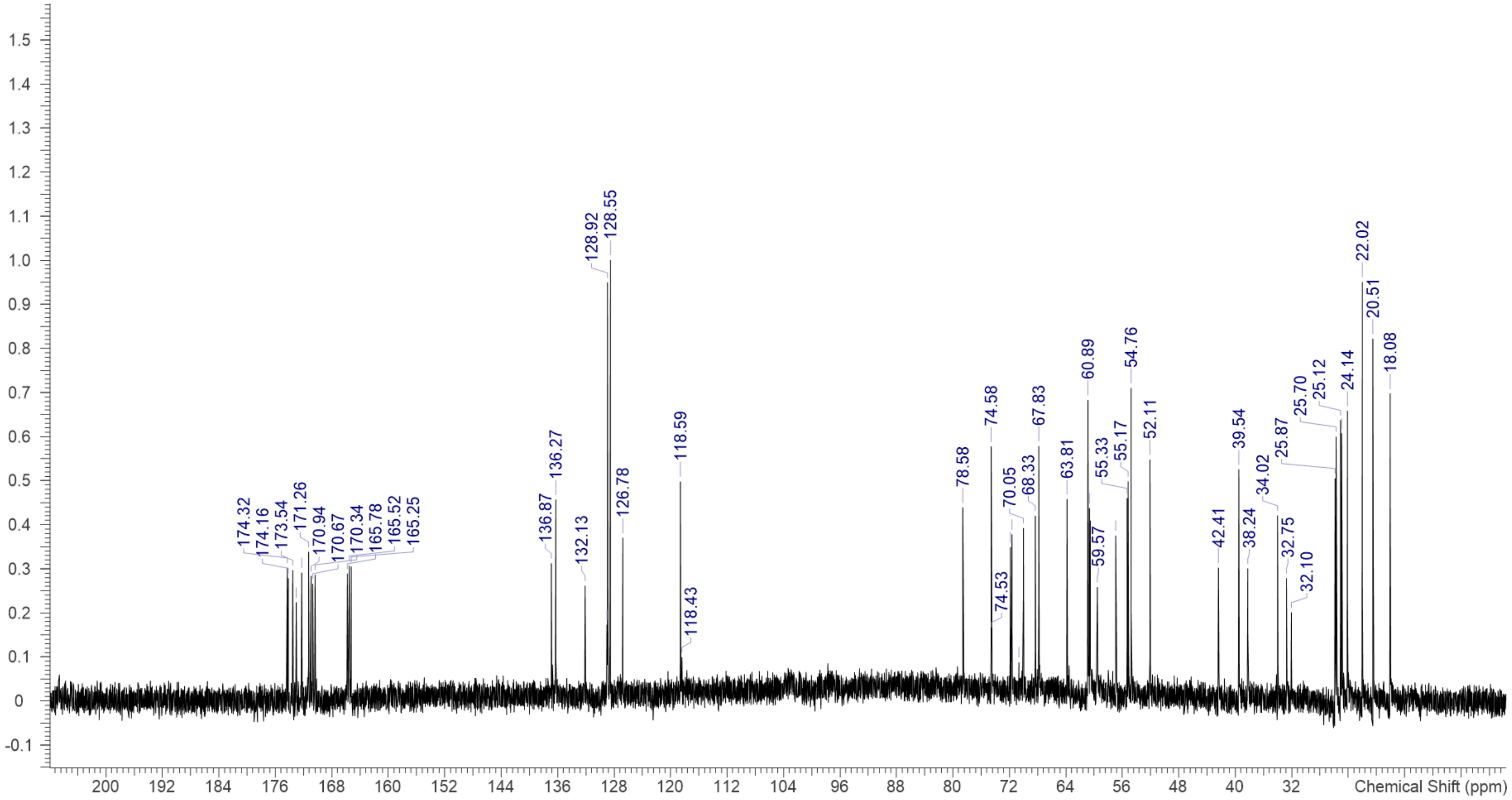

Figure S 64. ^{13}C NMR of synthetic Corramycin (1), 700 MHz, D_2O + FA.

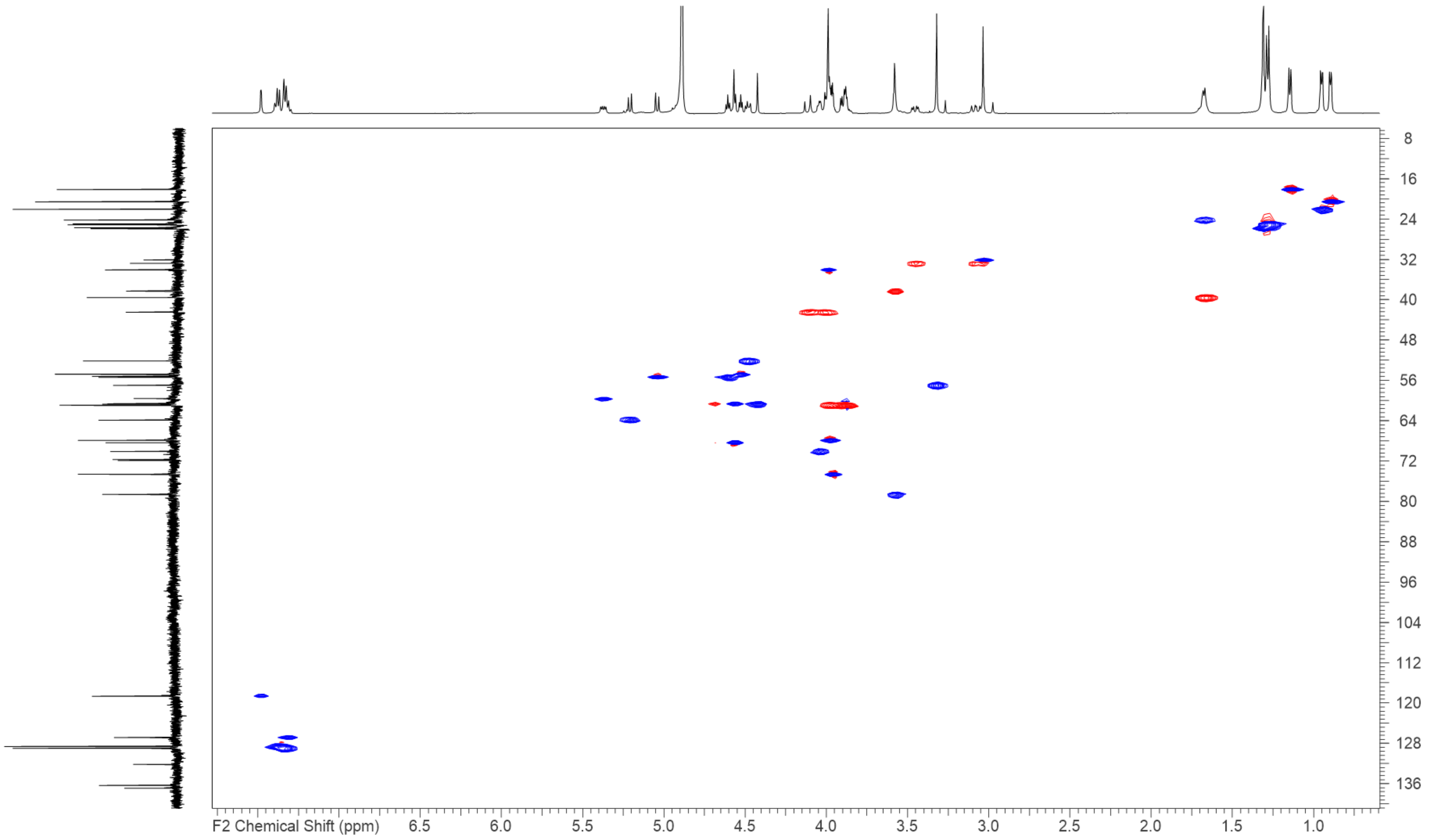

Figure S 65. HSQC NMR of synthetic Corramycin (1), 150/700 MHz, D_2O + FA.

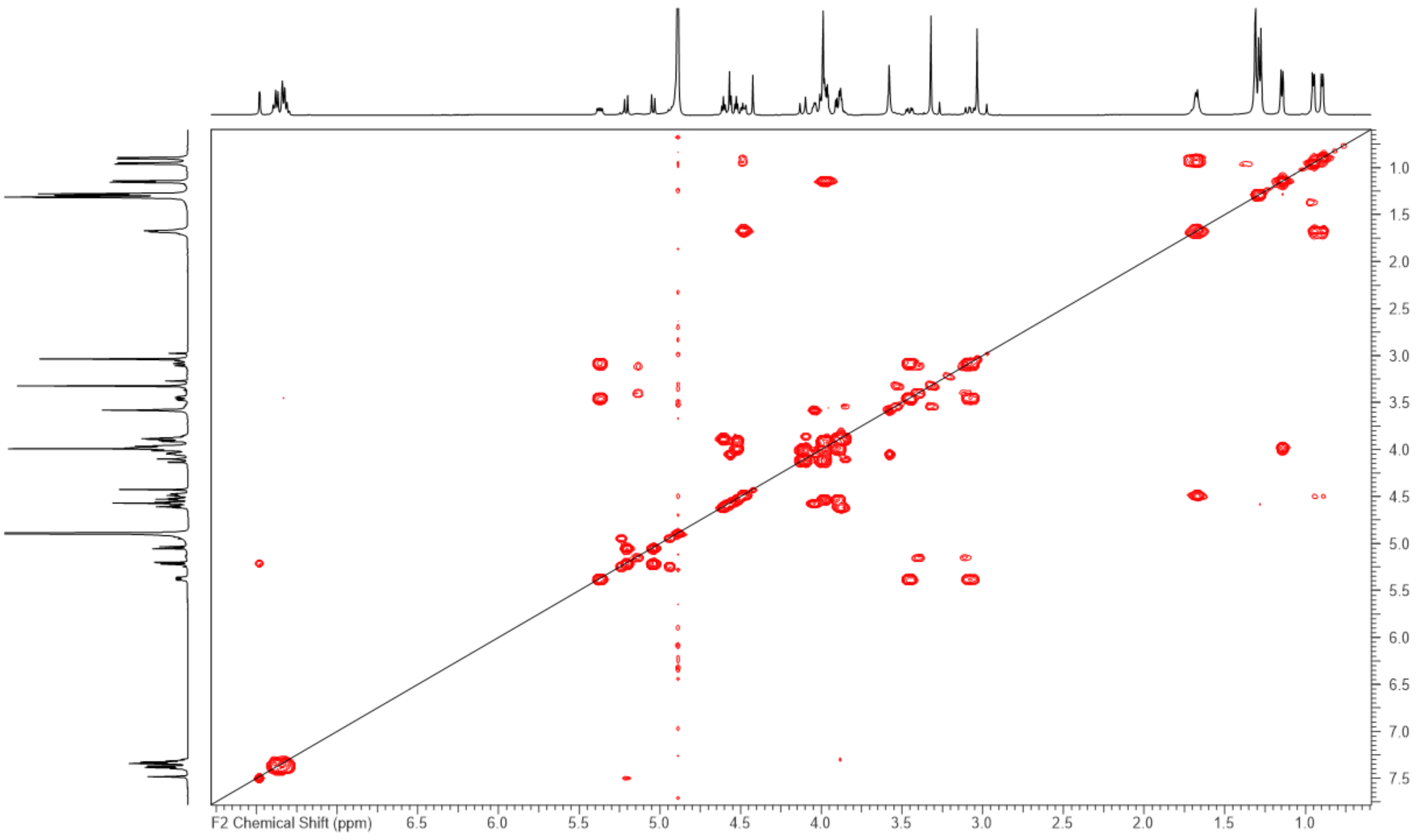

Figure S 66. COSY NMR of synthetic Corramycin (1), 700 MHz, D_2O + FA.

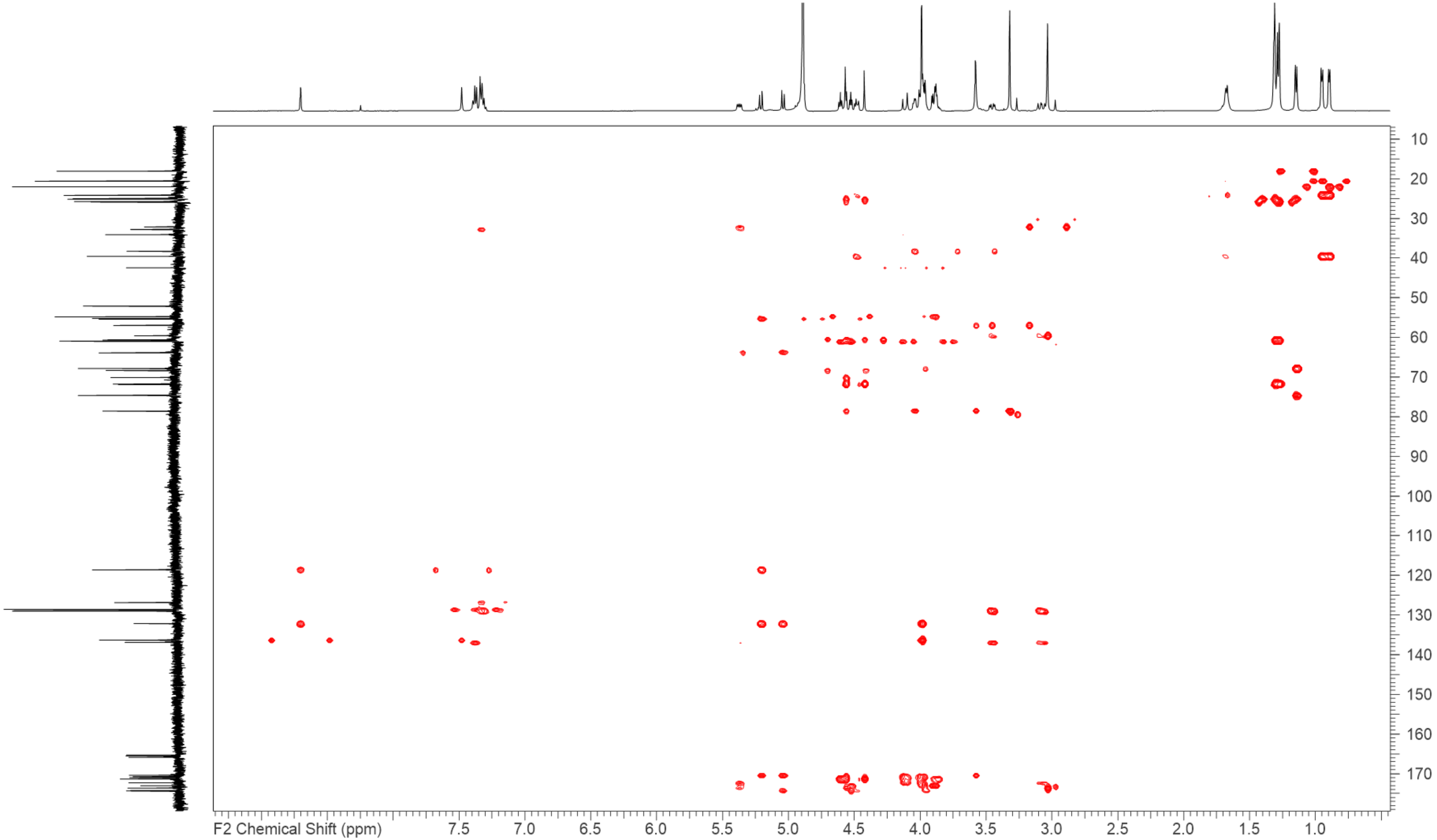

Figure S 67. HMBC NMR of synthetic Corramycin (1), 150/700 MHz, D_2O + FA.

10.3.2 Comparison: ^{1}H NMR and 13C NMR of synthetic and natural Corramycin

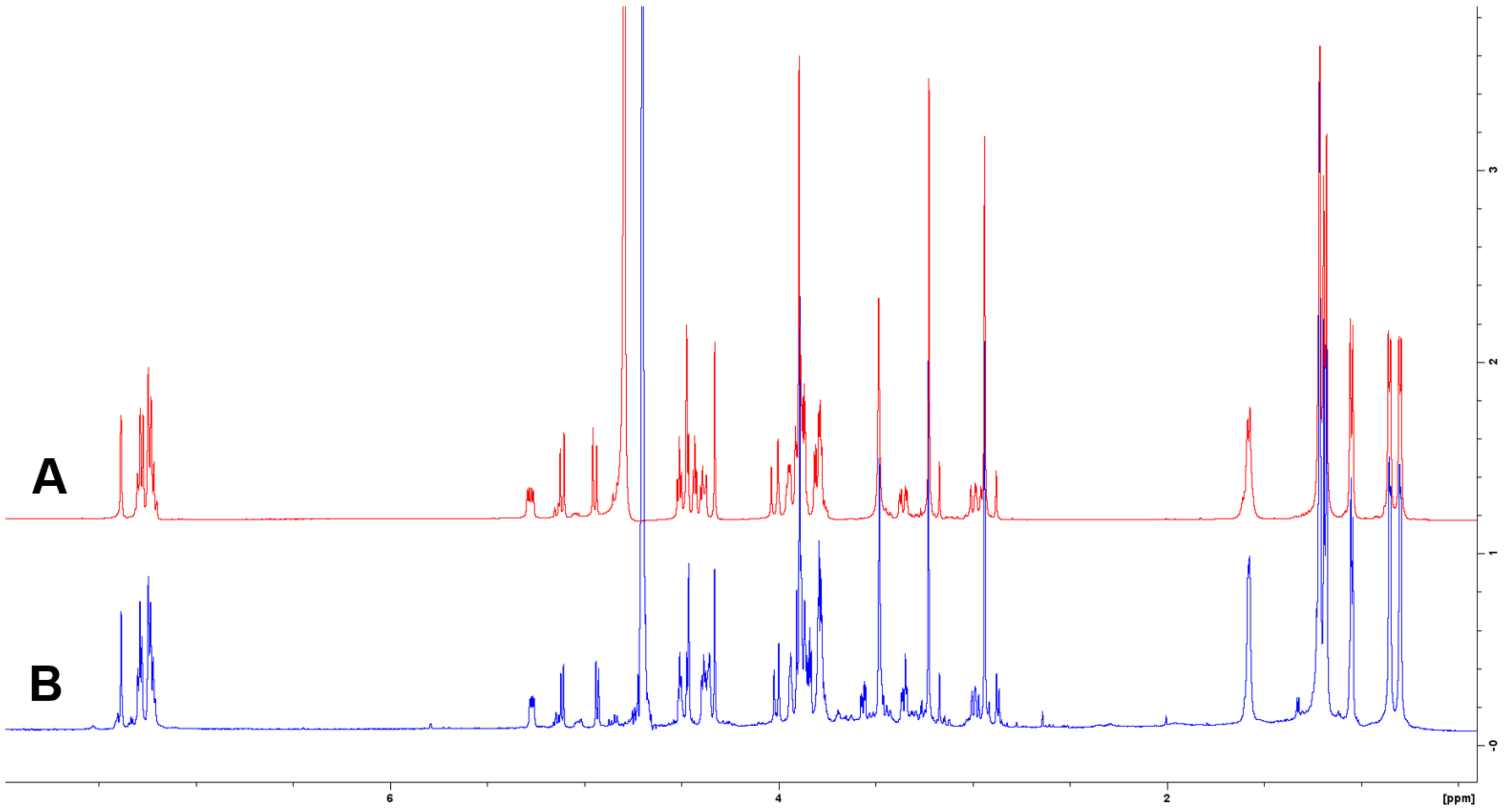

Figure S 68. ^{1}H NMR of synthetic (A, in red) and natural (B, in blue) Corramycin (1), 700 MHz, D2O + FA.

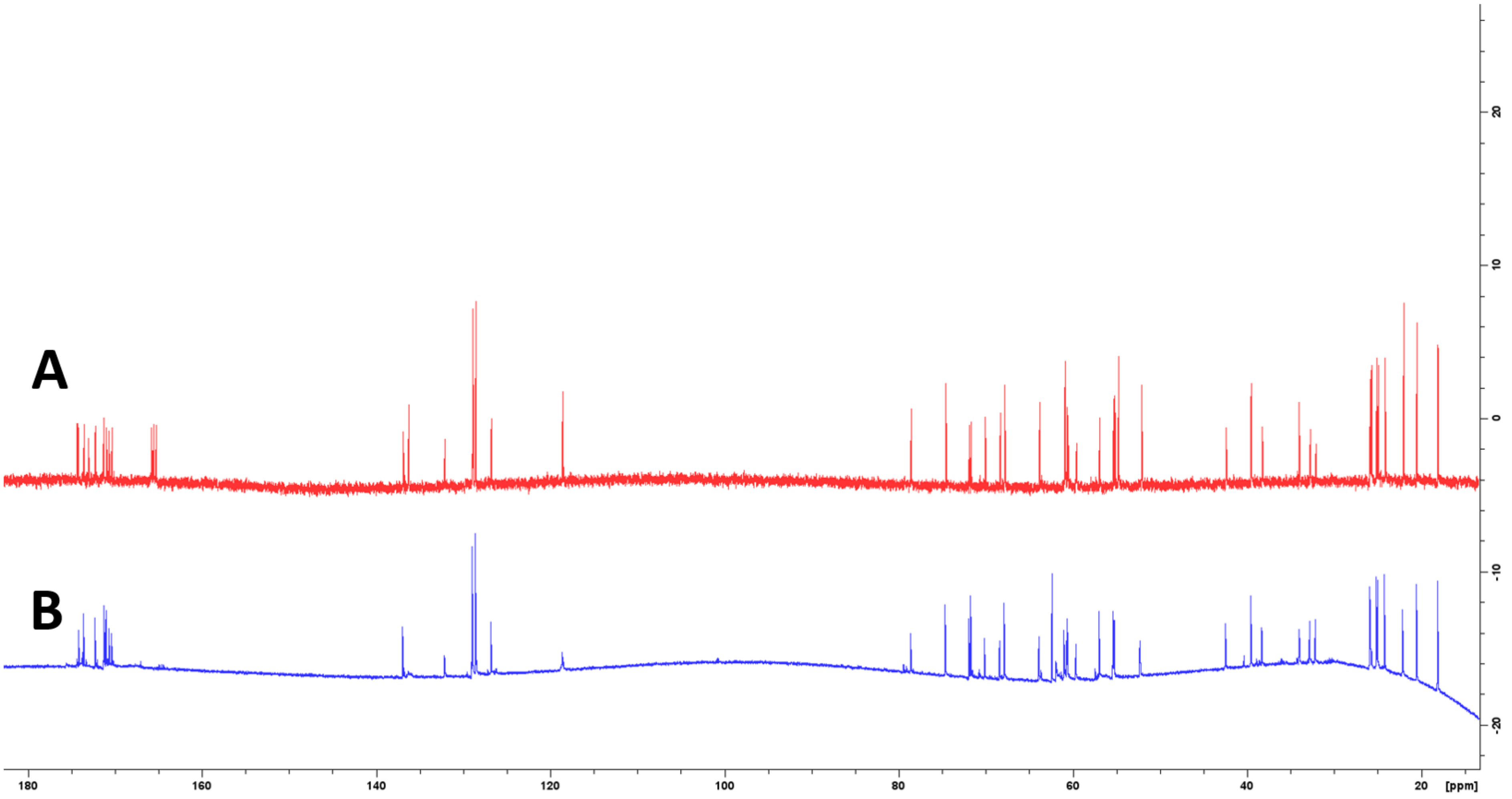

Figure S 69 ^{13}C NMR of synthetic (A, in red) and natural (B, in blue) Corramycin (1), 700 MHz, D2O + FA.

11. Resistance development of *E. coli* towards Corramycin

11.1 Time-kill-curve (TKC) experiment

TKCs of Corramycin against *E. coli* ACTCC25922 in CAMHB full medium and M9 minimal medium (containing the following supplements, 1 % glycerol, 2 mM $MgSO_4$ and 0.1 mM $CaCl_2$) are shown in Figure S 70. Bacterial inoculi were prepared by inoculation of 20 mL fresh CAMHB medium from an overnight culture and cultivation for 2 h until exponential growth phase was reached. CAMHB and M9 medium without Corramycin, with 4x and with 8x MIC of Corramycin were subsequently inoculated with 5×10^5 CFU mL^{-1} of *E. coli* ATCC25922 (Table S 4). The cultivation was performed for 48 h at 37 °C under constant shaking. Subsequently, 1 mL of the respective culture was diluted 1:10 in distilled water, immediately seeded on gelose and incubated overnight at 37 °C, followed by cell counting.

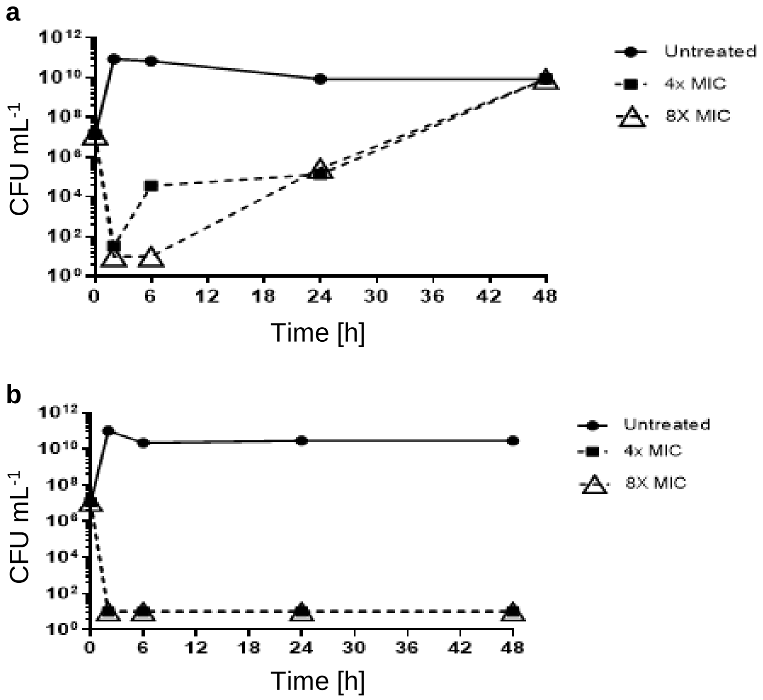

Figure S 70. Time-kill-curves (TKCs) for Corramycin at 4x MIC and 8x MIC using *E. coli* ATCC25922 grown in either CAMHB (a) or M9 medium (b).

11.2 Determination of the frequency of resistance (FoR)

The solid MIC of Corramycin was first evaluated on solid media by preparing TSB agar (1.5 % (w/v) trypton, 0.5 % (w/v) peptic digest of soya meal, 0.5 % (w/v) NaCl, 1.5 % (w/v) agar, pH 7.4) in 6-well plates with serial dilutions of Corramycin. Two spots of 1×10^5 CFU and 1×10^6 CFU *E. coli* ATCC25922 were seeded on the agar surface and incubated overnight at 37 °C. The solid MIC endpoint was determined as the lowest concentration of Corramycin at which there was no visible growth from these spots. Then, TSB agar plates containing the 4x solid MIC concentrations of Corramycin were prepared and seeded with 100 µL of a bacterial preparation concentrated by centrifugation, removal of parts of the supernatant and resuspending the cells, to 1×10^8 or 1×10^9 CFU per plate. The exact inoculum concentration was confirmed by serial dilution on agar plates and by counting of the grown colonies. The frequencies of resistance were calculated by counting the number of colonies growing on the plate containing Corramycin divided by the evaluated number of CFU in the inoculum (Table S 8)

Table S 8. Frequency of resistance (FoR) of *E. coli* ATCC25922 against Corramycin at 4 × MIC in MHB or the minimal medium M9.

	Medium	FoR (4 × MIC)
E. coli ATCC25922	MHB	3.8×10^{-6}
	M9	$<3 \times 10^{-10}$

12. Isolation of genomic DNA from *E. coli*, Illumina sequencing and sequence analysis

Individual *E. coli* colonies from the FoR or TKC experiments were picked and grown for 16 h in LB medium in shaking flasks at 37 °C. To prepare genomic DNA for whole genome sequencing, we used an Epicentre Masterpure DNA purification kit (Lucigen) according to the manufacturer's instructions. The DNA was resuspended in EB buffer (Qiagen) and subsequently diluted in molecular biology grade water to 0.2 µg μL^{-1}. Genomic DNA libraries were prepared using Illumina Nextera XT kit and Nextera XT index primers. The libraries were sequenced on an Illumina MiSeq platform (in-house) using the MiSeq V3 600 cycle reagent kit. The mutations were identified by mapping the genome sequence reads of the mutant *E.* coli to the wild type ATCC25922 *E. coli* strain genome sequence (GenBank accession: GCA_000401755.1).

13. λ red-mediated gene deletions of *sbmA* and *yejEF*

13.1 *sbmA* and *yejEF* single gene deletion

The deletions of *sbmA* and *yejEF* in *E. coli* were performed by homologous recombination using the λ-red recombinase method (according to standard protocols) to insert a recombinase flippase (FLP) recognition target (FRT) flanked by a Tetracycline or a Kanamycin resistance cassette obtained from pBR322 or pKD13 plasmids, respectively, into the target genes. Resistance cassette amplification was performed via PCR using oligonucleotides containing 18 bp sequences homologous to the resistance cassette on the respective plasmid, plus about 32 bp homologous sequences to the 5' and 3' untranslated region (UTR) of the *sbmA* or *yejEF* genes (Table S 9). The PCR was performed following the manufacturers protocol (Phusion polymerase kit; Thermo Fisher). The PCR-product was transformed into *E. coli* ATCC25922 according to standard protocols[13] and clones in which recombination was successful, were identified by selection using the respective antibiotic. Integration of the respective constructs into the genome was confirmed by PCR (Table S 9). Next, the thermosensitive plasmid pCP20, bearing the flippase encoding gene *flp* was independently transformed into the PCR-confirmed clones *E. coli* ATCC25922-*yejEF::kanR* and *E. coli* ATCC25922-*sbmA::TetC* and resulting clones were selected on Chloramphenicol or Ampicillin. Expression of the Flp gene allowed for the recombination of the FRT sequences flanking the resistance cassette, thereby deleting it and leaving a 81 bp long FRT sequence scar in the genome. The deletion of the resistance cassette was confirmed *via* PCR.

Table S 9. Oligonucleotides used for antibiotic resistance cassette amplification, *smbA* and *yejEF* gene deletions and verification of the deletions.

Oligonucleotides	Sequence	Template	Purpose
1	CCCTTTTCAAACTACAATCCTTTTTAAGCGTGTAGGCTGGAGCTGCTTC	Plasmid pKD13	Amplification of the *kanR* resistance cassette with *yejF* homologous sequences
2	CCGATTGAAAAATTTTGGTGTGGCAATTGCCGAACCCTTTTCAAACTAC	Plasmid pKD13	Amplification of the *kanR* resistance cassette with *yejF* homologous sequences
3	CCGCGTATAGACTTTGAGGGACGTTAATGATTCCGGGGATCCGTCGAC	Plasmid pKD13	Amplification of the *kanR* resistance cassette with *yejE* homologous sequences
4	CAGTGATATCAGCTATACGCTGGTCGATCCGCGTATAGACTTTGAGGGAC	Plasmid pKD13	Amplification of the *kanR* resistance cassette with *yejE* homologous sequences
5	GGCTTACTGGGCTACGAAGC	gDNA *of E. coli* ATCC25922-*yejE::kanR*	Verification of the *yejE* deletion
6	CCGATTGAAAAATTTTGGTGTG	gDNA *of E. coli* ATCC25922-*yejF::kanR*	Verification of the *yejF* deletion
7	CAGTCATAGCCGAATAGCCT	gDNA *of E. coli* ATCC25922-*yejF*	Verification of the *kanR* deletion
8	CGGTGCCCTGAATGAACTGC	gDNA *of E. coli* ATCC25922-*yejE*	Verification of the *kanR* deletion
9	CGATAAGAAGTTAGCAGGAGTGCATATGGAAACCATTATTATCATGAC	Plasmid pBR332	Amplification of the *TetC* resistance cassette with *sbmA* homologous sequences
10	CTTCTCCTTTTTAGCTCAAGGTATGGGTTACGCGCATTCACAGTTCTCCGC	Plasmid pBR332	Amplification of the *TetC* resistance cassette with *sbmA* homologous sequences
11	CAGGCTGGCGAACTAAGC	gDNA *of E. coli* ATCC25922-*sbmA*	Verification of the *TetC* deletion
12	CGCCCAGGCATCGCGGTC	gDNA *of E. coli* ATCC25922-*sbmA*	Verification of the *TetC* deletion

13.2 Complementation of SbmA and YejEF in the *E. coli* deletion mutant

The *sbmA* gene was amplified via PCR using genomic DNA from *E. coli* ATCC25922 as template sequence, thereby introducing *Nco*I and *Hind*III restriction site recognition sequences up- and downstream of the amplified *sbmA* sequence. Next, the PCR product was cloned into the pTrc99A plasmid (GeneBank accession: U13872.1) by restriction/ligation-based cloning techniques according to standard protocols[13]. In case of YejEF complementation, we introduced the entire operon *yejABEF* to ensure a balanced co-expression of all genes regulated by the same promoter system. The *yejABEF* coding sequences were amplified from genomic DNA of *E. coli* ATCC25922 in three independent PCRs according to standard protocols[13] and cloned into the pBAD202 plasmid, an arabinose-dependent expression vector (ThermoFisher) according to the manufacturer's instructions in the pBAD202 directional pTOPO expression kit (ThermoFisher). Transformation of the resulting plasmids into *E. coli* ATCC25922 Δ*sbmA*/Δ*yejEF* and selection on their respective resistance marker (Ampicillin for pTrc99A-*sbmA*, Kanamycin for pBAD202-*yejABEF*) ensured maintenance of the plasmids. Gene expression of *yejABEF* and *sbmA* was induced using 0.1 % arabinose or 200 µM IPTG, respectively. The induction was performed subsequently after inoculation of the 96 well plates and the MIC experiment was performed according to the previously described protocol.

14. Investigation of the Corramycin *in-vivo* activity in infected mice test animals and experimental ethics

All mouse experiments were carried out in strict accordance with the recommendations in the Drug Disposition/Montpellier DSAR Operational Center (Toulouse) and approved by the Ethical Committee (under protocol CEEA-122 2014-53). Twelve weeks old male Swiss albino mice (Charles River Laboratories, Janvier Laboratories) with a body weight of 30 to 35 g were used. The animals were group housed in plastic cages containing appropriate bedding, with a 12 h light and 12 h dark cycle. Food pellets were provided *ad libitum* and water was available at any time for each animal via water bottles. A mixture of isoflurane/O_2 (5%, (v/v)) was used exclusively at the time of intravenous treatment with Corramycin as a flash anesthetic and every effort was made to minimize suffering of the animals.

14.1 Infection of the test animals and treatment with Corramycin *in vivo*

For the *in vivo* infection and antibiotic treatment experiments a total of 75 male Swiss albino mice, divided into five groups with 15 animals, were used. Before infecting the test animals, the weight of each animal was determined (Suppl. Raw Data 1). Infection of the test animals with *E. coli* ATCC35218 was induced by injecting 200 µL of bacterial inoculum diluted to 1.10^8 CFU mL^{-1} in 0.9 % NaCl and 5 % mucin for a total of 2.10^7 CFU/mouse intraperitoneally. After 1 h and 3 h post infection, the animals were treated with 5 mL kg^{-1} body weight of the respective treatment solution. Group 1 was treated with 0.9 % NaCl (negative control), group 2 with Tienam (15 mg kg^{-1} Imipenem; positive control) and groups 3-5 with total doses of 10 mg kg^{-1}, 20 mg kg^{-1} and 30 mg kg^{-1} Corramycin, respectively. After 4 h post infection, five animals per group were killed by cervical dislocation and blood, spleen and kidneys were sampled, mixed, 1 g of tissue was homogenized in 1 mL water, then serial diluted and spread on gelose in order to determine the *E. coli* ATCC35218 CFU load. The survival and body weight of the remaining mice was monitored at 3, 6, 24, 48, 72 and 96 h post-treatment. All mice pictures were adapted from BioRender.com (2022).

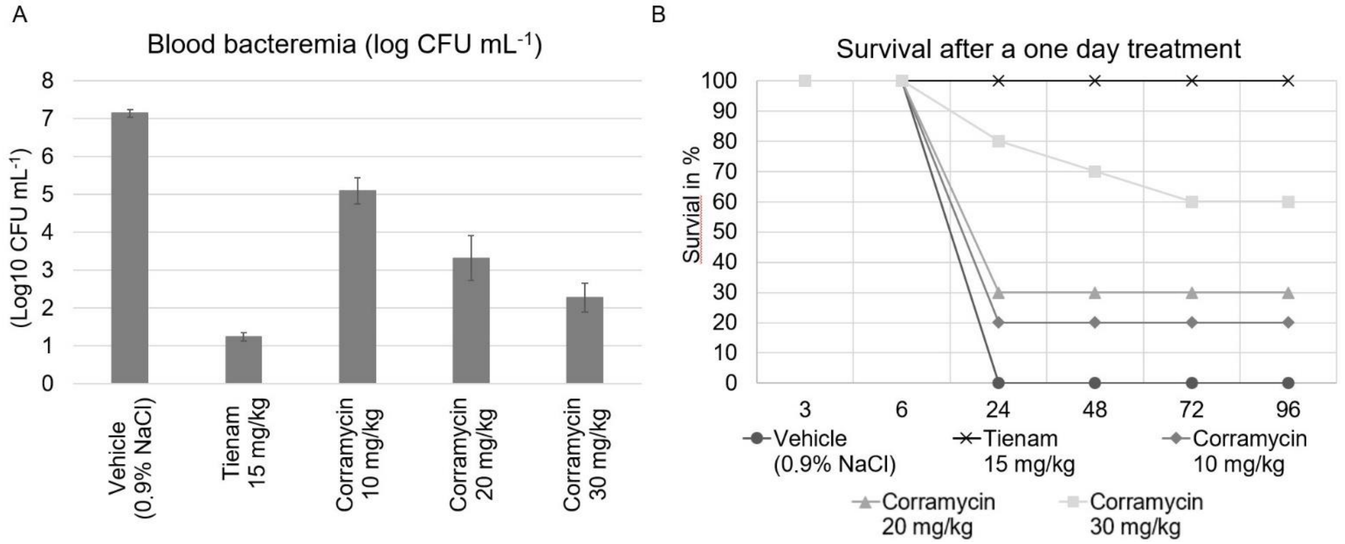

Figure S 71. A) Blood bacteremia experiment. *E. coli* ATCC35218 CFU load after treatment of infected mice with 10 mg kg^{-1}, 20 mg kg^{-1} or 30 mg kg^{-1} Corramycin. B) Survival of the *E. coli* ATCC35218 infected mice in %, after one day of treatment with either 0.9 % NaCl (negative control), Tienam (15 mg kg^{-1} Imipenem; positive control) or Corramycin in three different doses (10 mg kg^{-1}, 20 mg kg^{-1} or 30 mg kg^{-1}).

14.2 Corramycin elimination experiment

Native Corramycin systemic exposure was determined following a single intravenous (30 mg kg^{-1} expressed as active compound) bolus administration to male Swiss mice. Before sampling, animals were anaesthetized. For plasma analysis, blood samples were collected from the abdominal aorta of three animals per sampling time (5 min, 0.5, 1, 2, 4, 6, 8 and 24 h post administration) and transferred into glass tubes containing lithium heparin as anticoagulant. After 10 min centrifugation at 2.500 x g and +4°C, plasma was treated immediately by a Phenyl Methyl Sulfonyl Fluoride Methanolic (PMSF) solution (5 mg mL^{-1}) in the proportion of one volume of PMSF to 10 volumes of plasma. Then samples were frozen at -20 °C until analysis.

Lung, brain, kidney, heart and liver were collected at each sampling time (5 min, 0.5, 1, 2, 4, 6, 8 and 24 h post administration) pooled per sampling time, weighed and frozen at -20 °C until analysis. Native Corramycin was quantified in the plasma and tissues using an exploratory LC/MS-MS assay method in Early Bioanalysis Group of Toulouse (Montpellier DSAR Operational Center), France. (Suppl. Raw Data 1). The pharmacokinetic parameters were calculated from the arithmetic mean of the plasma concentrations or the tissue concentrations (lung, brain, kidney, heart and liver) using the program WinNonLin 5.2, non-compartmental models 200 and 201.

14.3 Determination of the effective dose for 50 % of the population (ED_{50})

E. coli ATCC25922Δ*yejEF, E. coli* ATCC25922Δ*sbmA and E. coli* ATCC25922Δ*yejEF/*Δ*sbmA* were validated in terms of virulence, to ensure that lethality occurred at the same inoculum compared to *E. coli* ATCC25922 wild type strain. The mice were infected using bacterial doses of 1, 5, 10, 20 and 40 mg kg^{-1} divided into two doses a day. After 1 h and 3 h post the last bacterial infection, Corramycin was administered and 24 h later blood, spleen and liver were collected. 1 g of tissue was homogenized in 1 mL water, then serial diluted and spread on gelose in order to determine the *E. coli* CFU load. The ED_{50}s of the compound could not be evaluated in blood and liver because the tested doses were very efficacious for the wild type *E. coli* and the single deletion mutants. However, spleen samples did provide a dose response with sufficient range of effect to calculate ED_{50}. The deletion of SbmA and YejEF in *E. coli* resulted in a mild decrease of susceptibility to Corramycin, affording ED_{50}s of 23.3 mg kg^{-1} and 21.4 mg kg^{-1}, respectively, compared to the wild type control strain (6.1 mg kg^{-1}). The double deletion of SbmA and YejEF, however, lead to a significant increase in resistance with an ED_{50} of >40 mg kg^{-1}.

References

[1] H. Frank, G. J. Nicholson, E. Bayer, *J. Chromatogr.* **1978**, *167*, 187.
[2] D. A. Evans, A. S. Kim, R. Metternich, V. J. Novack, *J. Am. Chem. Soc.* **1998**, *120*, 5921.
[3] T. Weber, K. Blin, S. Duddela, D. Krug, H. U. Kim, R. Bruccoleri, S. Y. Lee, M. A. Fischbach, R. Muller, W. Wohlleben et al., *Nucleic Acids Res.* **2015**, *43*, W237-43.
[4] F. Madeira, Y. m. Park, J. Lee, N. Buso, T. Gur, N. Madhusoodanan, P. Basutkar, A. R. N. Tivey, S. C. Potter, R. D. Finn et al., *Nucleic Acids Res* **2019**, *47*, W636-W641.
[5] M. Rottig, M. H. Medema, K. Blin, T. Weber, C. Rausch, O. Kohlbacher, *Nucleic Acids Res.* **2011**, *39*, W362-7.
[6] N. Ziemert, S. Podell, K. Penn, J. H. Badger, E. Allen, P. R. Jensen, *PLoS ONE* **2012**, *7*, e34064.
[7] E. Quevillon, V. Silventoinen, S. Pillai, N. Harte, N. Mulder, R. Apweiler, R. Lopez, *Nucleic Acids Res.* **2005**, *33*, W116-W120.
[8] D. Lando, D. J. Peet, J. J. Gorman, D. A. Whelan, M. L. Whitelaw, R. K. Bruick, *Genes Dev.* **2002**, *16*, 1466.
[9] A. Noma, R. Ishitani, M. Kato, A. Nagao, O. Nureki, T. Suzuki, *J. Biol. Chem.* **2010**, *285*, 34503.
[10] K. Hiom, *Curr. Biol.* **2009**, *19*, R523-5.
[11] H. Motamedi, A. Shafiee, S. J. Cai, S. L. Streicher, B. H. Arison, R. R. Miller, *J. Bacteriol.* **1996**, *178*, 5243.
[12] a) C. T. Walsh, H. W. Chen, T. A. Keating, B. K. Hubbard, H. C. Losey, L. S. Luo, C. G. Marshall, D. A. Miller, H. M. Patel, *Curr. Opin. Chem. Biol.* **2001**, *5*, 525; b) E. G. Johnson, S. B. Krasnoff, D. R. Bignell, W. C. Chung, T. Tao, R. J. Parry, R. Loria, D. M. Gibson, *Mol. Microbiol.* **2009**, *73*, 409.
[13] J. Sambrook, D. W. Russell, *Molecular cloning: A laboratory manual*, Cold Spring Harbor Laboratory Press, Cold Spring Harbor, NY, **2001**.
[14] E. Gasteiger, C. Hoogland, A. Gattiker, S. Duvaud, M. R. Wilkins, R. D. Appel, A. Bairoch in *The proteomics protocols handbook* (Ed.: J. M. Walker), Humana Press, Totowa, N.J., **2005**, pp. 571–607.
[15] T. J. McQuade, A. D. Shallop, A. Sheoran, J. E. Delproposto, O. V. Tsodikov, S. Garneau-Tsodikova, *Anal. Biochem.* **2009**, *386*, 244.
[16] a) M. Debono, M. Barnhart, C. B. Carrell, J. A. Hoffmann, J. L. Occolowitz, B. J. Abbott, D. S. Fukuda, R. L. Hamill, K. Biemann, W. C. Herlihy, *J Antibiot* **1987**, *40*, 761; b) M. Morikawa, Y. Hirata, T. Imanaka **2000**, *1488*, 211; c) J. Mares, J. Hajek, P. Urajova, J. Kopecky, P. Hrouzek, *PLoS ONE* **2014**, *9*, e111904.
[17] a) K. Arima, A. Kakinuma, G. Tamura, *Biochem. Biophys. Res. Commun.* **1968**, *31*, 488; b) F. Peypoux, M. T. Pommier, D. Marion, M. Ptak, B. C. Das, G. Michel, *J. Antibiot.* **1986**, *39*, 636; c) R. Maget-Dana, F. Peypoux, *Toxicology* **1994**, *87*, 151; d) E. H. Duitman, L. W. Hamoen, M. Rembold, G. Venema, H. Seitz, W. Saenger, F. Bernhard, R. Reinhardt, M. Schmidt, C. Ullrich et al., *Proc. Natl. Acad. Sci. USA* **1999**, *96*, 13294; e) K. Tsuge, T. Akiyama, M. Shoda, *J. Bacteriol.* **2001**, *183*, 6265; f) S. Yao, X. Gao, N. Fuchsbauer, W. Hillen, J. Vater, J. Wang, *Curr. Microbiol.* **2003**, *47*, 272.
[18] F. Yan, D. Auerbach, Y. Chai, L. Keller, Q. Tu, S. Hüttel, A. Glemser, H. A. Grab, T. Bach, Y. Zhang et al., *Angew. Chem. Int. Ed. Engl.* **2018**, *57*, 8754.
[19] W. C. Chan, P. D. White, *Fmoc solid phase peptide synthesis. A practical approach / edited by Weng C. Chan and Peter D. White*, Oxford University Press, Oxford, **2000**.
[20] a) M. Inoue, N. Shinohara, S. Tanabe, T. Takahashi, K. Okura, H. Itoh, Y. Mizoguchi, M. Iida, N. Lee, S. Matsuoka, *Nat. Chem.* **2010**, *2*, 280; b) J. E. Dettwiler, W. D. Lubell, *J. Org. Chem.* **2003**, *68*, 177.
[21] A. Solladie-Cavallo, J. L. Koessler, *J. Org. Chem.* **1994**, *59*, 3240.
[22] A. Solladie-Cavallo, M. C. Simon-Wermeister, J. Schwarz, *Organometallics* **1993**, *12*, 3743.
[23] A. Solladié-Cavallo, M. C. Simon, *Tetrahedron Lett.* **1989**, *30*, 6011.
[24] A. Solladié-Cavallo, B. Crescenzi, *Synlett* **2000**, *2000*, 327.
[25] A. Tanaka, K. Yamashita, *Chem. Lett.* **1981**, *10*, 319.
[26] U. Klein, K. Mohrs, H. Wild, W. Steglich, *Eur. J. Org. Chem.* **1987**, *1987*, 485.
[27] D. J. Smaltz, A. G. Myers, *J. Org. Chem.* **2011**, *76*, 8554.
[28] A. Solladié-Cavallo, J. L. Koessler, *J. Org. Chem.* **1994**, *59*, 3240.
